# Stereoselective Higher‐Order [10+4]‐ and [10+6] Cycloadditions Between Two Highly Unsaturated and Ambiphilic π‐Addends

**DOI:** 10.1002/chem.71093

**Published:** 2026-05-14

**Authors:** Jonas Faghtmann, Anne Kristensen, Fabien Fritz, Signe Sofie Pladsbjerg Andresen, Anne Rask Østergaard, Johannes Nygaard Lamhauge, K. N. Houk, Karl Anker Jørgensen

**Affiliations:** ^1^ Department of Chemistry Aarhus University Aarhus C Denmark; ^2^ Department of Chemistry and Biochemistry University of California Los Angeles California USA

**Keywords:** 3‐oxidopyridinium betaine, asymmetric organocatalysis, DFT calculations, higher‐order cycloaddition

## Abstract

A fundamental challenge in higher‐order cycloadditions (HOCs) is to control multiple layers of selectivity–peri‐, regio‐, diastereo‐, and enantioselectivity. Controlling these selectivities in HOCs is very challenging when they involve two highly unsaturated and ambiphilic π‐addends. Here, we study this complexity in enantioselective aminocatalyzed HOCs between isobenzofulvenes, reacting as 10π‐components, and 3‐oxidopyridinium betaines, reacting as 4π‐ or 6π‐components. The reaction generates three different products: an allowed [10+4] cycloadduct and two forbidden regioisomeric [10+6] cycloadducts. For the [10+6] reaction pathway, the 3‐oxidopyridinium betaine reacts as a 6π‐component with the fulvene scaffold in unprecedented cycloadditions. The outcome of these cycloadditions exhibits the challenge in controlling the peri‐ and regioselectivity in complex HOCs involving two highly unsaturated and ambiphilic π‐addends. We demonstrate that it is possible to optimize the cycloadditions toward the selective formation of the [10+4] cycloadduct or the [10+6] cycloadducts. Experimental investigations were conducted in combination with DFT calculations, in order to elucidate the origins of these selectivities.

## Graphical abstract

Aminocatalytically generated isobenzofulvenes and 3‐oxidopyridinium betaines act as ambiphilic reaction partners in orbital‐symmetry‐allowed [10+4] cycloadditions or formally symmetry‐forbidden concerted thermal [10+6] cycloadditions. The reaction furnishes three cycloadducts: one [10+4] cycloadduct and two regioisomeric [10+6] cycloadducts. Partial periselectivity is achieved through catalyst choice, although regioselectivity remains challenging to control. Nevertheless, the reaction proceeds in good combined yields with high diastereo‐ and enantioselectivity. DFT calculations were conducted to elucidate the reaction mechanism and the origin of the selectivity between the allowed [10+4] versus forbidden [10+6] pathways.

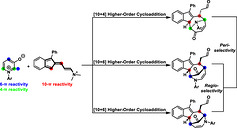

## 1 Introduction

Cycloaddition reactions are an important class of organic reactions, as exemplified by the frequent applications of these reactions for the synthesis of complex molecules ever since the discovery of the Diels‐Alder [4+2] cycloaddition in 1928 [1, 2, 3, 4, 5, 6, 7, 8, 9, 10, 11]. Extending cycloaddition chemistry beyond classical [4+2] cycloadditions involves the participation of larger conjugated π‐systems that engage more than six π‐electrons in a cyclic transition state (TS). Such transformations are commonly referred to as higher‐order cycloadditions (HOCs) [12]. These reactions hold considerable synthetic potential, as they enable the construction of diverse ring architectures beyond the six‐membered cycloadducts typically formed in [4+2] cycloadditions. Historically, HOCs have mostly been a theoretical curiosity, as early examples of HOCs were hampered by poor peri‐ and regioselectivity, and often with low yields of the cycloadducts formed [13, 14, 15, 16, 17]. However, during the last decade, asymmetric organocatalysis has found applications in generating new directions in HOCs, allowing for control of all layers of selectivity in HOCs–peri‐, regio‐, diastereo‐, and enantioselectivity. This has led to the development of various organocatalysed HOCs with generally high overall selectivity, and thus predictable product outcomes [18, 19, 20, 21]. Organocatalysed HOCs have begun to be reliable reactions in the synthetic chemist's toolbox, as exemplified by recent applications in natural product synthesis [22, 23, 24].

These accomplishments have inspired our group to pursue unexplored cycloaddition reactions, thereby expanding the boundaries of HOCs. These HOCs have predominantly been based on higher‐order enamine intermediates reacting with simpler π‐addends, thereby leading to controllable and predictable product outcomes [25, 26, 27, 28, 29, 30]. In contrast, if both reacting partners are highly unsaturated and display multifaceted reactivity profiles, controlling all layers of selectivity in HOCs becomes more troublesome. To challenge the contemporary boundaries for HOCs, we here explore a cycloaddition between a catalytically formed electron‐rich higher‐order enamine intermediate and a poly‐unsaturated electron‐deficient substrate.

**SCHEME 1:**
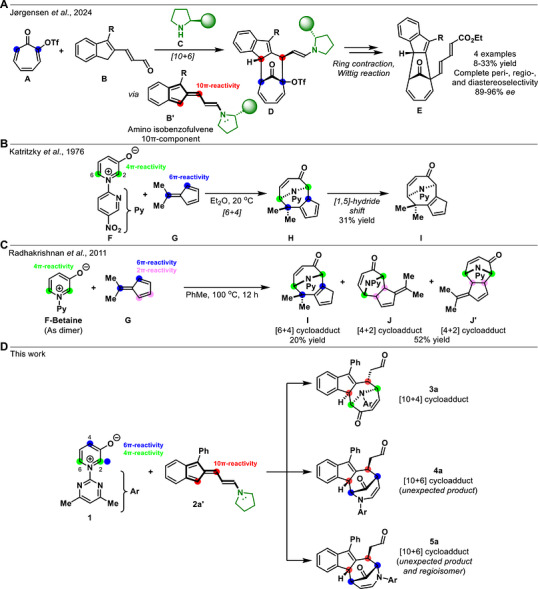
(A) The [10+6] cycloaddition between amino isobenzofulvene intermediate **B’** and tropone derivative **A**. (B) The first example of a HOC involving 3OPBs. (C) Issue of periselectivity in the reaction between 3OPBs and pentafulvenes. (D) This work: enantioselective higher‐order cycloaddition of 3OPBs with amino isobenzofulvene intermediate **2a’**. Py = 5‐nitropyridyl; Ar = 3,5‐dimethylpyrimidyl.

Electron‐deficient π‐addends containing more than 4π‐electrons are rather scarce in the literature. The most prevalent class of such π‐components are tropones, which have a long and rich history in HOCs [13, 14, 16, 17, 31, 32, 33, 34, 35]. Recently, we reported a [10+6] cycloaddition in which a troponoid derivative **A** reacts as a 6π‐component with an aminocatalytically generated isobenzofulvene **B’** (generated from indene carbaldehyde **B**) as a 10π‐component (Scheme 1A). [36] The [10+6] cycloadducts **D** undergo spontaneous ring contraction. After in situ Wittig transformation, adducts **E** are formed with high overall selectivities, but low yields, similar to the results observed for HOCs in the 60s and 70s [13, 14, 15, 16, 17].

In the present work, we will investigate the cycloadditions of this 10π‐component with a different class of electron‐deficient higher‐order substrates–the 3‐oxidopyridinium betaines (3OPBs) [37, 38, 39, 40, 41]. which recently have been merged with enantioselective aminocatalyzed cycloadditions [42, 43].

HOCs of 3OPBs with pentafulvenes have been reported. The first example by Katritzky et al. involves the cycloaddition of electron‐deficient 3OPB **F** with 6,6‐dimethylfulvene **G**. A higher‐order [6+4] cycloaddition affording **H** was observed, and after a[1,5]‐hydride shift, the bridged bicyclo[6.3.0]undecane adduct **I** was formed in 31% yield (Scheme 1B) [44]. Radhakrishnan et al. explored the generality of this reaction [45]. They found that 3OPB reacts with 6,6‐disubstituted pentafulvenes generating products of type **I** with control of diastereo‐, regio‐, and periselectivity in good yields. The reactivity was also investigated under other reaction conditions, using the dimeric species at elevated temperatures [46]. These conditions complicated the regio‐ and periselectivity, giving rise to the formation of up to three different products as exemplified by the reaction with the dimer of **F‐Betaine** and 6,6‐dimethylpentafulvene **G**. The reaction yields the [6+4] cycloadduct **I** in 20% yield and the two regioisomeric [4+2] cycloadducts **J** and **J’** in a combined 52% yield (Scheme 1C). The 3OPBs react as a 4π‐component in all cases, at the 2,6‐positions [47], and the observed regio‐ and periselectivity originates from the multifaceted reactivity patterns of the pentafulvenes, and not the 3OPB. Recently, Yamamoto et al. published a computational study of the reaction between 1‐(2‐pyrimidyl)‐3‐oxidopyridinium betaine and 6,6‐dimethylfulvene [48]. The calculations revealed a possible bifurcating pathway involving a [6+4]/[4+2] ambimodal TS; however, only the [6+4] cycloadduct was experimentally observed. They found that the [4+2] cycloadduct rearranges into the observed [6+4] product, a finding which suggests that the observed cycloadducts applying 3OPB in HOCs could be generated *via* more complex mechanistic pathways.

Inspired by the previous works, we will in the present work investigate the HOCs of catalytically formed amino isobenzofulvenes with 3OPBs. When 3OPB **1** was reacted with the amino isobenzofulvene intermediate **2a’**, three cycloadducts were formed; a thermally allowed [10+4] cycloadduct **3a**, and two unexpected and orbital‐symmetry forbidden regioisomeric [10+6] cycloadducts **4a** and **5a** (Scheme 1D; see Supporting Information). Previous studies with 3OPB showed only 4π‐reactivity (*i.e*., at the 2,6‐positions). In contrast, the regio‐ and periselectivity observed in the present work originates from the versatile reactivity modes of the 3OPB and not the fulvene partner as observed by Radhakrishnan et al., even though related systems have been shown to act as 8π‐components in HOCs [49]. The computational studies by Yamamoto do not explain the formation of the products observed in this reaction [42, 43].

Controlling regio‐ and periselectivity between the cycloadducts **3**, **4**, and **5** proved challenging, and therefore highlights a limitation in controlling the product outcome in complex HOCs involving two poly‐unsaturated and ambiphilic π‐addends. We have found that it is possible to identify divergent reaction conditions that can favour the [10+4] cycloadduct **3** or provide the two regioisomeric [10+6] products **4** and **5**, depending on the nature of the aminocatalyst. In addition, the cycloadducts can be generated in good, combined yields and with high diastereo‐ and enantioselectivity. To elucidate these new unexpected HOCs, DFT calculations were undertaken to investigate the reactivity pattern observed for the two pericyclic pathways.

## 2 Results and Discussion

### 2.1 Optimisation of Reaction Conditions

In the screening process, we encountered challenges related to the stability of the products. The isolated [10+4] cycloadducts **3** were generally stable. However, the [10+6] cycloadducts appeared to be unstable, in particular products **4**, which could not be isolated in analytical pure form. Products **5** could be isolated but had to be handled with care and analysed shortly after purification to ensure minimal degradation. To overcome these hurdles, we attempted various in situ transformations of the [10+6] cycloadduct (see Supporting Information).

We initially aimed at optimising the cycloaddition toward the stable [10+4] cycloadduct **3a** by reacting the dimer of 3OPB **1** with indene carbaldehyde **2a**. Our investigation started by using the salt **1‐HCl** as the source of reactive betaine in the presence of triethylamine as a base and employing catalyst **C1** in CH_2_Cl_2_ at 5 °C. This afforded the cycloadducts in 64% combined yield in an equimolar ratio of the [10+4] and [10+6] cycloadducts and with 79% *ee* of **3a** (Table 1, entry 1). Using the dimer **1** as the source of the betaine alongside with **1‐HCl** as an additive provided the cycloadducts in a similar yield, but in a different ratio and an increase in enantioselectivity of cycloadduct **3a** (entry 2). Next, we tested various solvents and catalysts. Employing **C2** or **C3** in Cl_2_CHCH_2_Cl did not alter the periselectivity significantly (entry 3 and 4). Using **C4** improved selectivity toward **3a** which was formed with excellent enantioselectivity (98% *ee*, entry 5). An increase in temperature to room temperature (rt) afforded a minor increase in favour of **3a** (entry 6), and adding lithium tetrafluoroborate as a second additive enhanced this selectivity further (entry 7). Changing the solvent to *o*‐dichlorobenzene (*o*DCB) and refining the concentration provided **3a** in approximately 2.5:1 ratio of the [10+4] to [10+6] cycloadducts with an acceptable combined yield of 61% and 95% *ee* of **3a** (entry 8–10). We then returned to the challenge of achieving [10+6] periselectivity. A catalyst and solvent screening under similar conditions as in entry 10 revealed that **C3** in trichloroethylene (TCE) provided the two regioisomers **4a** and **5a** as the major adducts (entry 11), and finally, refinement of concentration yielded the [10+6] cycloadducts with high periselectivity (4:45:51 ratio of **3a**:**4a**:**5a**), and the major product **5a** was isolated with an excellent enantioselectivity of 91% *ee* (entry 12).

**TABLE 1:** Optimisation of reaction conditions toward peri‐ and enantioselectivity for [10+4] and [10+6] cycloadditions.

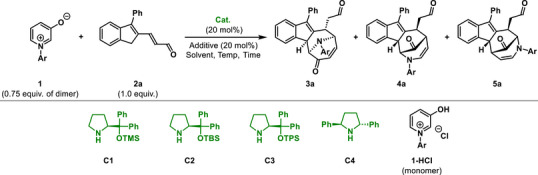									

Entry	Cat.	Additive	Solvent [M]	Temp. [°C]	Time [h]	Conv.^a^ [%]	Yield^a^ [%]	Selectivity^a^ (3a:4a:5a)	*ee* ^b^ [%]
1^c^	**C1**	Et_3_N	CH_2_Cl_2_ (0.4)	5	18	>95	64	50:25:25	79
2	**C1**	**1‐HCl**	CH_2_Cl_2_ (0.4)	5	18	>95	68	38:32:28	87
3	**C2**	**1‐HCl**	Cl_2_CHCH_2_Cl (0.4)	5	96	66	62	56:22:22	91
4	**C3**	**1‐HCl**	Cl_2_CHCH_2_Cl (0.4)	5	48	85	62	50:25:25	84
5	**C4**	**1‐HCl**	Cl_2_CHCH_2_Cl (0.4)	5	72	88	64	56:22:22	98
6	**C4**	**1‐HCl**	Cl_2_CHCH_2_Cl (0.4)	rt	24	95	70	60:20:20	96
7	**C4**	**1‐HCl**, LiBF_4_	Cl_2_CHCH_2_Cl (0.4)	rt	18	>95	78	65:27:8	91
8	**C4**	**1‐HCl**, LiBF_4_	Cl_2_CHCH_2_Cl (0.4)	rt	48	>95	60	70:22:8	90
9	**C4**	**1‐HCl**, LiBF_4_	*o*DCB (0.4)	rt	48	95	67	70:20:10	—
**10**	**C4**	**1‐HCl**, **LiBF_4_**	***o*DCB (0.2)**	**rt**	**48**	**>95**	**61**	**73:5:22**	**95**
11	**C3**	**1‐HCl**, LiBF_4_	TCE (0.4)	rt	21	91	70	15:33:51	—
**12**	**C3**	**1‐HCl**, **LiBF_4_**	**TCE (0.05)**	**rt**	**24**	**88**	**77**	**4:45:51**	**91**

The reactions were carried out on a 0.05 mmol scale. In all cases, the reaction proceeds with >20:1 d.r.

^a^ Determined by ^1^H NMR spectroscopy of the crude reaction mixture using methyl 4‐methyl‐3‐nitrobenzoate as an internal standard. The specified yield is the combined yield of all cycloadducts.

^b^ Determined by chiral stationary phase ultra‐performance convergence chromatography (UPC^2^). The specified *ee* value is for the major isomer.

^c^ Using **1‐HCl** (1.5 equiv) instead of the dimer together with Et_3_N (1.5 equiv) as the source of reactive betaine. Ar = 3,5‐dimethylpyrimidyl, *o*DCB = *o*‐dichlorobenzene, TCE = trichloroethylene.

During the optimisation process, we noticed that product ratios changed during the reaction course, even after full conversion, indicating in situ degradation of the products. We investigated this observation by tracking the conversion of indene carbaldehyde **2a** and the formation of the cycloadducts over time by ^1^H NMR spectroscopy. This was performed under each set of optimised reaction conditions; the optimised reaction conditions toward the [10+4] cycloadduct **3a** (Table 1, entry 10 = Conditions A), and the optimised reaction conditions toward the set of [10+6] cycloadducts **4a** and **5a** (Table 1, entry 12 = Conditions B).

**FIGURE 1:**
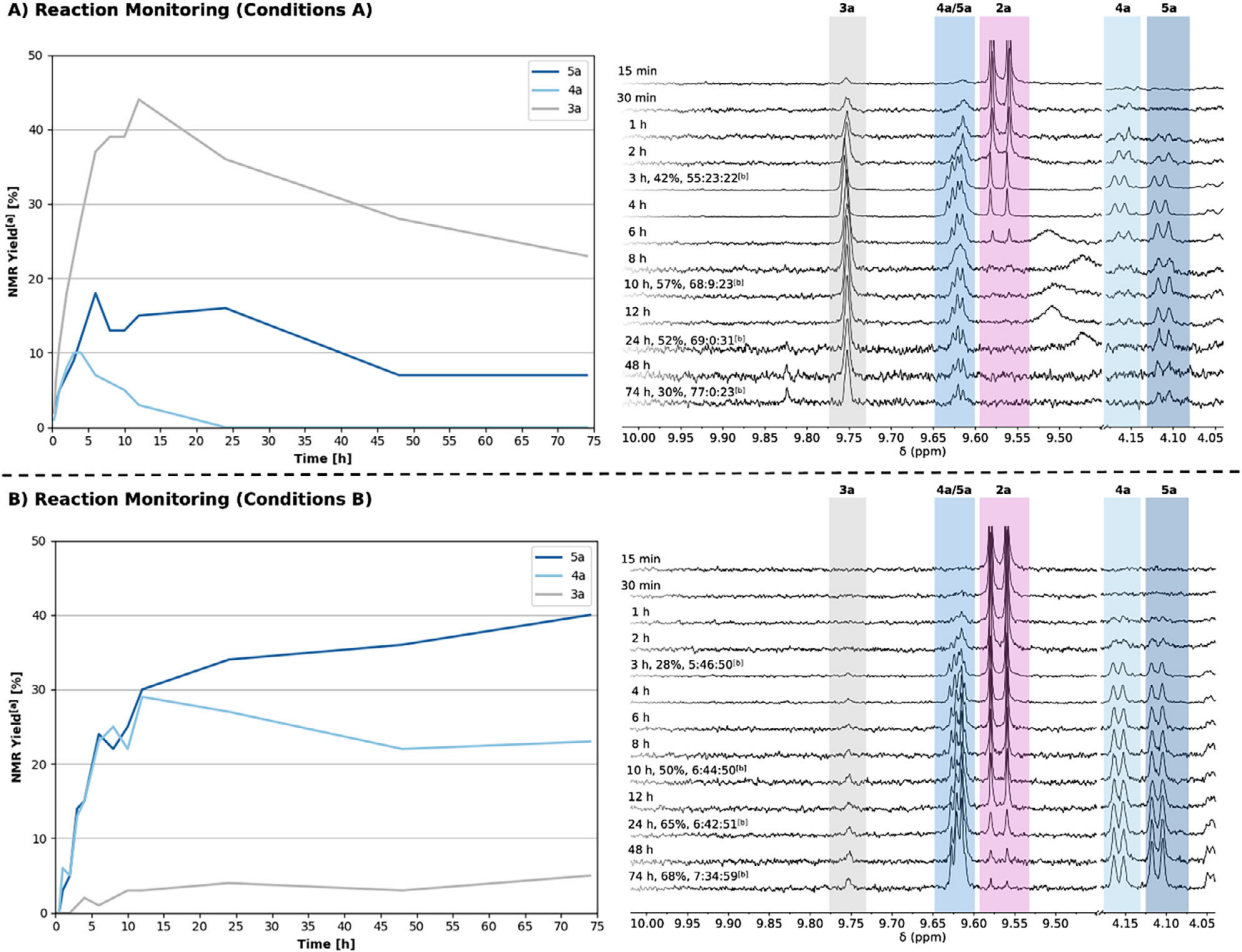
Monitoring reaction progress using ^1^H NMR spectroscopy under (A) Conditions A (entry 10) and (B) Conditions B (entry 12). [a] Determined by using methyl 4‐methyl‐3‐nitrobenzoate as an internal standard. [b] The specified yield is the combined yield of all cycloadducts. The ratio is determined by using methyl 4‐methyl‐3‐nitrobenzoate as an internal standard.

Under Conditions A, a similar rate of formation of **3a** and **4a**+**5a** was observed within the first few hours (Figure 1A). This is shown by the product ratio after 3 h (55:23:22 ratio of **3a**:**4a**:**5a**). However, after prolonged stirring it becomes apparent that all cycloadducts are unstable under these conditions, but in particular [10+6] cycloadduct **4a** is unstable as exemplified by the product ratio at 10 h (68:9:23 ratio of **3a**:**4a**:**5a**), and absence of **4a** after 24 h. These results suggest that **C4** does not only ensure periselective formation of **3a**, but also that **4a** and **5a** is degraded faster under the [10+4] reaction conditions. Thus, we propose that this instability could be attributed to degradative pathways mediated by the *endo*‐cyclic enamine functionality in products **4** and **5**. Based on these observations, the reaction was terminated after 24 h, at which the NMR yield of **3a** is high, but also **4a** and **5a** have degraded significantly, formally affording **3a** selectively.

Under Conditions B, a simpler kinetic profile was observed (Figure 1B). The reaction is generally slower, which could be attributed to the bulky nature of catalyst **C3**. Periselective formation of the [10+6] cycloadducts **4a** and **5a** (in approximately a 1:1 ratio) is observed as only minor amounts of **3a** are formed. Furthermore, **4a** and **5a** seem to be more stable under these reaction conditions but **4a** degrades slightly after 10 h.

### 2.2 Examples of [10+4] Cycloadducts 3 and [10+6] Cycloadducts 5

Despite previously reported low yields for [10+6] cycloadditions [36], we investigated the reactivity for a few variations of the indene carbaldehyde scaffold in the [10+4]‐ and [10+6] cycloadditions with 3OPB. First, we considered the formation of the [10+4] cycloadduct applying Conditions A (Table 2, top). Indene carbaldehyde **2a** afforded the cycloadducts in 61% NMR yield and with 95% *ee* of the major isomer **3a** and isolated in 39% yield. We were pleased to find that the introduction of methoxy groups at the benzo‐fusion afforded cycloadducts **3b** and **3c** with similar NMR yields, ratios, and excellent and high enantioselectivities (98% *ee* and 85% *ee*, 13% and 35% isolated yields, for **3b** and **3c**, respectively). Introduction of a bromo substituent at these positions on the benzo‐fusion led to lower NMR yields but still provided the [10+4] cycloadducts **3d** and **3e** as the major products in excellent enantioselectivities (94% *ee* and 99% *ee*, 10% and 19% isolated yields, respectively). The absolute configuration was determined by X‐ray crystallography of **3d**.

**TABLE 2:** Examples of [10+4]‐ and [10+6] cycloadditions.

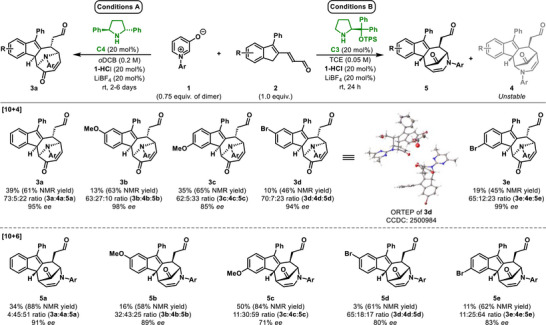

In all cases the d.r. is >20:1. Isolated yield given is for the isolated isomer. Reaction times for each entry, see Supporting Information. NMR yield is determined by using methyl 4‐methyl‐3‐nitrobenzoate as an internal standard. *ee* is determined by UPC^2^. The determined *ee* value is only for the shown product. Ar = 3,5‐dimethylpyrimidyl.

For the [10+6] cycloaddition, applying Conditions B (Table 2, bottom), the NMR yield, product ratio, and enantiomeric excess for the stable [10+6] cycloadduct **5a** are 88% combined NMR yield, 4:45:51 ratio of **3a**:**4a**:**5a**, and 91% *ee* of **5a** and isolated in 34% yield. The absolute stereochemistry was determined by ECD analysis by comparing an experimentally obtained spectrum with a computationally predicted spectrum (see Supporting Information). The cycloaddition of indene carbaldehyde **2b** afforded the cycloadducts in a lower NMR yield of 58%: the major isomer **4b** could be isolated in a nonpure state (see Supporting Information for structural assignment) and adduct **5b** was obtained with 89% *ee* and 16% isolated yield. Cycloadduct **5c** was formed as the major product in a high combined NMR yield of 84% and 50% isolated yield, but with lower enantioselectivity (71% *ee*). This could be in accordance with the reactivity‐selectivity principle–the substrate is more reactive, hence a higher yield and lower enantioselectivity. Introducing a bromo substituent at the 5‐position in the benzofusion moiety gave a combined yield of 61%, but in favour of the [10+4] cycloadduct **3d**, thus providing the [10+6] cycloadduct **5d** with 80% *ee*. One explanation for the observed preference of **3d** under Conditions B could be that the reactive amino isobenzofulvene intermediate **2d’** is more deactivated, since the bromo substituent cannot mesomerically donate electron density to the reactive 2’‐position. Hence, the thermodynamically favoured adduct **3d** is formed as the major isomer. In contrast, when the bromo substituent was introduced at the 6‐position a similar combined NMR yield was obtained, but now the major adduct was the [10+6] cycloadduct **5e**, formed with 83% *ee* and isolated in 11% yield.

### 2.3 DFT Calculations and Proposed Mechanism

We explored these reactions by DFT, using the B3LYP‐D3(BJ)/Def2‐TZVPP/SMD(*o*DCB)//ωB97X‐D/pcseg‐1/SMD(*o*DCB) functionals and basis sets with the Gaussian 09 and 16 programs [50, 51, 52, 53, 54, 55, 56, 57, 58]. All reported energies are in kcal mol^−1^ relative to the sum of the energies of **1**, **2a**, and **C4** (see Supporting Information).

**FIGURE 2:**
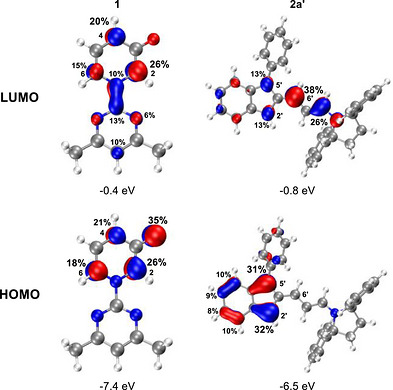
Orbital coefficients and energy levels for the HOMO and LUMO of the two reacting components **1** and **2a’**. Images were created using VMD [59].

First, the orbital coefficients and densities were computed to rationalise the reactivity pattern resulting in the formation of the [10+4] cycloadduct and the two [10+6] cycloadducts. In Figure 2, the orbital atomic densities and coefficients are shown in colour for the HOMO and LUMO orbitals of 3OPB **1** and amino isobenzofulvene intermediate **2a’**. The HOMO of **2a’** shows equal electron densities at position 2’ and 5’, thus these carbons are the most nucleophilic. Due to the steric hindrance caused by the phenyl group, reactivity will be limited to position 2’ as observed by the experiments. The LUMO of **1** has highest density at position 2, implying that this carbon is most electrophilic and thus positions 2 and 2’ are forming the first carbon‐carbon bond in **3a** and **4a**.

Although, contrary to the expected nucleophile/electrophile pattern for this system, amino isobenzofulvene intermediate **2a’** can also serve as the electrophile and 3OPB **1** as the nucleophile. The largest orbital density for the LUMO of **2a’** is found at position 6’ indicating that this is the most electrophilic position, typical for fulvenes. The HOMO of **1** shows a high electron density at the oxygen, followed by the largest contribution from position 2. Connecting position 6’ in **2a’** with position 2 in **1** correlates with the reactivity observed for the generation of cycloadduct **5a**.

Before discussing the calculated reaction pathways, it is important to note that cycloadduct **3a** results from a [10+4] cycloaddition, which is symmetry‐allowed under thermal conditions. By contrast, cycloadducts **4a** and **5a** formally correspond to [10+6] cycloadditions, which are orbital‐symmetry forbidden as concerted thermal pericyclic reactions.

The reaction pathway for the [10+4] cycloaddition was computed using the *C*_2_‐symmetrical catalyst **C4** where >20:1 d.r., 95% *ee* and a 73:5:22 ratio of **3a**:**4a**:**5a** was observed. Both literature and the crystal structure of **3d** show a preference for the *endo*‐approach [60]. consequently, only this pathway was considered in the computational analysis.

**FIGURE 3:**
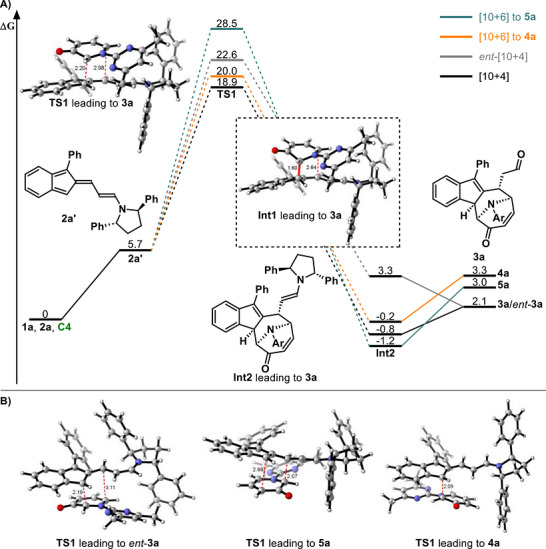
(A) Energy profile for the [10+4] HOC. The black, grey, orange, and blue pathways show the reaction profile for the [10+4] HOC, *ent*‐[10+4] HOC, [10+6] HOC to **4a**, and [10+6] HOC to **5a**, respectively. A plain line indicates the direct connection of two ground‐state structures without exploration of their associated TSs. All energies are Gibbs free energies in kcal mol^−1^ relative to the sum of **1**, **2a**, and **C4**. Only **TS1**, **Int1**, and **Int2** leading to **3a** are shown. (B) TSs geometries for **TS1** leading to *ent*‐**3a**, **4a**, and **5a**. Images were generated using CYLView [62].

The reaction for the formation of cycloadduct **3a** is initiated by condensation of the aldehyde **2a** with the organocatalyst **C4** generating amino isobenzofulvene intermediate **2a’** acting as the reactive nucleophile for the cycloaddition, which approaches 3OPB **1** in an *endo*‐fashion in **TS1**, where the first bond is formed with a TS energy of 18.9 kcal mol^−1^ (Figure 3). Next, attempts to locate a stable intermediate with one bond formed were performed, although this could not be found. The intrinsic reaction coordinate showed an asynchronous TS converging directly to cycloadduct **Int2** with both bonds formed indicating that the cycloaddition is a highly asynchronous concerted cycloaddition (see Supporting Information). In **TS1**, a bond‐forming distance of 2.20 Å is observed for the first bond, whereas the second forming bond distance is 2.98 Å. Furthermore, a third partial bond at 3.07 Å is observed between position 4 in **1** and position 5’ in **2a’**. Such a bond is typically found for ambimodal TSs [61], but since the first bond is formed with a length of 1.60 Å (**Int1**), the two reacting carbons in the second bond formation are found to be 2.64 Å apart, and the bond between position 4 in **1** and position 5’ in **2a’** is 2.99 Å, it renders this bond formation unfavourable to form as the bulky phenyl substituent is located at position 5’.

Next, the TS for the formation of the enantiomer of cycloadduct **3a** was computed. An activation energy that is 3.7 kcal mol^−1^ higher than that for **3a** is found and leads to a computed enantioselectivity of >99% *ee* which is in accordance with the 95% *ee* found experimentally. A noncovalent interaction (NCI) analysis was performed revealing more pronounced favourable electrostatic interactions between the iminium ion and the betaine for **TS1** leading to **3a** compared to **TS1** leading to *ent*‐**3a** (see Supporting Information for NCI analysis).

The TS energies for the [10+6] pathways were computed using catalyst **C4** to investigate the preference for the [10+4] cycloadduct **3a** by applying this catalyst. In both cases, the TS energy was found to be higher in energy than **TS1** which provide cycloadduct **3a**, but in the opposite order to that found experimentally. The computed energies indicate that the [10+6] cycloadduct **4a** should be the only [10+6] regioisomer observed experimentally, as an energy difference of 8.5 kcal mol^−1^ is found between the two [10+6] regioisomers. The instability of adduct **4a** complicates the investigation of this matter as adduct **5a** is the only [10+6] cycloadduct that can be isolated. From the reaction monitoring, **4a** is formed in almost the same amount as **5a**, but this still does not explain the energy difference (Figure 1). Examination of the two TSs for the regioisomers of the [10+6] cycloaddition shows that **TS1** for **4a** formation only forms the first bond, thereby making it stepwise instead of concerted. However, no TS for the second bond formation could be located and no further examination of this reaction path was considered due to the experimentally observed instability of **4a**.

When examining **TS1** for the formation of cycloadduct **5a**, a different nucleophile/electrophile pattern is observed: the first bond is found to form between position 6’ of amino isobenzofulvene intermediate **2a’** now acting as an electrophile (LUMO) and position 2 in the 3OPB **1** acting as a nucleophile (HOMO). This change in reactivity pattern makes the two reacting components ambiphilic, which further adds to the complexity of these higher HOCs (see Supporting Information). Based on the computed orbital energies, the HOMO‐LUMO gap is very similar no matter which reacting partner acts as the nucleophile or as the electrophile (Figure 2).

The Hirschfeld charges for each TS was computed showing relatively nonpolar allowed [10+4] cycloadditions whereas the two forbidden [10+6] cycloadditions were more polarised (see Supporting Information). As anticipated from **TS1** leading to **5a**, an opposite polarisation pattern was observed for this TS relative to the three others. Concerted forbidden pathways have been shown to occur for electrocyclization when high polarisation is present [63, 64]. This also seems to be the case for the [10+6] cycloaddition leading to **5a**. The [10+6] cycloaddition for formation of **4a** follows a stepwise zwitterionic pathway to circumvent the orbital symmetry control indicating that this is a case of competing relatively nonpolar allowed [10+4] and polar forbidden [10+6] cycloadditions with similar activation barriers where diastereo‐ and enantioselectivity is still possible to control by aminocatalysis [65].

After **Int2** formation, hydrolysis of the catalyst delivers the cycloadducts. The energy of the product **3a** is found to be 2.1 kcal mol^−1^ rendering it endergonic, indicating that the cycloadducts are not stable relative to the reactants. This can be explained by the decrease in NMR yield over time as shown in Figure 1A where formation of all three products is diminishing over time.

## 3 Conclusion

We have demonstrated that aminocatalytically generated isobenzofulvene intermediates can act as ambiphilic 10π‐components. With electron‐deficient 3‐oxidopyridinium betaines, they participate as electron‐rich 10π‐partners in an orbital‐symmetry‐allowed [10+4] higher‐order cycloaddition. In addition, two regioisomeric [10+6] cycloadducts are also formed, despite such processes being formally symmetry‐forbidden for concerted thermal pericyclic reactions. While one pathway involves the isobenzofulvene acting as the electron‐rich component, the other unexpectedly proceeds with reversed polarity, where the isobenzofulvene behaves as the electron‐deficient 10π‐partner and the 3‐oxidopyridinium betaine acts as the electron‐rich component. This multifaceted reactivity leads to the formation of three distinct higher‐order cycloadducts, underscoring the challenge of controlling complex HOCs between polyunsaturated and ambiphilic π‐components. We showcase that it was possible to develop a methodology to obtain either [10+4]‐ or [10+6] cycloadducts in good, combined NMR yields, and with high diastereo‐ and enantioselectivities. DFT calculations shine light on the multifaceted reactivity patterns, and the control of peri‐ and regioselectivity can be accounted for by the frontier molecular orbital of the aminocatalytically generated isobenzofulvene intermediates and 3‐oxidopyridinium betaines.

## Conflicts of Interest

The authors declare that they have no known competing financial interests or personal relationships that could have appeared to influence the work reported in this paper.

## Supporting information




**Supporting File 1**The authors have cited additional references within the Supporting Information [66‐72].
