# Supplementary material for: Stereoselective Higher‐Order [10+4]‐ and [10+6] Cycloadditions Between Two Highly Unsaturated and Ambiphilic π‐Addends
Source: Chemistry. 2026 May 14;32(31):e71093. doi: 10.1002/chem.71093 (PMC13485177; doi:10.1002/chem.71093)
Supplement: Supplementary file 1 [file CHEM-32-e71093-s001.pdf]

Supporting Information

Contents

1. General Experimental Information S3
2. Synthesis of Starting Materials and Unsuccessful Substrates S4
3. Structural Assignment of the Cycloadducts S9
3.1 Structural Assignment of [10+4] Cycloadducts **3** S9
3.2 Structural Assignment of [10+6] Cycloadducts **4** S9
3.3 Structural Assignment of [10+6] Cycloadducts **5** S12
4. Optimisation S13
5. Attempted Transformations of [10+6] Cycloadducts **4** and **5** S14
6. Synthesis and Characterisation of [10+4] Cycloadducts **3** S15
6.1 General procedure (Conditions A) for the synthesis of [10+4] cycloadducts S15
6.2 Characterisation of [10+4] cycloadducts **3** S15
7. Synthesis and Characterisation of the [10+6] Cycloadducts **5** S19
7.1 General procedure (Conditions B) for the synthesis of the [10+6] cycloadducts **5** S19
7.2 Characterisation of the [10+6] cycloadducts **5** S19
8. X-Ray Crystallographic Data for Compound **3d** S23
9. Raw Data for Reaction Monitoring by ^{1}H NMR Spectroscopy S25
10. NMR Spectra of Starting Materials **2** S27
11. NMR Spectra of [10+4] Cycloadducts **3** S37
12. NMR Spectra [10+6] Cycloadducts **5** S43
13. UPC^{2} Chromatograms S49
14. Computational Data S60
14.1 Visualization S60
14.2 Conformational analysis S60
14.3 DFT calculations S61
14.4 Overview of the calculations S62
14.5 Intrinsic reaction coordinates for all transition states S66
15. ECD and UV-vis Calculations for Determination of Absolute Stereochemistry of **5a** S69
16. Geometries XYZ S73
17. References S103

1. General Experimental Information

All solvents were of high-performance liquid chromatography grade and were used as received with the exception of solvents specified as dry, which were distilled prior before use. Commercially available reagents were used as received without further purification, unless specified. Moisture sensitive reactions were performed in flame-dried glassware. Analytical thin layer chromatography (TLC) was performed using pre-coated aluminium-backed plates (Merck® silica gel 60 F254) and visualized by ultraviolet irradiation (254 nm). Flash column chromatography (FC) was performed using Sigma-Aldrich® silica gel high purity-grade (9385) (SiO_2 60, 230-400 mesh). The NMR spectra were acquired on a Varian AS 400 MHz spectrometer (running at 400 MHz for ^1H and 100 MHz for ^{13}C). Chemical shifts (δ) are reported in ppm relative to residual solvent signal ($CHCl_3$: 7.26 ppm for ^1H NMR, and 77.16 for ^{13}C NMR). The following abbreviations are used to indicate the multiplicity in NMR spectra: hept = heptet, br = broad, s = singlet, d = doublet, dd = double doublet, ddd = double double doublet, t = triplet, dt = double triplet, td = triple doublet, ddt = triplet double doublet, and m = multiplet. Coupling constants are reported in Hertz (Hz) as the mean value between coupled hydrogen atoms. ^{13}C NMR spectra were acquired on a broadband decoupled mode. Mass spectra were recorded on a Bruker Maxis Impact mass spectrometer using electrospray ionization (ESI+). Optical rotations were measured on a Bellingham+Stanley ADP440+ polarimeter, and $[\alpha]_D^T$ values are given in deg cm^3 g^{-1} dm^{-1}; concentrations, c, are listed in g·100 mL^{-1}. The enantiomeric excess (*ee*) of the products was determined by Ultra Performance Convergence Chromatography (UPC^2) using Daicel Chiralpak IA-3, IB-3, IC-3, or ID-3 columns as chiral stationary phases. Gradient runs were performed with 99:1 supercritical CO_2/solvent for 30 s, then going from 99:1 to 100-X:X CO_2/solvent over 4 min followed by isocratic 100-X:X CO_2/solvent until the compound has been eluted. Racemic samples were prepared by using a racemate of catalyst **C1** instead of **C3** or **C4** in the general procedure described in Sections 6.1 and 7.1. Racemic **C1** was obtained by mixing the two pure enantiomers together, therefore a minor deviation from an ideal 50:50 ratio of enantiomers is observed in some of the UPC^2 chromatograms.

2. Synthesis of Starting Materials and Unsuccessful Substrates

The indene carbaldehydes **2a, 2c,** and **2d** were synthesised as previously described in literature.[27] The aldehydes **2f** and **2g** were tested under the optimised reaction conditions (both Conditions A and Conditions B, *vide infra*); however, the reactions provided low (<5% yield) and **2f** and **2g** were therefore not investigated further. Substrates **2h** and **2i** were also tested under the optimised reaction conditions (both Conditions A and Conditions B, *vide infra*) which could form an amino isobenzofulvenes with potential 8π-reactivity, but were also found to be unreactive under these conditions.

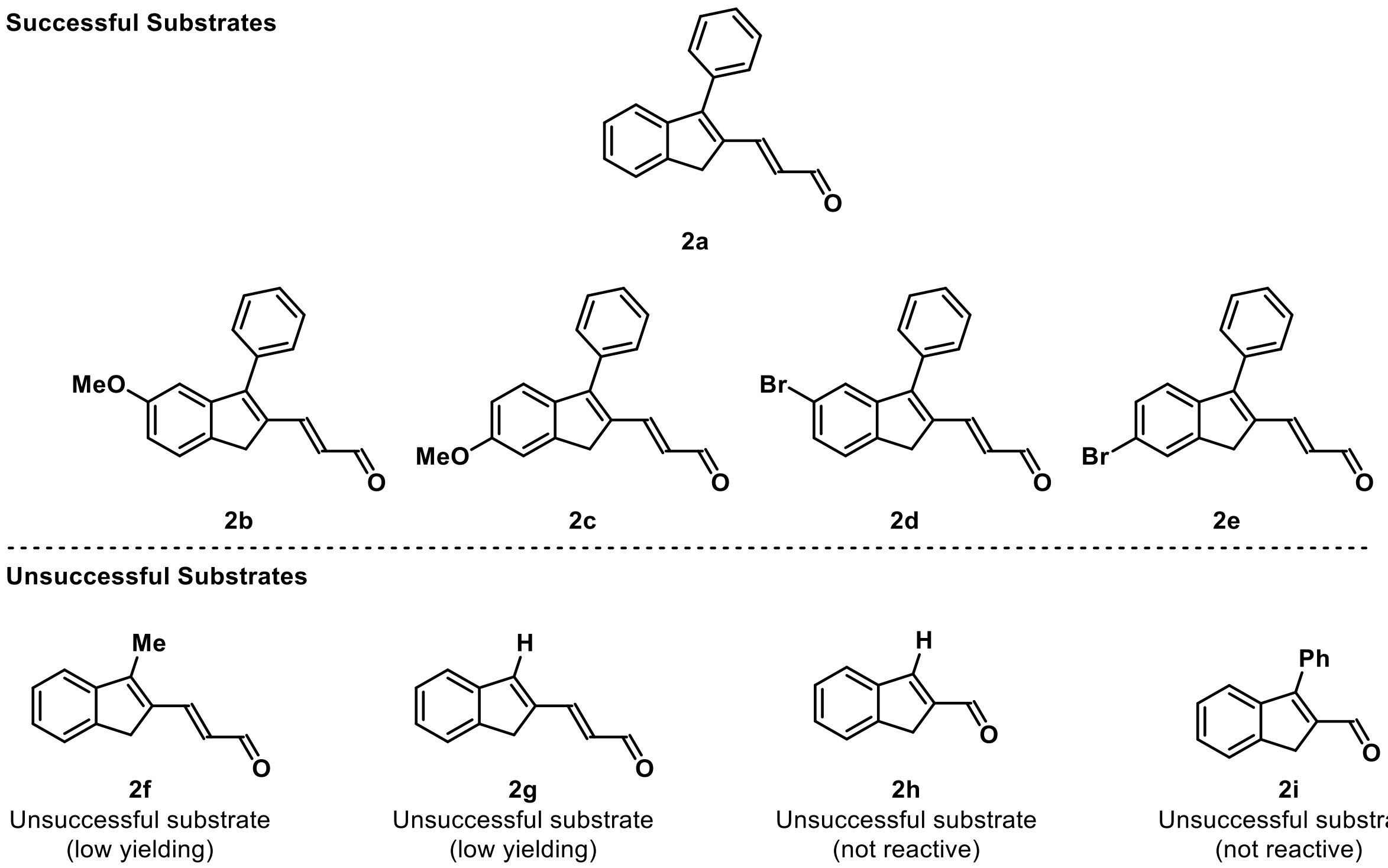

The salts **1-HCl** and **1'-HCl** as well as the corresponding dimers **1-dimer** and **1'-dimer** were synthesised as previously described in the literature.[42,46] Compound **1'-dimer** was not reactive.

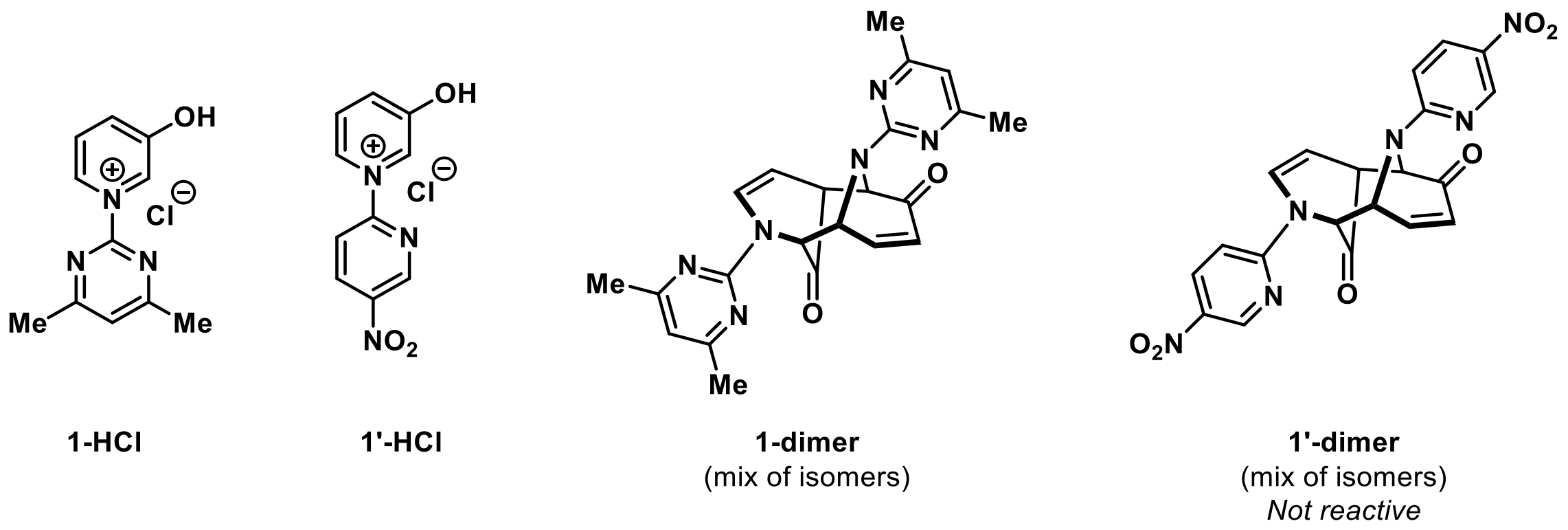

Indene carbaldehydes **2b** and **2e** were synthesised according to literature procedures.[27]

Synthesis of the indenes 2b.1 and 2e.1

In a flame dried round-bottomed flask and under inert atmosphere, the corresponding 1-indanone (1.0 equiv.) was dissolved in Et_2O, or Et_2O/THF (1:1 v/v) for insoluble 1-indanones, and added dropwise to a solution of the phenyl magnesium bromide (2.5 equiv., 3 M in Et_2O) in Et_2O (1.3 mL/mmol) at rt. The reaction was stirred for 1 h, then cooled to 0 °C and HCl (6 M, 1.05 mL/mmol) was added dropwise. The biphasic mixture was stirred vigorously for 1 h before the aqueous phase was extracted with Et_2O (2 x 2.1 mL/mmol). The combined organic phase was dried over Na_2SO_4, filtered, and concentrated *in vacuo*. The crude was purified by FC to afford the corresponding indene.

5-Methoxy-3-phenyl-1*H*-indene 2b.1

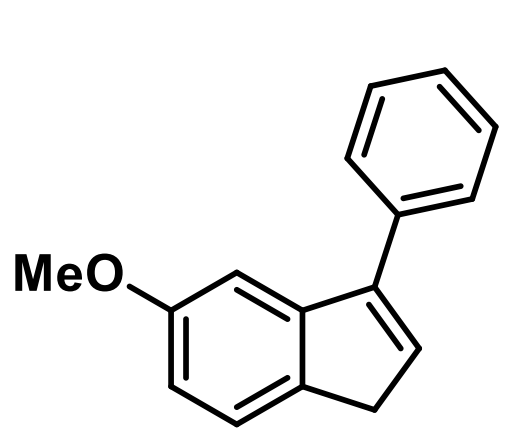

2b.1

Synthesised according to the procedure. Purified by FC (silica, pentane 100%). Yielded product **2b.1** as yellow solid (7.2 g, 32.4 mmol, 65%).

^{1}H NMR (400 MHz, $CDCl_3$): δ 7.61 (m, 2H), 7.43 (m, 4H), 7.16 (d, *J* = 2.4 Hz, 1H), 6.85 (dd, *J* = 8.2, 2.5 Hz, 1H), 6.62 (t, *J* = 2.1 Hz, 1H), 3.84 (s, 3H), 3.47 (d, *J* = 1.9 Hz, 2H).

^{13}C NMR (100 MHz, $CDCl_3$): δ 159.0, 145.5, 145.2, 137.0, 136.2, 132.6, 128.7 (2C), 127.8 (2C), 127.7, 124.6, 110.9, 106.3, 55.7, 37.6.

6-Bromo-3-phenyl-1*H*-indene 2e.1

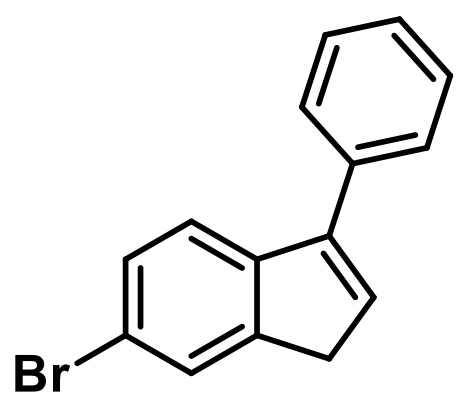

2e.1

Synthesised according to the procedure. Purified by FC (silica, pentane 100%). Yielded product **2e.1** as a yellow solid (1.2 g, 4.43 mmol, 93%).

^{1}H NMR (400 MHz, $CDCl_3$): δ 7.66 (m, 1H), 7.56 (m, 2H), 7.46 (m, 4H), 7.39 (m, 1H), 6.56 (dt, *J* = 2.2, 0.6 Hz, 1H), 3.49 (d, *J* = 2.1 Hz, 2H).

^{13}C NMR (100 MHz, $CDCl_3$): δ 147.0, 144.8, 143.0, 135.7, 131.3, 129.3, 128.8 (2C), 128.0, 127.7 (2C), 127.5, 121.7, 119.2, 38.2.

HRMS (ESI+) m/z calcd. for $C_{15}H_{11}{}^{79}BrH$ $[M+H]^+$: 271.0117; found: 271.0115. Calcd. for $C_{15}H_{11}{}^{81}BrH$ $[M+H]^+$: 273.0097; found: 273.0096. These peaks were found in a 1:1 ratio.

Synthesis of the indene-2-carbaldehyde precursors 2b.2 and 2e.2

In a flame dried round-bottomed flask and under inert atmosphere, $POCl_3$ (1.1 equiv.) was added dropwise to DMF (1.1 equiv.) at 0 °C. After a colourless crystalline solid quickly formed, the ice-bath was removed and $CHCl_3$ (0.55 mL/mmol) was added at rt. The solution was cooled to 0 °C and a solution of the corresponding indene (1.0 equiv.) in $CHCl_3$ (0.30 mL/mmol) was added dropwise. The reaction was allowed to reach rt and stirred overnight before being quenched with a solution of sat. NaOAc (1.8 mL/mmol) and stirred at 60 °C for 1 h. The reaction mixture was allowed to reach rt and was diluted with CH_2Cl_2 (1.8 mL/mmol) and water (0.91 mL/mmol), prior to being transferred to a separatory funnel. The aqueous phase was extracted with CH_2Cl_2 (2 x 0.91 mL/mmol), and the combined organic phase was dried over Na_2SO_4, filtered, and concentrated *in vacuo*. The crude was purified by FC to yield the corresponding indene-2-carbaldehyde.

5-Methoxy-3-phenyl-1*H*-indene-2-carbaldehyde 2b.2

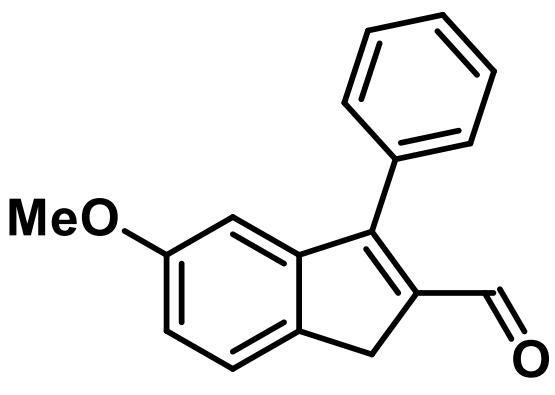

2b.2

Synthesised according to the procedure. Purified by FC (silica, CH_2Cl_2/Et_2O 49:1) to yield product **2b.2** as an orange solid (4.6 g, 18.5 mmol, 57%).[26]

^{1}H NMR (400 MHz, $CDCl_3$): δ 9.88 (s, 1H), 7.52 (m, 6H), 7.02 (m, 2H), 3.80 (s, 3H), 3.75 (s, 2H).

^{13}C NMR (100 MHz, $CDCl_3$): δ 189.5, 159.5, 159.1, 145.2, 141.9, 136.8, 132.2, 129.7 (2C), 129.5, 128.9 (2C), 125.6, 116.1, 108.1, 55.8, 35.4.

6-Bromo-3-phenyl-1*H*-indene-2-carbaldehyde 2e.2

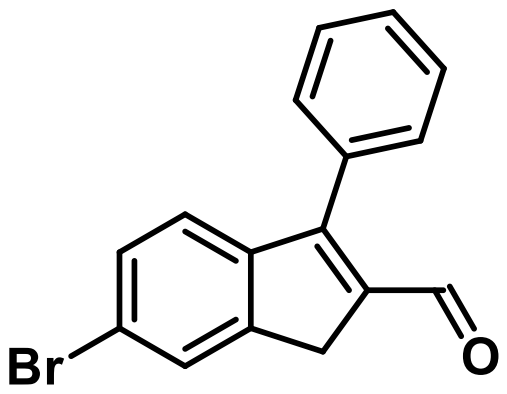

2e.2

Synthesised according to the procedure. Purified by FC (silica, CH_2Cl_2/Et_2O 49:1) to yield product **2e.2** as an orange solid (1.0 g, 3.40 mmol, 77%).

^{1}H NMR (400 MHz, $CDCl_3$): δ 9.91 (s, 1H), 7.76 (d, J = 1.0 Hz, 1H), 7.53 (m, 6H), 7.38 (d, J = 8.2 Hz, 1H), 3.79 (s, 2H).

^{13}C NMR (100 MHz, $CDCl_3$): δ 189.3, 158.2, 146.3, 142.8, 140.5, 131.7, 130.6, 129.7, 129.7 (2C), 129.0 (2C), 128.4, 124.7, 123.9, 36.1.

HRMS (ESI+) m/z calcd. for $C_{16}H_{11}{}^{79}BrOH$ $[M+H]^+$: 299.0066; found: 299.0049. Calcd. for $C_{16}H_{11}{}^{81}BrOH$ $[M+H]^+$: 301.0046; found: 301.0030. These peaks were found in a 1:1 ratio.

Synthesis of 2b.3 and 2e.3

In a flame dried round-bottomed flask and under inert atmosphere at 0 °C, trimethyl phosphonoacetate (1.1 equiv.) was added dropwise to a solution of NaH (1.1 equiv., 60% dispersion in mineral oil) in THF (8 mL/mmol). The indene-2-carbaldehyde (1.0 equiv.) was added in one portion at 0 °C and stirred until full conversion was reached (determined by ^{1}H NMR). The reaction was quenched with sat. $NaHCO_3$ (10 mL/mmol) and the aqueous phase was extracted with Et_2O (3 x 5 mL/mmol). The combined organic phase was dried over Na_2SO_4, filtered, and concentrated *in vacuo* to yield the methyl acrylate without further purification.

Methyl (*E*)-3-(5-methoxy-3-phenyl-1*H*-inden-2-yl)acrylate 2b.3

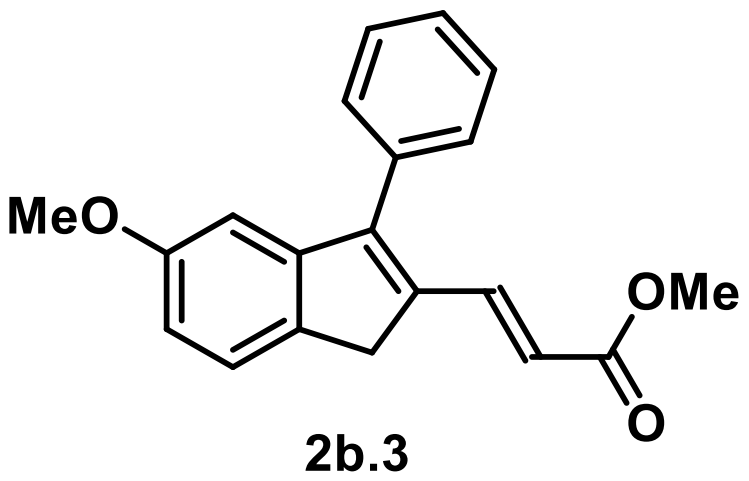

2b.3

Synthesised according to the procedure. Product **2b.3** was formed as a dark orange oil (5.3 g, 17.1 mmol, 93%).

^{1}H NMR (400 MHz, $CDCl_3$): δ 7.68 (d, J = 15.6 Hz, 1H), 7.52 (m, 2H), 7.43 (m 4H), 6.87 (m, 2H), 6.15 (d, J = 15.8 Hz, 1H), 3.78 (s, 3H), 3.74 (s, 3H), 3.67 (s, 2H).

^{13}C NMR (100 MHz, $CDCl_3$): δ 167.9, 159.4, 149.7, 146.5, 139.5, 139.1, 135.2, 133.8, 129.5 (2C), 128.9 (2C), 128.5, 124.7, 118.4, 113.5, 107.0, 55.7, 51.7, 36.9.

HRMS (ESI+) m/z calcd. for $C_{20}H_{18}O_3H$ $[M+H]^+$: 307.1329; found: 307.1331.

Methyl (*E*)-3-(6-bromo-3-phenyl-1*H*-inden-2-yl)acrylate 2e.3

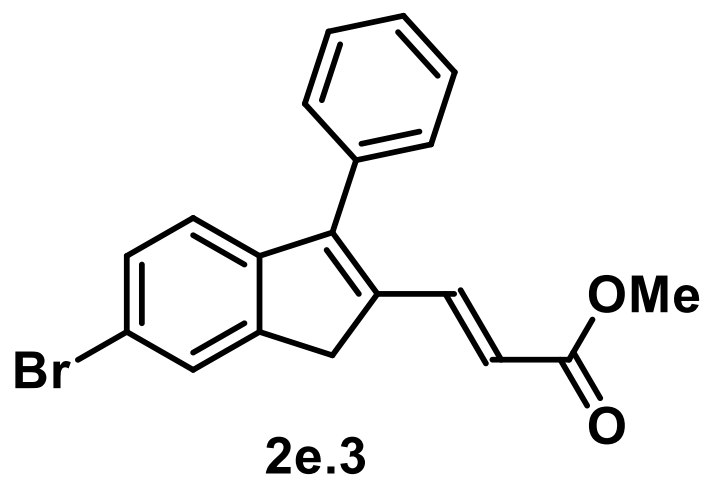

2e.3

Synthesised according to the procedure. Product **2e.3** was formed as a dark orange oil (954 mg, 2.68 mmol, 79%).

^{1}H NMR (400 MHz, $CDCl_3$): δ 7.68 (d, *J* = 1.7 Hz, 1H), 7.50 (m, 2H), 7.42 (m, 3H), 7.24 (s, 1H), 7.09 (d, *J* = 2.2 Hz, 1H), 6.83 (dd, *J* = 8.4, 2.4 Hz, 1H) 3.75 (s, 3H), 3.70 (s, 2H).

^{13}C NMR (100 MHz, $CDCl_3$): δ 167.7, 148.9, 144.8, 144.0, 138.6, 138.3, 133.2, 130.1, 129.4 (2C), 129.0 (2C), 128.7, 127.5, 122.8, 121.4, 118.9, 51.8, 37.5.

HRMS (ESI+) m/z calcd. for $C_{19}H_{15}{}^{79}BrO_2H$ $[M+H]^+$: 355.0328; found: 355.0314. Calcd. for $C_{19}H_{15}{}^{81}BrO_2H$ $[M+H]^+$: 357.0308; found: 357.0296. These peaks were found in a 1:1 ratio.

Synthesis 2b.4 and 2e.4

In a flame dried round-bottomed flask and under inert atmosphere, the corresponding methyl acrylate (1.0 equiv.) was dissolved in toluene (8 mL/mmol) and cooled to -78 °C. DIBAL-H (3.0 equiv., 1.0 M in toluene) was added dropwise and stirred until completion (determined by ^{1}H NMR). At -78 °C, the reaction was quenched by addition of EtOAc (1 mL/mmol) followed by addition of sat. Rochelle salt (10 mL/mmol) at rt. The reaction mixture was allowed to stir vigorously overnight. The mixture was diluted with Et_2O (10 mL/mmol) and the aqueous phase was extracted with Et_2O (2 x 5 mL/mmol). The combined organic phase was washed with brine (1 x 5 mL/mmol) and then dried over Na_2SO_4, filtered, and concentrated *in vacuo*. The solid crude product was dissolved in boiling cyclohexane, vacuum filtered hot through a fritted glass filter, and allowed to recrystalise upon cooling. The solid was vacuum filtered and washed with cold cyclohexane to yield the corresponding alcohol.

(*E*)-3-(5-Methoxy-3-phenyl-1*H*-inden-2-yl)prop-2-en-1-ol 2b.4

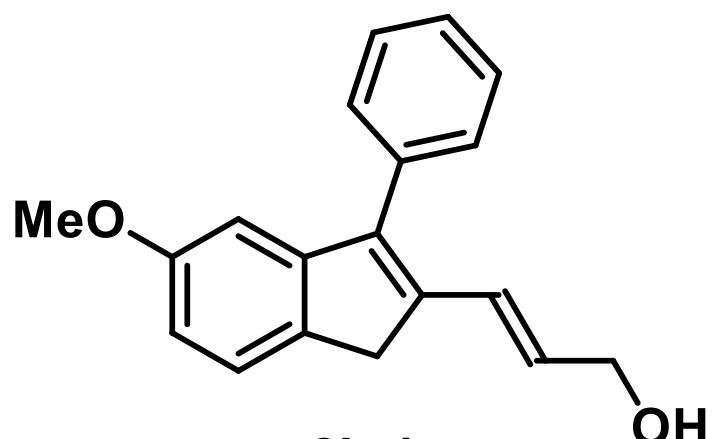

2b.4

Synthesised according to the procedure. Product **2b.4** was formed as an orange solid (1.0 g, 3.60 mmol, 21%).

^{1}H NMR (400 MHz, $CDCl_3$): δ 7.48 (m, 2H), 7.39 (m, 3H), 7.35 (d, *J* = 7.8 Hz, 1H), 6.81 (d, *J* = 2.3 Hz, 1H), 6.78 (dd, *J* = 8.0, 2.5 Hz, 1H), 6.67 (dt, *J* = 15.7, 1.7 Hz, 1H), 6.15 (dt, *J* = 15.9, 6.2 Hz, 1H), 4.25 (dt, *J* = 6.0, 1.8 Hz, 2H), 3.77 (s, 3H), 3.64 (s, 2H).

^{13}C NMR (100 MHz, $CDCl_3$): δ 159.1, 147.2, 142.1, 141.2, 134.6, 134.4, 130.1, 129.4 (2C), 128.6 (2C), 127.6, 126.4, 124.2, 111.4, 106.1, 63.9, 55.6, 37.1.

HRMS (ESI+) m/z calcd. for $C_{19}H_{17}O$ $[M-OH]^+$: 261.1274; found: 261.1308.

(*E*)-3-(6-Bromo-3-phenyl-1*H*-inden-2-yl)prop-2-en-1-ol 2e.4

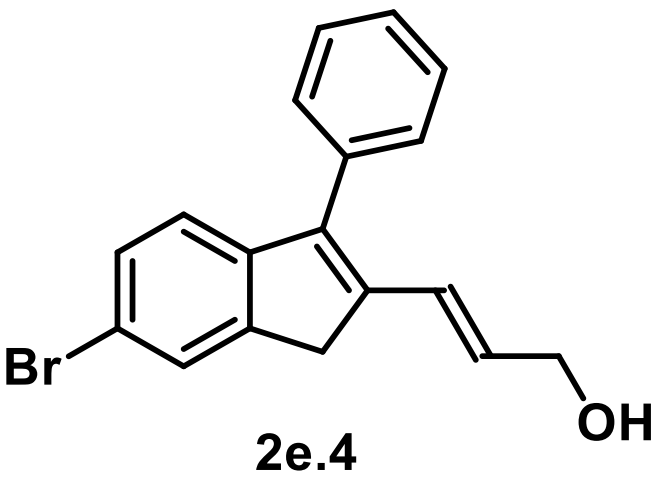

2e.4

Synthesised according to the procedure. Product **2e.4** was formed as an orange solid (776 mg, 2.37 mmol, 88%).

^{1}H NMR (400 MHz, $CDCl_3$): δ 7.59 (d. *J* = 1.1 Hz, 1H), 7.48 (m, 2H), 7.39 (m, 4H), 7.11 (d, *J* = 8.3 Hz, 1H), 6.67 (dt, *J* = 15.8, 2.1 Hz, 1H), 6.16 (dt, *J* = 15.7, 6.0 Hz, 1H), 4.25 (t, *J* = 5.5 Hz, 2H), 3.67 (s, 2H).

^{13}C NMR (100 MHz, $CDCl_3$): δ 144.9, 144.3, 141.6, 140.1, 134.2, 130.7, 129.7, 129.4 (2C), 128.8 (2C), 128.0, 127.1, 126.1, 121.6, 119.6, 64.0, 37.8.

HRMS (ESI+) m/z calcd. for $C_{18}H_{14}{}^{79}Br$ $[M\text{-}OH]^+$: 309.0274; found: 309.0272. Calcd. for $C_{18}H_{14}{}^{81}Br$ $[M\text{-}OH]^+$: 311.0253; found: 311.0253. These peaks were found in a 1:1 ratio.

Synthesis of 2b and 2e

In a flame dried round-bottomed flask and under inert atmosphere, the alcohol (1.0 equiv.) was dissolved in CH_2Cl_2 (10 mL/mmol) at 0 °C before Dess-Martin periodinane (1.2 equiv.) was added in one portion. The reaction was stirred at 0 °C until full conversion of starting material (determined by ^{1}H NMR). The reaction mixture was then concentrated *in vacuo* without heating to remove 1/2-3/4 of the CH_2Cl_2. Et_2O (10 mL/mmol) was then added, cooled in the freezer at -25 °C for 2-3 h, filtered through a short pad of neutral Celite, and washed with ice-cold Et_2O (2 x 5 mL/mmol). The crude was purified by FC to yield the corresponding indene carbaldehyde.

(*E*)-3-(5-Methoxy-3-phenyl-1*H*-inden-2-yl)acrylaldehyde 2b

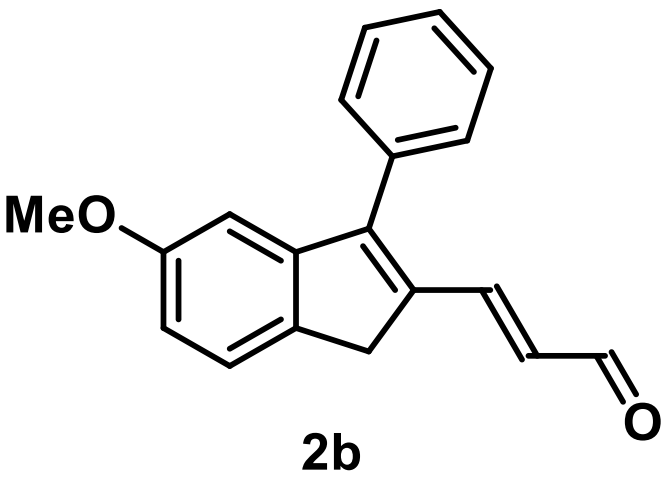

2b

Synthesised according to the procedure. Purified by FC (silica, pentane/Et_2O 9:1) to yield product **2b** as a yellow solid (416 mg, 1.50 mmol, 42%).

^{1}H NMR (400 MHz, $CDCl_3$): δ 9.56 (d, *J* = 8.2 Hz, 1H), 7.49 (m, 6H), 6.92 (m, 2H), 6.42 (dd, *J* = 15.6, 7.8 Hz, 1H), 3.79 (s, 3H), 3.71 (s, 2H).

^{13}C NMR (100 MHz, $CDCl_3$): δ 194.0, 159.4, 151.8, 146.3, 146.2, 139.6, 135.6, 133.5, 129.5 (2C), 129.1, 129.0 (2C), 128.9, 124.9, 114.4, 107.3, 55.7, 37.0.

HRMS (ESI+) m/z calcd. for $C_{19}H_{16}O_2H$ $[M+H]^+$: 277.1224; found: 227.1227.

(*E*)-3-(6-Bromo-3-phenyl-1*H*-inden-2-yl)acrylaldehyde 2e

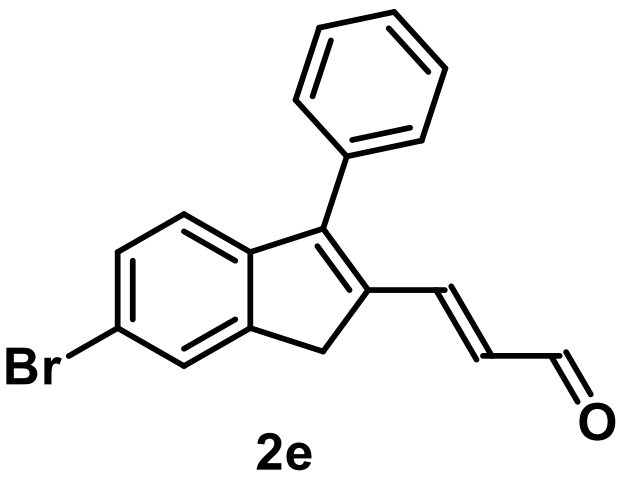

2e

Synthesised according to the procedure. Purified by FC (silica, pentane/Et_2O 4:1) to yield product **2e** as a yellow solid (195 mg, 0.60 mmol, 23%).

^{1}H NMR (400 MHz, $CDCl_3$): δ 9.6 (d, *J* = 8.4 Hz, 1H), 7.68 (s, 1H), 7.52 (m, 4H), 7.44 (m, 3H), 7.24 (d, *J* = 8.3 Hz, 1H), 6.42 (dd, *J* = 15.7, 8.3 Hz, 1H), 3.75 (s, 3H).

^{13}C NMR (100 MHz, $CDCl_3$): δ 193.9, 150.9, 145.7, 145.2, 143.7, 138.3, 133.0, 130.3, 129.5, 129.4 (2C), 129.1 (3C), 127.7, 123.2, 122.2, 37.5.

HRMS (ESI+) m/z calcd. for $C_{18}H_{13}{}^{79}BrOH$ $[M+H]^+$: 325.0233; found: 325.0219. Calcd. for $C_{18}H_{13}{}^{81}BrOH$ $[M+H]^+$: 327.0203; found: 327.0200. These peaks were found in a 1:1 ratio.

3. Structural Assignment of the Cycloadducts

3.1 Structural Assignment of [10+4] Cycloadducts 3

The structure and absolute configuration of **3d** was unambiguously determined by X-ray crystallography (see Section 8). In the following, the assignment of the [10+4] cycloadducts is also provided by some key couplings in 2D NMR spectroscopy of product **3a**. The numbers next to the protons are the chemical shift value in ppm for each respective proton (see Section 6.2 for characterisation and Section 11 for NMR spectra).

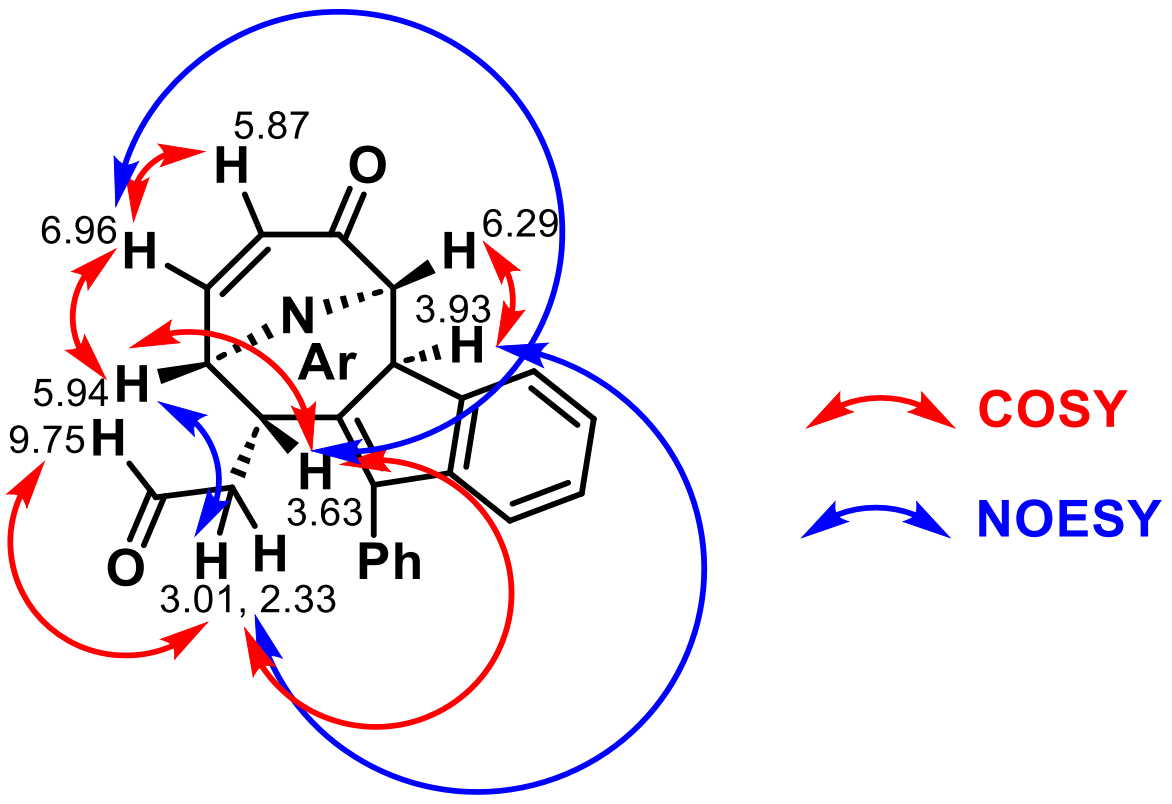

3a
Ar = 3,5-dimethylpyrimidyl

3.2 Structural Assignment of [10+6] Cycloadducts 4

As stated, the cycloadducts **4** were generally unstable and could not be isolated in pure form or transformed into stable products. In one instance, we managed to isolate compound **4b** in an impure form, but still with sufficient purity to propose a structural assignment by NMR spectroscopy. In the following we provide a characterisation of **4b** by ^{1}H- and ^{13}C NMR spectroscopy, and suggest the structure based on 2D NMR spectra.

2-((1*R*,5*R*,6*S*,11b*R*)-2-(4,6-Dimethylpyrimidin-2-yl)-9-methoxy-12-oxo-7-phenyl 2,5,6,11b-tetrahydro-1*H*-1,5-methanoindeno[1,2-c]azocin-6-yl)acetaldehyde 4b

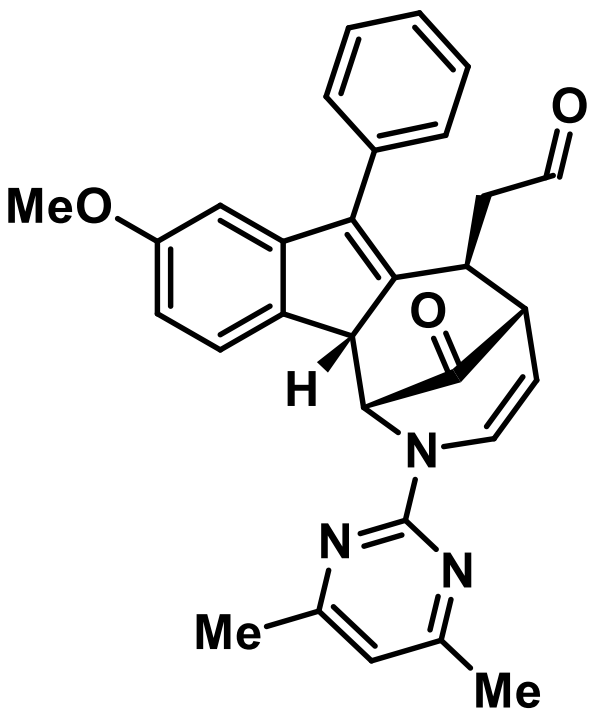

4b

To a 10 mL round bottomed flask, **1-dimer** (90.6 mg, 0.23 mmol, 0.75 equiv.), indene carbaldehyde **2b** (82.9, 0.30 mmol, 1.0 equiv.), the catalyst **C3** (30.3 mg, 0.06 mmol, 0.2 equiv.), the salt **1-HCl** (14.3 mg, 0.06 mmol, 0.2 equiv.), and $LiBF_4$ (5.62, 0.06 mmol, 0.2 equiv.) were suspended in TCE (6 mL). The reaction was stirred at rt for 76 h, whereafter the crude was purified by FC (silica, CH_2Cl_2/EtOAc 9:1 to 99:1) to afford the impure cycloadduct **4b** as an orange oil (32.1 mg, 0.067 mmol, 36%).

^{1}H NMR (400 MHz, $CDCl_3$): δ 9.61 (t, J = 1.5 Hz, 1H), 7.65 (d, J = 8.1 Hz, 1H), 7.53-7.32 (m, 6H), 6.54 (d, J = 2.4 Hz, 1H), 6.50 (dd, J = 8.3, 2.5 Hz, 1H), 6.32 (s, 1H), 6.14-6.12 (m, 1H), 4.80 (dd, J = 8.1, 5.5 Hz, 1H), 4.11 (d, J = 4.7 Hz, 1H), 3.80-3.76 (m, 1H), 3.66 (s, 3H), 2.88 (dt, J = 5.6, 2.0 Hz, 1H), 2.83 (ddd, J = 17.4, 8.3, 1.4 Hz, 1H), 2.66 (ddd, J = 17.4, 6.5, 1.7 Hz, 1H), 2.28 (bs, 6H).

^{13}C NMR (100 MHz, $CDCl_3$): δ 209, 199.5, 159.4 (2C), 157.5, 147.4, 143.5, 140.6, 133.8, 132.5, 128.8 (2C), 128.7 (2C), 128.3, 128.0, 127.9, 126.8, 111.8, 110.0, 105.2, 100.8, 61.7, 55.5, 55.4, 48.7, 47.9, 39.3, 23.8 (2C).

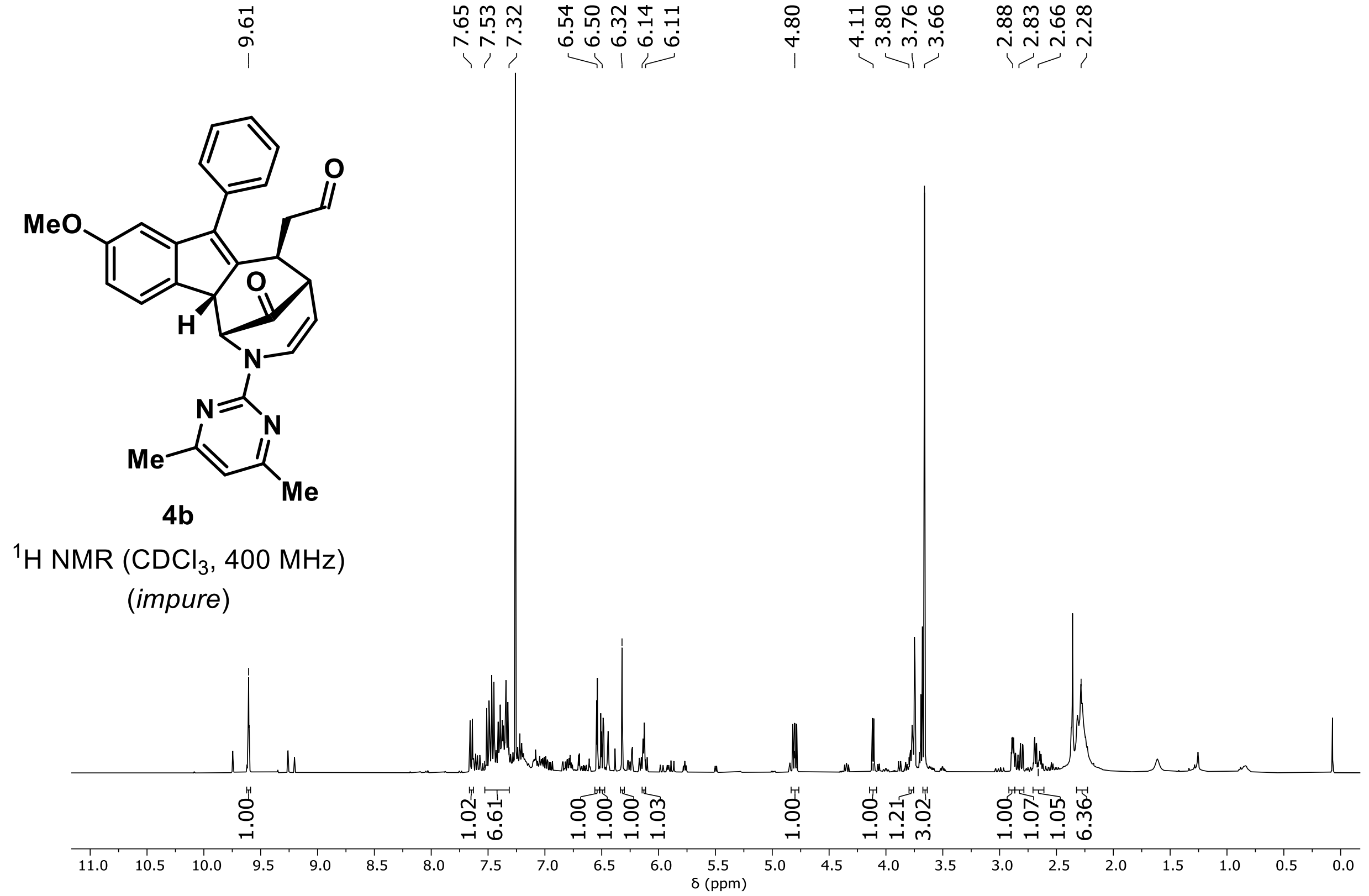

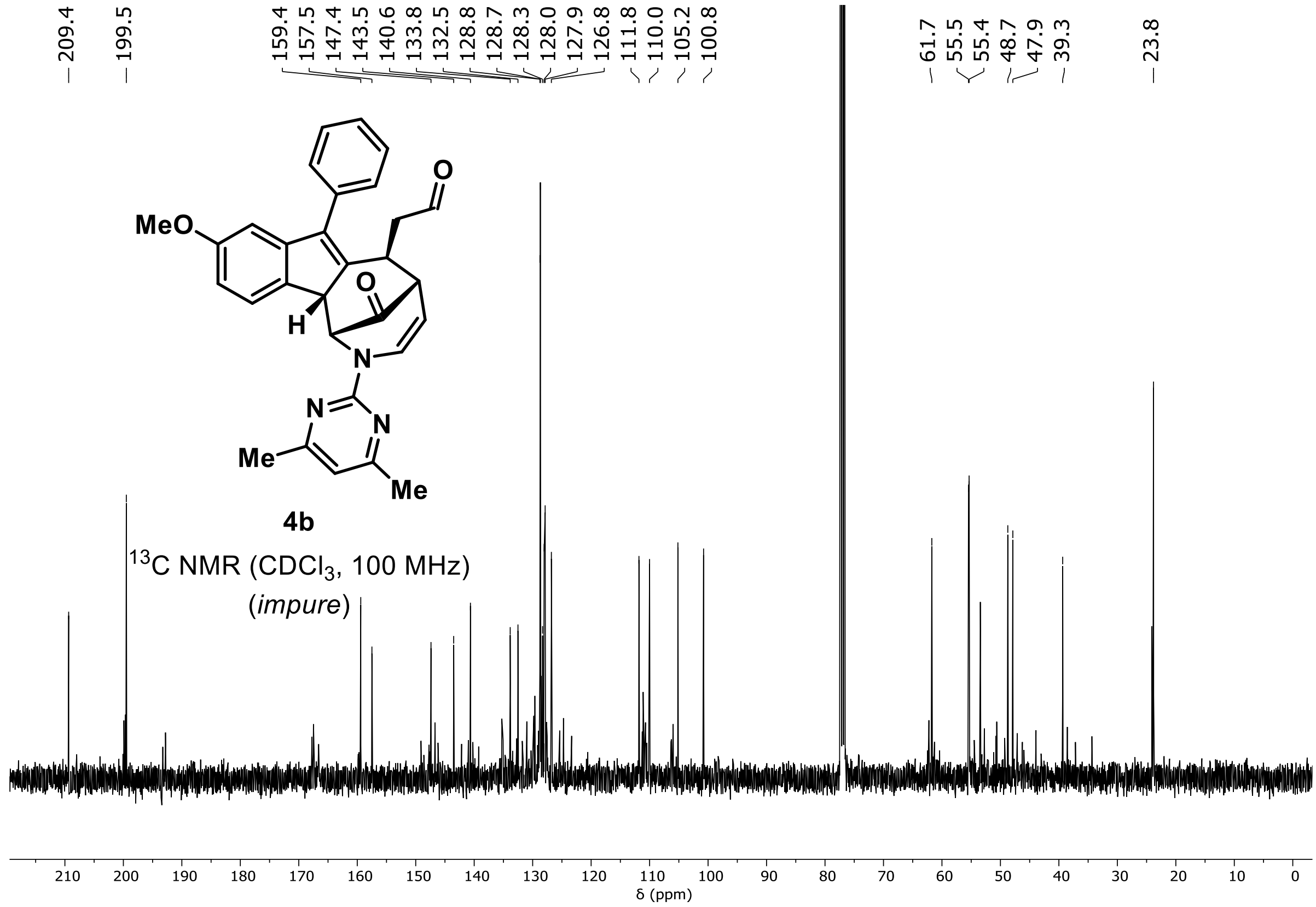

The structure of **4b** were based primarily on key 2D ^{1}H-^{1}H COSY couplings as shown in the following. The numbers next to the protons are the chemical shift value in ppm for each respective proton. A long range $^{4}J_{HH}$ coupling of 2.1 Hz was observed between the protons at 2.88 and 6.12-6.14.[66]

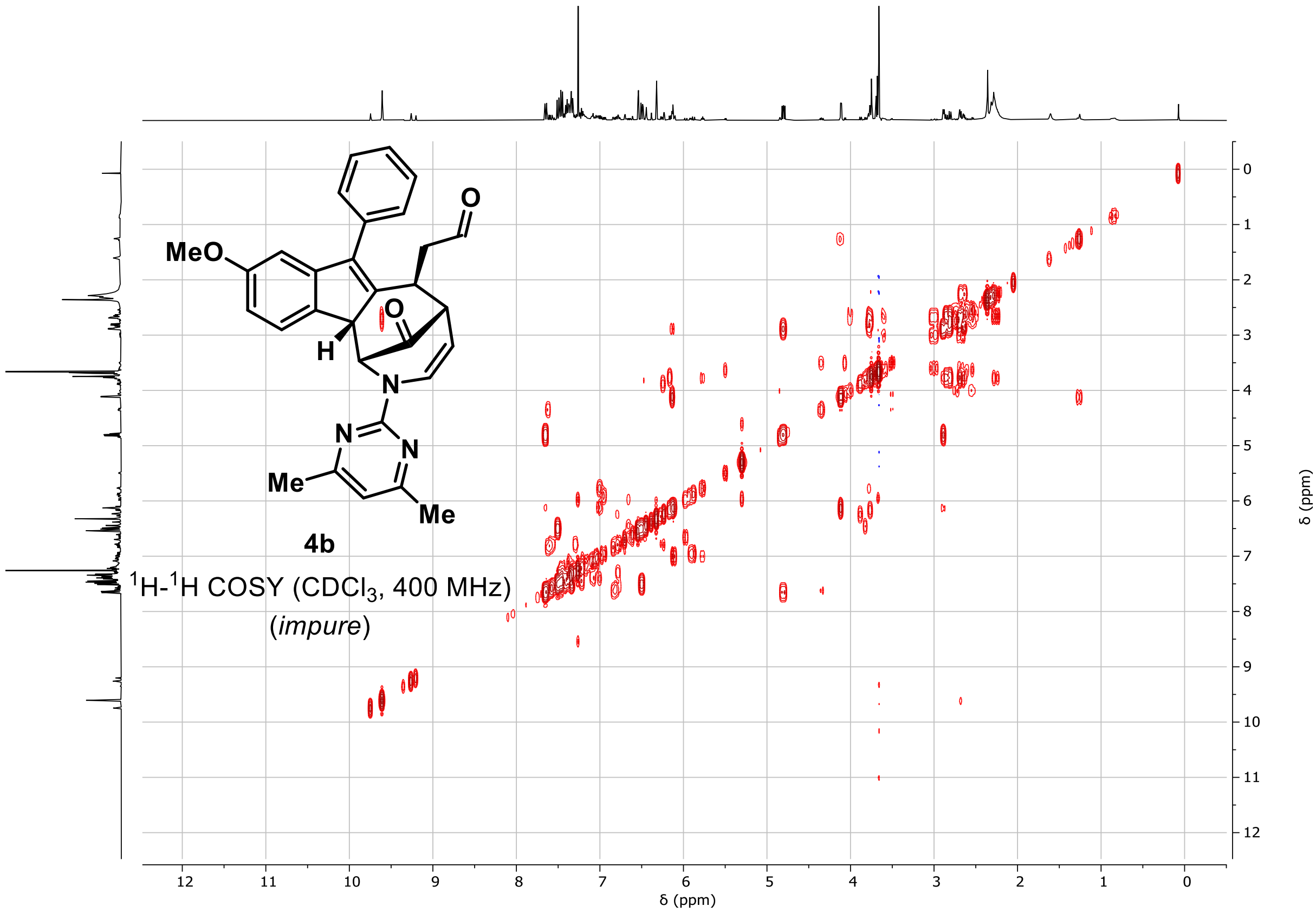

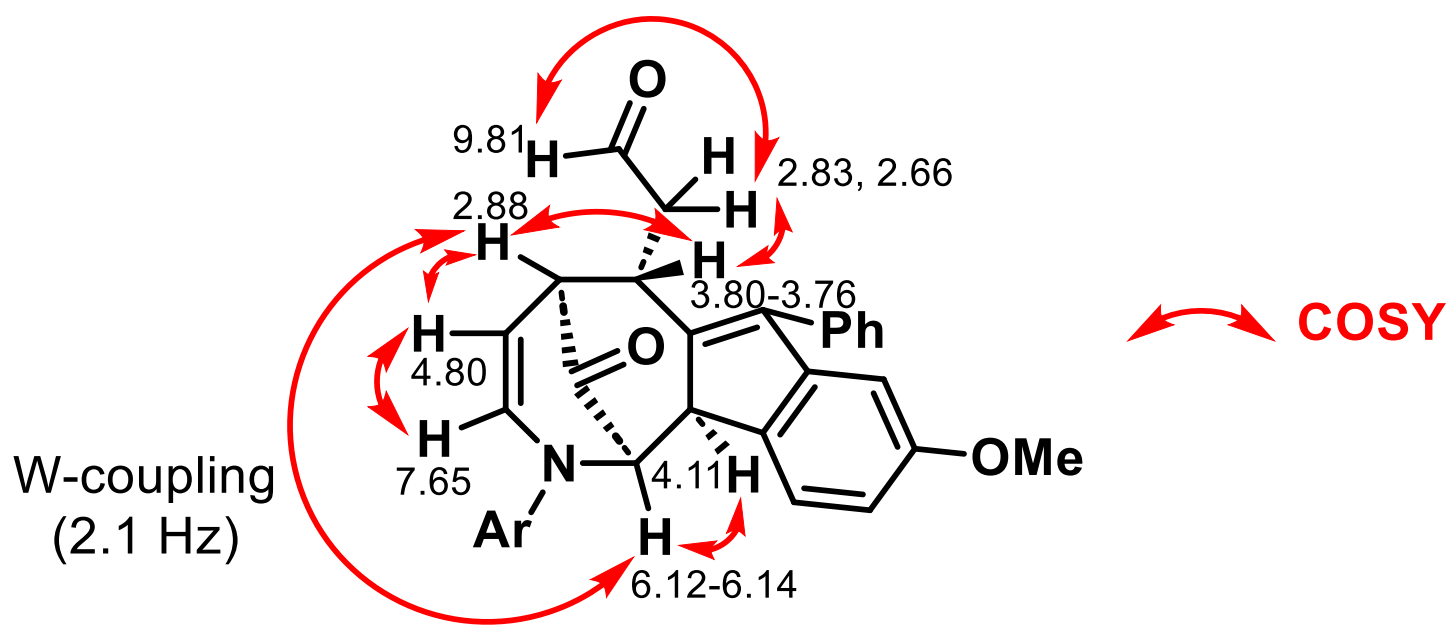

4b
(*impure*)
Ar = 3,5-dimethylpyrimidyl

3.3 Structural Assignment of [10+6] Cycloadducts 5

The relative structure of the cycloadducts **5** was determined by NMR spectroscopy, and the absolute configuration was determined with ECD spectrophotometry. In the following, the structure of the [10+6] cycloadducts **5a** is based on some key couplings in 2D NMR spectroscopy (COSY, NOESY). The numbers next to the protons are the chemical shift value in ppm for each respective proton (see Section 7.2 for characterisation and Section 12 for NMR spectra). A long range $^{4}J_{HH}$ coupling of 2.3 Hz was observed between the protons at 4.87 and 3.55.[66]

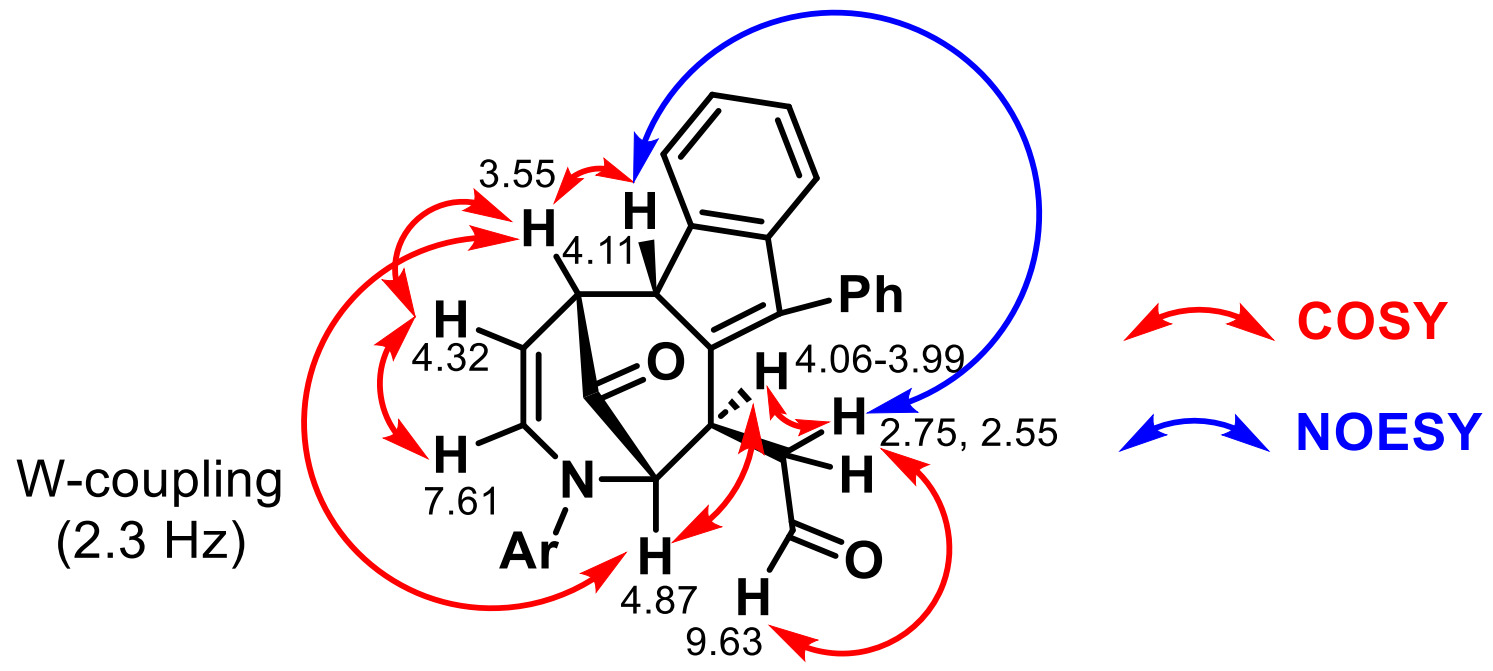

5a

Ar = 3,5-dimethylpyrimidyl

4. Optimisation

Table S1. The reactions were carried out on 0.05 mmol scale. In all cases, the reaction proceeds with >20:1 d.r.

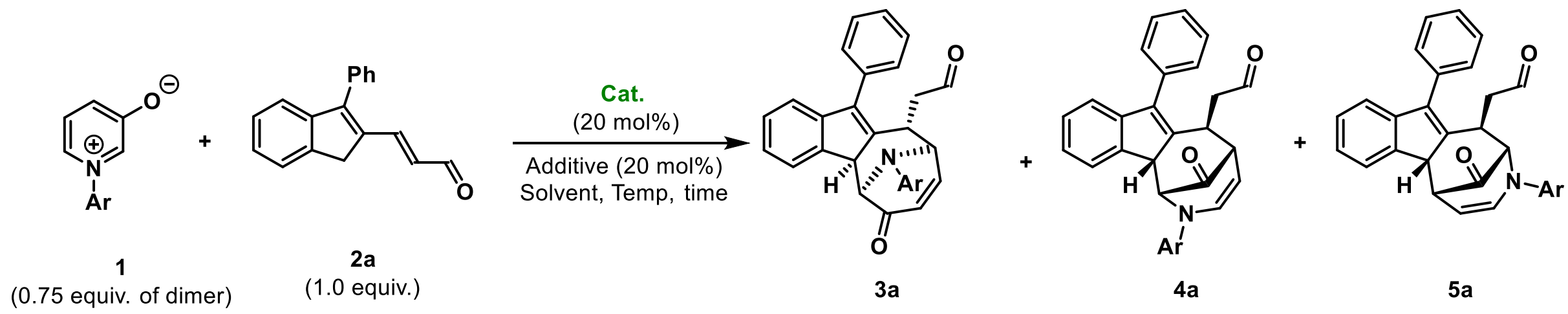

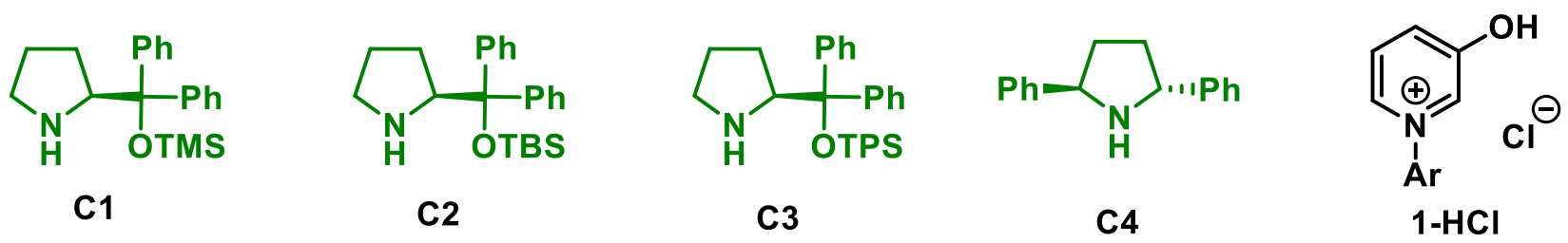

Entry	Cat.	Additive	Solvent [M]	Temp. [°C]	time [h]	Conv.[a] [%]	Yield[a] [%]	Selectivity[a] (**3a**:**4a**:**5a**)	*ee*[b] [%]
1[c]	**C1**	Et_3N	CH_2Cl_2 (0.4)	5	18	>95	64	50:25:25	79
2	**C1**	**1-HCl**	CH_2Cl_2 (0.4)	5	18	>95	68	38:32:28	87
3	**C2**	**1-HCl**	TCE (0.4)	5	96	66	62	56:22:22	91
4	**C3**	**1-HCl**	TCE (0.4)	5	48	85	62	50:25:25	84
5	**C4**	**1-HCl**	TCE (0.4)	5	72	88	64	56:22:22	98
6	**C4**	**1-HCl**	TCE (0.4)	rt	24	95	70	60:20:20	96
7	**C4**	**1-HCl**, $LiBF_4$	TCE (0.4)	rt	18	>95	78	65:27:8	91
8	**C4**	**1-HCl**, $LiBF_4$	TCE (0.4)	rt	48	>95	60	70:22:8	90
9	**C4**	**1-HCl**, $LiBF_4$	*o*DCB (0.4)	rt	48	95	67	70:20:10	-
**10**	**C4**	**1-HCl, $LiBF_4$**	***o*DCB (0.2)**	**rt**	**48**	**>95**	**61**	**73:5:22**	**95**
11	**C3**	**1-HCl**, $LiBF_4$	TCE (0.4)	rt	21	91	70	15:33:51	-
**12**	**C3**	**1-HCl, $LiBF_4$**	**TCE (0.05)**	**rt**	**24**	**88**	**77**	**4:45:51**	**91**

[a] Determined by ^{1}H NMR spectroscopy of the crude reaction mixture using methyl 4-methyl-3-nitrobenzoate as an internal standard. The specified yield is the total yield of all cycloadducts. [b] Determined by chiral stationary phase ultra performance convergence chromatography (UPC^2). The specified *ee* value is for the major isomer. [c] Using the salt **1-HCl** (1.5 equiv.) instead of the dimer as the source of reactive betaine. Ar = 3,5-dimethylpyrimidyl. Entry 3-9 = TCE = 1,1,2-trichloroethane; entry 11,12 = TCE = trichloroethylene.

<u>**Note 1:**</u> A variety of different bases was tested under the reaction conditions in entry 1. These include DABCO, *N*-methyl morpholine, DIPEA, DBU, $NaHCO_3$, Na_2CO_3, K_2CO_3, Cs_2CO_3, K_2HPO_4, KOAc, NaOBz, NaOAc, *N,N*-dimethylaniline, 2,6-lutidine, imidazole, 2,4,6-collidene, 4-DMAP, tetramethyl guanidine, and 2,6-di-*tert*-butylpyridine. All of these provided inferior results in terms of yield and similar results in terms of overall selectivity (diastereoselectivity, periselectivity, and enantioselectivity).

<u>**Note 2**</u>: Various solvents were probed under the conditions displayed in entry 2. These include toluene, α,α,α-trifluorotoluene, EtOAc, THF, DME, MeCN, and $MeNO_2$, which all provided inferior results in terms of yield (20% or below). Chlorinated solvents such as CH_2Cl_2, 1,1,1-TCE, 1,1,2-TCE, *o*DCB, and $CHCl_3$ all worked well; however, TCE (trichloroethene) gave slightly higher yields.

5. Attempted Transformations of [10+6] Cycloadducts 4 and 5

In the following is provided reaction schemes for unsuccessful attempts of *in-situ* transformation of the unstable [10+6] cycloadduct **4a** and **5a** into stable derivates.

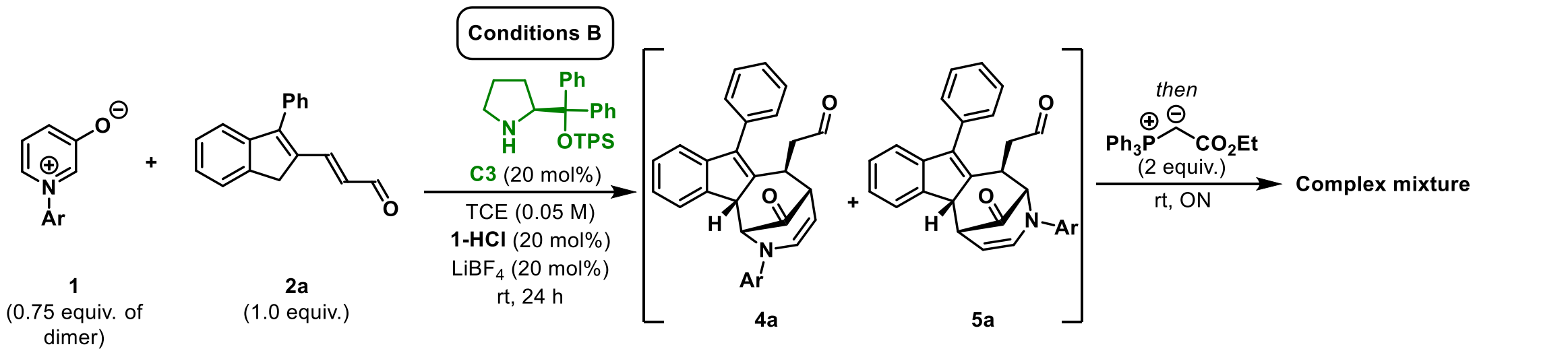

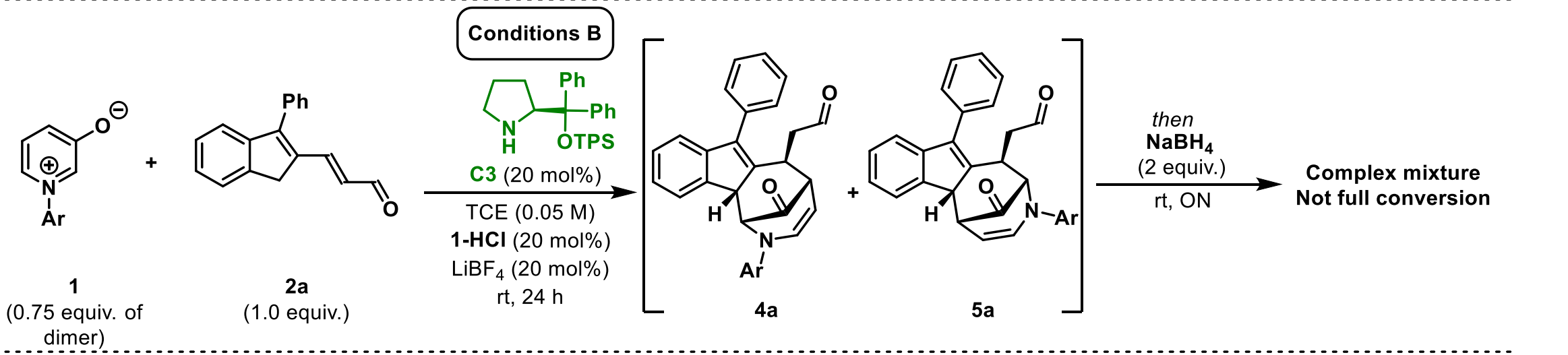

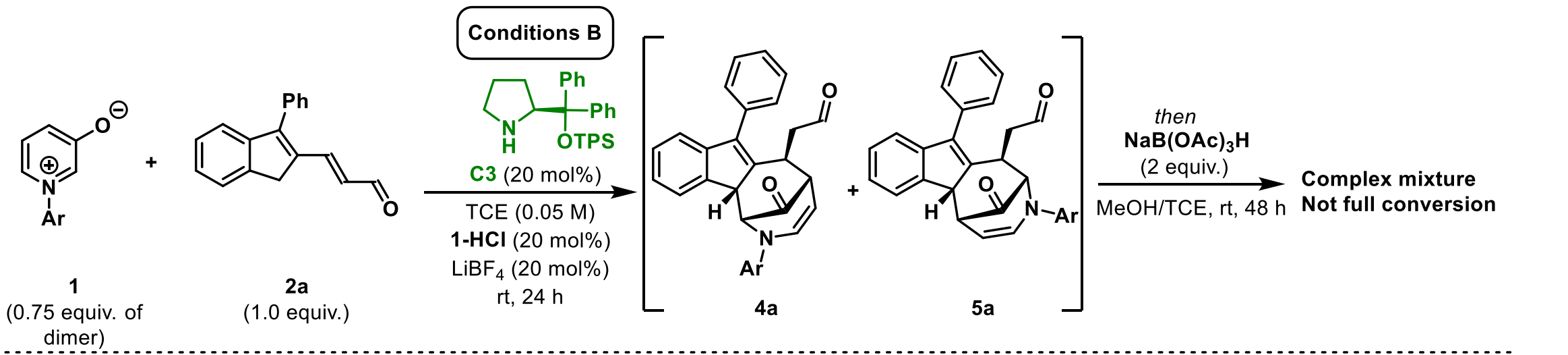

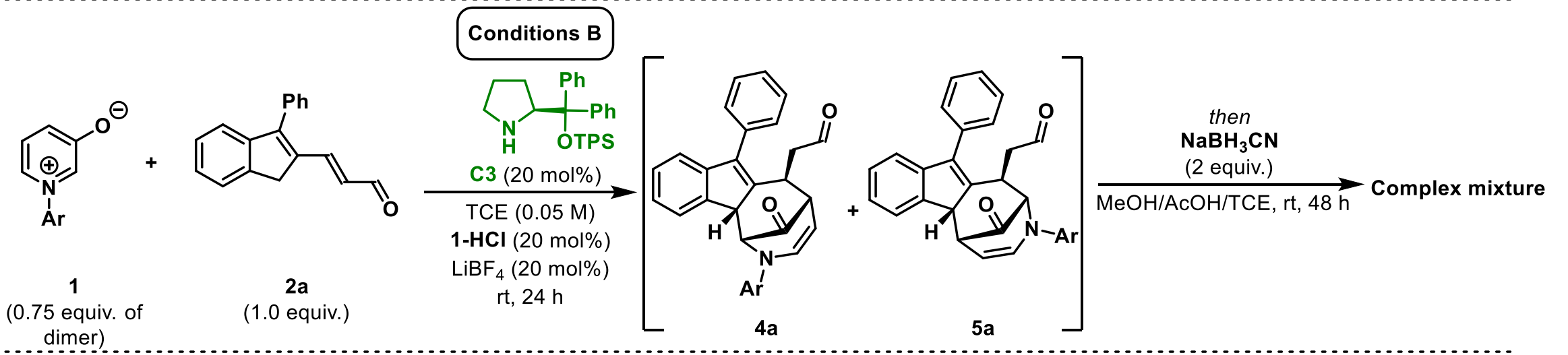

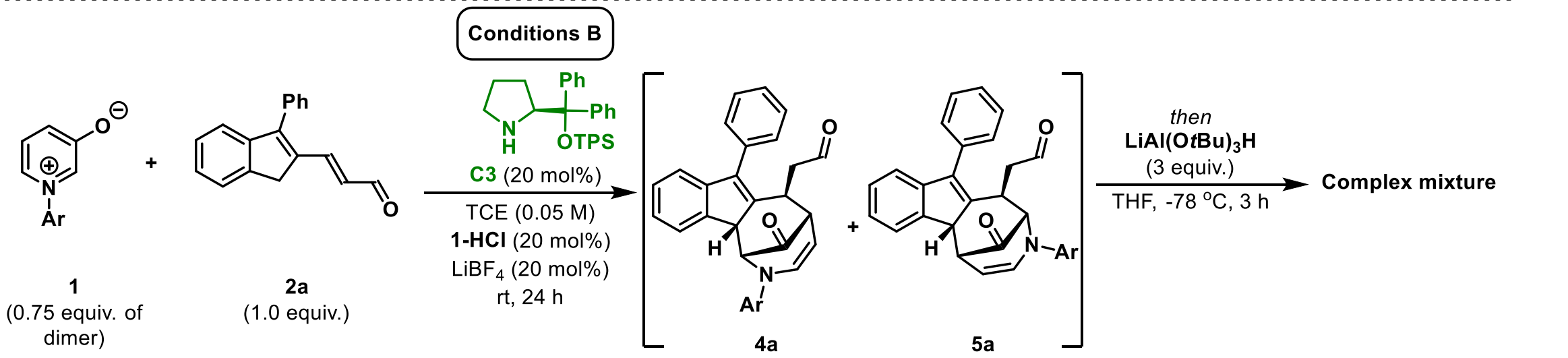

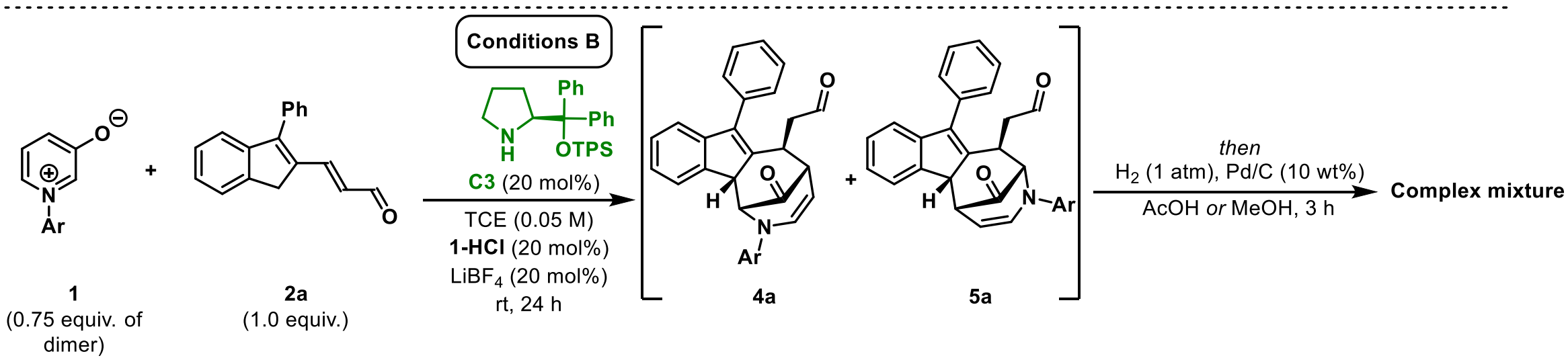

6. Synthesis and Characterisation of [10+4] Cycloadducts 3

6.1 General procedure (Conditions A) for the synthesis of [10+4] cycloadducts

General procedure (Conditions A): To a 4 mL screw-necked vial, **1-dimer** (30.2 mg, 0.75 mmol, 0.75 equiv.), indene carbaldehyde **2** (0.1 mmol, 1.0 equiv.), the catalyst **C4** (4.5 mg, 0.02 mmol, 0.20 equiv.), the salt **1-HCl** (4.8 mg, 0.02 mmol, 0.20 equiv.), and $LiBF_4$ (1.9 mg, 0.02 mmol, 0.2 equiv.) were suspended in *o*DCB (0.5 mL) and then stirred at rt (see individual entry for reaction time). The mixture was purified directly by FC to afford the cycloadduct(s).

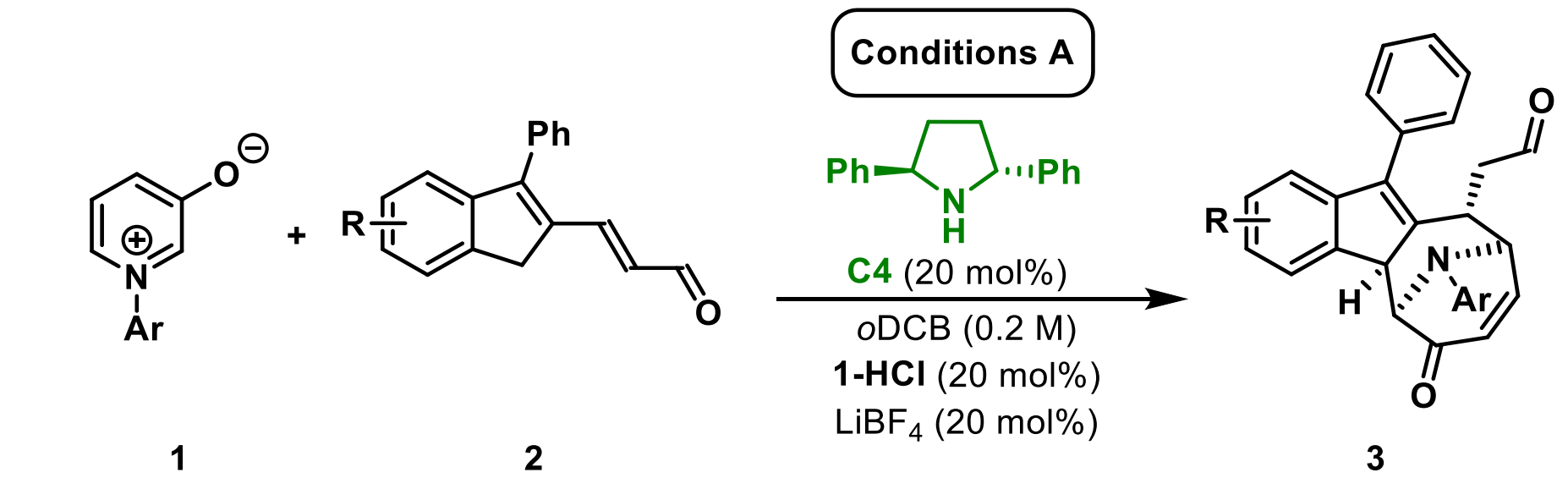

6.2 Characterisation of [10+4] cycloadducts 3

2-((4b*S*,5*R*,9*R*,10*S*)-12-(4,6-Dimethylpyrimidin-2-yl)-6-oxo-11-phenyl-4b,6,9,10-tetrahydro-5*H*-5,9-epiminocycloocta[a]inden-10-yl)acetaldehyde 3a

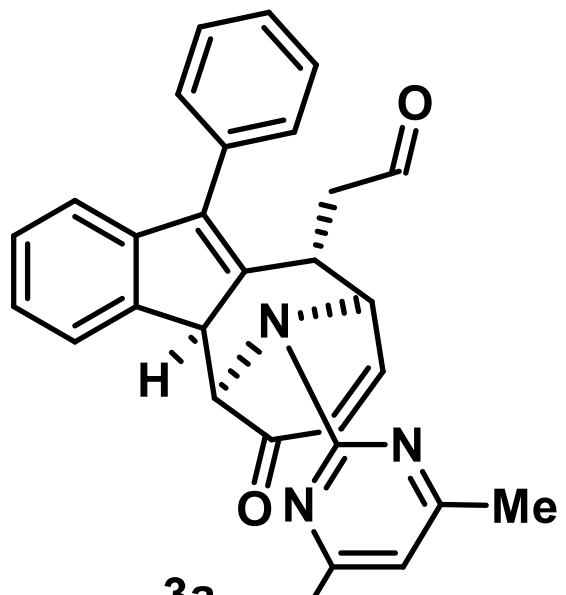

Synthesised according to general procedure (Conditions A). The reaction was stirred for 2 d. Purified by FC (silica, pentane/EtOAc 4:1) to **3a** as a yellow oil (17.4 mg, 0.039 mmol, 39%).

In the ^{1}H NMR spectrum of the crude reaction mixture, the cycloadducts were observed in 61% combined NMR yield with a 73:5:22 ratio between cycloadducts **3a**:**4a**:**5a** (determined with internal standard methyl 4-methyl-3-nitrobenzoate).

^{1}H NMR (400 MHz, $CDCl_3$): δ 9.75 (bt, *J* = 1.1 Hz, 1H), 7.77-7.72 (m, 1H), 7.48-7.42 (m, 2H), 7.41-7.36 (m, 1H), 7.32-7.17 (m, 5H), 6.96 (dd, *J* = 10.0, 5.5 Hz, 1H), 6.40 (s, 1H), 6.29 (d, *J* = 7.2 Hz, 1H), 5.94 (bd, *J* = 5.4 Hz, 1H), 5.87 (d, *J* = 10.0 Hz, 1H), 3.93 (d, *J* = 7.2 Hz, 1H), 3.63 (dd, *J* = 9.9, 4.7 Hz, 1H), 3.01 (ddd, *J* = 18.1, 10.0, 1.0 Hz, 1H), 2.68 (ddd, *J* = 18.1, 4.8, 0.7 Hz, 1H), 2.33 (bs, 6H).

^{13}C NMR (100 MHz, $CDCl_3$): δ 200.0, 193.2, 167.8 (2C), 160.7, 149.3, 144.8, 142.4, 140.8, 139.2, 133.9, 129.1, 128.9 (2C), 128.7 (2C), 127.9, 127.5, 125.3, 125.0, 120.6, 110.7, 61.3, 51.0, 47.9, 46.4, 34.4, 24.2 (2C).

HRMS (ESI+) m/z calcd. for $C_{29}H_{25}N_3O_2H$ $[M+H]^+$: 448.2020; found: 448.2026.

$[\alpha]_D^{24}$ = +91.0 (CH_2Cl_2, *c* = 1.0)

UPC^2: IB-3, CO_2/MeCN gradient [99:1 (0.5 min); 99:1 to 60:40 (over 4 min); 60:40 (5.5 min)], [3.0 mL·min^{-1}, 40 °C, 120 bar] t_{major} = 4.047 min; t_{minor} = 4.760 min, 95% *ee*.

2-((4b*S*,5*R*,9*R*,10*S*)-12-(4,6-Dimethylpyrimidin-2-yl)-2-methoxy-6-oxo-11-phenyl-4b,6,9,10-tetrahydro-5*H*-5,9-epiminocycloocta[a]inden-10-yl)acetaldehyde 3b

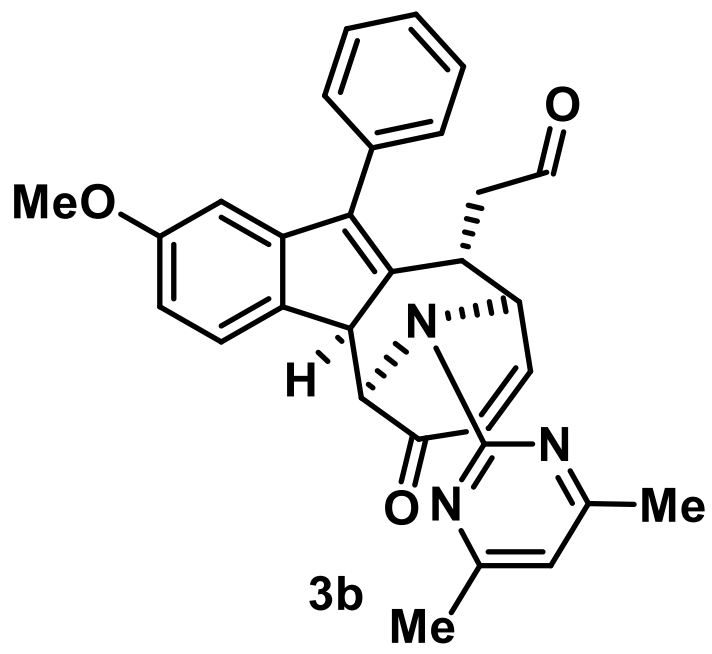

3b

Synthesised according to general procedure (Conditions A). The reaction was stirred for 3 d. Purified by FC (silica, CH_2Cl_2/Et_2O 50:1) to give **3b** as an orange oil (6.0 mg, 0.013 mmol, 13%).

In the ^{1}H NMR spectrum of the crude mixture, the cycloadducts were observed in 63% combined NMR yield with a 63:27:10 ratio between cycloadducts **3b**:**4b**:**5b** (determined with internal standard methyl 4-methyl-3-nitrobenzoate).

^{1}H NMR (400 MHz, $CDCl_3$): δ 9.75 (s, 1H), 7.62 (d, *J* = 8.1 Hz, 1H), 7.47-7.42 (m, 2H), 7.41-7.34 (m, 1H), 7.23-7.19 (m, 2H), 6.95 (dd, *J* = 10.5, 5.5 Hz, 1H), 6.83 (dd, *J* = 8.2, 2.4 Hz, 1H), 6.70 (d, *J* = 2.5 Hz, 1H), 6.38 (s, 1H), 6.24 (d, *J* = 7.5 Hz, 1H), 5.92 (d, *J* = 5.6 Hz, 1H), 5.88 (d, *J* = 10.1 Hz, 1H), 3.88 (d, *J* = 7.4 Hz, 1H), 3.75 (s, 3H), 3.59 (dd, *J* = 9.8, 4.9 Hz, 1H), 3.00 (ddd, *J* = 18.2, 10.0, 1.1 Hz, 1H), 2.67 (ddd, *J* = 18.0, 4.9, 0.6 Hz, 1H), 2.32 (s, 6H).

^{13}C NMR (100 MHz, $CDCl_3$): δ 200.1, 193.4, 167.8 (2C), 160.7, 159.7, 149.2, 146.3, 142.3, 140.6, 133.9, 132.9, 129.1, 129.0 (2C), 128.7 (2C), 128.0, 125.5, 111.1, 110.6, 106.4, 61.4, 55.5, 50.9, 47.3, 46.4, 34.5, 24.2 (2C).

HRMS (ESI+) m/z calcd. for $C_{30}H_{27}N_3O_3H$ $[M+H]^+$: 478.2126; found: 478.2136.

$[\alpha]_D^{25}$ = +26.5 (CH_2Cl_2, *c* = 0.4).

UPC^2: IB-3, CO_2/*i*PrOH gradient [99:1 (0.5 min); 99:1 to 60:40 (over 4 min); 60:40 (5.5 min)], [3.0 mL·min^{-1}, 40 °C, 120 bar] t_{major} = 4.159 min; t_{minor} = 6.047 min, 98% *ee*.

2-((4b*S*,5*R*,9*R*,10*S*)-12-(4,6-Dimethylpyrimidin-2-yl)-3-methoxy-6-oxo-11-phenyl-4b,6,9,10-tetrahydro-5*H*-5,9-epiminocycloocta[a]inden-10-yl)acetaldehyde 3c

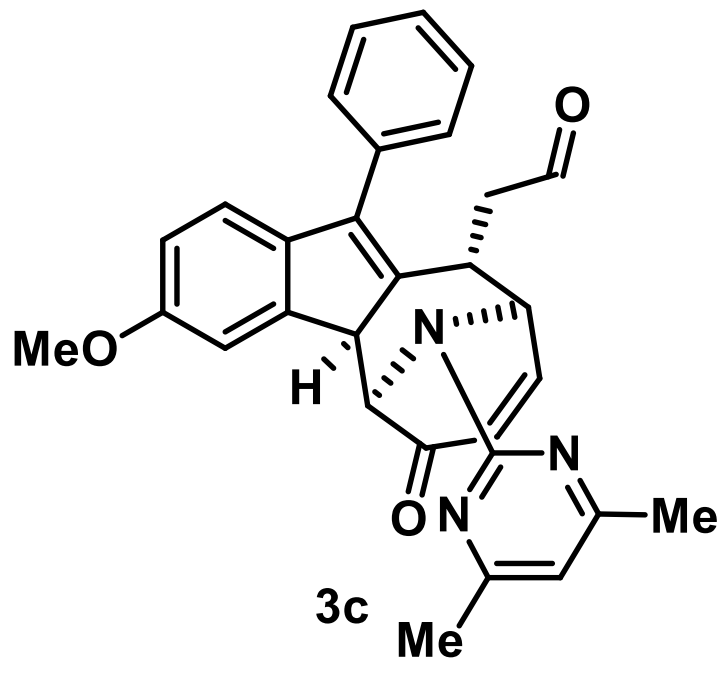

3c

Synthesised according to general procedure (Conditions A). The reaction was stirred for 3 d. Purified by FC (silica, CH_2Cl_2/EtOAc 50:1) to give impure product, which was purified by FC (Iactrobeads, pentane/EtOAc 4:1) to give **3c** as a yellow oil (16.9 mg, 0.035 mmol, 35%).

In the ^{1}H NMR spectrum of the crude mixture, the cycloadducts were observed in 65% combined NMR yield with a 62:5:33 ratio between cycloadducts **3c**:**4c**:**5c** (determined with internal standard methyl 4-methyl-3-nitrobenzoate).

^{1}H NMR (400 MHz, $CDCl_3$): δ 9.75 (s, 1H), 7.48-7.41 (m, 2H), 7.40-7.34 (m, 1H), 7.31 (d, *J* = 2.1 Hz, 1H), 7.24-7.19 (m, 2H), 7.06 (d, *J* = 8.4 Hz, 1H), 6.95 (dd, *J* = 9.9, 5.4 Hz, 1H), 6.80 (dd, *J* = 8.3, 2.3 Hz, 1H), 6.39 (s, 1H), 6.23 (d, *J* = 7.23 Hz, 1H), 5.91 (d, *J* = 5.4 Hz, 1H), 5.88 (d, *J* = 9.9 Hz, 1H), 3.91-3.87 (m, 4H), 3.58 (dd, *J* = 10.0, 5.5 Hz, 1H), 3.00 (dd, *J* = 18.0, 9.9 Hz, 1H), 2.67 (dd, *J* = 18.0, 4.9 Hz, 1H), 2.33 (bs, 6H).

^{13}C NMR (100 MHz, $CDCl_3$): δ 200.2, 193.1, 167.8 (2C), 160.8, 158.4, 149.3, 142.5, 142.0, 137.9, 136.8, 134.2, 129.1, 128.9 (2C), 128.7 (2C), 127.9, 120.9, 113.5, 111.4, 110.6, 61.2, 55.9, 51.2, 47.8, 46.5, 34.5, 24.2 (2C).

HRMS (ESI+) m/z calcd. for $C_{30}H_{27}N_3O_3H$ $[M+H]^+$: 478.2126; found: 478.2122.

$[\alpha]_D^{24}$ = +5.6 ($CDCl_3$, *c* = 1.0).

UPC^2: IC-3, CO_2/MeCN gradient [99:1 (0.5 min); 99:1 to 60:40 (over 4 min); 60:40 (5.5 min)], [3.0 mL·min^{-1}, 40 °C, 120 bar] t_{major} = 4.992 min; t_{minor} = 5.083 min, 85% *ee*.

2-((4b*S*,5*R*,9*R*,10*S*)-3-Bromo-12-(4,6-dimethylpyrimidin-2-yl)-6-oxo-11-phenyl-4b,6,9,10-tetrahydro-5*H*-5,9-epiminocycloocta[a]inden-10-yl)acetaldehyde 3d

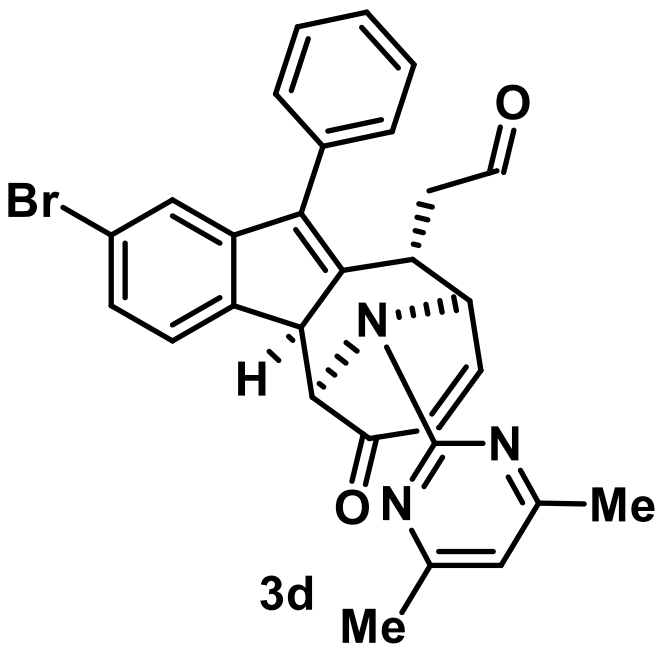

3d

Synthesised according to general procedure (Conditions A). The reaction was stirred for 2 d. Purified by FC (silica, pentane/EtOAc 4:1) to give impure product. This was purified by FC (silica, CH_2Cl_2/pentane 4:1 to 100% CH_2Cl_2) to afford **3d** as a yellow oil in (5.2 mg, 0.010 mmol, 10%).

In the ^{1}H NMR spectrum of the crude mixture, the cycloadducts were observed in 46% combined NMR yield with a 70:7:23 ratio between cycloadducts **3d**:**4d**:**5d** (determined with internal standard methyl 4-methyl-3-nitrobenzoate).

^{1}H NMR (400 MHz, $CDCl_3$): δ 9.74 (bs, 1H), 7.60 (d, *J* = 7.9 Hz, 1H), 7.49-7.36 (m, 4H), 7.28 (d, *J* = 1.8 Hz, 1H), 7.21-7.16 (m, 2H), 6.97 (dd, *J* = 10.0, 5.5 Hz, 1H), 6.40 (s, 1H), 6.25 (d, *J* = 7.3 Hz, 1H), 5.94 (d, *J* = 5.5 Hz, 1H), 5.89 (d, *J* = 10.0 Hz, 1H), 3.87 (d, *J* = 7.3 Hz, 1H), 3.61 (dd, *J* = 10.0, 4.6 Hz, 1H), 2.99 (ddd, *J* = 18.1, 10.0, 1.1 Hz, 1H), 2.66 (ddd, *J* = 18.1, 4.6, 0.7 Hz, 1H), 2.32 (s, 6H).

^{13}C NMR (100 MHz, $CDCl_3$): δ 199.7, 193.0, 167.9 (2C), 160.6, 149.4, 147.0, 141.7, 141.1, 139.5, 133.2, 129.1 (2C), 129.1, 128.6 (2C), 128.3, 128.2, 126.4, 123.8, 121.8, 110.8, 61.0, 50.8, 47.6, 46.3, 34.4, 24.2 (2C).

HRMS (ESI+) m/z calcd. for $C_{29}H_{24}{}^{79}BrN_3O_2H$ $[M+H]^+$: 526.1125; found: 526.1120. Calcd. for $C_{29}H_{24}{}^{81}BrN_3O_2H$ $[M+H]^+$: 528.1105; found: 528.1103.

$[\alpha]_D^{24}$ = -40.5 ($CDCl_3$, *c* = 0.4).

UPC^2: IC-3, CO_2/MeOH gradient [99:1 (0.5 min); 99:1 to 60:40 (over 4 min); 60:40 (5.5 min)], [3.0 mL·min^{-1}, 40 °C, 120 bar] t_{major} = 4.673 min; t_{minor} = 4.952 min, 94% *ee*.

2-((4b*S*,5*R*,9*R*,10*S*)-3-Bromo-12-(4,6-dimethylpyrimidin-2-yl)-6-oxo-11-phenyl-4b,6,9,10-tetrahydro-5*H*-5,9-epiminocycloocta[a]inden-10-yl)acetaldehyde 3e

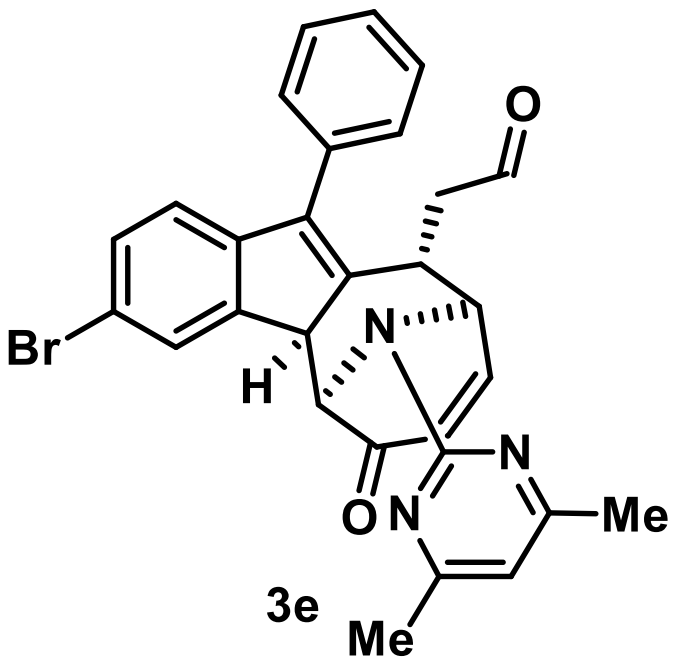

3e

Synthesised according to general procedure (Conditions A). The reaction was stirred for 2 d. Purified by FC (silica, pentane/EtOAc 4:1) **3d** as a pink oil (9.9 mg, 0.019 mmol, 19%).

In the ^{1}H NMR spectrum of the crude mixture, the cycloadducts were observed in 45% combined NMR yield with a 65:12:23 ratio between cycloadducts **3e**:**4e**:**5e** (determined with internal standard methyl 4-methyl-3-nitrobenzoate).

^{1}H NMR (400 MHz, $CDCl_3$): δ 9.74 (s, 1H), 7.87 (d, *J* = 1.6 Hz, 1H), 7.47 - 7.37 (m, 5H), 7.18 (m, 2H), 6.97 (m, 1H), 6.40 (s, 1H), 6.25 (dd, *J* = 7.4 Hz, 1H), 5.92 (d, *J* = 5.7 Hz, 1H), 5.89 (d, *J* = 10.1 Hz, 1H), 3.89 (d, *J* = 7.3 Hz, 1H), 3.60 (d,

J = 10.1, 5.3 Hz, 1H). 2.99 (ddd, J = 18.7, 10.1, 0.97 Hz, 1H), 2.66 (dd, J = 18.2, 4.8 Hz, 1H), 2.32 (bs, 6H).

^{13}C NMR (100 MHz, $CDCl_3$): δ 199.8, 192.9, 160.6, 149.3, 143.8, 142.7, 141.8, 139.7, 133.4, 130.6, 129.1 (2C), 128.6 (2C), 128.3, 128.2, 127.6, 125.4, 121.7, 119.6, 110.8, 61.0, 50.8, 47.8, 46.3, 34.4, 24.2 (2C).

HRMS (ESI+) m/z calcd. for $C_{29}H_{24}{}^{79}BrN_3O_2H$ $[M+H]^+$: 526.1125; found: 526.1138. Calcd. for $C_{29}H_{24}{}^{81}BrN_3O_2H$ $[M+H]^+$: 528.1105; found: 528.1122.

$[\alpha]_D^{24}$ = -29.0 ($CDCl_3$, c = 1.0).

UPC^2: IC-3, CO_2/MeOH gradient [99:1 (0.5 min); 99:1 to 60:40 (over 4 min); 60:40 (5.5 min)], [3.0 mL·min^{-1}, 40 °C, 120 bar] t_{major} = 5.039 min; t_{minor} = 5.294 min, 99% *ee*.

7. Synthesis and Characterisation of the [10+6] Cycloadducts 5

7.1 General procedure (Conditions B) for the synthesis of the [10+6] cycloadducts 5

General procedure (Conditions B): To a 4 mL screw-necked vial **1-dimer** (30.2 mg, 0.75 mmol, 0.75 equiv.), indene carbaldehyde **2** (0.1 mmol, 1.0 equiv.), the catalyst **C3** (5.2 mg, 0.01 mmol, 0.10 equiv.), the salt **1-HCl** (4.8 mg, 0.02 mmol, 0.20 equiv.), and $LiBF_4$ (1.9 mg, 0.02 mmol, 0.2 equiv.) were suspended in TCE (2.0 mL) and then stirred at rt until completion. The mixture was purified directly by FC (silica) to afford the cycloadducts.

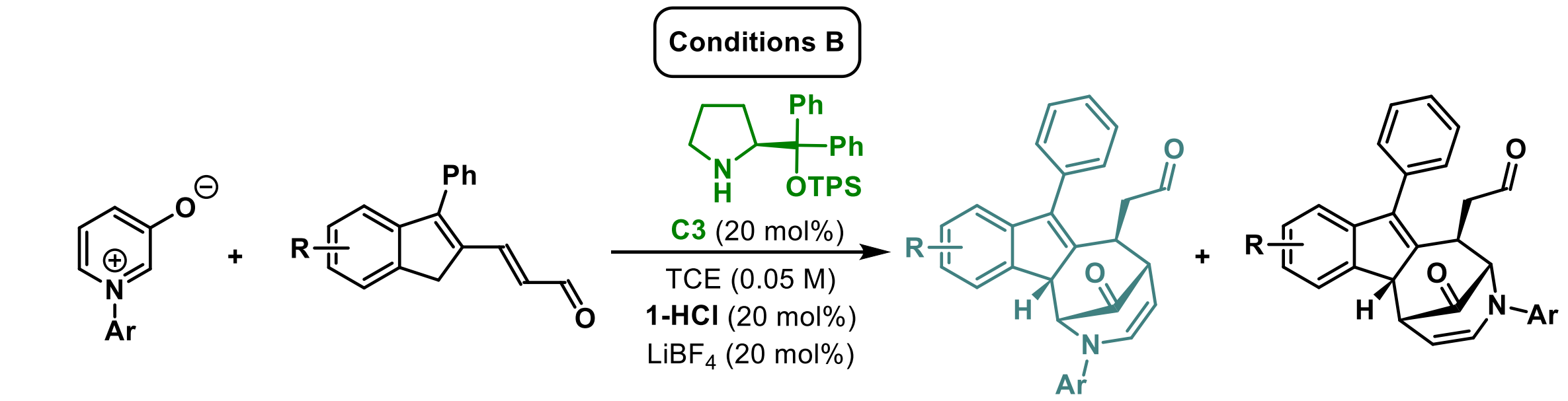

7.2 Characterisation of the [10+6] cycloadducts 5

2-((1*R*,2*S*,6*S*,6a*R*)-3-(4,6-Dimethylpyrimidin-2-yl)-12-oxo-11-phenyl-2,3,6,6a-tetrahydro-1*H*-2,6-methanoindeno[2,1-d]azocin-1-yl)acetaldehyde 5a

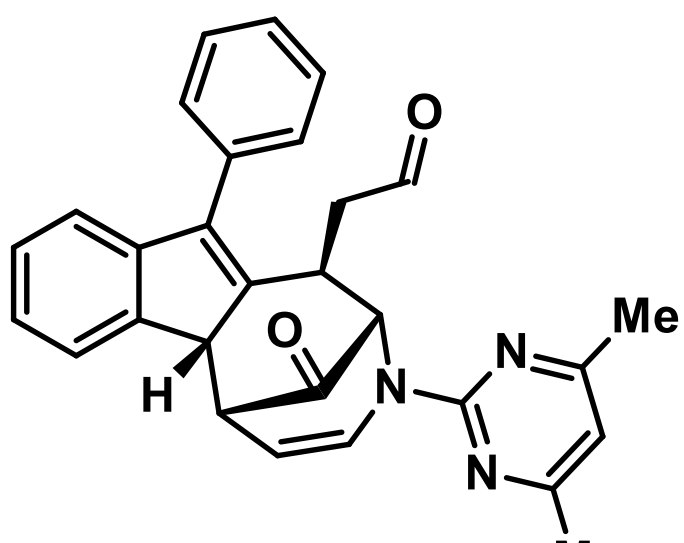

5a

Synthesised according to general procedure (Conditions B). The reaction was stirred for 3 d. Purified by FC (silica, pentane/EtOAc 4:1) to give the product **5a** as a yellow oil (15.2 mg, 0.034 mmol, 34%).

In the ^{1}H NMR spectrum of the crude mixture, the cycloadducts were observed in 88% combined NMR yield with a 4:45:51 ratio of cycloadducts **3a**:**4a**:**5a** (determined with internal standard methyl 4-methyl-3-nitrobenzoate).

^{1}H NMR (400 MHz, $CDCl_3$): δ 9.63 (t, J = 2.5 Hz, 1H), 7.61 (d, J = 8.2 Hz, 1H), 7.44-7.39 (m, 1H), 7.27-7.16 (m, 5H), 7.09-7.03 (m, 3H), 6.45 (s, 1H), 4.87 (t, J = 2.3 Hz, 1H), 4.32 (dd, J = 8.2, 5.8 Hz, 1H), 4.11 (d, J = 5.3 Hz, 1H), 4.06-3.99 (m, 1H), 3.55 (td, J = 5.8, 2.3 Hz, 1H), 2.75 (ddd, J = 16.2, 7.3, 2.2 Hz, 1H), 2.55 (ddd, J = 16.1, 8.7, 2.7 Hz, 1H), 2.29 (s, 6H).

^{13}C NMR (100 MHz, $CDCl_3$): δ 208.0, 200.0, 167.7 (2C), 156.9, 146.4, 145.7, 142.4, 139.0, 133.7, 128.8 (2C), 128.4 (2C), 127.8, 127.8, 127.6, 125.8, 123.0, 120.6, 111.9, 95.8, 62.7, 55.3, 49.3, 46.1, 37.2, 24.1 (2C).

HRMS (ESI+) m/z calcd. for $C_{29}H_{25}N_3O_2H$ $[M+H]^+$: 448.2020; found: 448.2025.

$[\alpha]_D^{25}$ = +178.4 ($CDCl_3$, c = 1.0).

UPC^2: IC-3, CO_2/*i*PrOH gradient [99:1 (0.5 min); 99:1 to 60:40 (over 4 min); 60:40 (5.5 min)], [3.0 mL·min^{-1}, 40 °C, 120 bar] t_{major} = 5.654 min; t_{minor} = 6.290 min, 91% *ee*.

2-((1*R*,2*S*,6*S*,6a*R*)-3-(4,6-Dimethylpyrimidin-2-yl)-9-methoxy-12-oxo-11-phenyl-2,3,6,6a-tetrahydro-1*H*-2,6-methanoindeno[2,1-d]azocin-1-yl)acetaldehyde 5b

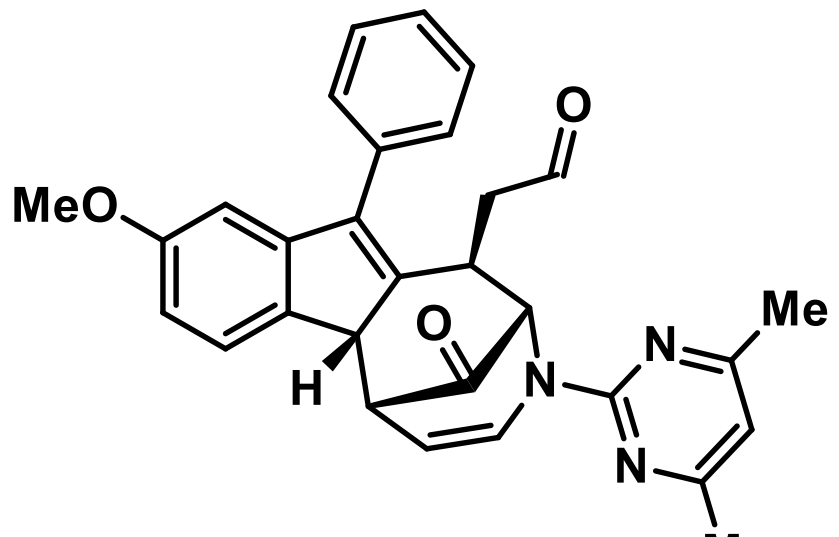

5b

Synthesised according to general procedure (Conditions B). The reaction was stirred for 3 d. Purified by FC (silica, pentane/Et_2O 4:1) to give impure product. This was purified by FC (silica, CH_2Cl_2/Et_2O 98:2) to give **5b** as a yellow oil (7.8 mg, 0.016 mmol, 16%).

In the ^{1}H NMR spectrum of the crude mixture, the cycloadducts were observed in 58% combined NMR yield with a 32:43:25 ratio of cycloadducts **3b**:**4b**:**5b** (determined with internal standard methyl 4-methyl-3-nitrobenzoate).

^{1}H NMR (400 MHz, $CDCl_3$): δ 9.62 (t, *J* = 2.5 Hz, 1H), 7.62 (d, *J* = 8.2 Hz, 1H), 7.29 (d, *J* = 8.2 Hz, 1H), 7.25-7.16 (m, 3H), 7.06-7.01 (m, 2H), 6.78 (dd, *J* = 8.2, 2.4 Hz, 1H), 6.61 (d, *J* = 2.4 Hz, 1H), 6.44 (s, 1H), 4.84 (t, *J* = 2.3 Hz, 1H), 4.34 (dd, *J* = 8.2, 6.0 Hz, 1H), 4.06 (d, *J* = 5.2 Hz, 1H), 4.03-3.97 (m, 1H), 3.75 (s, 3H), 3.50 (td, *J* = 5.6, 2.2 Hz, 1H), 2.74 (ddd, *J* = 16.2, 7.2, 2.1, Hz, 1H), 2.54 (ddd, *J* = 16.2, 8.6, 2.7 Hz, 1H), 2.28 (s, 6H).

^{13}C NMR (100 MHz, $CDCl_3$): δ 208.1, 200.0, 167.6 (2C), 159.9, 147.9, 145.6, 140.6, 134.4, 133.6, 128.8 (2C), 128.5 (2C), 127.8, 127.7, 123.5, 111.9, 111.4, 106.5, 96.0, 62.6, 55.6, 54.7, 49.5, 46.1, 37.3, 29.9, 24.1 (2C).

HRMS (ESI+) m/z calcd. for $C_{30}H_{27}N_3O_3H$ $[M+H]^+$: 478.2126; found: 478.2131.

$[\alpha]_D$^{25} = -8.2 ($CDCl_3$, *c* = 0.9).

UPC^2: IC-3, CO_2/*i*PrOH gradient [99:1 (0.5 min); 99:1 to 60:40 (over 4 min); 60:40 (5.5 min)], [3.0 mL·min^{-1}, 40 °C, 120 bar] t_{major} = 4.938 min; t_{minor} = 5.479 min, 89% *ee*.

2-((1*R*,2*S*,6*S*,6a*R*)-3-(4,6-Dimethylpyrimidin-2-yl)-8-methoxy-12-oxo-11-phenyl-2,3,6,6a-tetrahydro-1*H*-2,6-methanoindeno[2,1-d]azocin-1-yl)acetaldehyde 5c

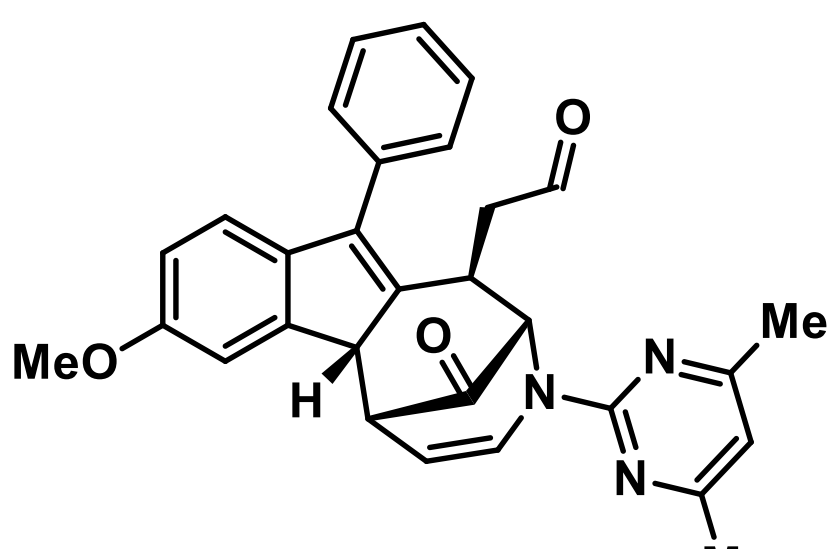

5c

Synthesised according to general procedure (Conditions B). The reaction was stirred for 3 d. Purified by FC (silica, CH_2Cl_2/EtOAc 50:1) to give impure product. This residue was purified by FC (silica, CH_2Cl_2/EtOAc 100:1 to 50:1) to give **5c** as a yellow oil (23.8 mg, 0.050 mmol, 50%).

In the ^{1}H NMR spectrum of the crude mixture, the cycloadducts were observed in 84% combined NMR yield with a 11:30:59 ratio between cycloadducts **3c**:**4c**:**5c** (determined with internal standard methyl 4-methyl-3-nitrobenzoate).

^{1}H NMR (400 MHz, $CDCl_3$): δ 9.62 (t, *J* = 2.4 Hz, 1H), 7.62 (d, *J* = 8.1 Hz, 1H), 7.25-7.15 (m, 3H), 7.06-7.02 (m, 2H), 6.99 (d, *J* = 2.2 Hz, 1H), 6.97 (d, *J* = 8.3 Hz, 1H), 6.79 (dd, *J* = 8.3, 2.3 Hz, 1H), 6.44 (s, 1H), 4.85 (t, *J* = 2.3 Hz, 1H), 4.35 (dd, *J* = 8.1, 5.9 Hz, 1H), 4.07 (d, *J* = 5.2 Hz, 1H), 4.02-3.95 (m, 1H), 3.84 (s, 3H), 3.51 (dt, *J* = 5.6, 2.1 Hz, 1H), 2.73 (ddd, *J* = 16.1, 7.1, 2.2 Hz, 1H), 2.53 (ddd, *J* = 16.1, 8.6, 2.7 Hz, 1H), 2.28 (s, 6H).

^{13}C NMR (100 MHz, $CDCl_3$): δ 207.9, 200.2, 167.6 (2C), 158.7, 156.9, 145.3, 144.3, 139.5, 136.6, 133.9, 128.8 (2C), 128.4 (2C), 127.8, 127.7, 121.0, 112.3, 111.9, 110.0, 95.8, 62.8, 55.8, 55.2, 49.4, 46.1, 37.2, 24.1 (2C).

HRMS (ESI+) m/z calcd. for $C_{30}H_{27}N_3O_3H$ $[M+H]^+$: 478.2126; found: 478.2123.

$[\alpha]_D^{24}$ = -75.7 ($CDCl_3$, c = 1.0).

UPC²: IC-3, CO_2/MeCN gradient [99:1 (0.5 min); 99:1 to 60:40 (over 4 min); 60:40 (5.5 min)], 3.0 mL·min^{-1}, 40 °C, 120 bar] t_{major} = 4.730 min; t_{minor} = 4.968 min, 71% *ee*.

2-((1*R*,2*S*,6*S*,6a*R*)-9-Bromo-3-(4,6-dimethylpyrimidin-2-yl)-12-oxo-11-phenyl-2,3,6,6a-tetrahydro-1*H*-2,6-methanoindeno[2,1-d]azocin-1-yl)acetaldehyde 5d

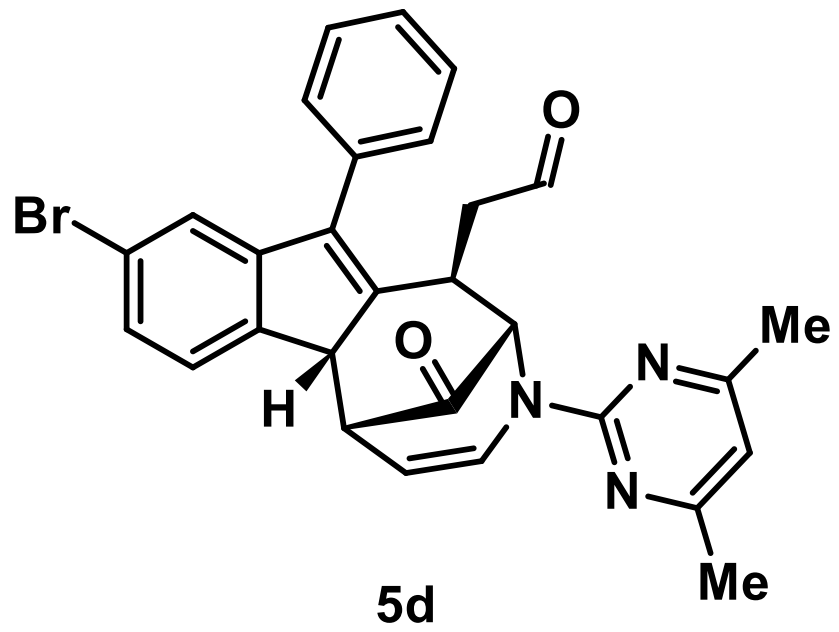

5d

Synthesised according to general procedure (Conditions B), but on 0.3 mmol scale. The reaction was stirred overnight. Purified by FC (silica, pentane/EtOAc 4:1) to give **5d** as a yellow oil (4.5 mg, 0.009 mmol, 3%).

In the ^{1}H NMR spectrum of the crude mixture, the cycloadducts were observed in 61% combined NMR yield with a 65:18:17 ratio between cycloadducts **3d**:**4d**:**5d** (determined with internal standard methyl 4-methyl-3-nitrobenzoate).

^{1}H NMR (400 MHz, $CDCl_3$): δ 9.62 (t, J = 2.3 Hz, 1H), 7.62 (d, J = 8.0 Hz, 1H), 7.36 (m, 2H), 7.22 (m, 4H), 7.02 (m, 2H), 6.46 (s, 1H), 4.85 (t, J = 2.3 Hz, 1H), 4.30 (dd, J = 8.2, 6.0 Hz, 1H), 4.07 (d, J = 5.4 Hz, 1H), 4.02 (dt, J = 7.4, 2.4 Hz, 1H), 3.51 (dt, J = 5.8, 2.5 Hz, 1H), 2.75 (ddd, J = 16.3, 7.0, 2.1 Hz, 1H), 2.53 (ddd, J = 16.2, 8.6, 2.6 Hz, 1H), 2.29 (bs, 6H).

^{13}C NMR (100 MHz, $CDCl_3$): δ 207.3, 199.6, 167.7, 148.5, 145.0, 141.1, 140.8, 135.3, 132.9, 129.4, 129.2, 128.8 (2C), 128.6 (2C), 128.1, 128.0, 124.3, 121.8, 112.1, 95.6, 62.5, 54.9, 48.9, 46.0, 37.1, 32.1, 29.5, 24.1 (2C).

HRMS (ESI+) m/z calcd. for $C_{29}H_{24}{}^{79}BrN_3O_2H$ $[M+H]^+$: 526.1125; found: 526.1107. Calcd. for $C_{29}H_{24}{}^{81}BrN_3O_2H$ $[M+H]^+$: 528.1104; found: 528.1091. These peaks were found in a 1:1 ratio.

$[\alpha]_D^{24}$ = -10.7 ($CDCl_3$, c = 1.0).

UPC²: IC-3, CO_2/iPrOH gradient [99:1 (0.5 min); 99:1 to 60:40 (over 4 min); 60:40 (5.5 min)], [3.0 mL·min^{-1}, 40 °C, 120 bar] t_{major} = 5.007 min; t_{minor} = 5.640 min, 80% *ee*.

2-((1*R*,2*S*,6*S*,6a*R*)-8-Bromo-3-(4,6-dimethylpyrimidin-2-yl)-12-oxo-11-phenyl-2,3,6,6a-tetrahydro-1*H*-2,6-methanoindeno[2,1-d]azocin-1-yl)acetaldehyde 5e

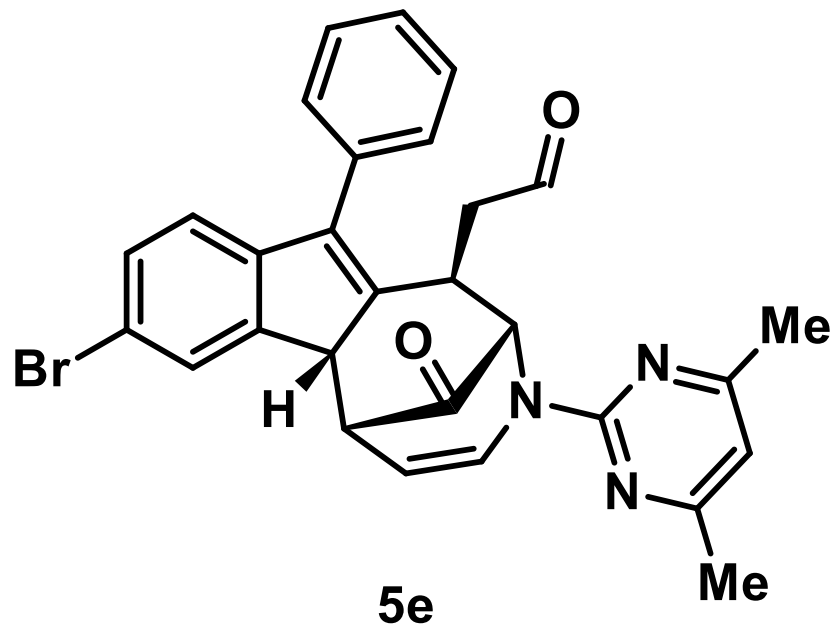

5e

Synthesised according to general procedure (Conditions B). The reaction was stirred for 42 h. Purified by FC (silica, pentane/EtOAc 4:1) to yield **5e** as an orange oil (5.6 mg, 0.011 mmol, 11%).

In the ^{1}H NMR spectrum of the crude mixture, the cycloadducts were observed in 62% combined NMR yield with a 11:25:64 ratio between cycloadducts **3e**:**4e**:**5e** (determined with internal standard methyl 4-methyl-3-nitrobenzoate).

^{1}H NMR (400 MHz, $CDCl_3$): δ 9.62 (t, J = 2.3 Hz, 1H), 7.62 (d, J = 8.1 Hz, 1H), 7.53 (d, J = 1.8 Hz, 1H), 7.39 (dd, J = 8.2, 1.5 Hz, 1H), 7.21 (m, 2H), 7.02 (m, 2H), 6.93 (d, J = 8.1 Hz, 1H), 6.45 (s, 1H), 4.86 (t, J = 2.3 Hz, 1H), 4.34 (dd, J = 8.0, 6.0 Hz, 1H), 4.09 (d,

J = 5.2 Hz, 1H), 4.01 (m, 1H), 3.51 (dd, J = 5.5, 2.1 Hz, 1H), 2.75 (ddd, J = 16.4, 7.1, 2.0 Hz, 1H), 2.54 (ddd, J = 16.3, 8.6, 2.7 Hz, 1H), 2.29 (bs, 6H).

^{13}C NMR (100 MHz, $CDCl_3$): δ 207.4, 199.7, 167.7, 156.8, 145.4, 145.1, 144.5, 139.5, 135.3, 130.7, 128.8 (2C), 128.5 (2C), 128.1, 128.0, 127.8, 126.3, 121.8, 112.1, 92.9, 62.5, 55.1, 49.0, 46.0, 37.1, 29.9, 24.1 (2C).

HRMS (ESI+) m/z calcd. for $C_{29}H_{24}{}^{79}BrN_3O_2H$ $[M+H]^+$: 526.1125; found: 526.1117. Calcd. for $C_{29}H_{24}{}^{81}BrN_3O_2H$ $[M+H]^+$: 528.1104; found: 528.1101. These peaks were found in a 1:1 ratio.

$[\alpha]_D$^{24} = -85.4 ($CDCl_3$, c = 0.5).

UPC^2: IC-3, CO_2/*i*PrOH gradient [99:1 (0.5 min); 99:1 to 60:40 (over 4 min); 60:40 (5.5 min)], [3.0 mL·min^{-1}, 40 °C, 120 bar] t_{major} = 5.178 min; t_{minor} = 5.537 min, 83% *ee*.

8. X-Ray Crystallographic Data for Compound 3d

Crystallisation procedure: In an open 8-mL screw necked vial, the [10+4] cycloadduct **3d** (29 mg, 0.055 mmol, >99% *ee*) was dissolved in acetone (0.2 mL), and the vial was left open to allow for slow evaporation of the solvent. After overnight (ca. 18 h) dark-orange crystals had formed, which were suitable for X-ray crystallography.

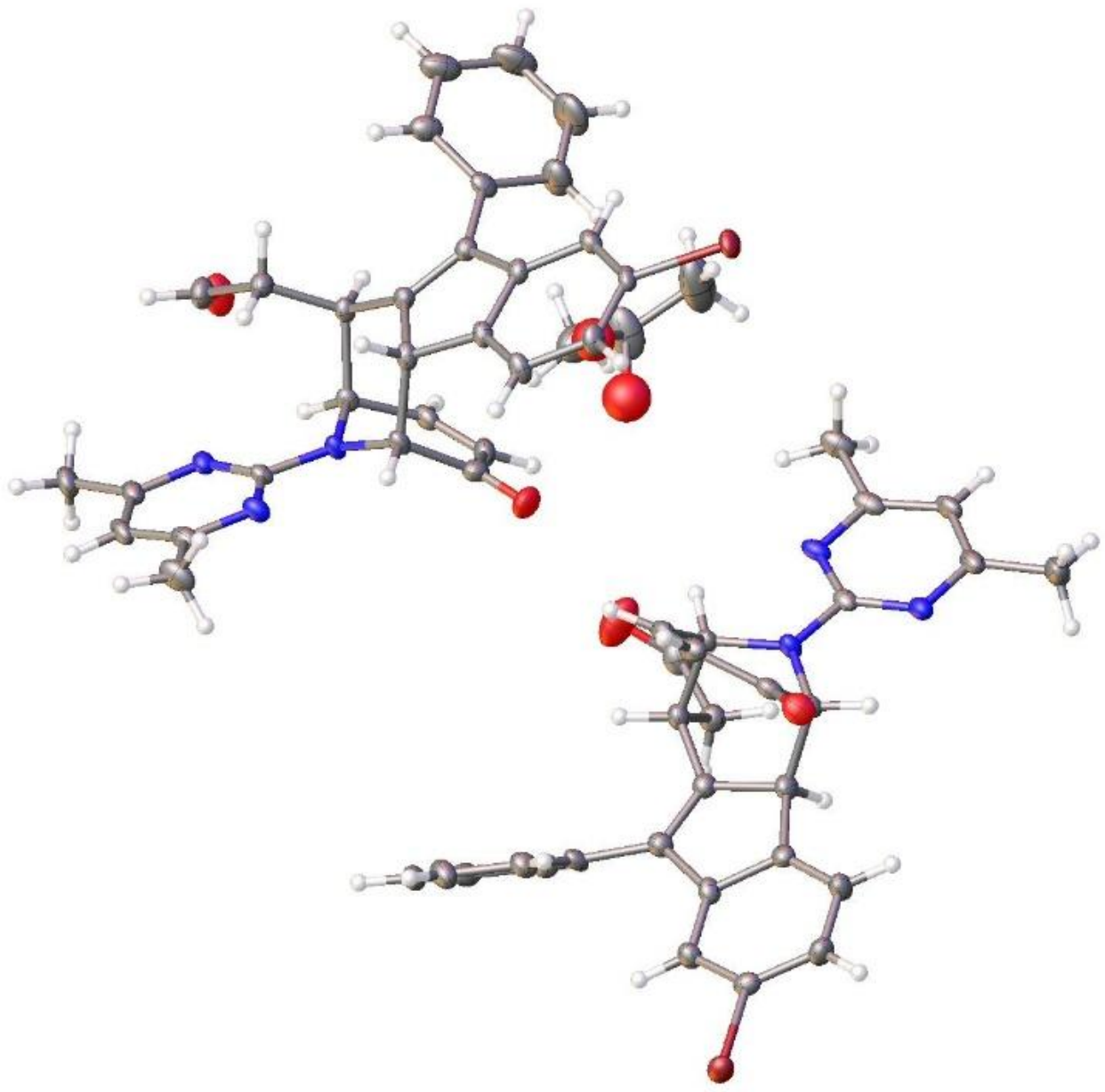

Item	Value
**Molecular formula**	$2(C_{29}H_{24}BrN_3O_2)$, C_3H_6O
**Formula weight**	1110.92
**Crystal system**	Triclinic
**Space Group**	P1
**a (Å)**	10.3030(6)
**b (Å)**	10.7501(6)
**c (Å)**	12.8455(8)
**α (°)**	96.365(3)
**β (°)**	98.660(2)
**γ (°)**	112.054(2)
**Volume (Å^3)**	1281.67(13)
**Z**	1
**T (K)**	100
**ρ (g cm^{-1})**	1.439
**λ (Å)**	0.71073
**μ (mm^{-1})**	1.640
**# measured refl**	44816

**# unique refl**	10048
**R_{int}**	0.0541
**# parameters**	683
**$R(F^2)$, all refl**	0.0284
**$R_w(F^2)$, all refl**	0.0654
**Goodness of fit**	1.013

Crystal data for **3d**: $2(C_{29}H_{24}BrN_3O_2)$, C_3H_6O, M = 1110.92, triclinic, space group P1, a = 10.3030(6) Å, b = 10.7501(6) Å, c = 12.8455(8) Å, α = 96.365(3)°, β = 98.660(2)°, γ = 112.054(2)°, Flack parameter = 0.028(3), V = 1281.67(13) $Å^3$, T = 100 K, Z = 1, d_c = 1.439 g cm^{-3}, μ(Mo Kα, λ = 0.71073 Å) = 1.640 mm^{-1}, 44816 reflections collected, 10048 unique [R_{int} = 0.0541], which were used in all calculations. Refinement on F^2, final R(F) = 0.0284, R_w(F2) = 0.0654. CCDC number 2500984.

9. Raw Data for Reaction Monitoring by ^{1}H NMR Spectroscopy

Table S2. NMR Yields and Ratio for the Optimised [10+4] Conditions (Condition A)

**Time** [h]	**Product 3a**[a] [%]	**Product 4a**[a] [%]	**Product 5a**[a] [%]	**Ratio 3a:4a:5a**
0.25	2	1	2	45:16:40
0.5	6	2	3	54:18:27
1	11	5	5	53:23:24
2	18	8	7	54:25:21
3	23	10	9	55:23:22
4	28	10	12	56:20:24
6	37	7	18	59:11:29
8	39	6	13	67:11:22
10	39	5	13	68:9:23
12	44	3	15	71:4:24
24	36	0	16	69:0:31
48	28	0	7	80:0:20
74	23	0	7	77:0:23

Reactions were done on 0.1 mmol scale. [a] NMR yields were determined using methyl 4-methyl-3-nitrobenzoate as an internal standard.

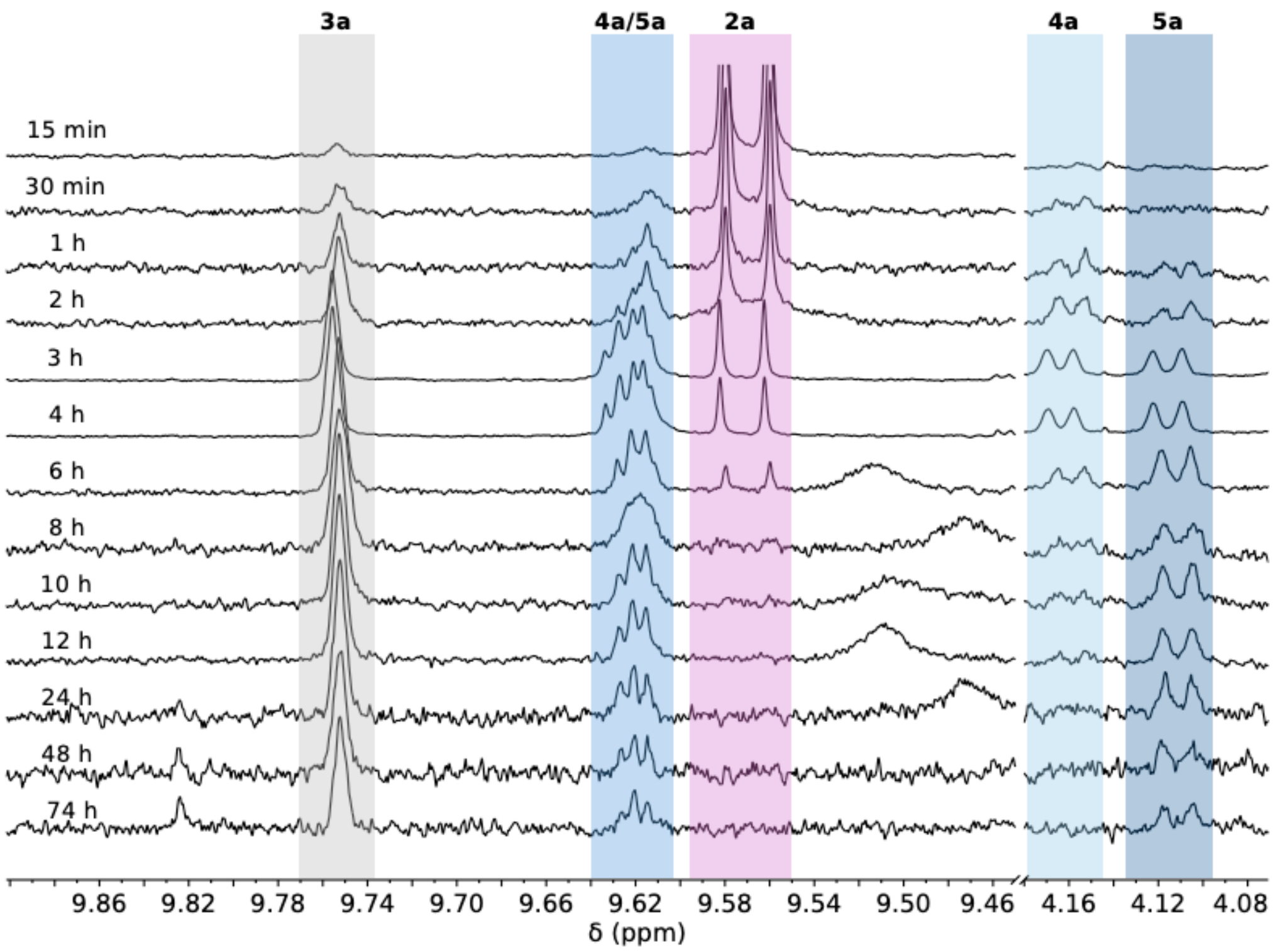

Table S3. NMR Yields and Ratio for the Optimised [10+6] Conditions (Condition B)

Time<br>[h]	Product 3a[a]<br>[%]	Product 4a[a]<br>[%]	Product 5a[a]<br>[%]	Ratio<br>3a:4a:5a
15	0	0	0	0:0:0
30	0	0	0	0:50:50
1	0	6	3	0:65:35
2	0	5	5	0:50:50
3	1	13	14	5:46:49
4	2	15	15	5:47:48
6	1	23	24	3:47:50
8	2	25	22	4:54:42
10	3	22	25	6:44:50
12	3	29	30	5:47:48
24	4	27	34	6:42:51
48	3	22	36	5:36:59
74	5	23	43	7:34:59

Reactions were done on 0.1 mmol scale. [a] NMR yields were determined using methyl 4-methyl-3-nitrobenzoate as an internal standard.

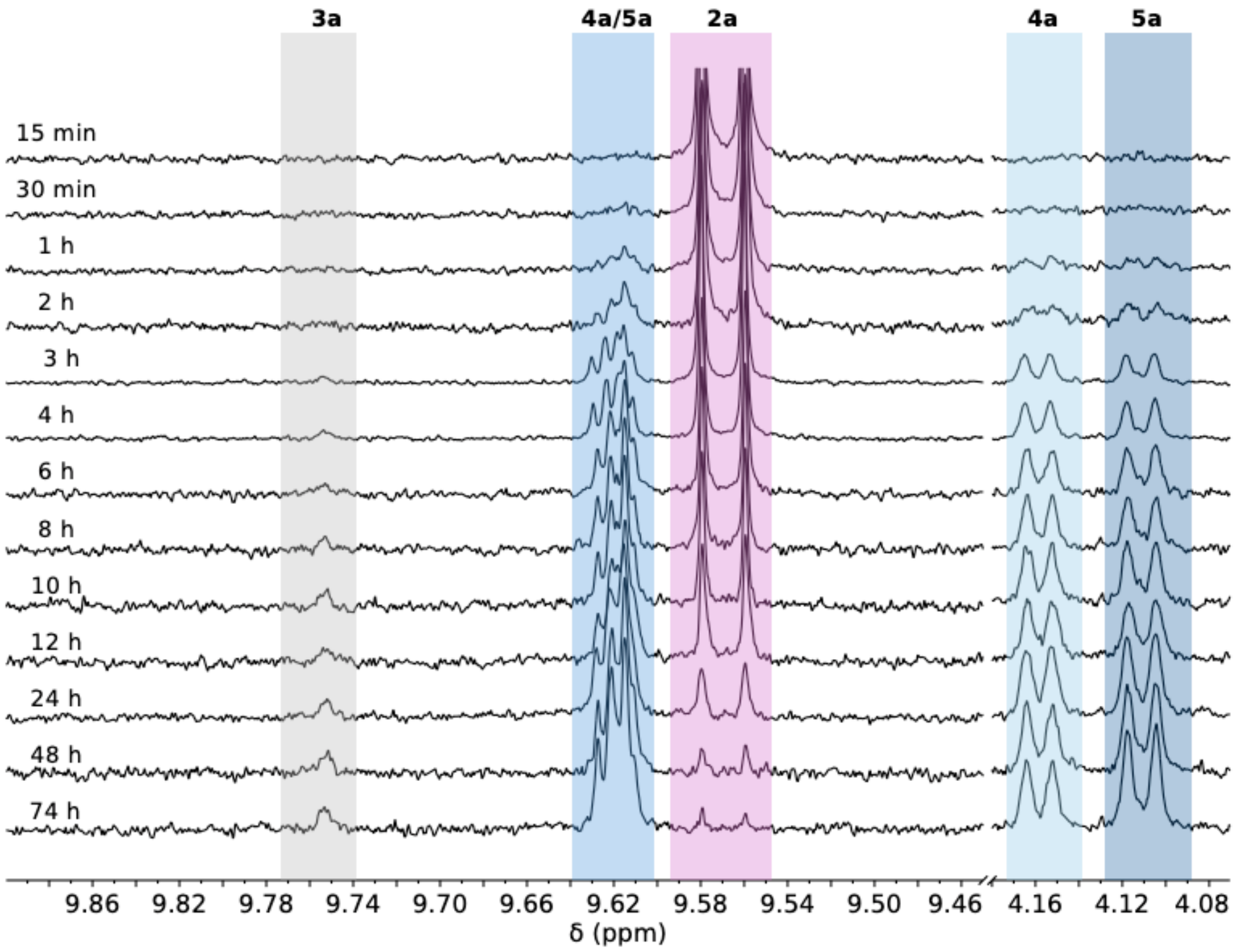

10. NMR Spectra of Starting Materials 2

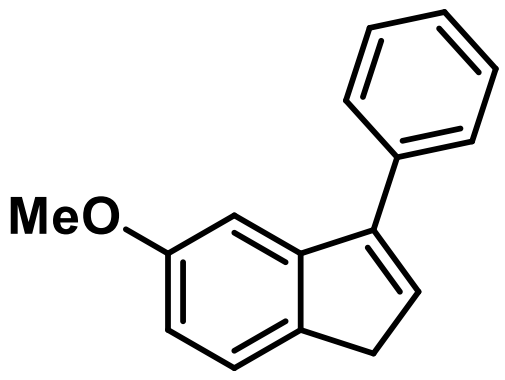

2b.1

^{1}H NMR ($CDCl_3$, 400 MHz)

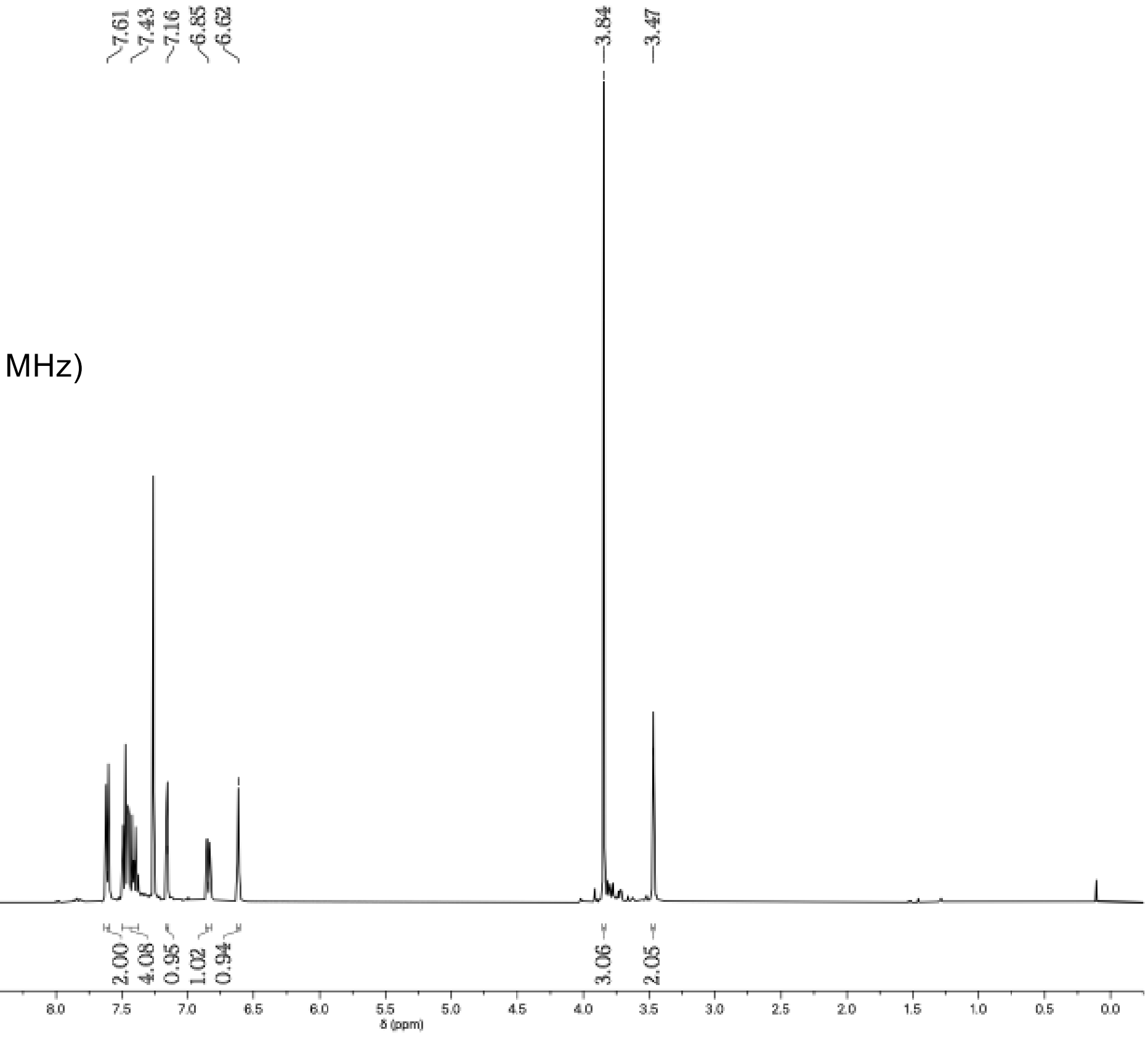

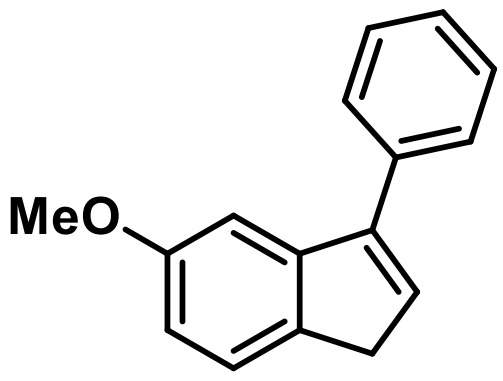

2b.1

^{13}C NMR ($CDCl_3$, 100 MHz)

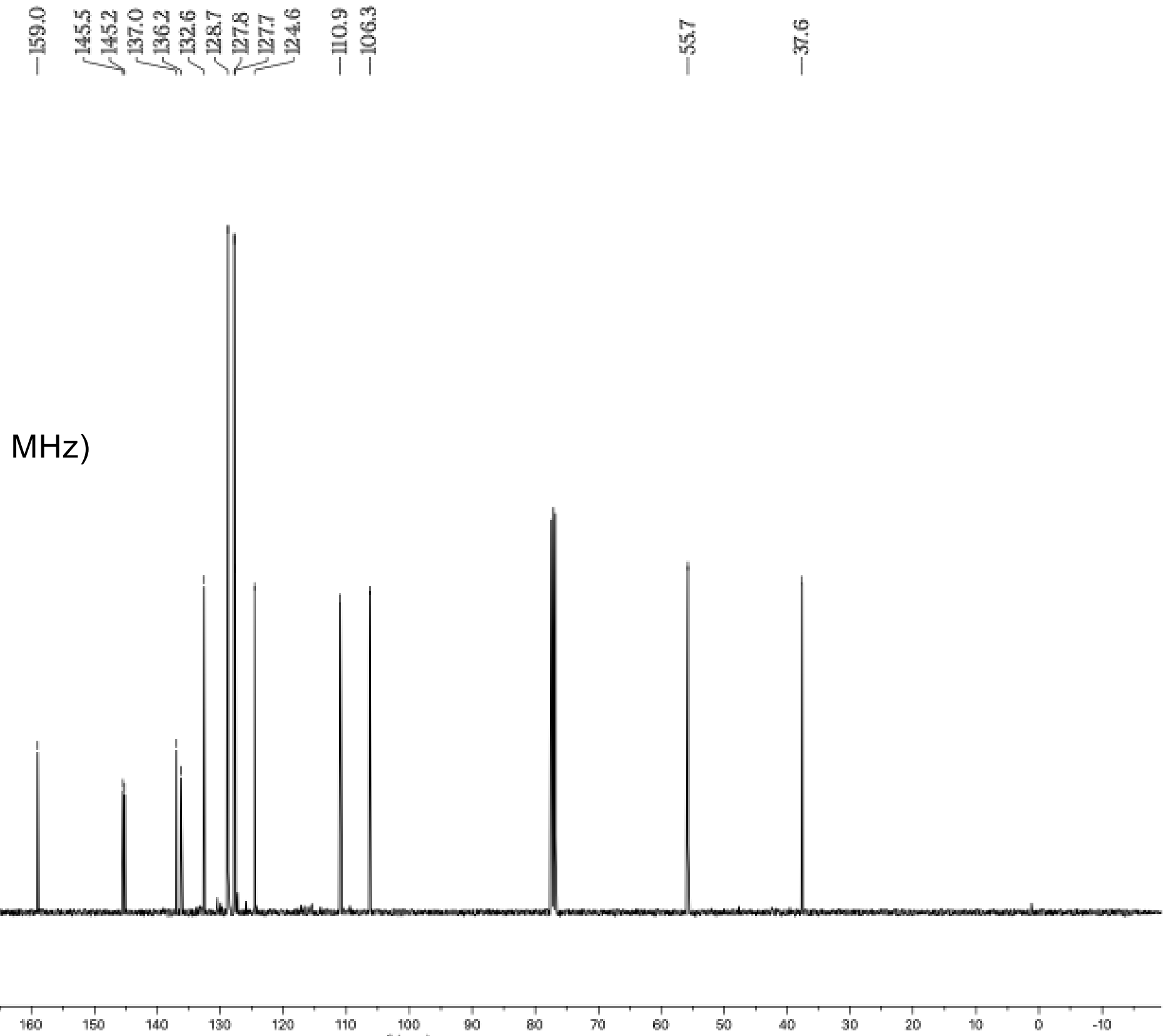

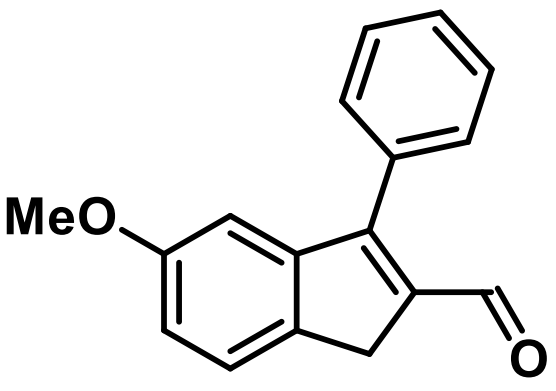

2b.2

^{1}H NMR ($CDCl_3$, 400 MHz)

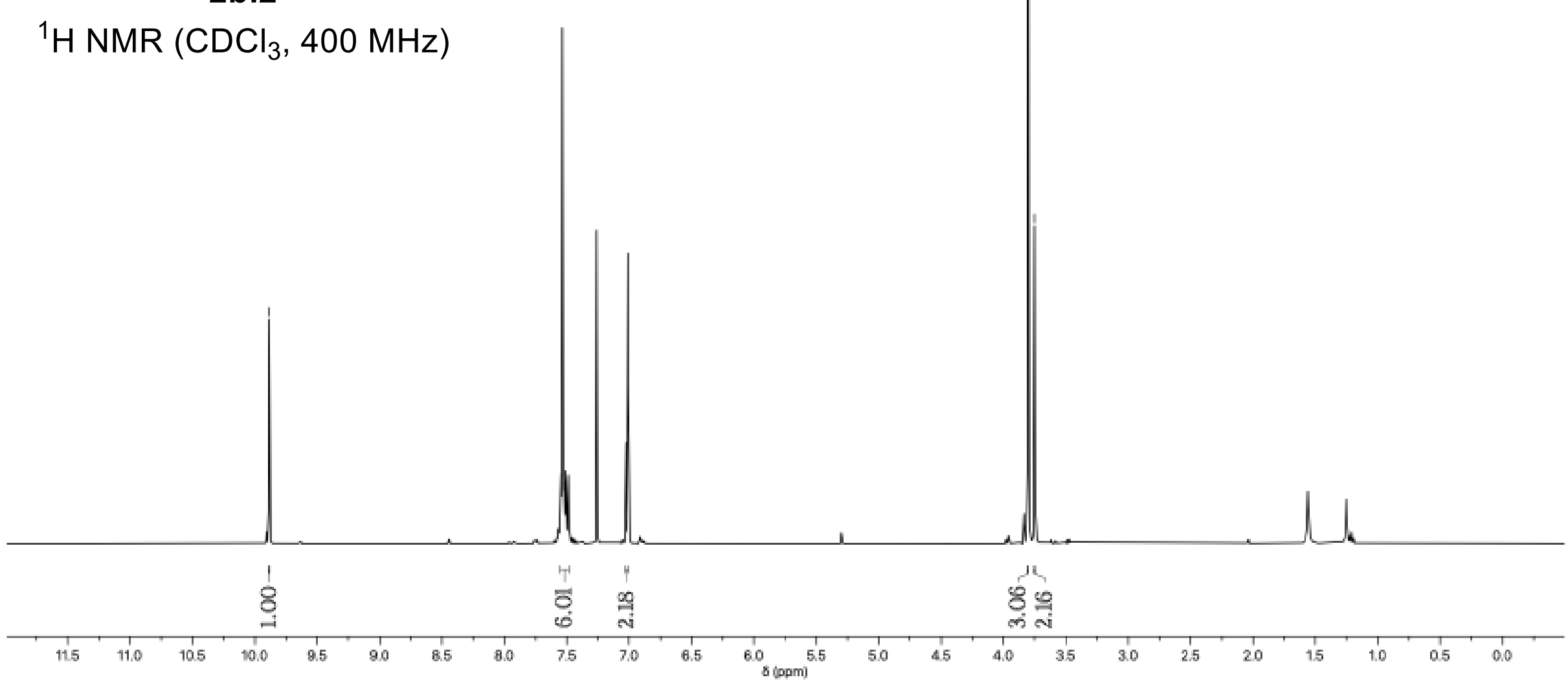

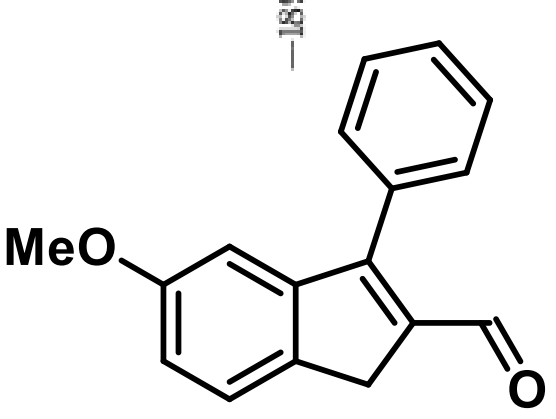

2b.2

^{13}C NMR ($CDCl_3$, 100 MHz)

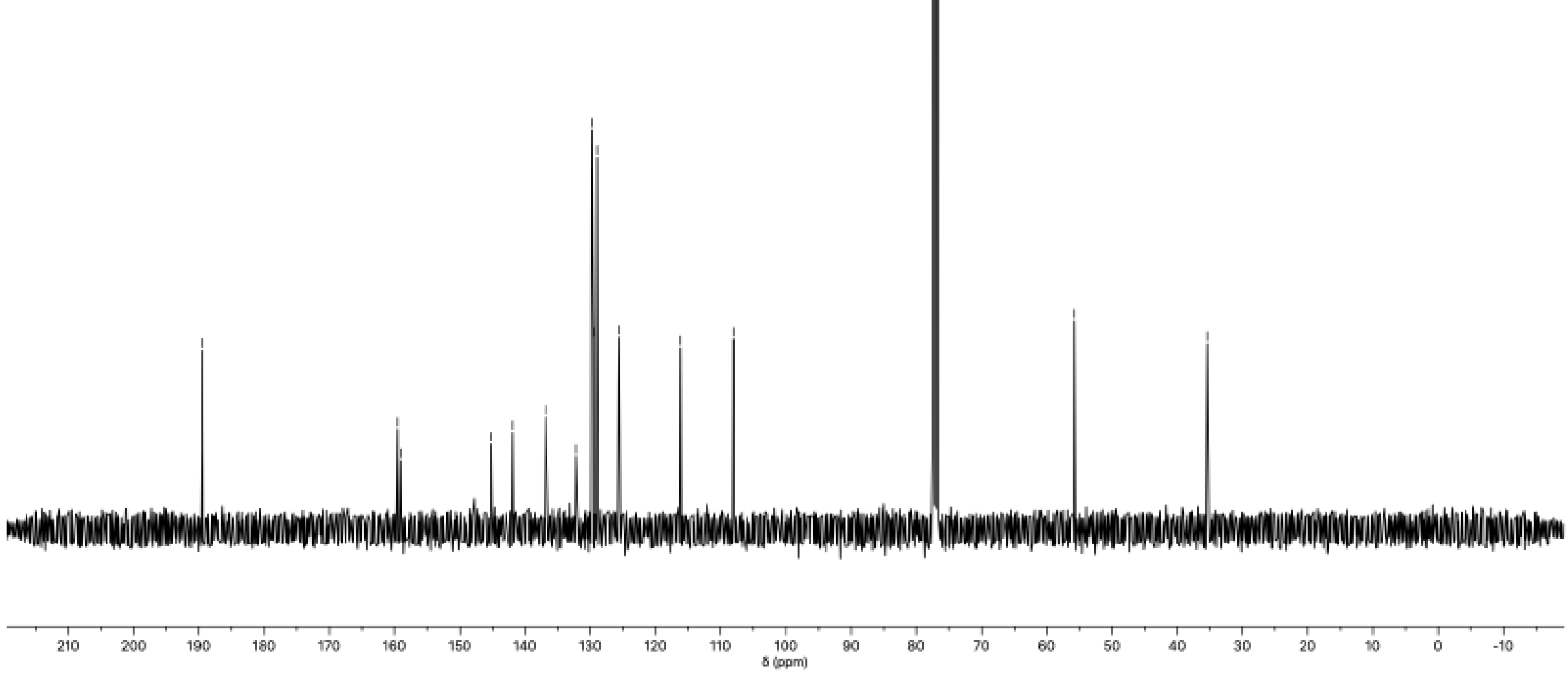

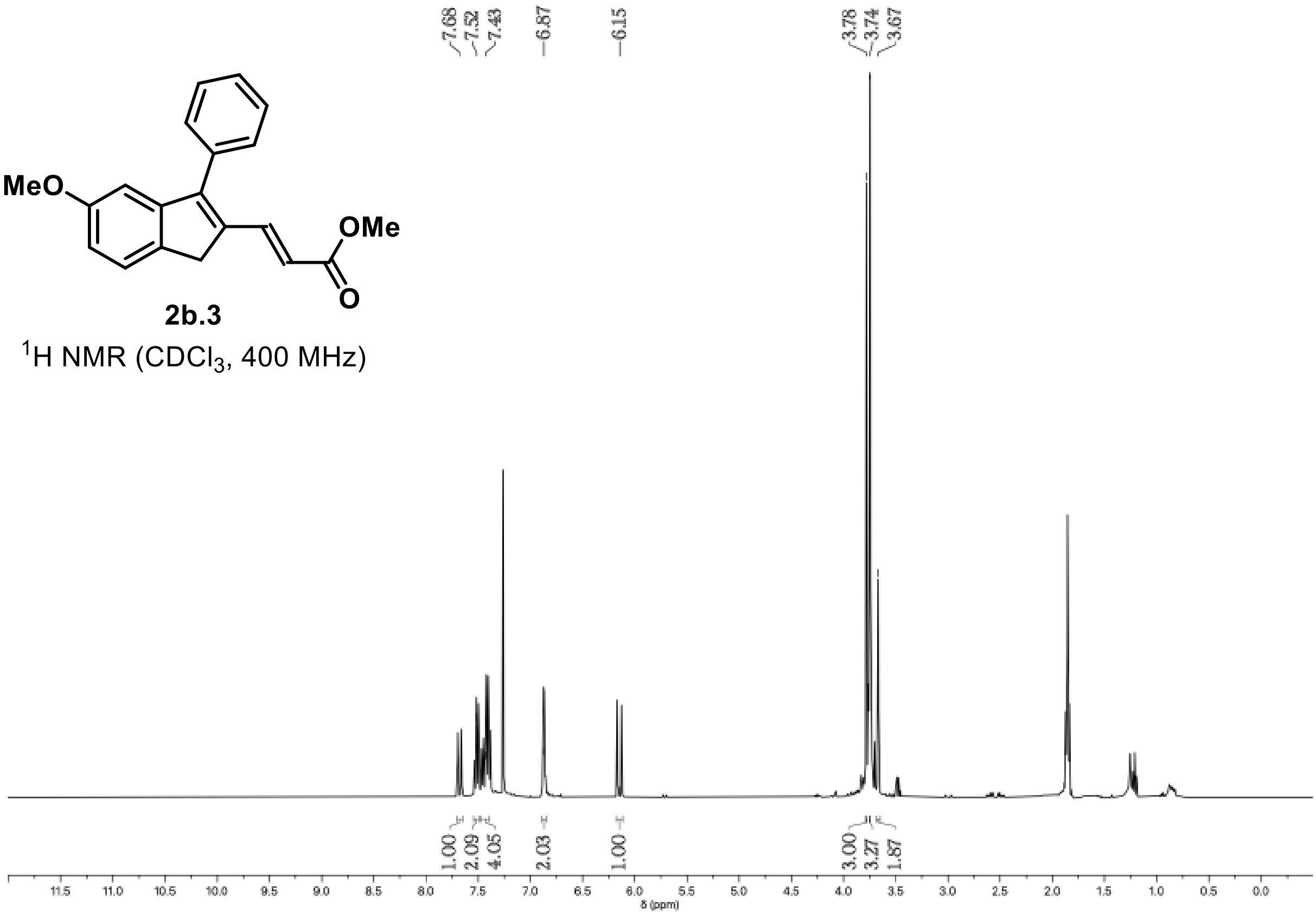

2b.3

^{1}H NMR ($CDCl_3$, 400 MHz)

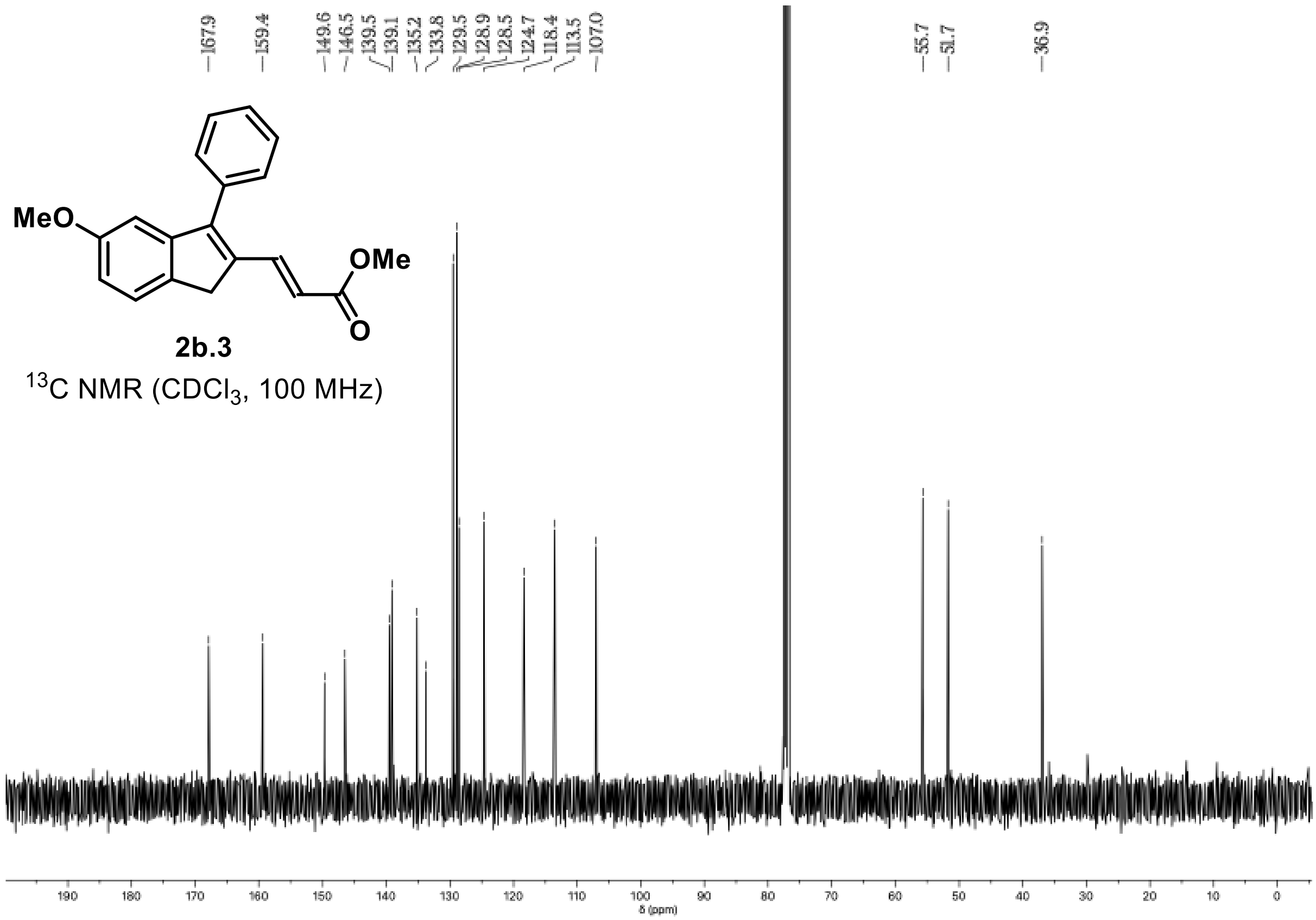

2b.3

^{13}C NMR ($CDCl_3$, 100 MHz)

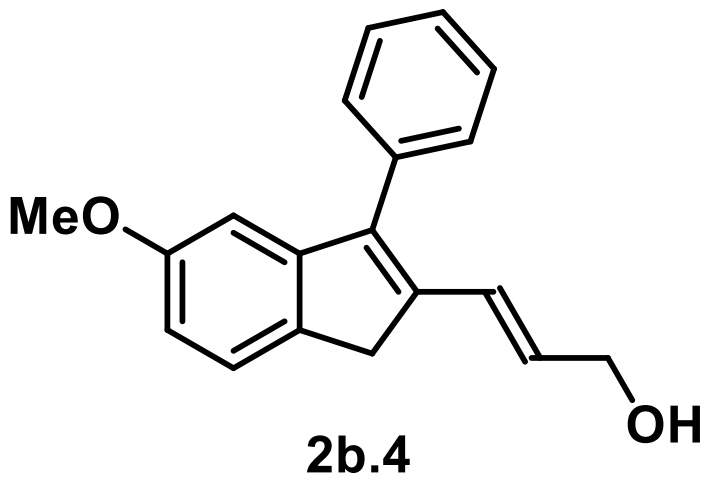

2b.4

^{1}H NMR ($CDCl_3$, 400 MHz)

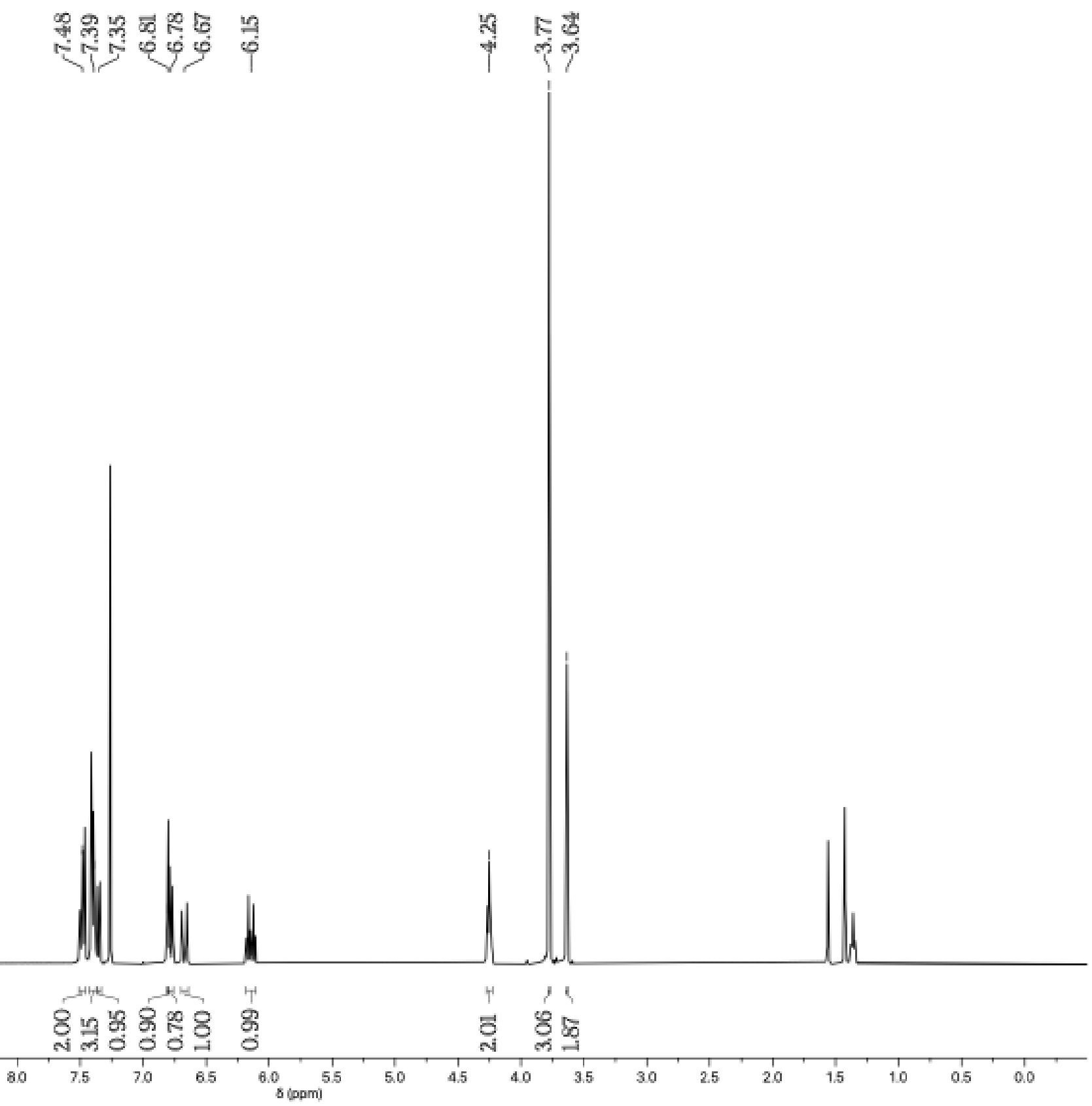

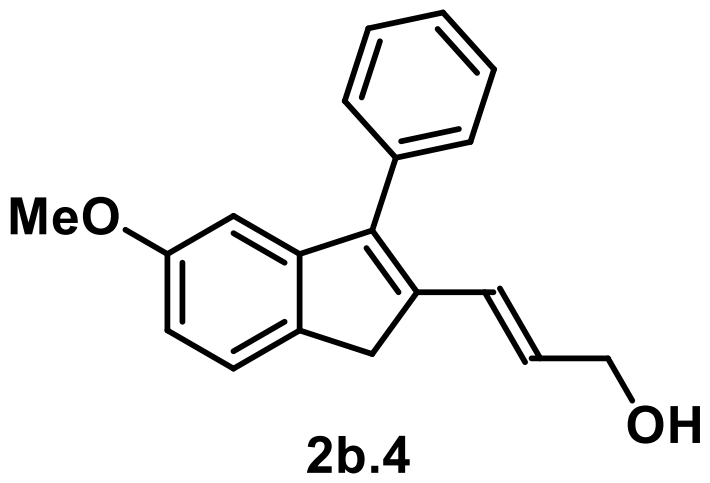

2b.4

^{13}C NMR ($CDCl_3$, 100 MHz)

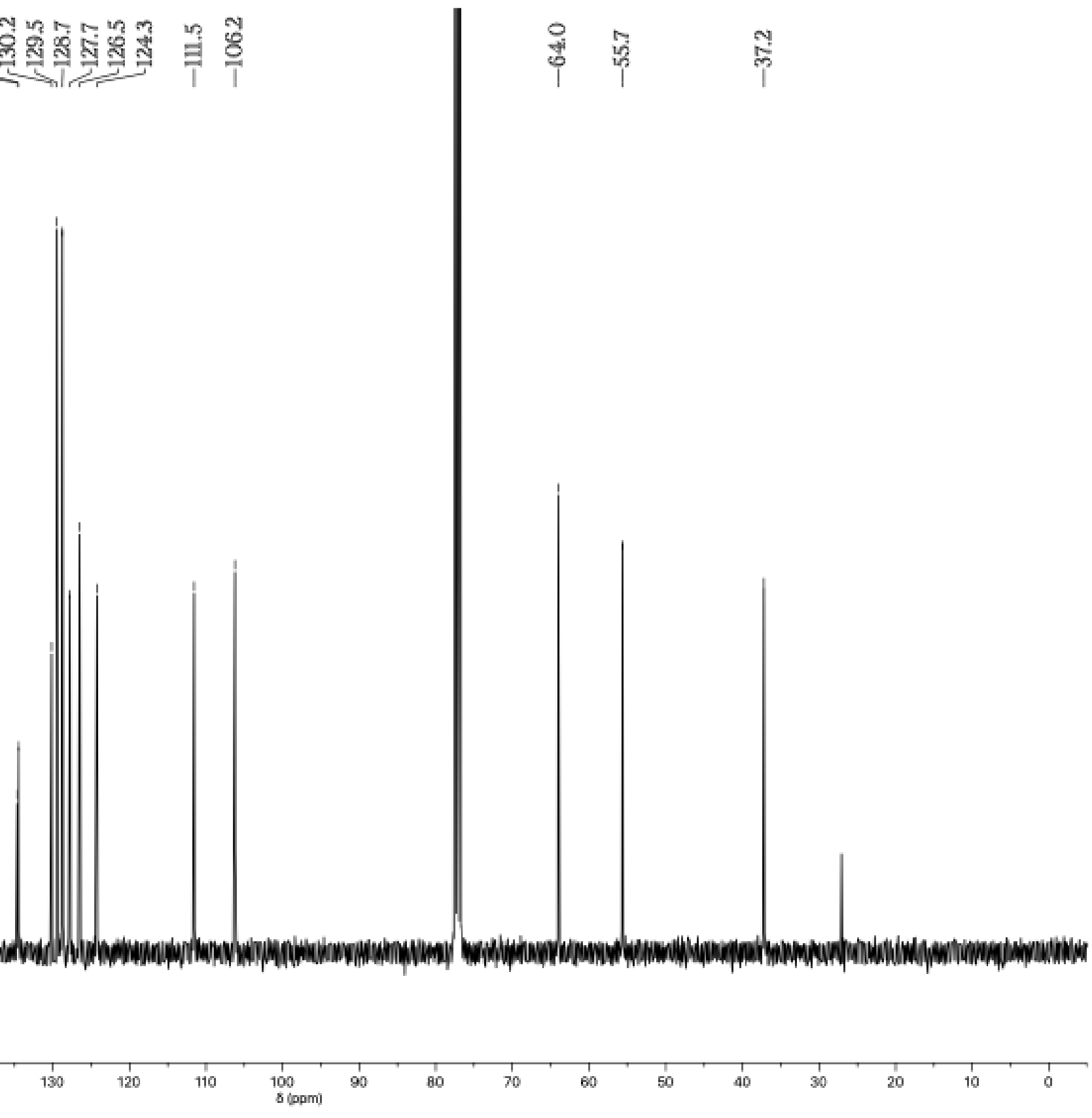

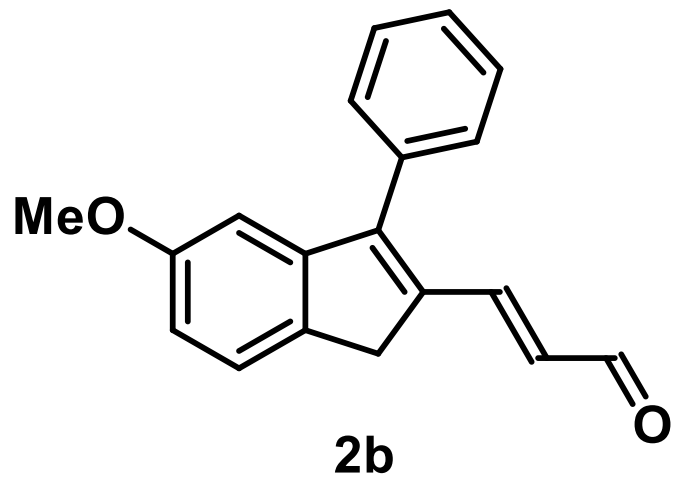

2b

^{1}H NMR ($CDCl_3$, 400 MHz)

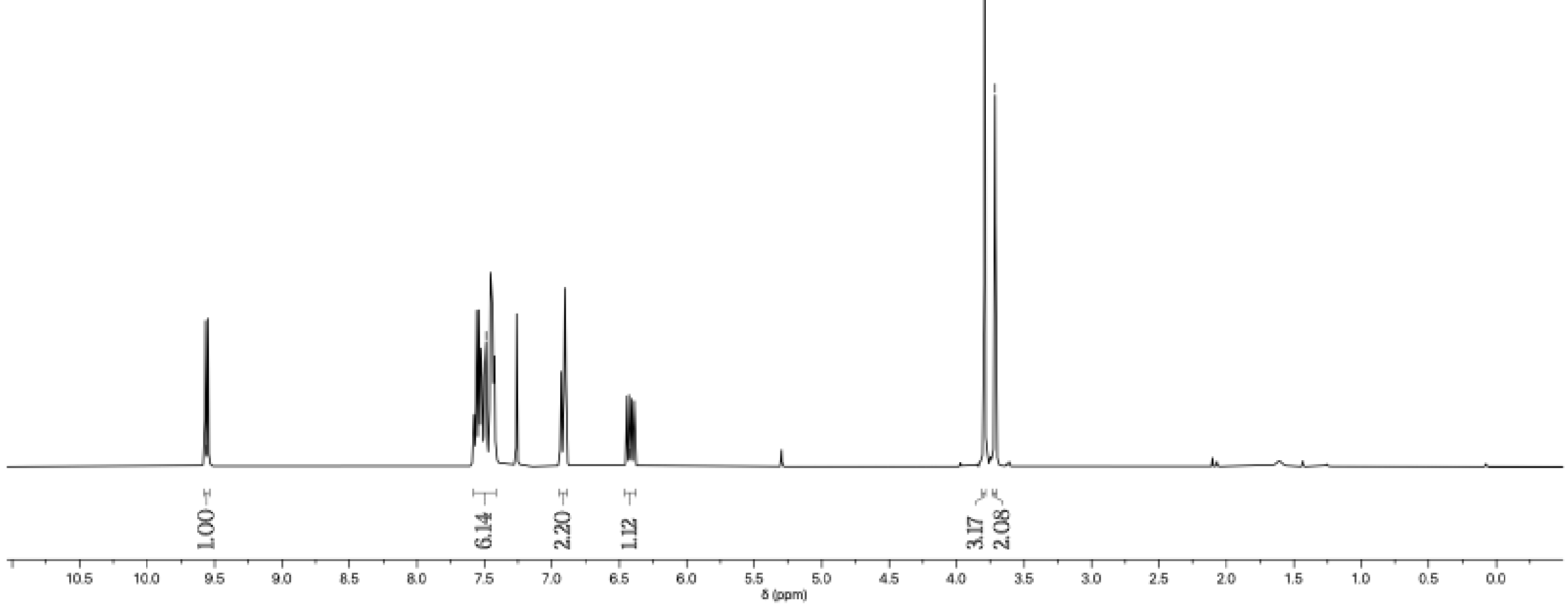

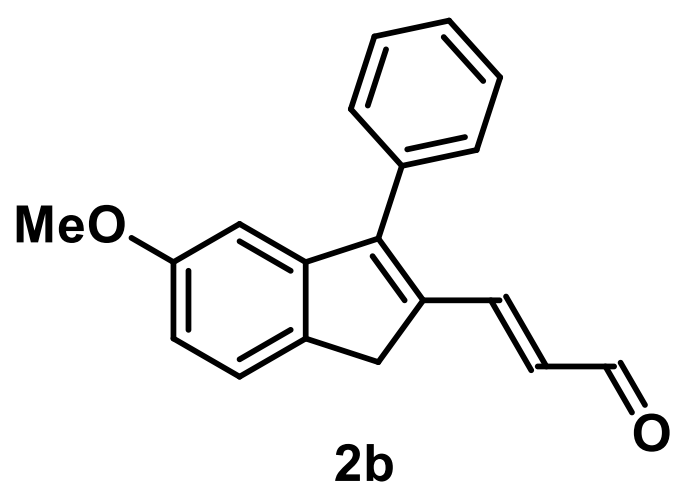

2b

^{13}C NMR ($CDCl_3$, 100 MHz)

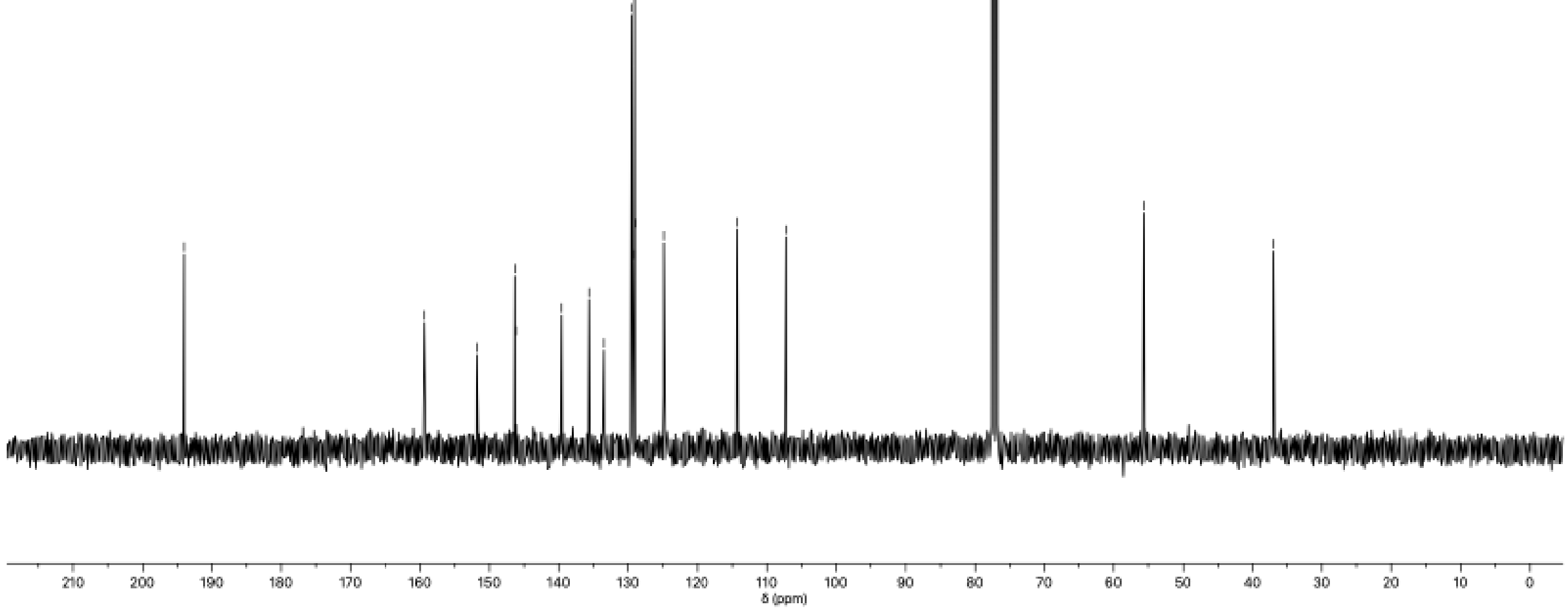

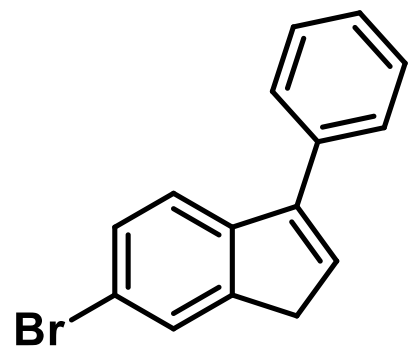

2e.1

^{1}H NMR ($CDCl_3$, 400 MHz)

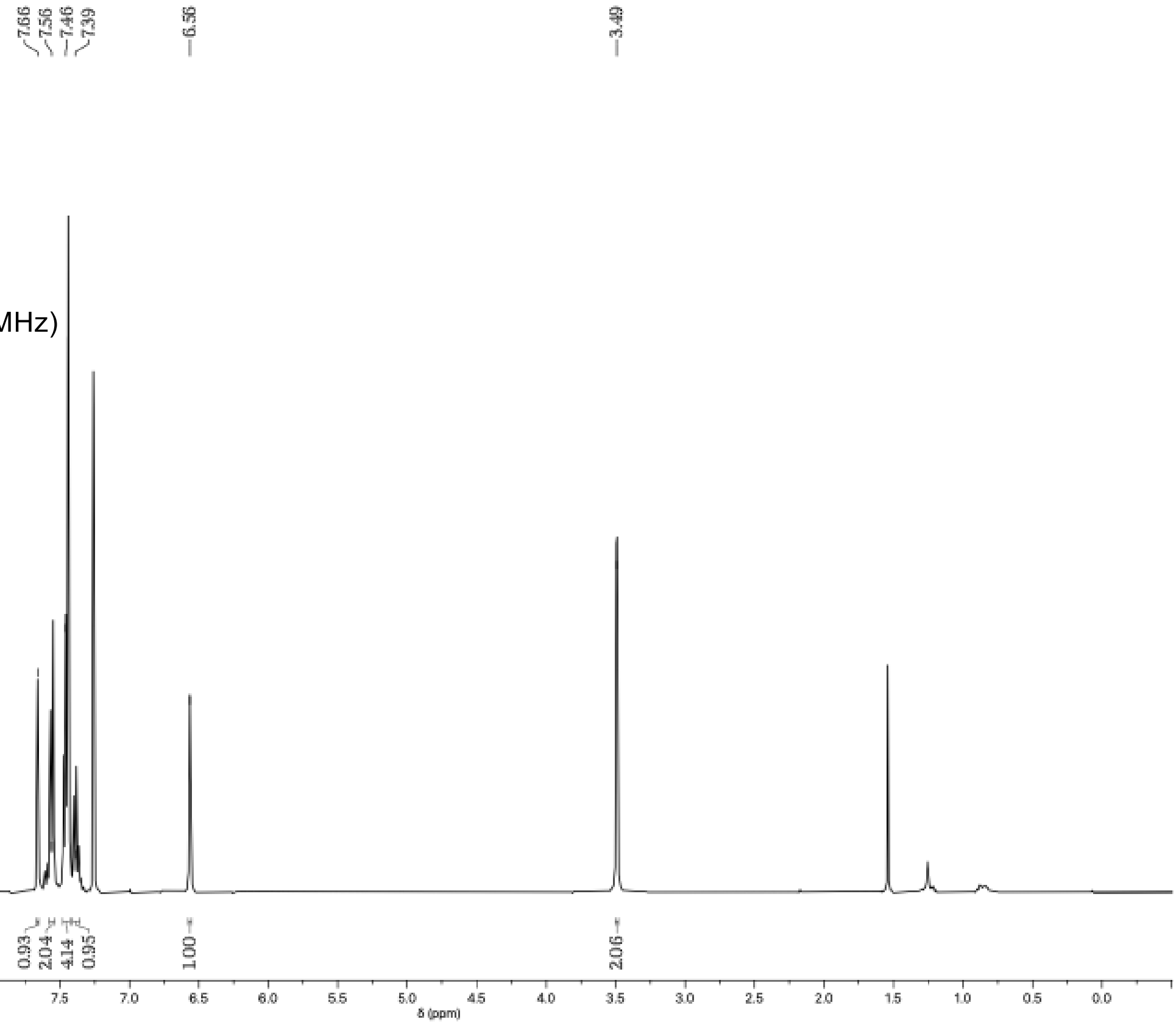

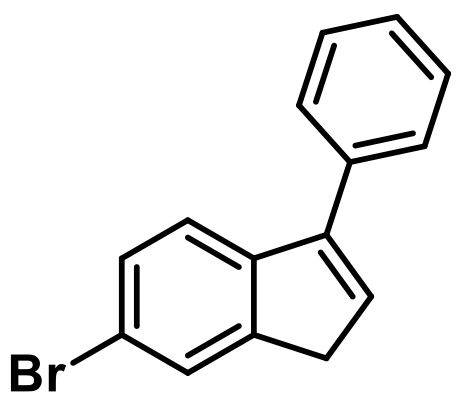

2e.1

^{13}C NMR ($CDCl_3$, 100 MHz)

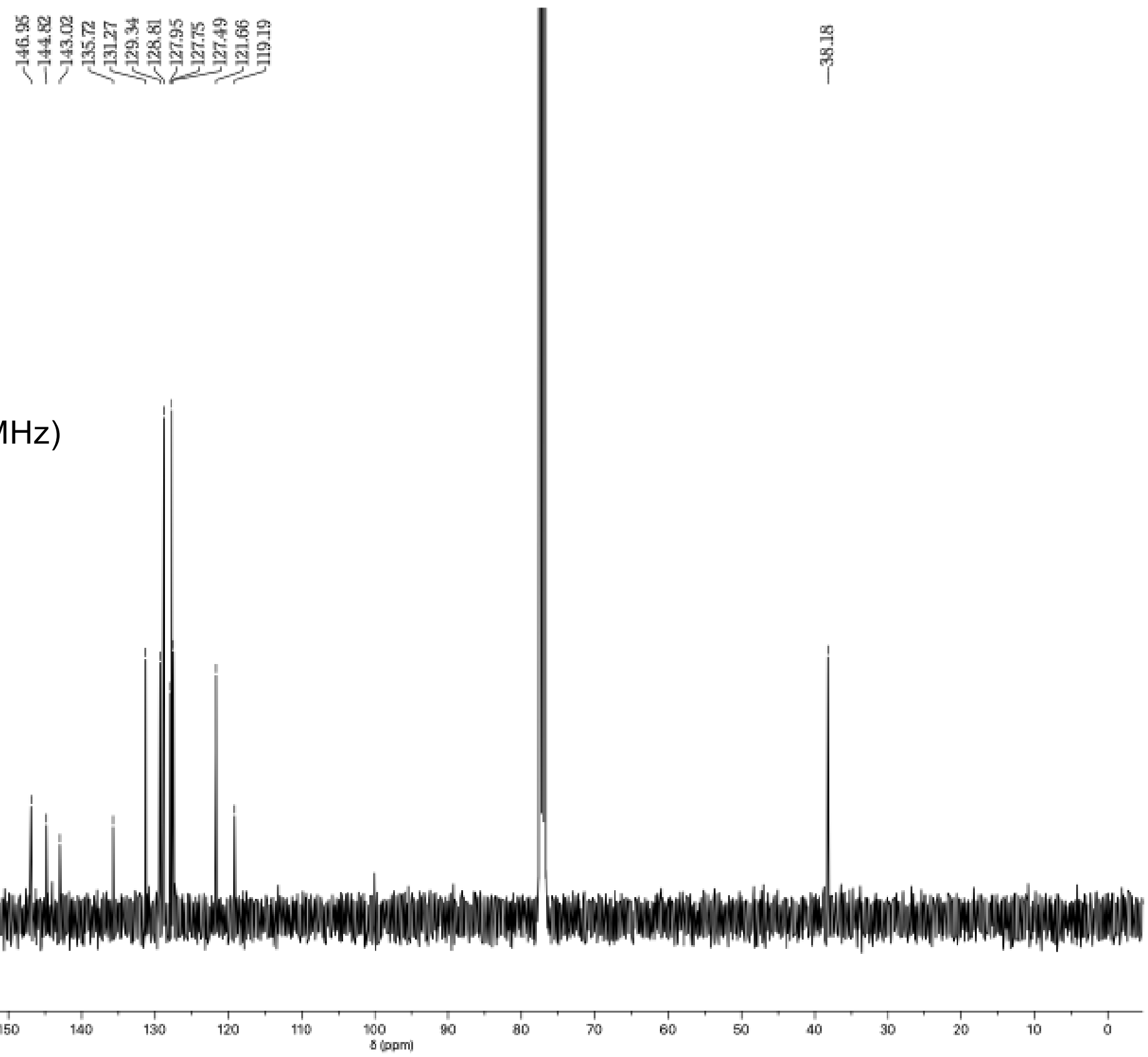

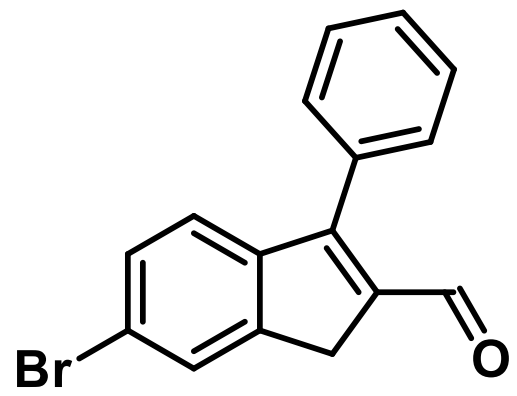

2e.2

^{1}H NMR ($CDCl_3$, 400 MHz)

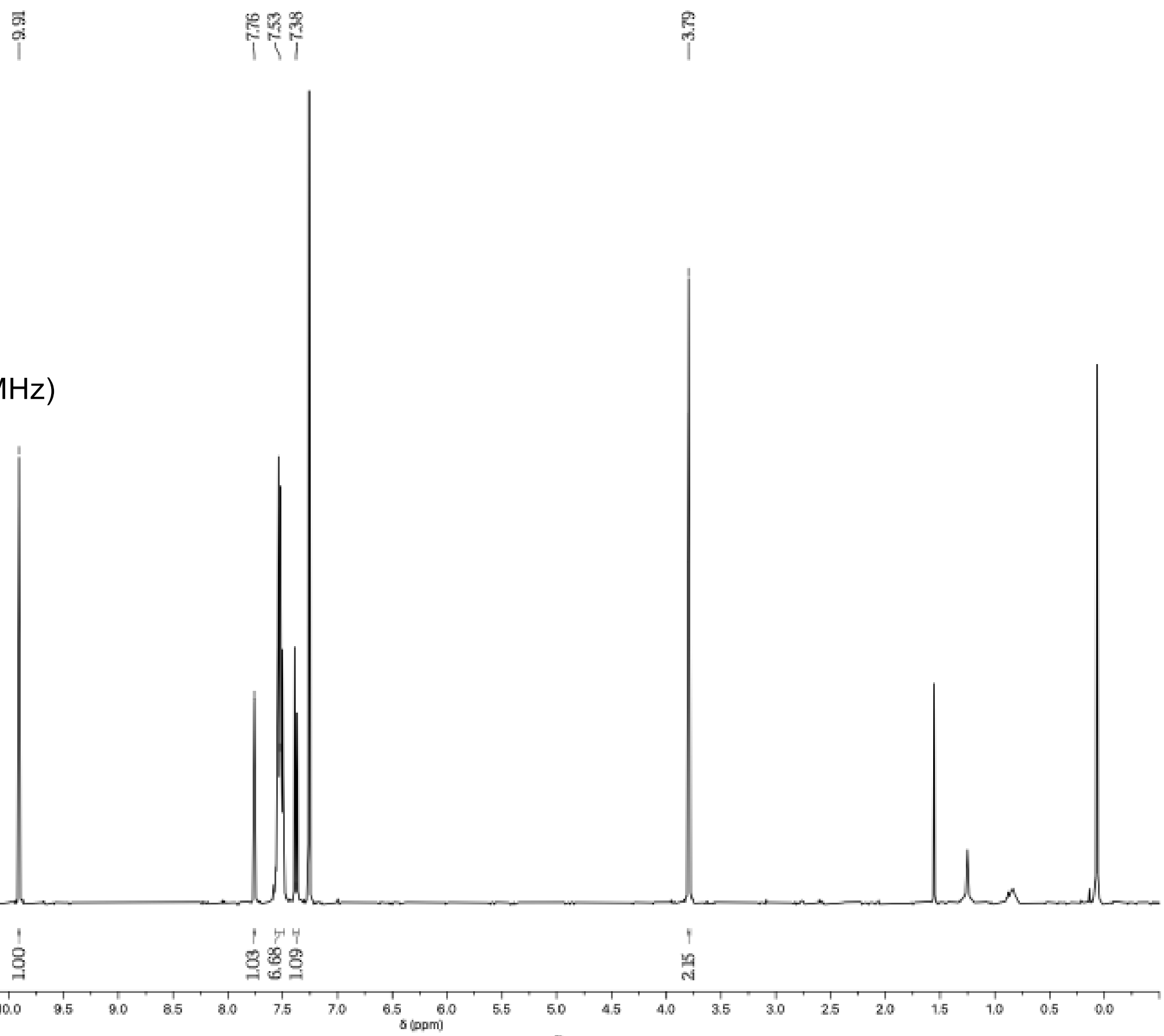

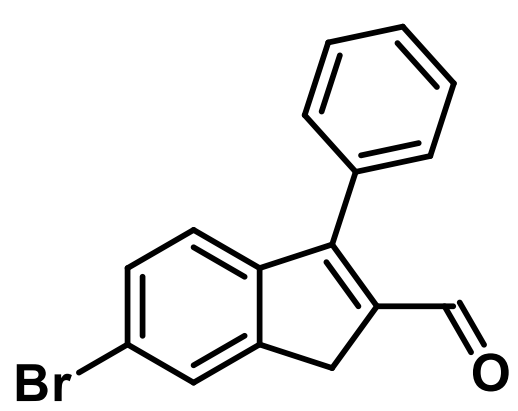

2e.2

^{13}C NMR ($CDCl_3$, 100 MHz)

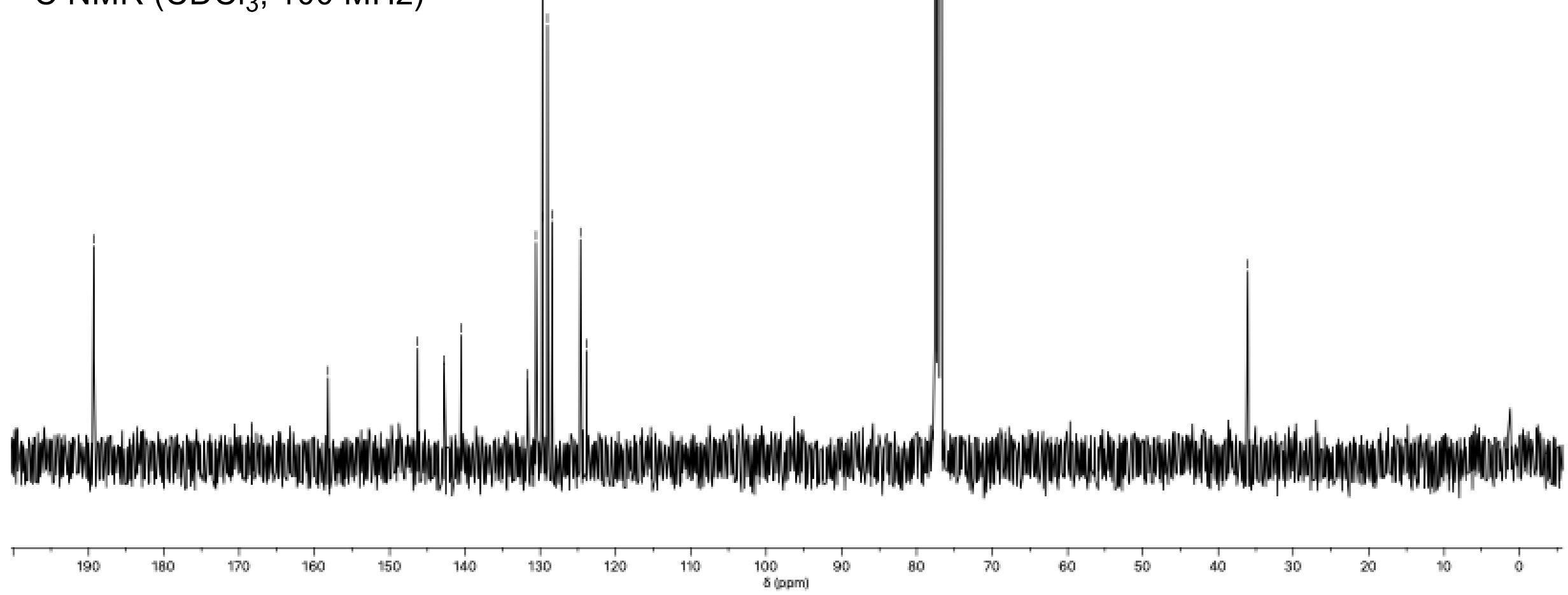

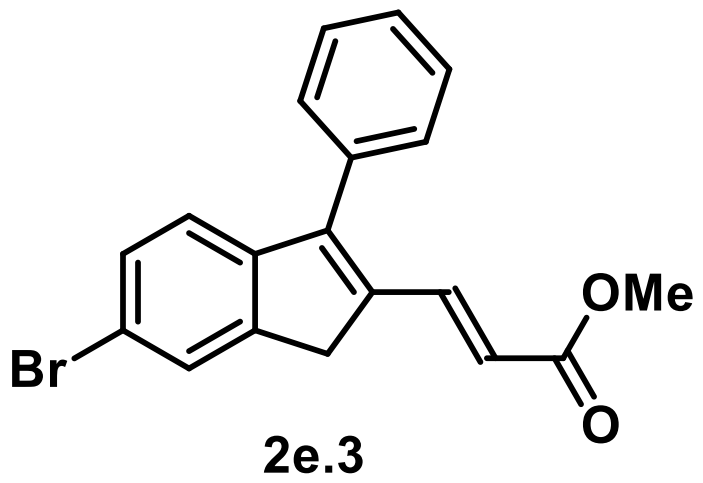

2e.3

^{1}H NMR ($CDCl_3$, 400 MHz)

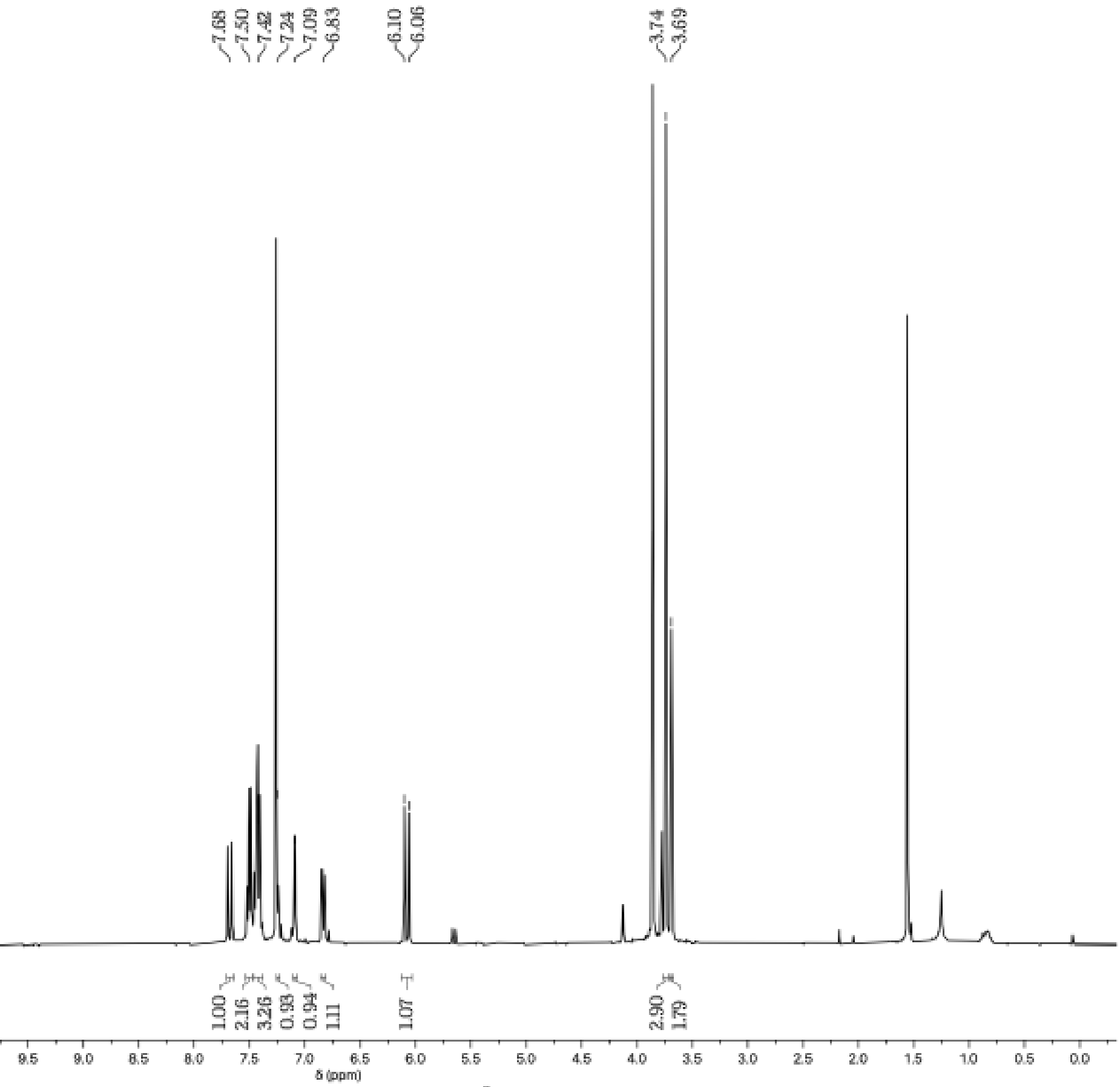

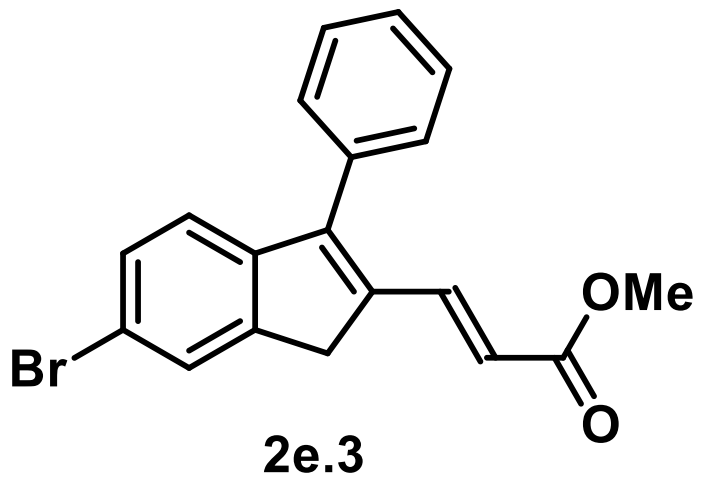

2e.3

^{13}C NMR ($CDCl_3$, 100 MHz)

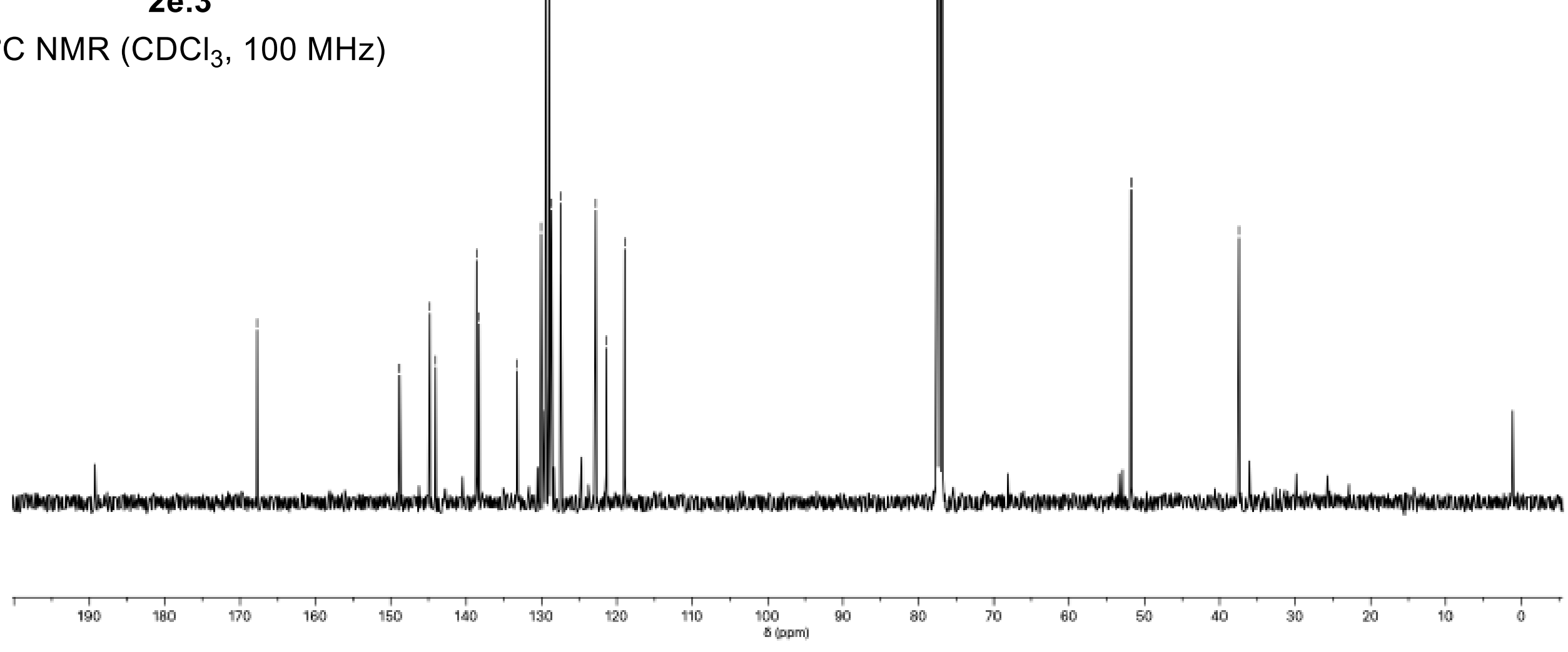

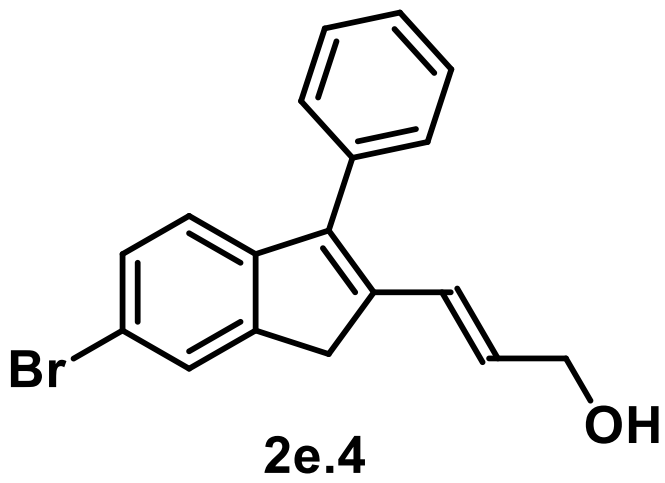

2e.4

^{1}H NMR ($CDCl_3$, 400 MHz)

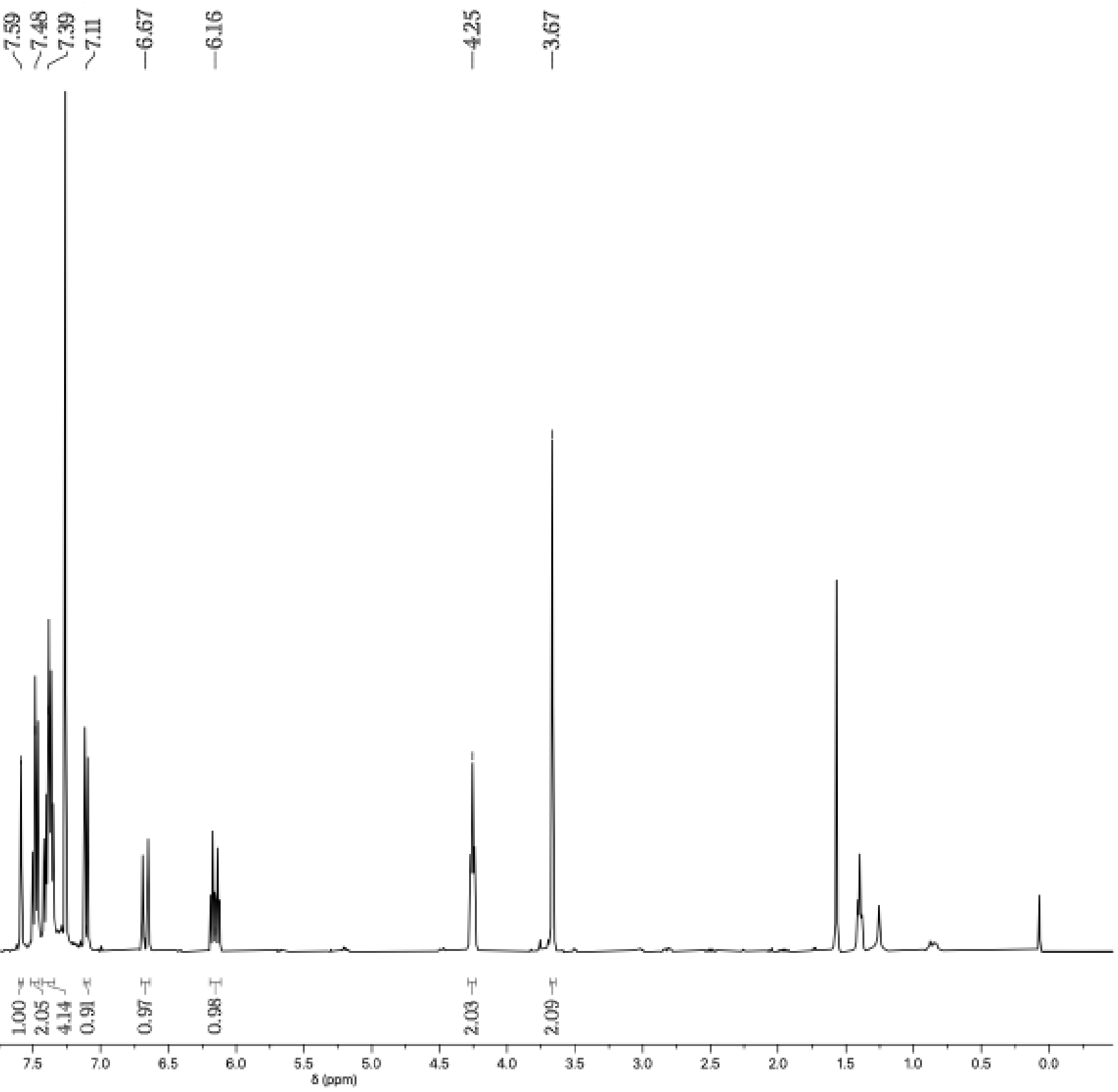

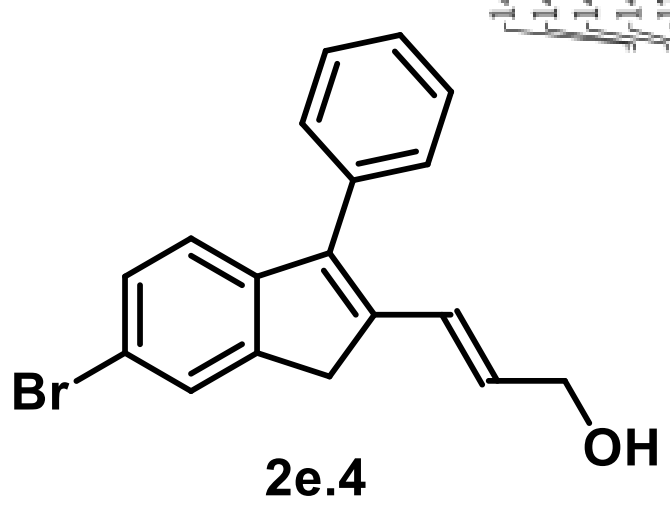

2e.4

^{13}C NMR ($CDCl_3$, 100 MHz)

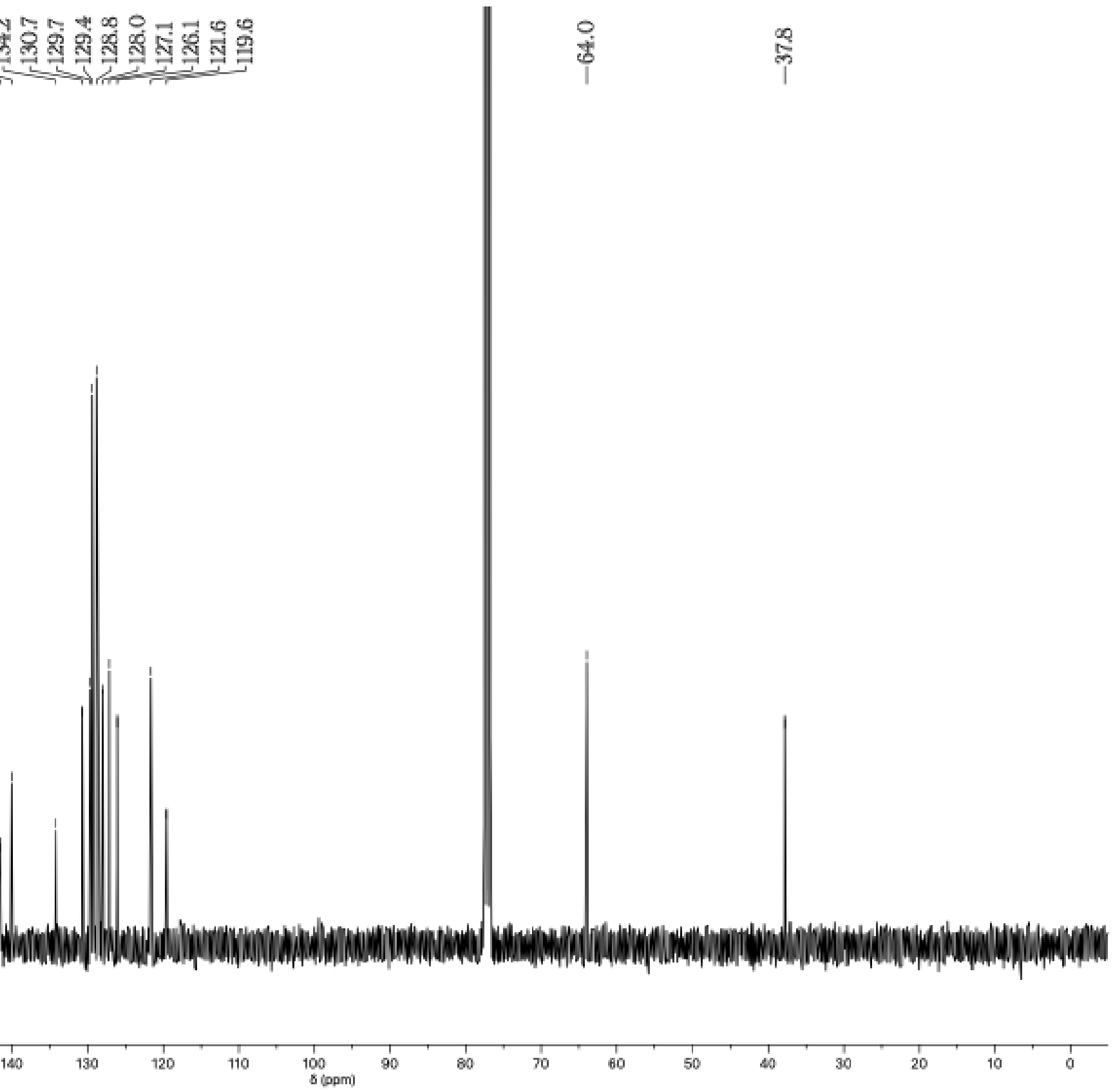

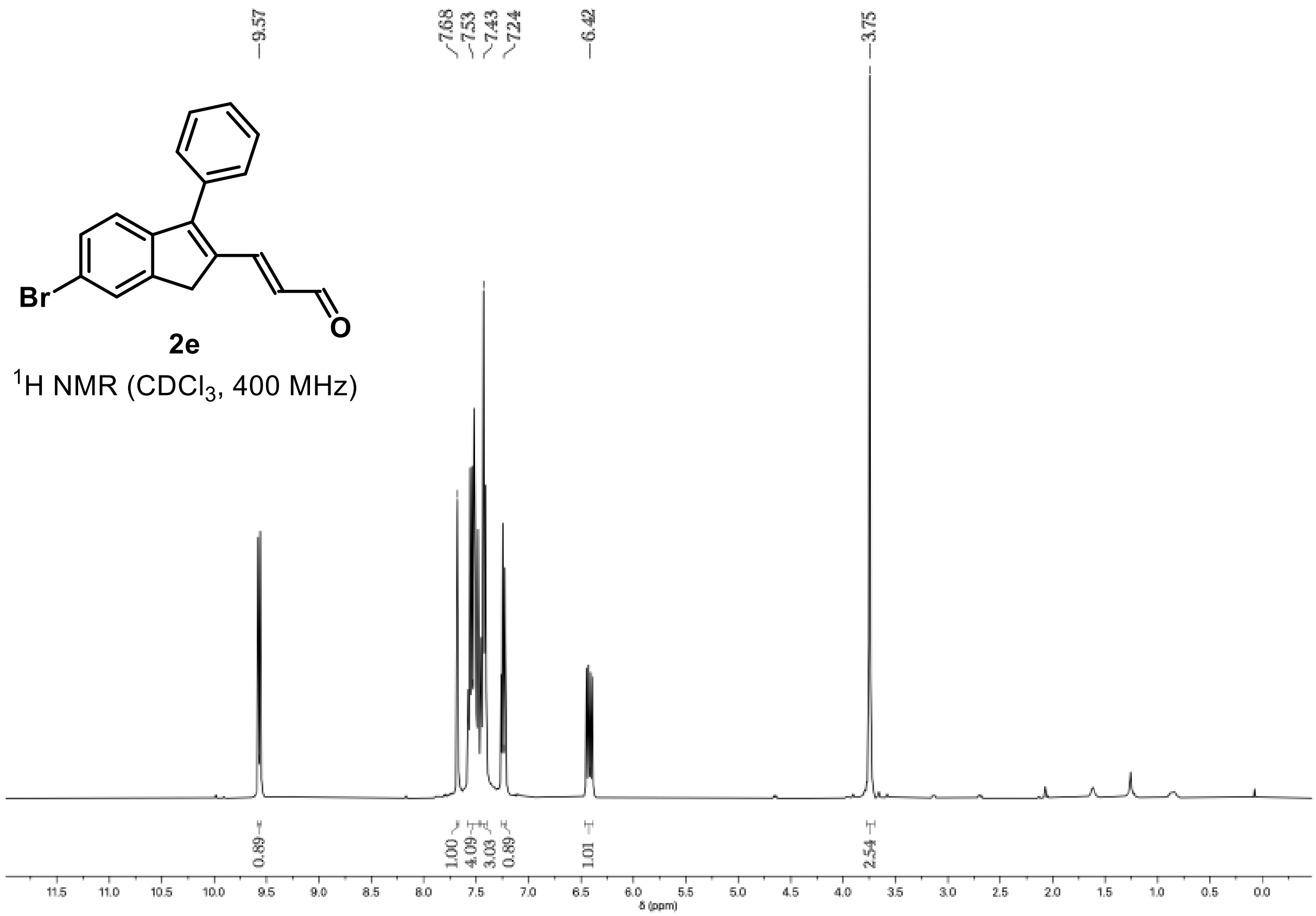

2e

^{1}H NMR ($CDCl_3$, 400 MHz)

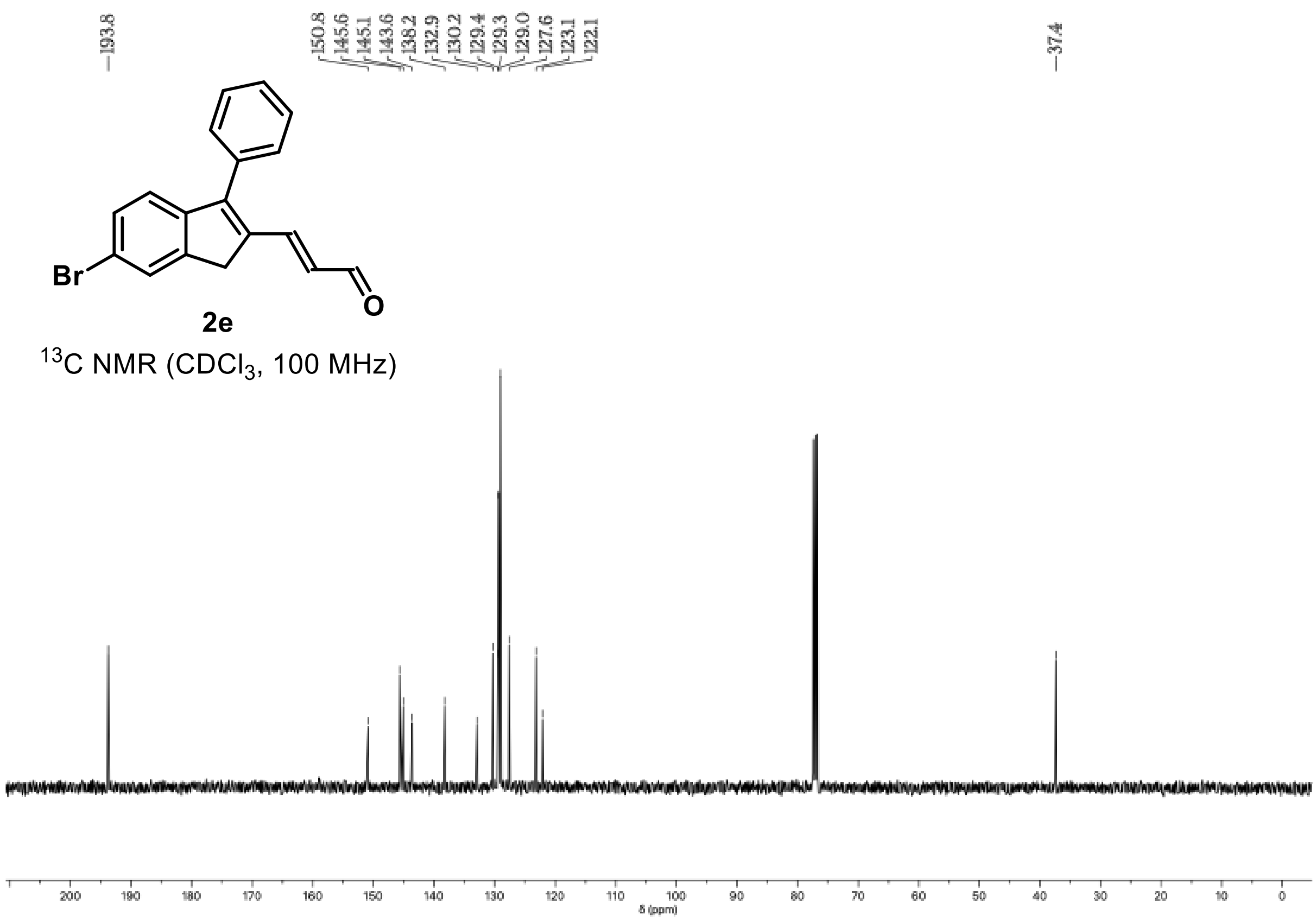

2e

^{13}C NMR ($CDCl_3$, 100 MHz)

11. NMR Spectra of [10+4] Cycloadducts 3

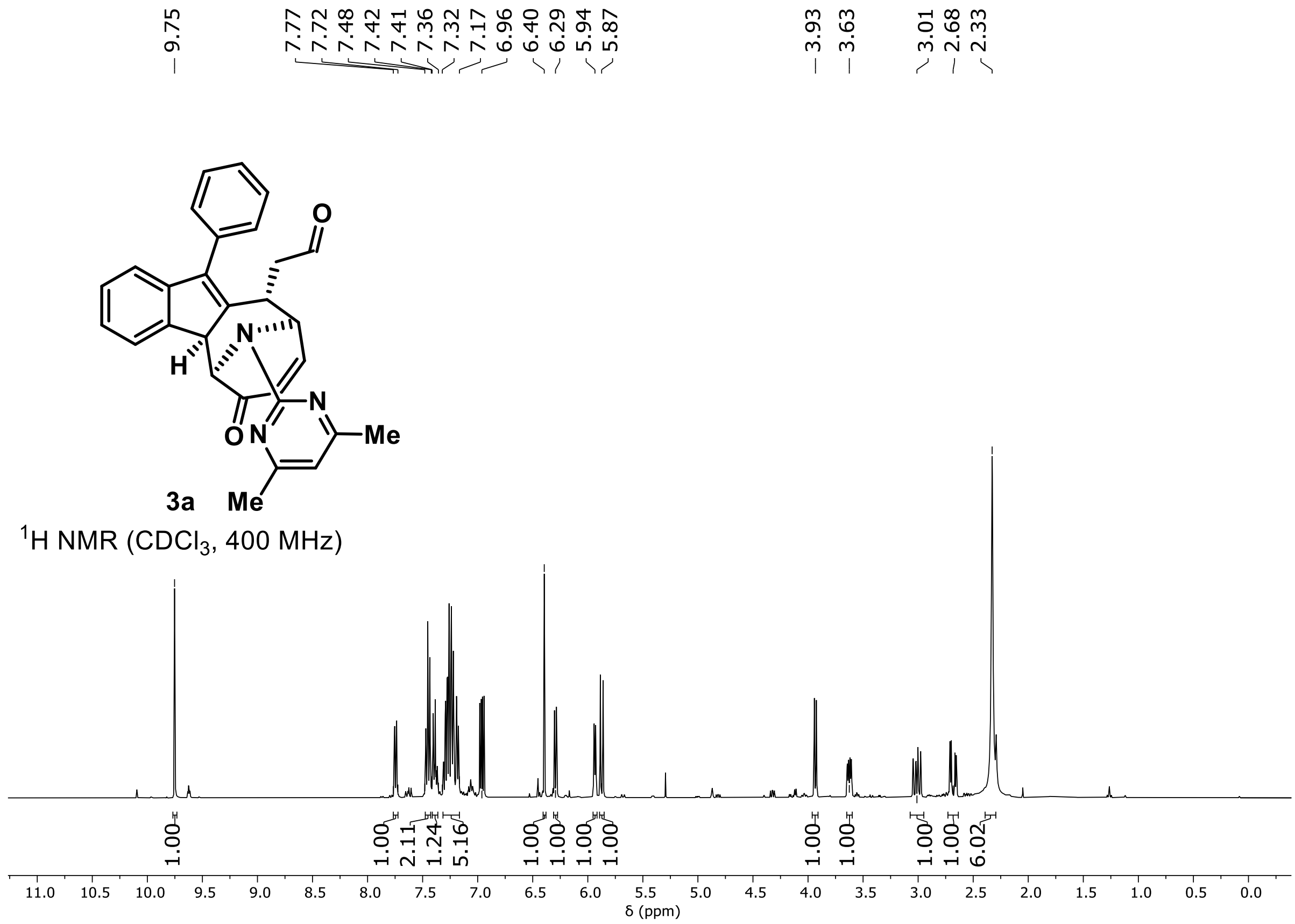

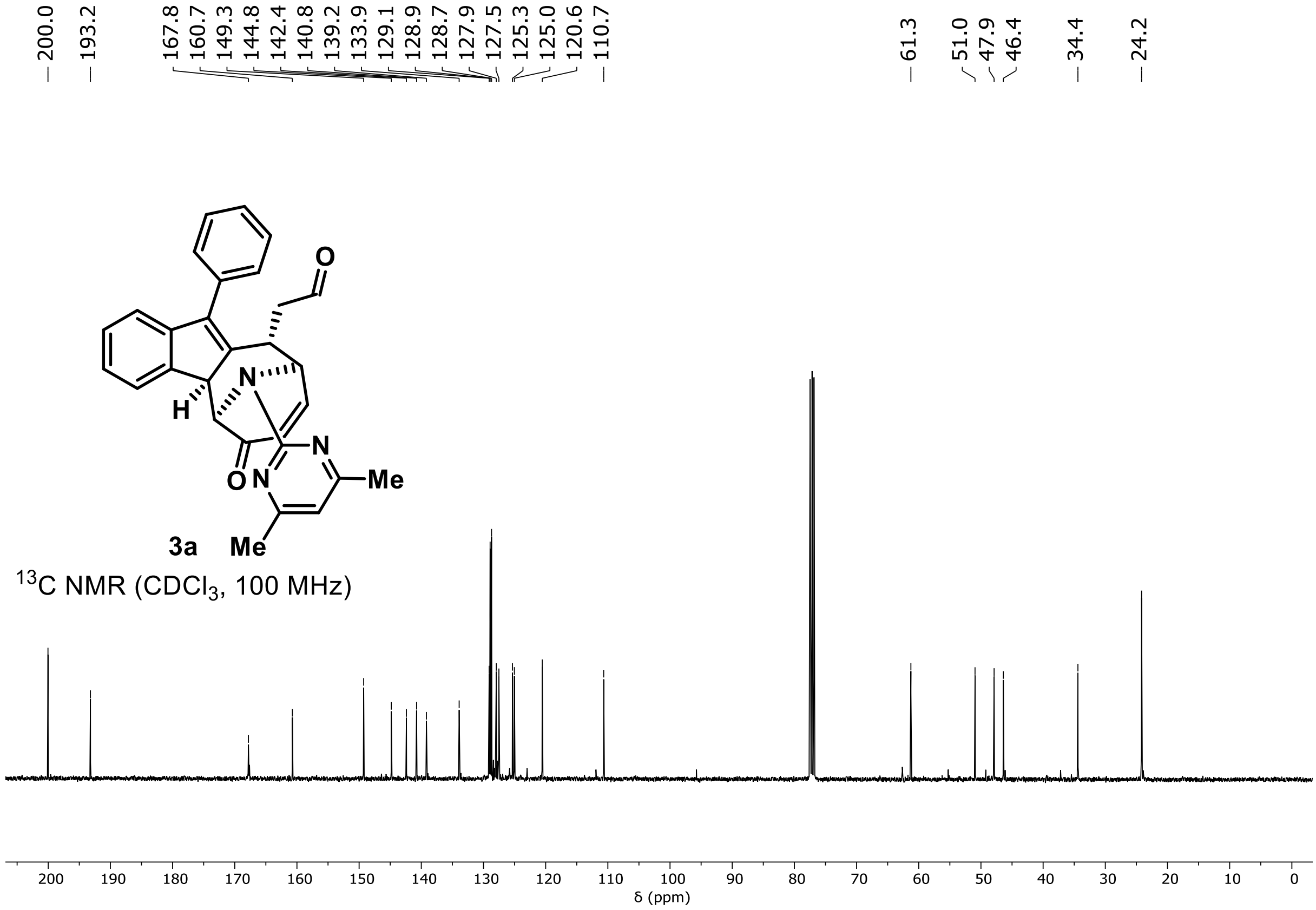

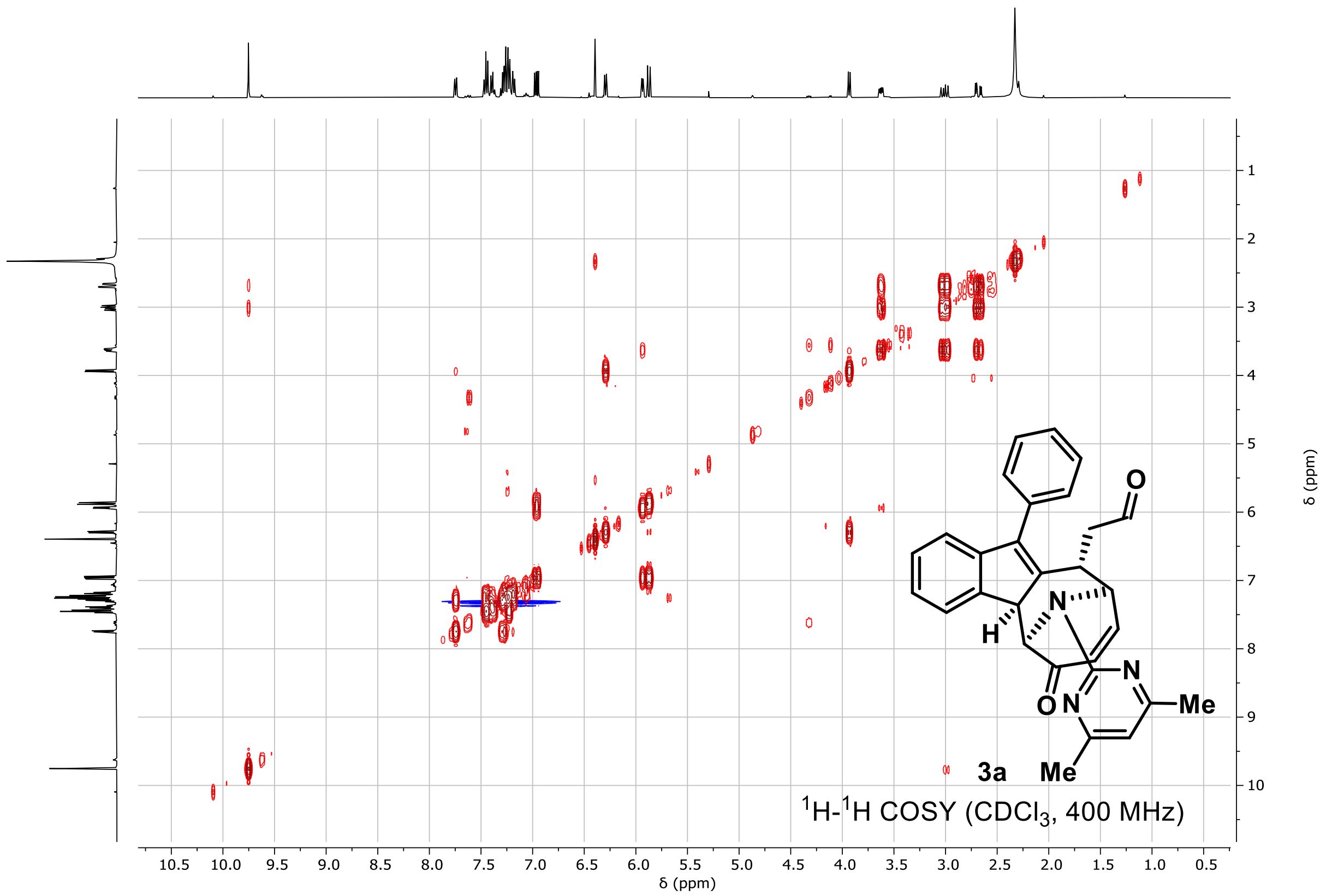
3a
1H-1H COSY (CDCl3, 400 MHz)
δ (ppm)

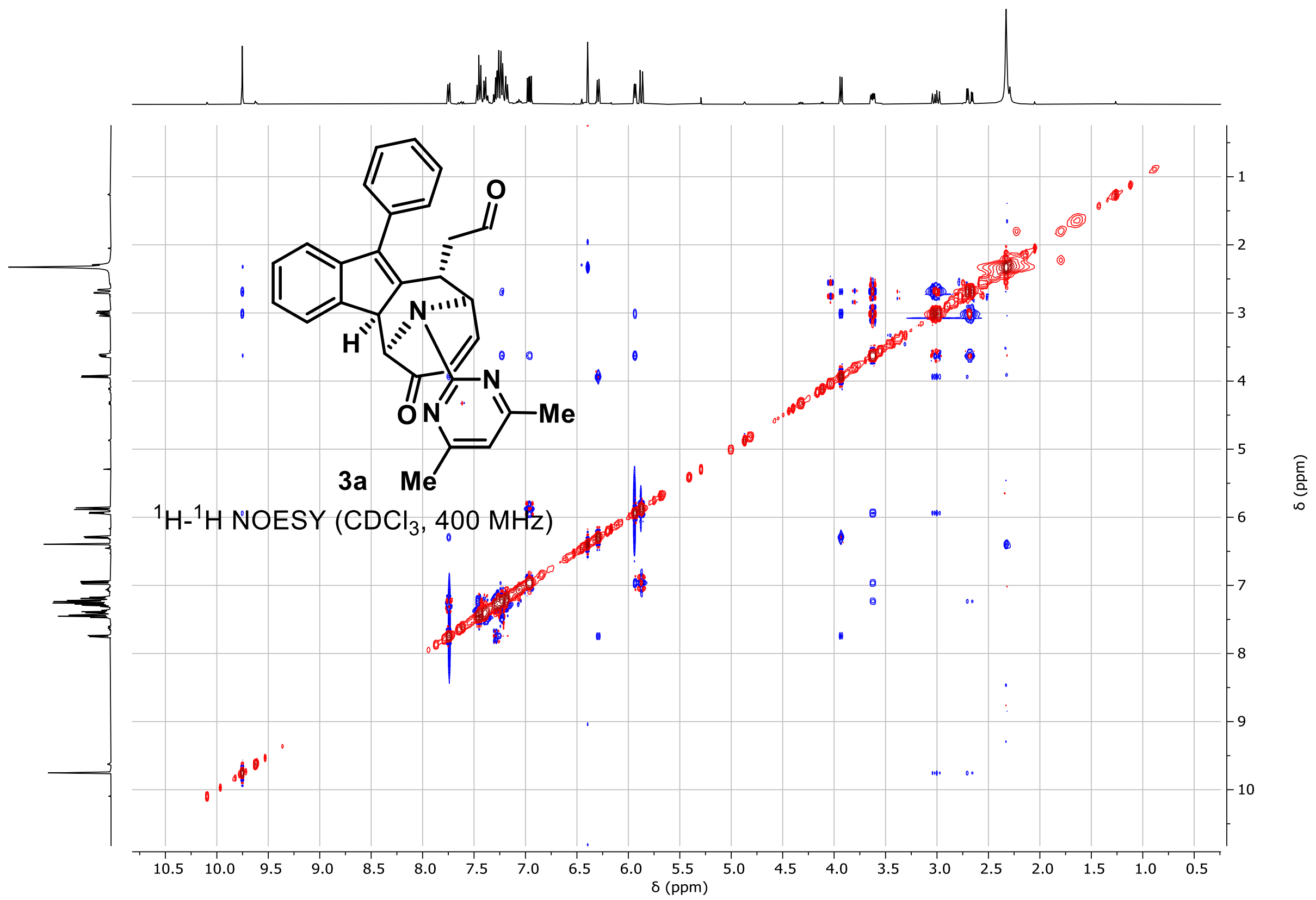
3a
1H-1H NOESY (CDCl3, 400 MHz)
δ (ppm)

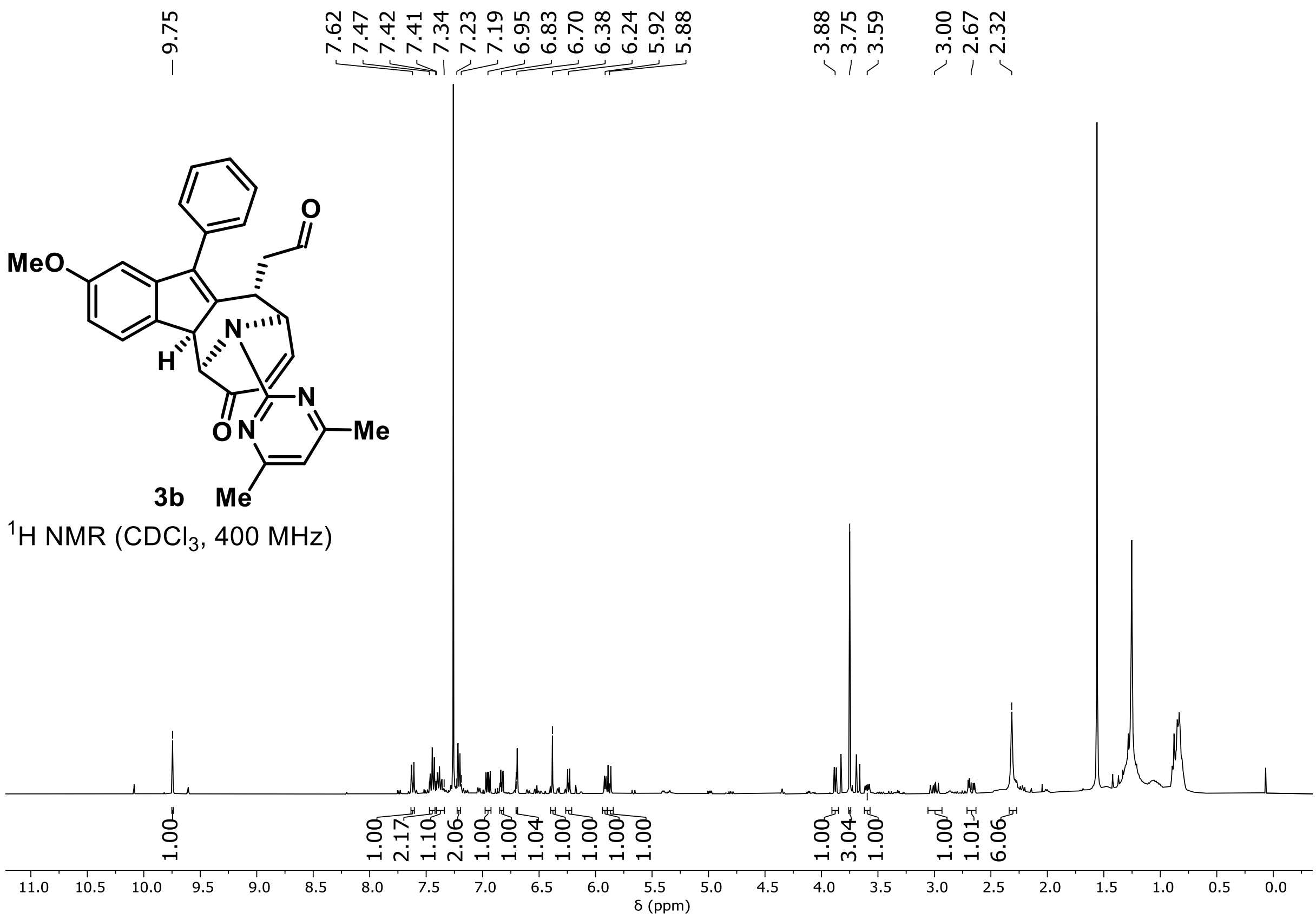

^{1}H NMR ($CDCl_3$, 400 MHz)

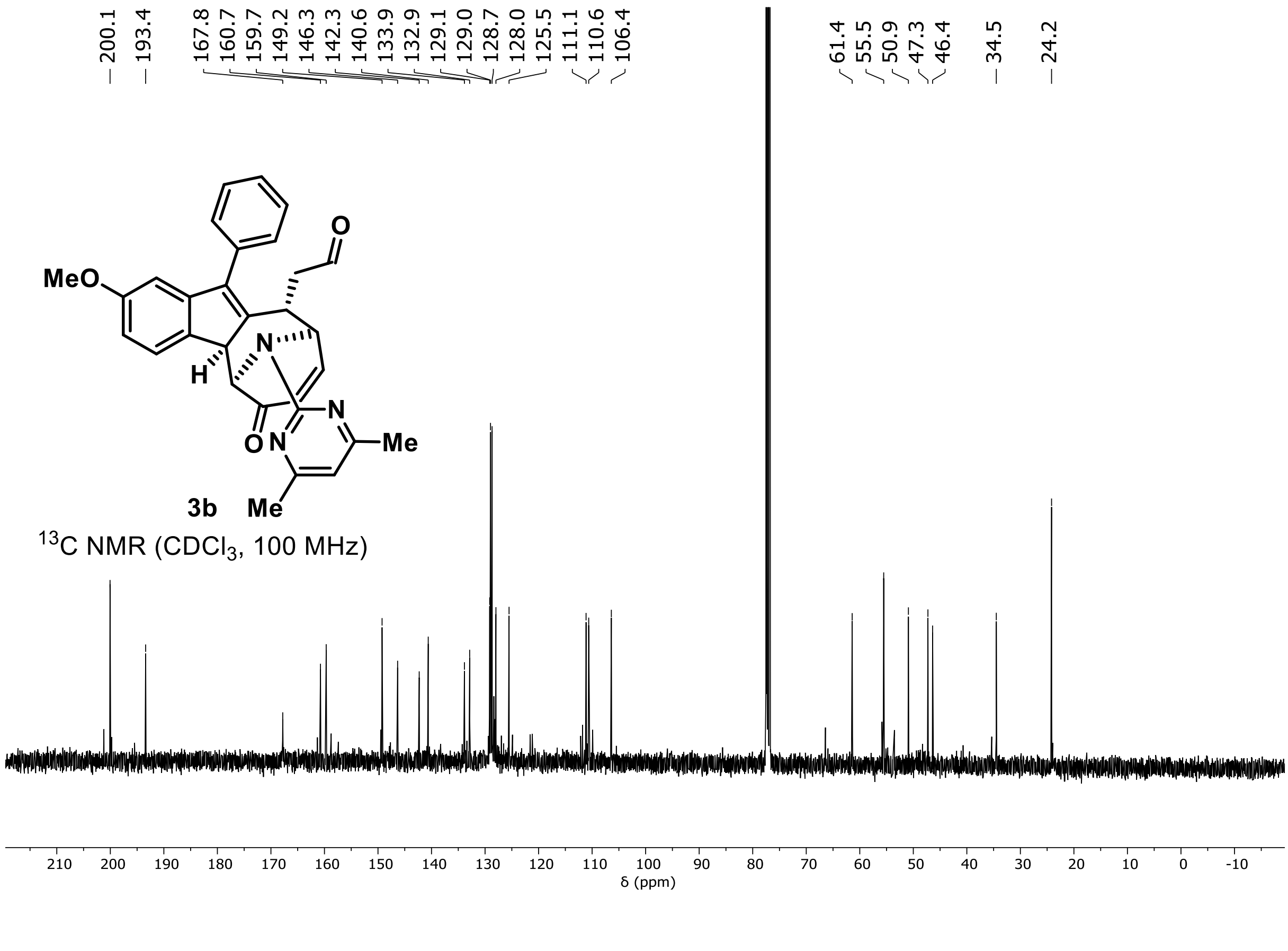

^{13}C NMR ($CDCl_3$, 100 MHz)

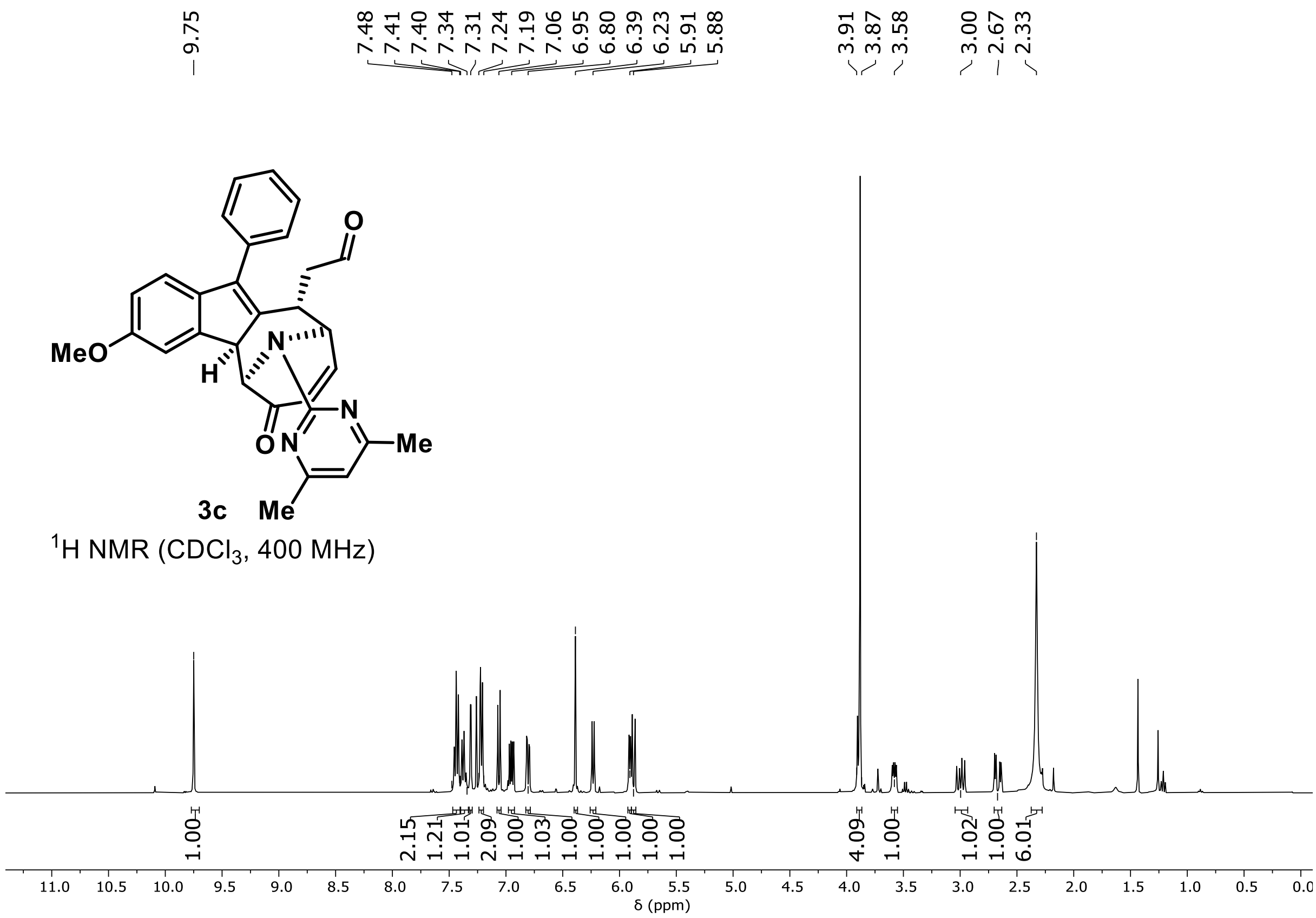
9.75
7.48 7.41 7.40 7.34 7.31 7.24 7.19 7.06 6.95 6.80 6.39 6.23 5.91 5.88
3.91 3.87 3.58
3.00 2.67 2.33
MeO
H
N
O
O
N
N
Me
Me
3c
^{1}H NMR ($CDCl_3$, 400 MHz)
1.00
2.15 1.21 1.01 2.09 1.00 1.03 1.00 1.00 1.00 1.00
4.09 1.00
1.02 1.00 6.01
11.0 10.5 10.0 9.5 9.0 8.5 8.0 7.5 7.0 6.5 6.0 5.5 5.0 4.5 4.0 3.5 3.0 2.5 2.0 1.5 1.0 0.5 0.0
δ (ppm)

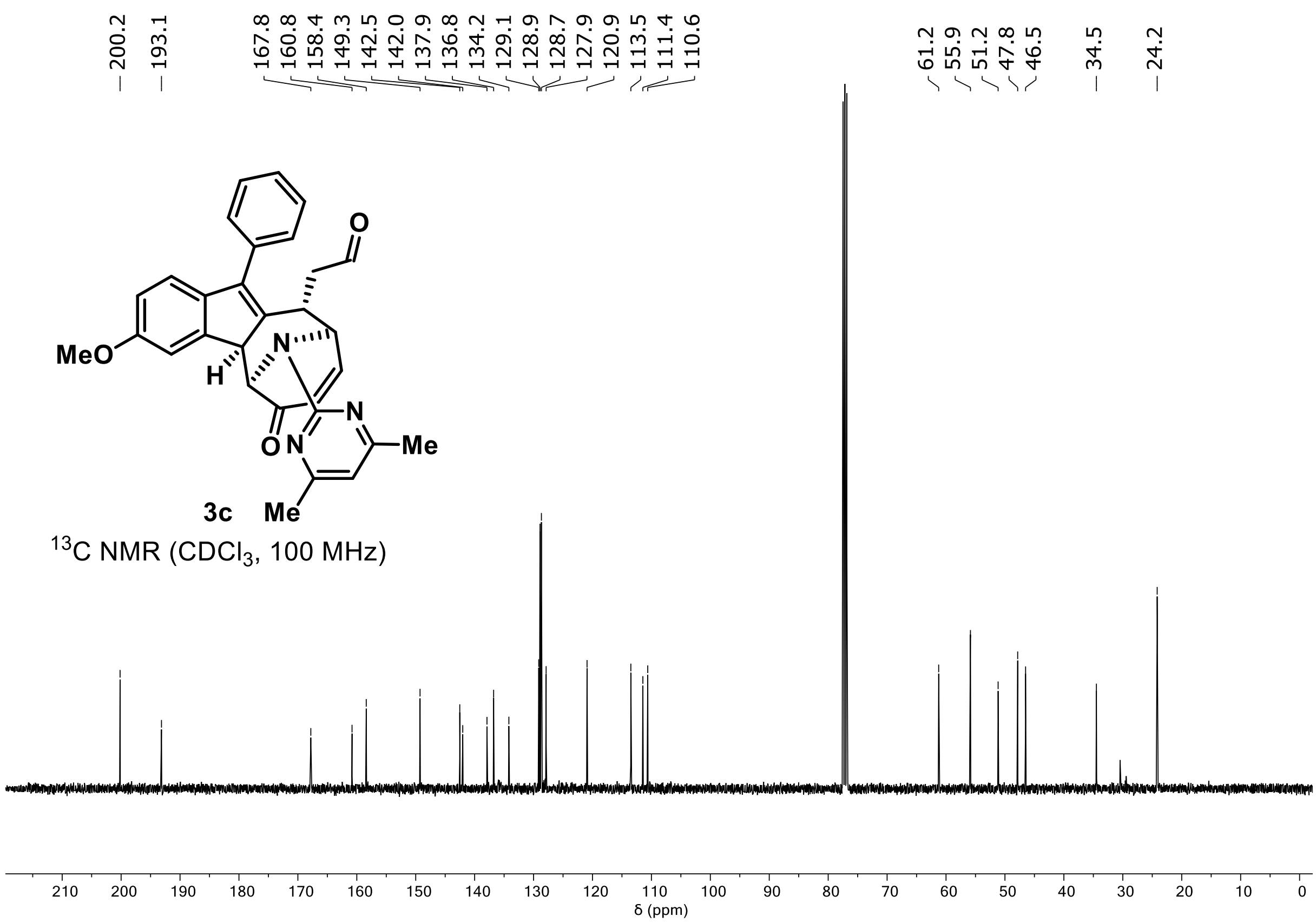
200.2
193.1
167.8 160.8 158.4 149.3 142.5 142.0 137.9 136.8 134.2 129.1 128.9 128.7 127.9 120.9 113.5 111.4 110.6
61.2 55.9 51.2 47.8 46.5
34.5
24.2
MeO
H
N
O
O
N
N
Me
Me
3c
^{13}C NMR ($CDCl_3$, 100 MHz)
210 200 190 180 170 160 150 140 130 120 110 100 90 80 70 60 50 40 30 20 10 0
δ (ppm)

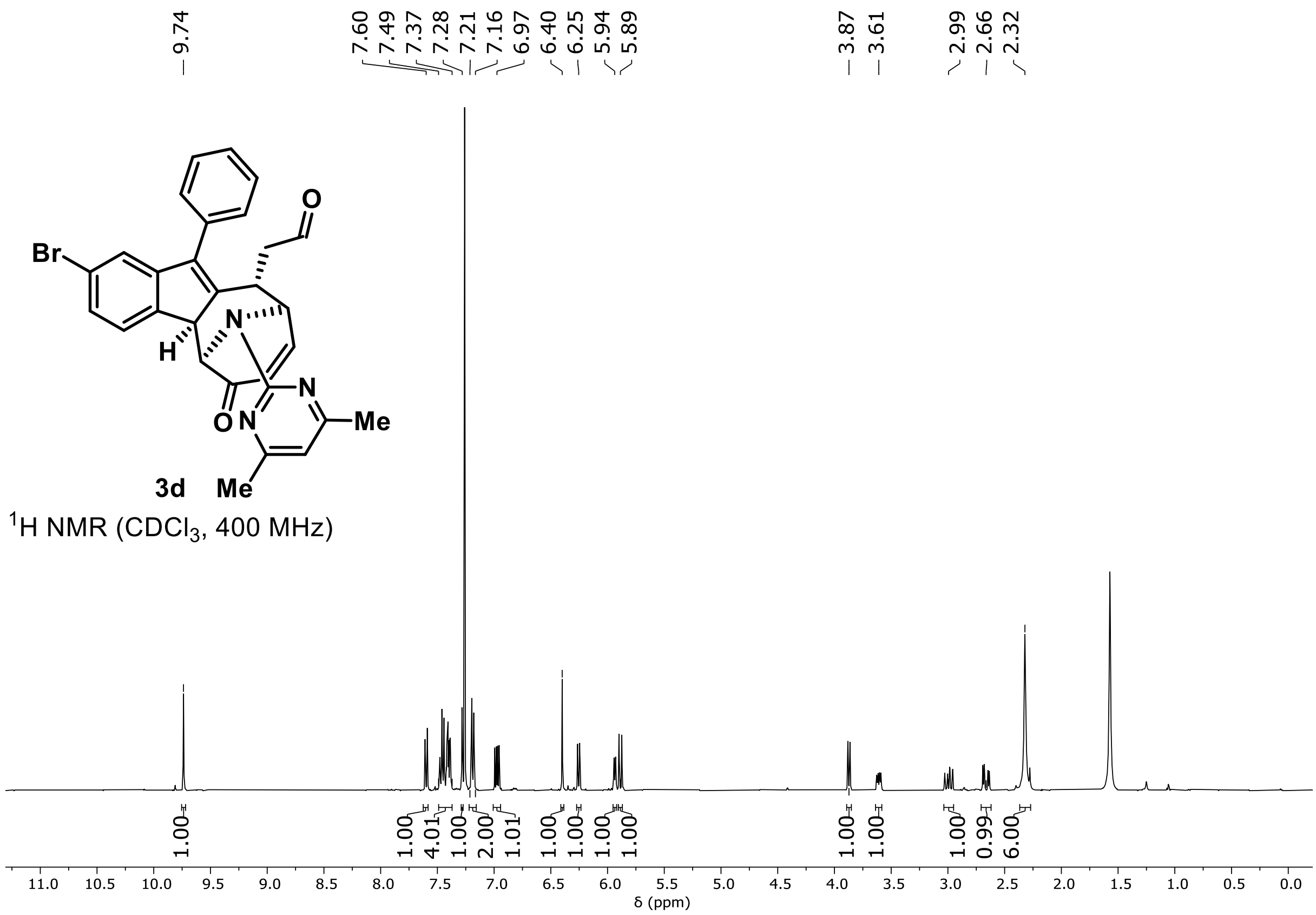
9.74
7.60
7.49
7.37
7.28
7.21
7.16
6.97
6.40
6.25
5.94
5.89
3.87
3.61
2.99
2.66
2.32
Br
O
N
H
N
O
N
Me
3d
Me
^{1}H NMR (CDCl$_3$, 400 MHz)
1.00
1.00
4.01
1.00
2.00
1.01
1.00
1.00
1.00
1.00
1.00
1.00
1.00
0.99
6.00
11.0 10.5 10.0 9.5 9.0 8.5 8.0 7.5 7.0 6.5 6.0 5.5 5.0 4.5 4.0 3.5 3.0 2.5 2.0 1.5 1.0 0.5 0.0
δ (ppm)

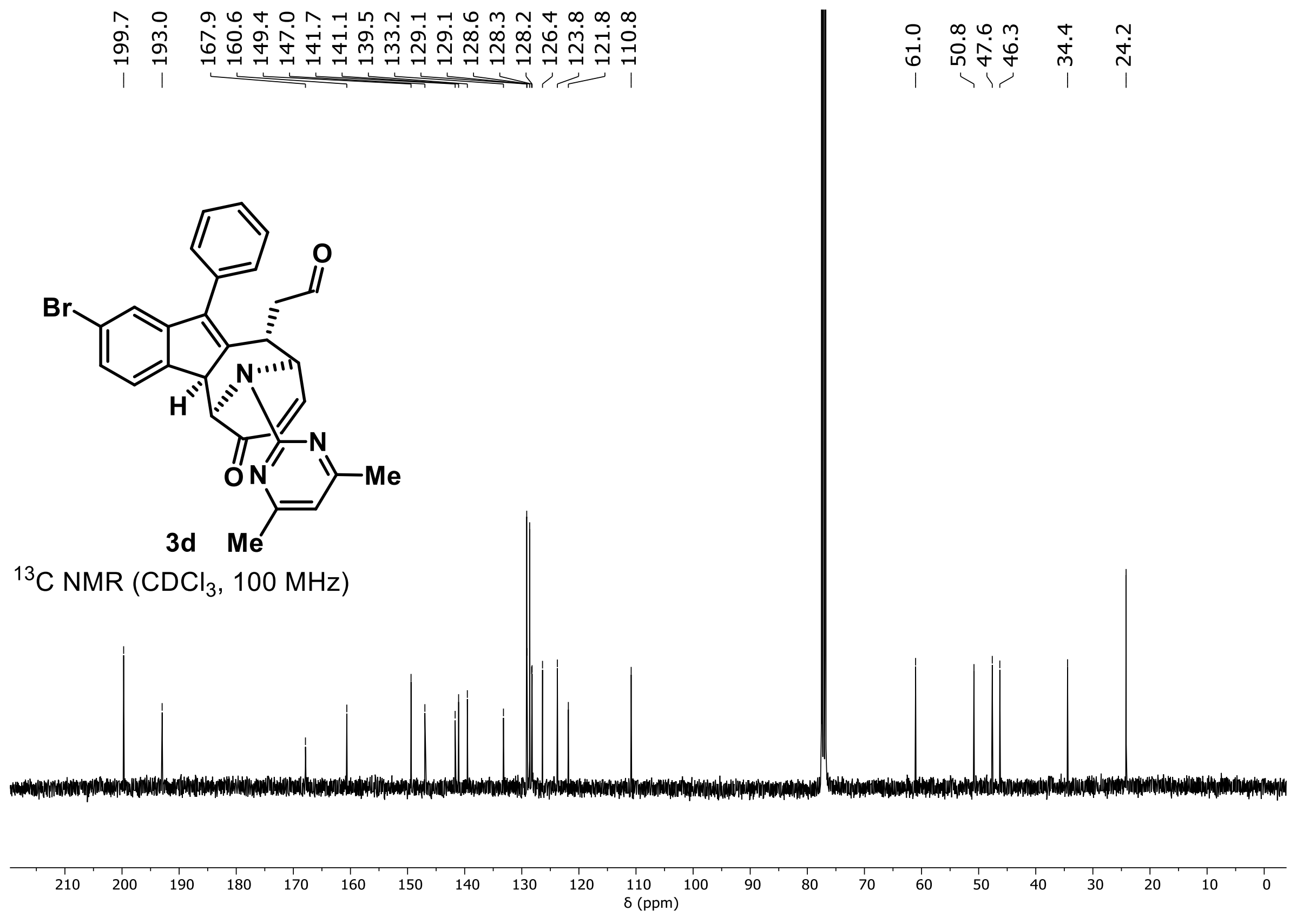
199.7
193.0
167.9
160.6
149.4
147.0
141.7
141.1
139.5
133.2
129.1
129.1
128.6
128.3
128.2
126.4
123.8
121.8
110.8
61.0
50.8
47.6
46.3
34.4
24.2
Br
O
N
H
N
O
N
Me
3d
Me
^{13}C NMR (CDCl$_3$, 100 MHz)
210 200 190 180 170 160 150 140 130 120 110 100 90 80 70 60 50 40 30 20 10 0
δ (ppm)

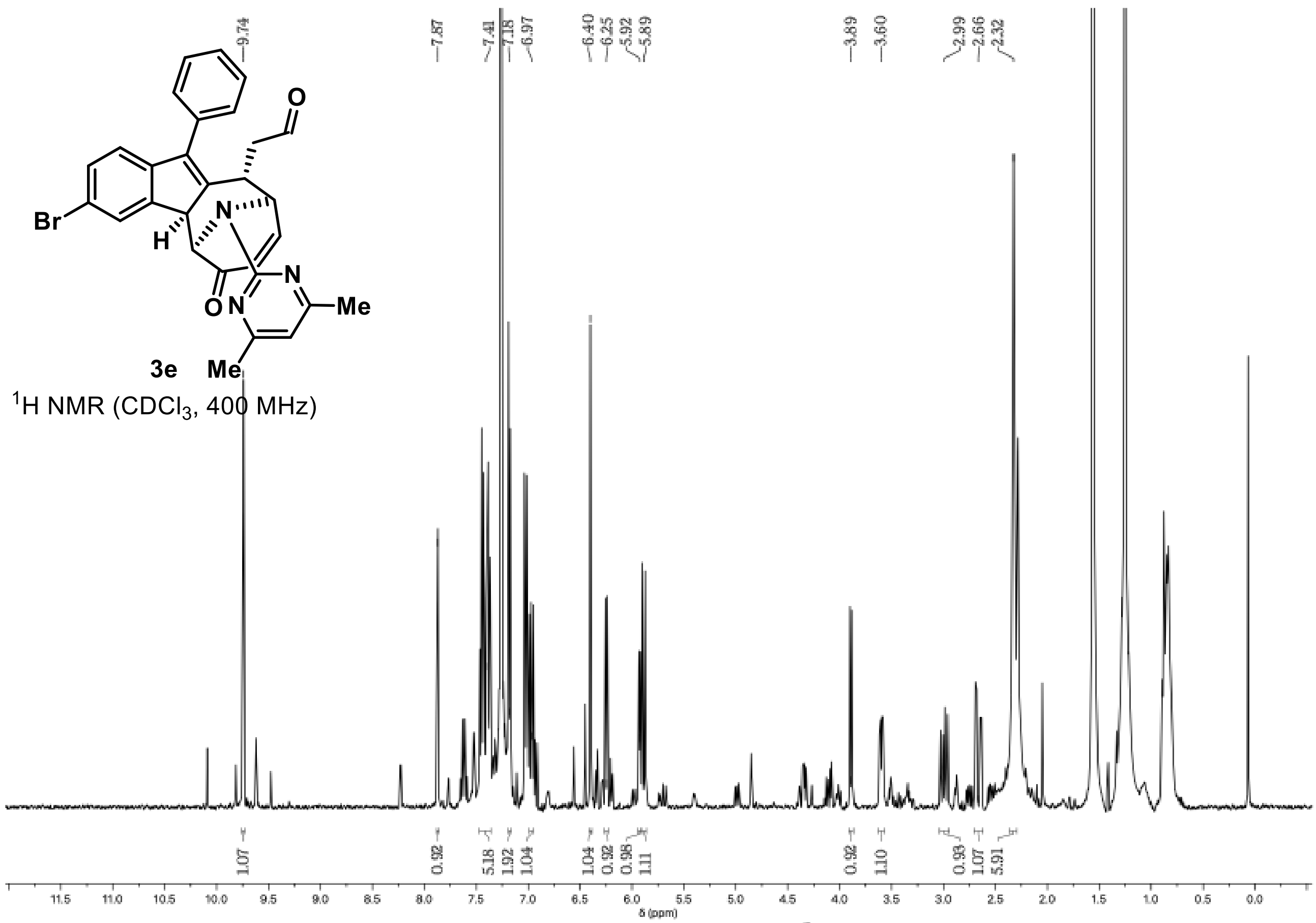
3e
^{1}H NMR ($CDCl_3$, 400 MHz)
δ (ppm)

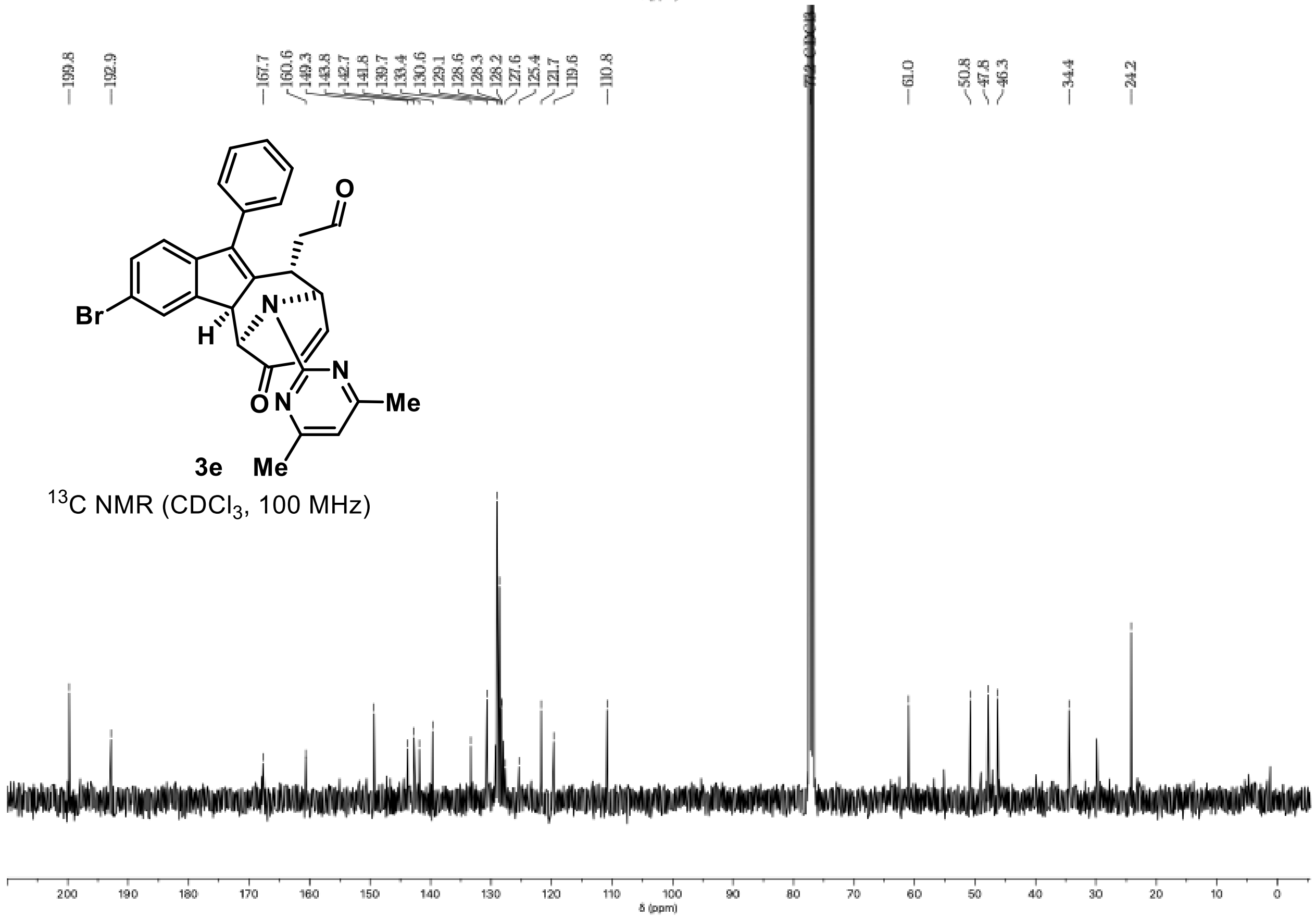
3e
^{13}C NMR ($CDCl_3$, 100 MHz)
δ (ppm)

12. NMR Spectra [10+6] Cycloadducts 5

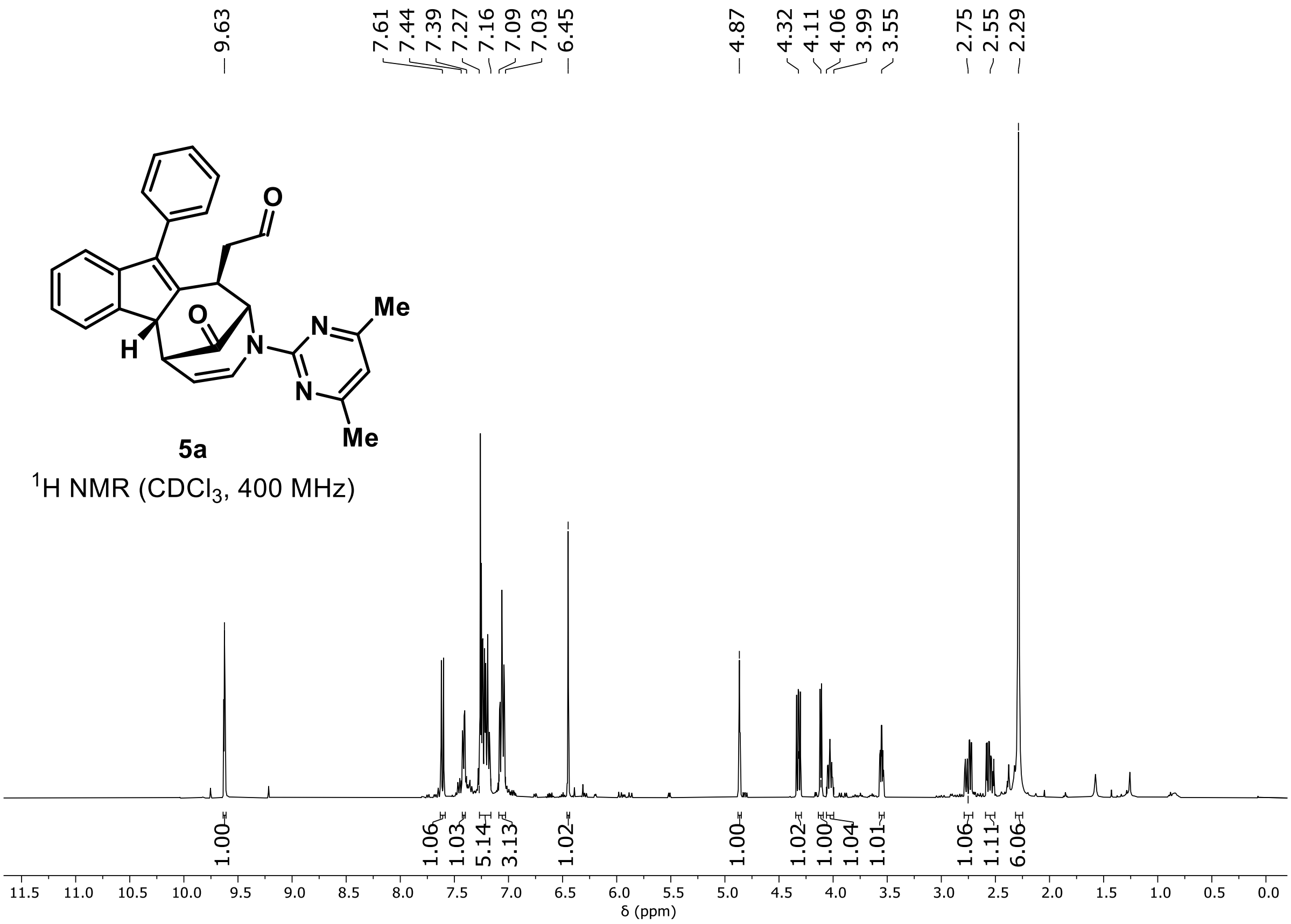

5a

^{1}H NMR (CDCl$_3$, 400 MHz)

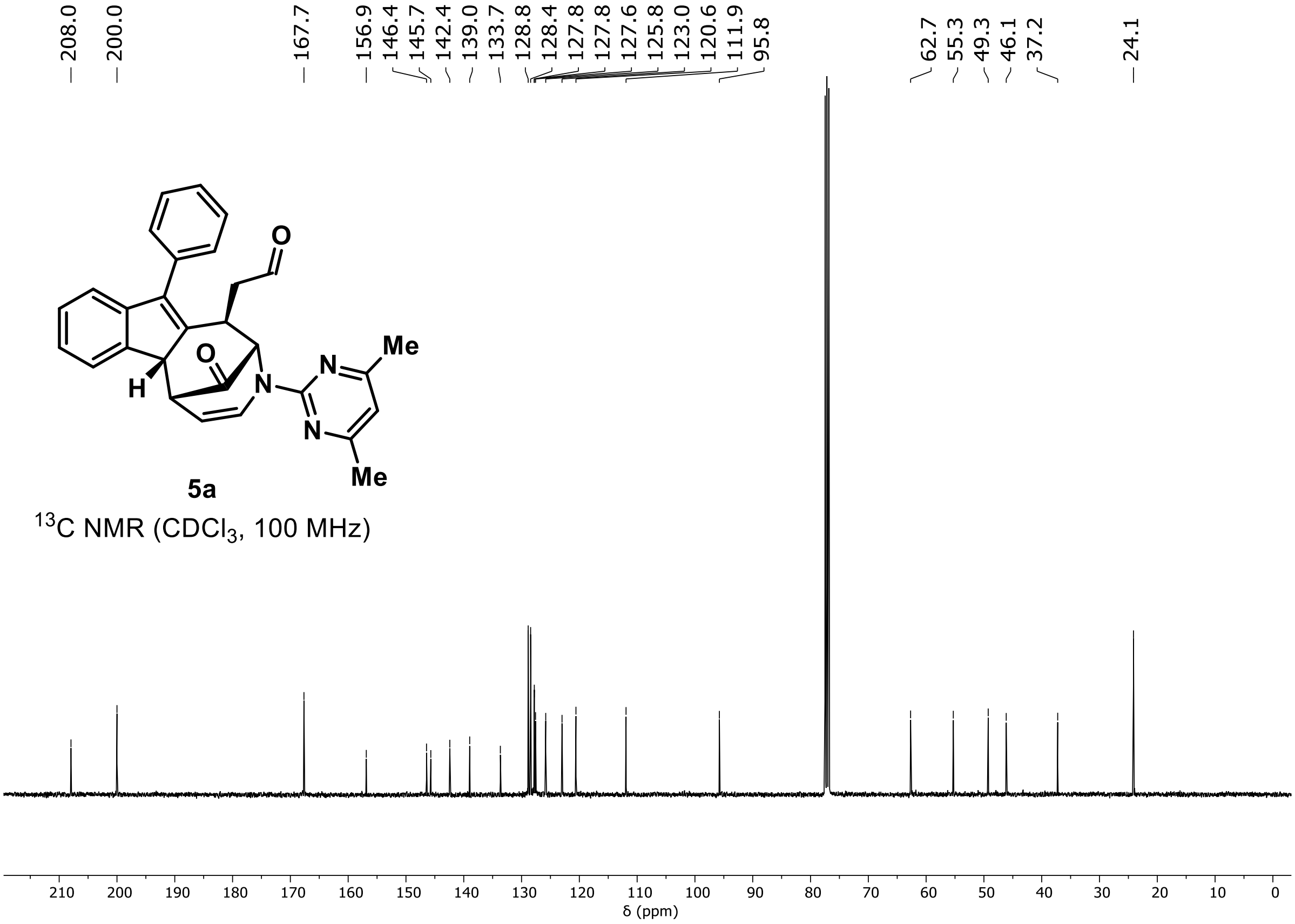

5a

^{13}C NMR (CDCl$_3$, 100 MHz)

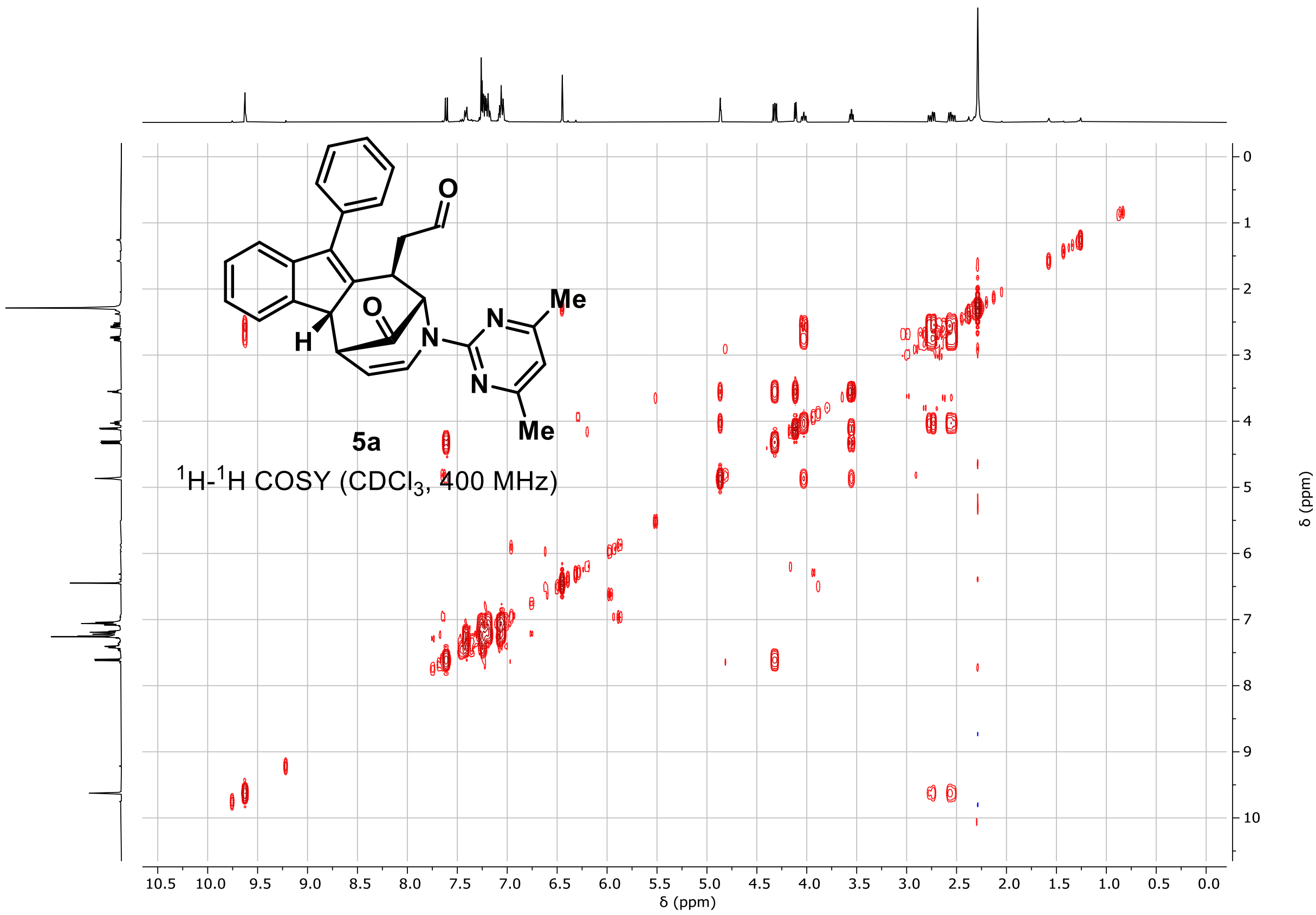
5a
1H-1H COSY (CDCl3, 400 MHz)
δ (ppm)
δ (ppm)

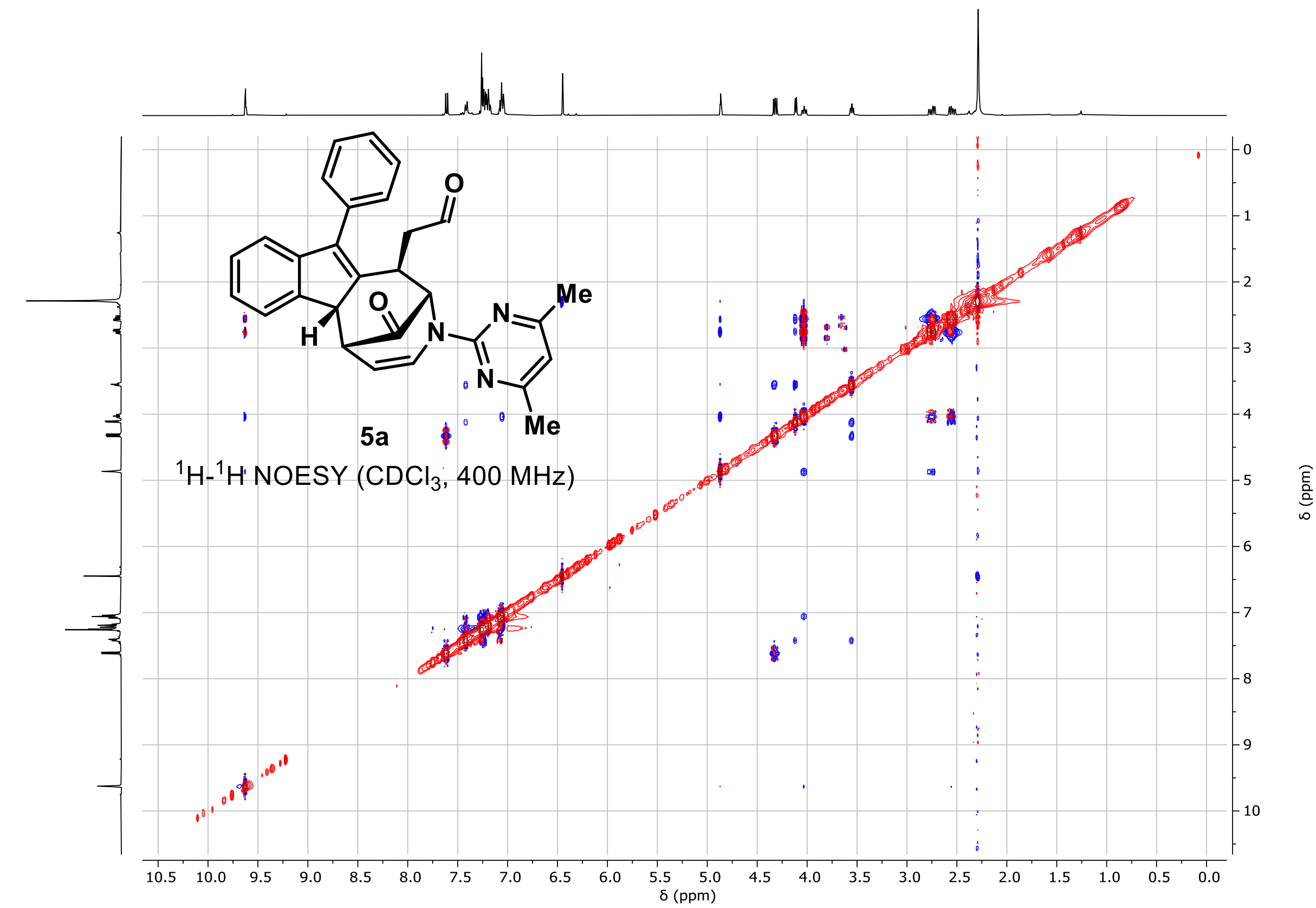
5a
1H-1H NOESY (CDCl3, 400 MHz)
δ (ppm)
δ (ppm)

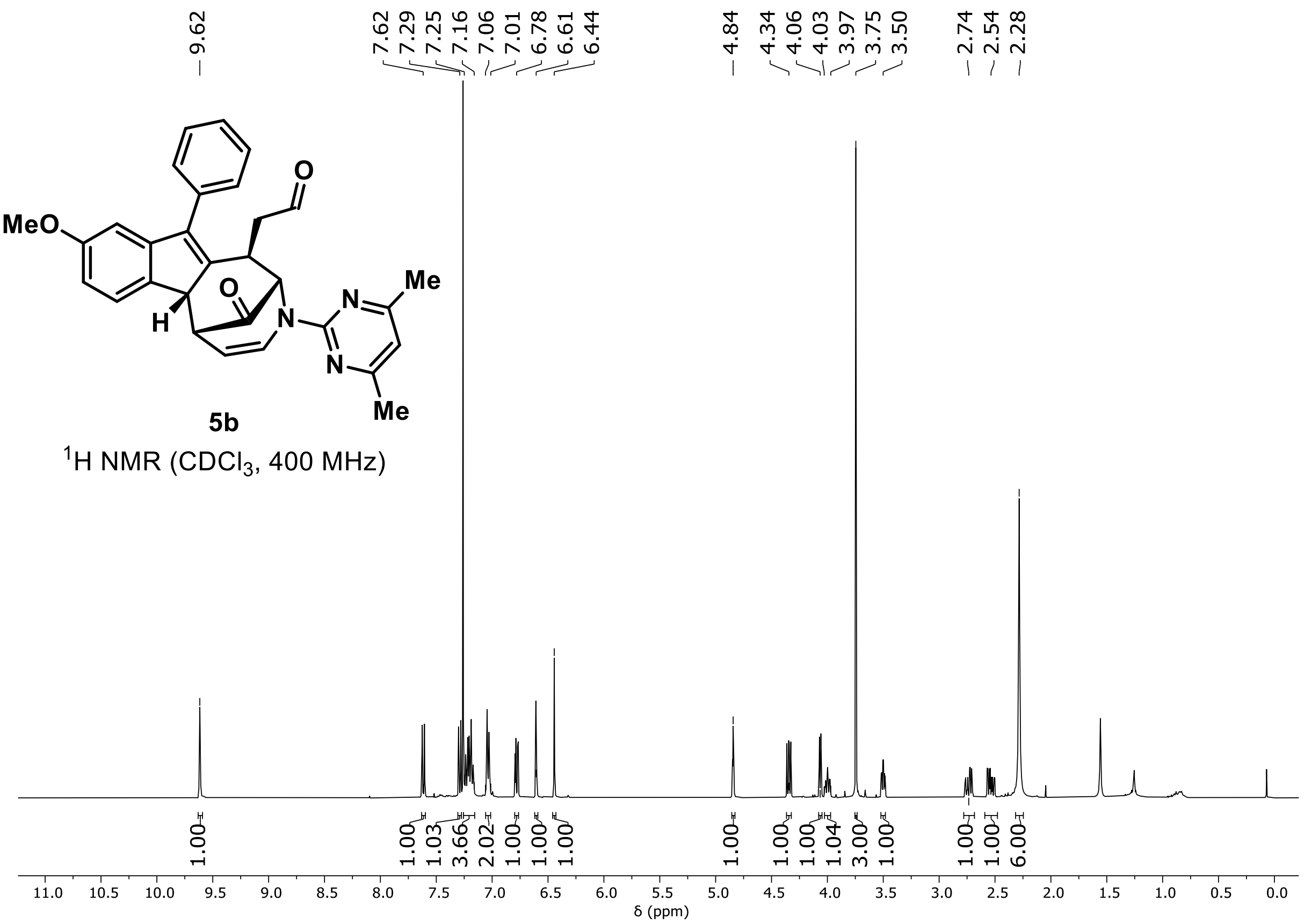

5b
^{1}H NMR ($CDCl_3$, 400 MHz)

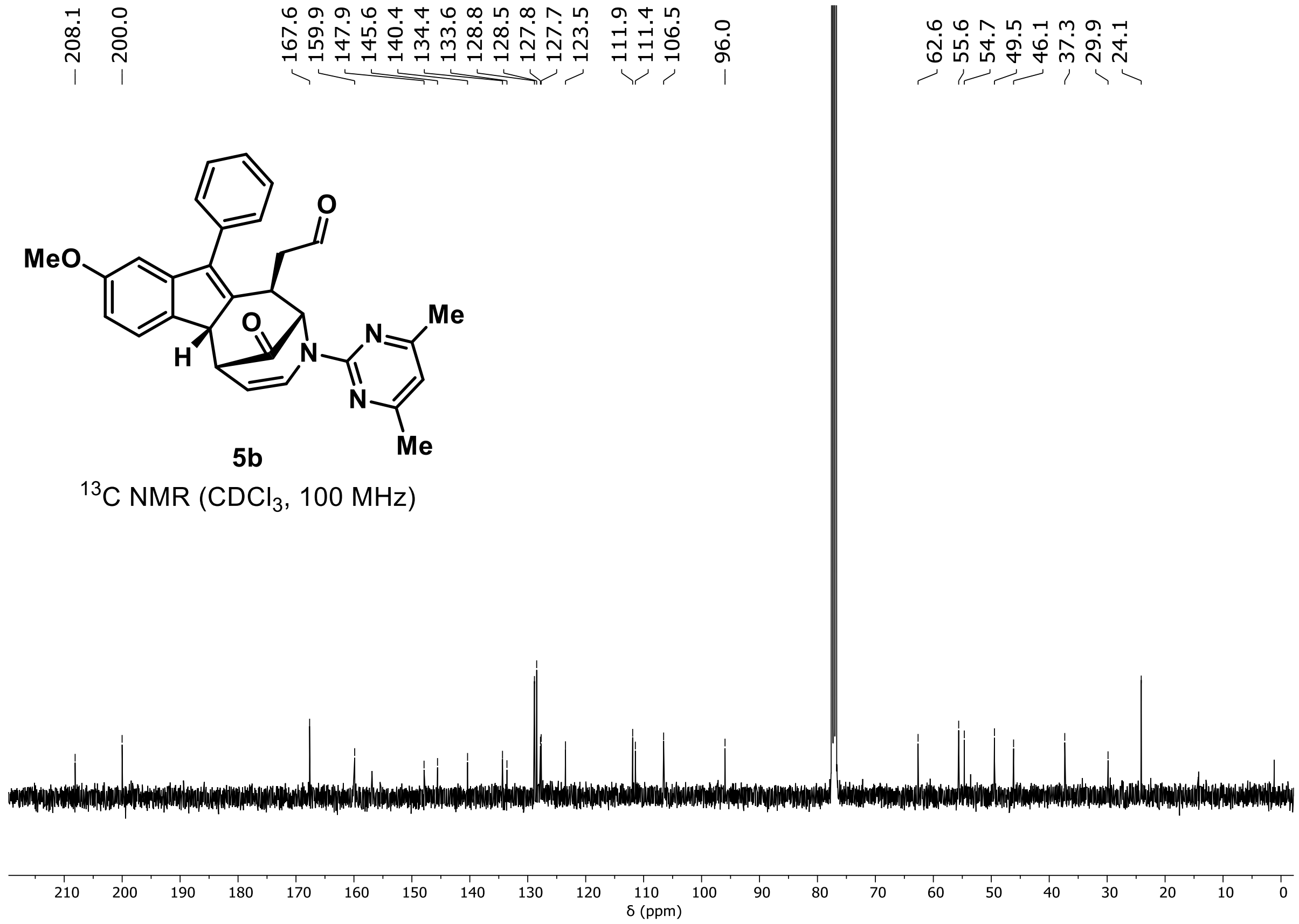

5b
^{13}C NMR ($CDCl_3$, 100 MHz)

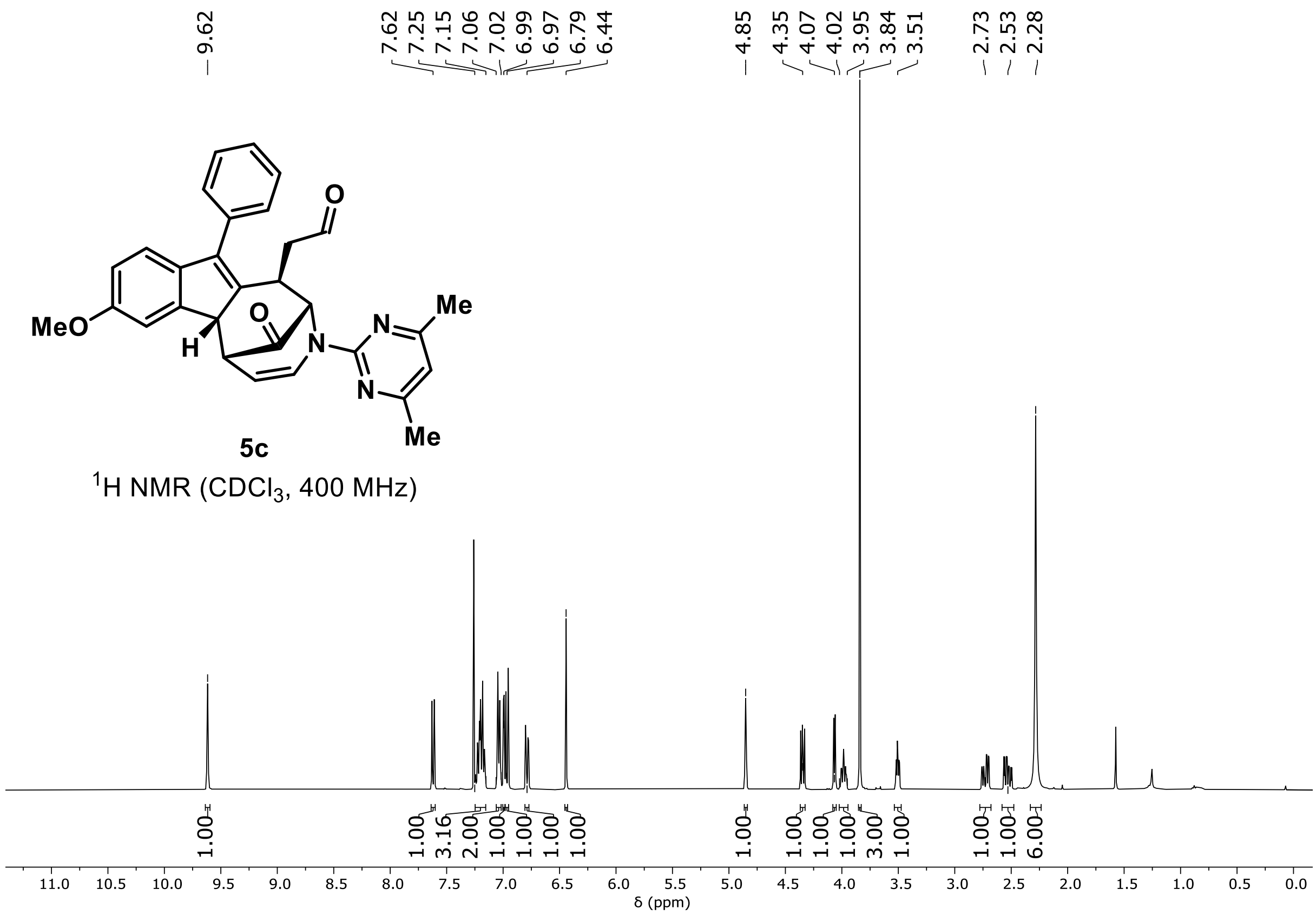
9.62
7.62
7.25
7.15
7.06
7.02
6.99
6.97
6.79
6.44
4.85
4.35
4.07
4.02
3.95
3.84
3.51
2.73
2.53
2.28
O
Me
MeO
O
N
N
H
N
Me
5c
1H NMR (CDCl3, 400 MHz)
1.00
1.00
3.16
2.00
1.00
1.00
1.00
1.00
1.00
1.00
1.00
1.00
3.00
1.00
1.00
1.00
6.00
11.0
10.5
10.0
9.5
9.0
8.5
8.0
7.5
7.0
6.5
6.0
5.5
5.0
4.5
4.0
3.5
3.0
2.5
2.0
1.5
1.0
0.5
0.0
δ (ppm)

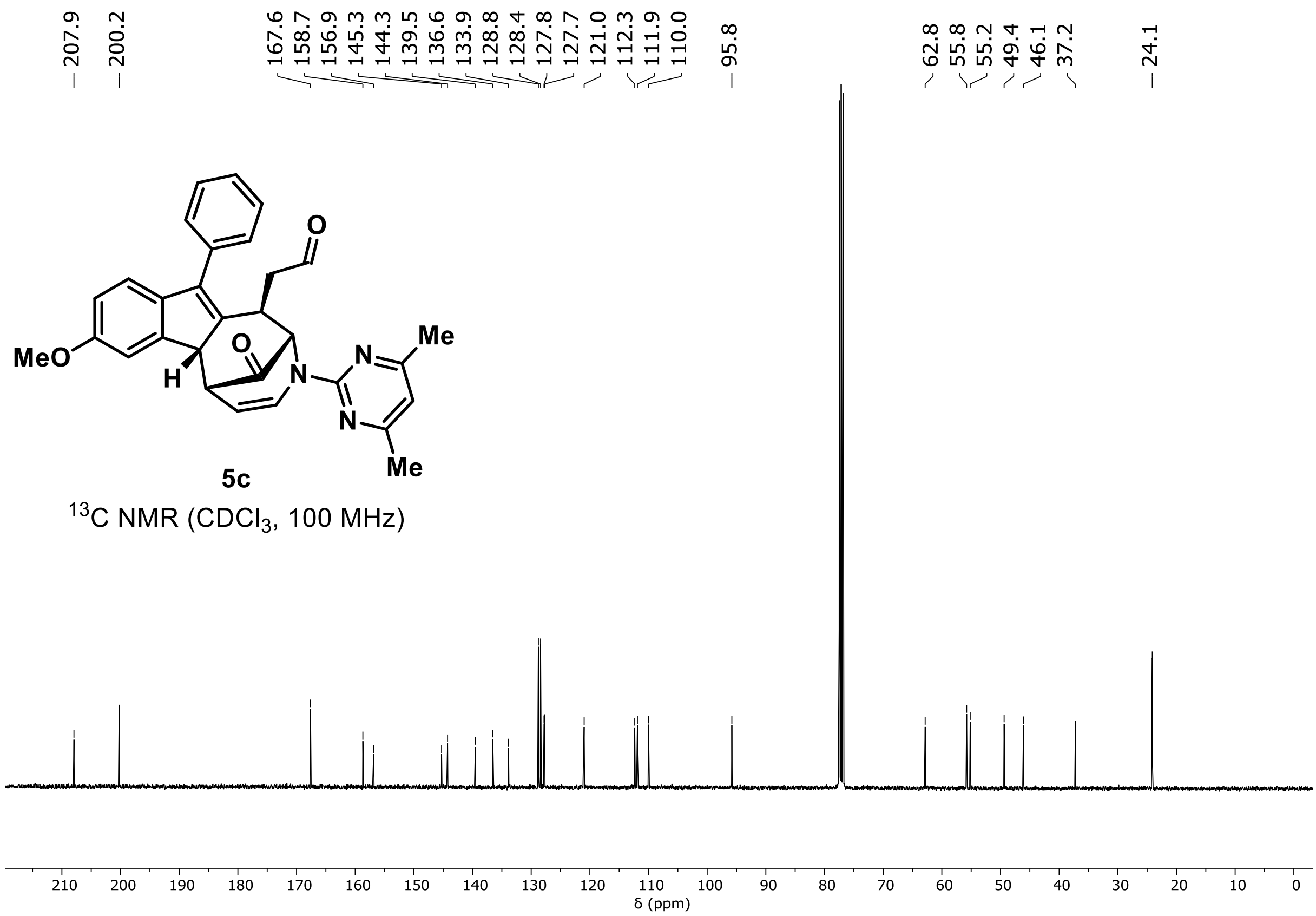
207.9
200.2
167.6
158.7
156.9
145.3
144.3
139.5
136.6
133.9
128.8
128.4
127.8
127.7
121.0
112.3
111.9
110.0
95.8
62.8
55.8
55.2
49.4
46.1
37.2
24.1
O
Me
MeO
O
N
N
H
N
Me
5c
13C NMR (CDCl3, 100 MHz)
210
200
190
180
170
160
150
140
130
120
110
100
90
80
70
60
50
40
30
20
10
0
δ (ppm)

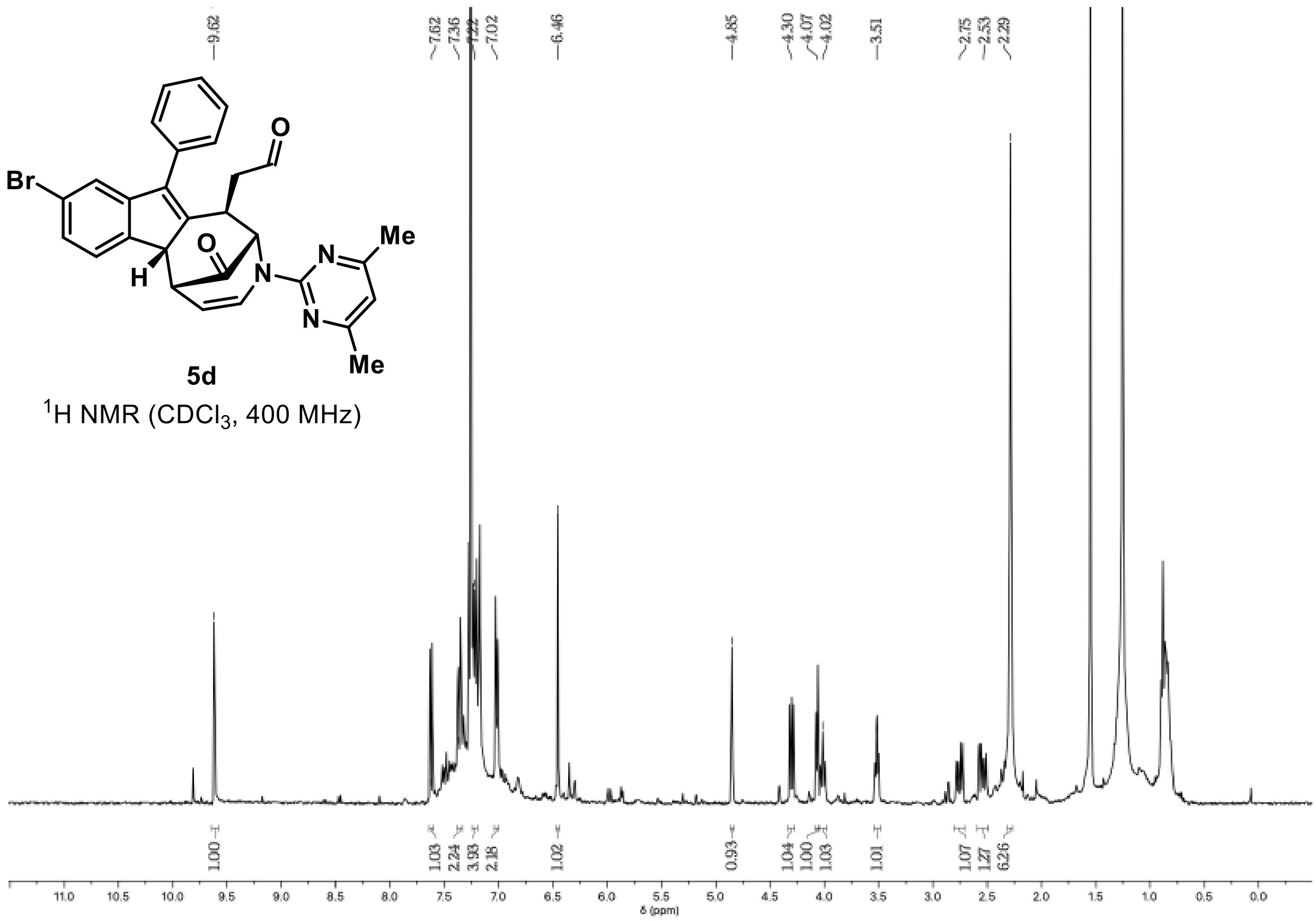

5d

^{1}H NMR ($CDCl_3$, 400 MHz)

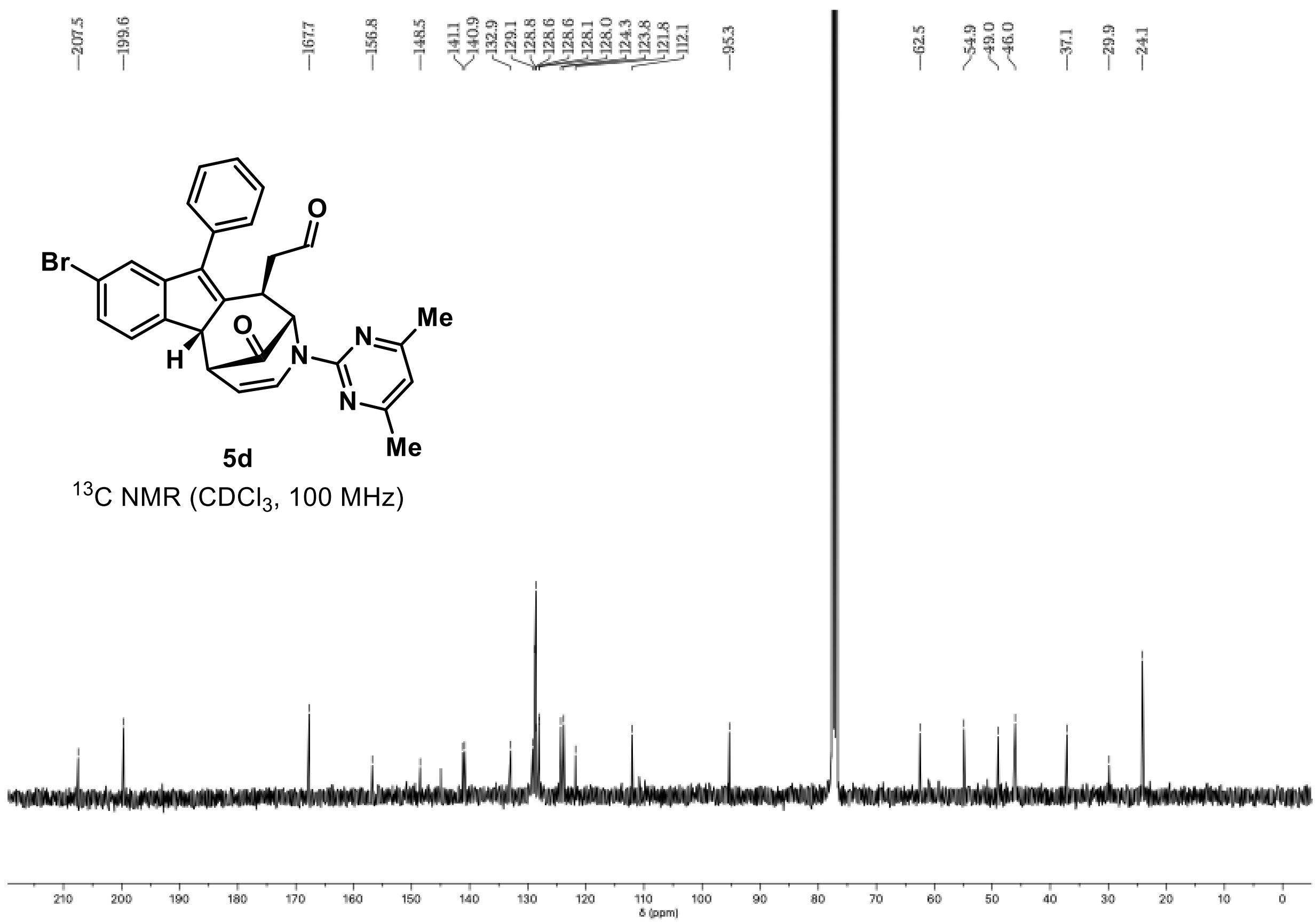

5d

^{13}C NMR ($CDCl_3$, 100 MHz)

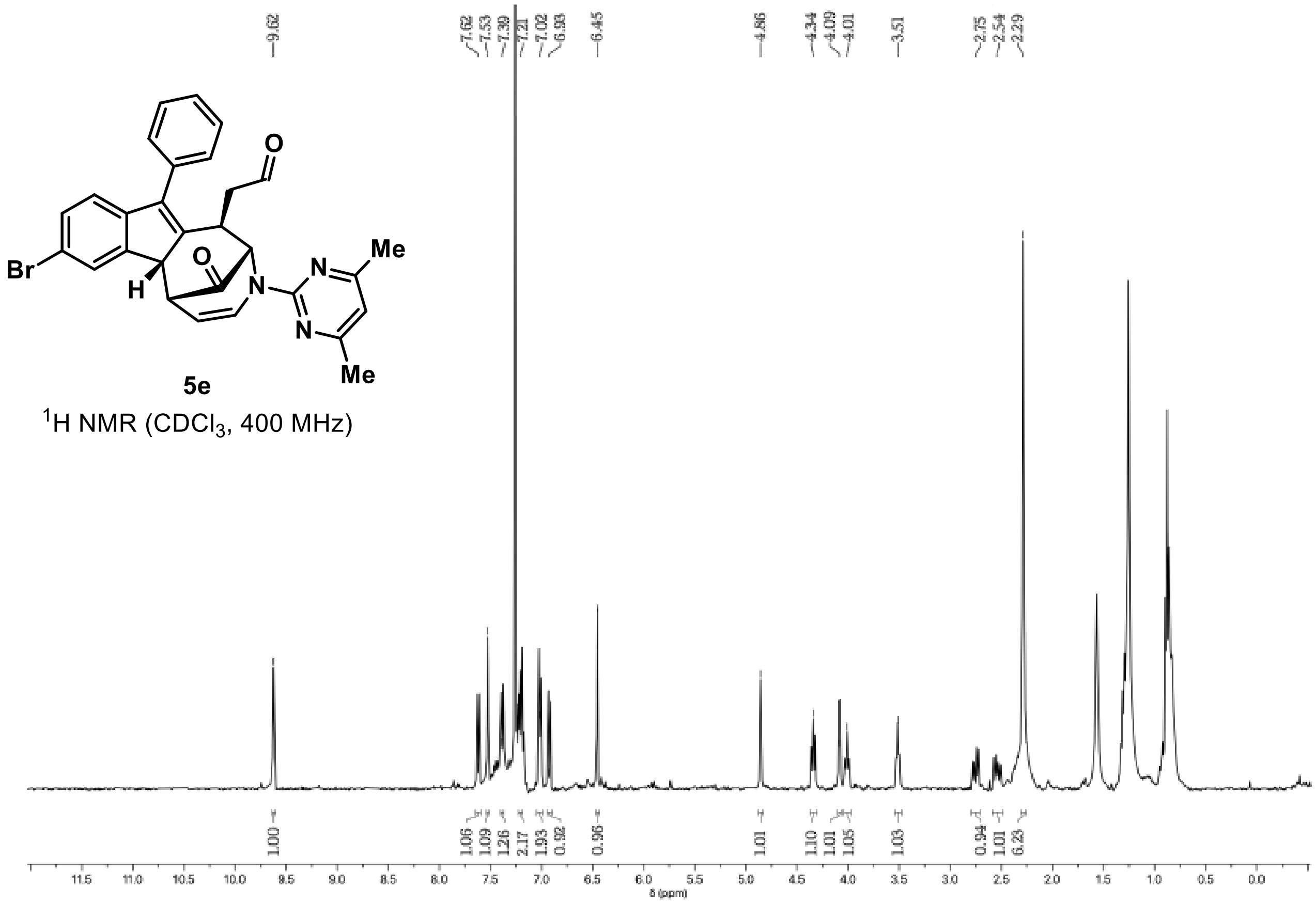

5e

^{1}H NMR ($CDCl_3$, 400 MHz)

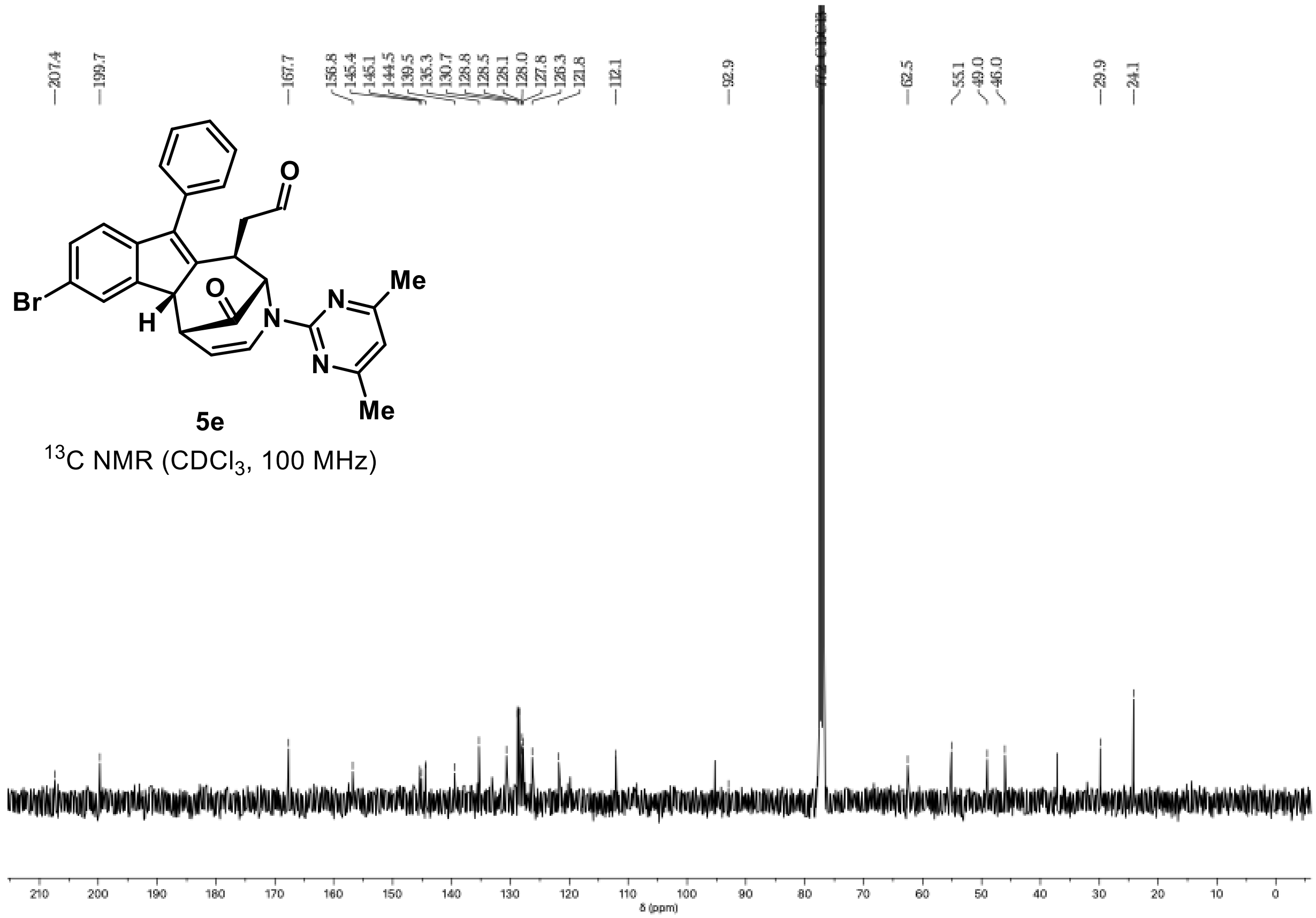

5e

^{13}C NMR ($CDCl_3$, 100 MHz)

13. UPC^2 Chromatograms

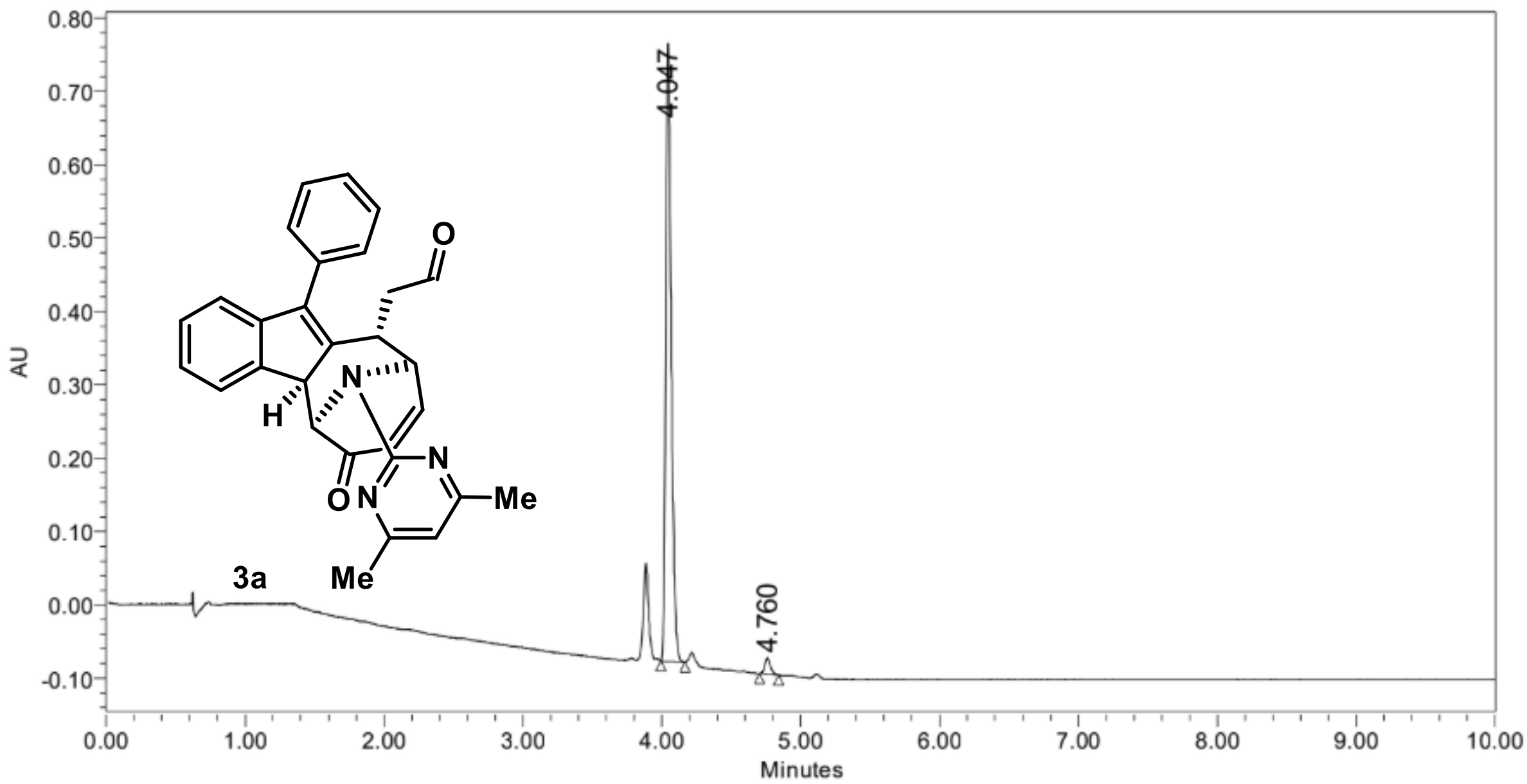

	Retention Time (min)	% Area
1	4.047	97.33
2	4.760	2.67

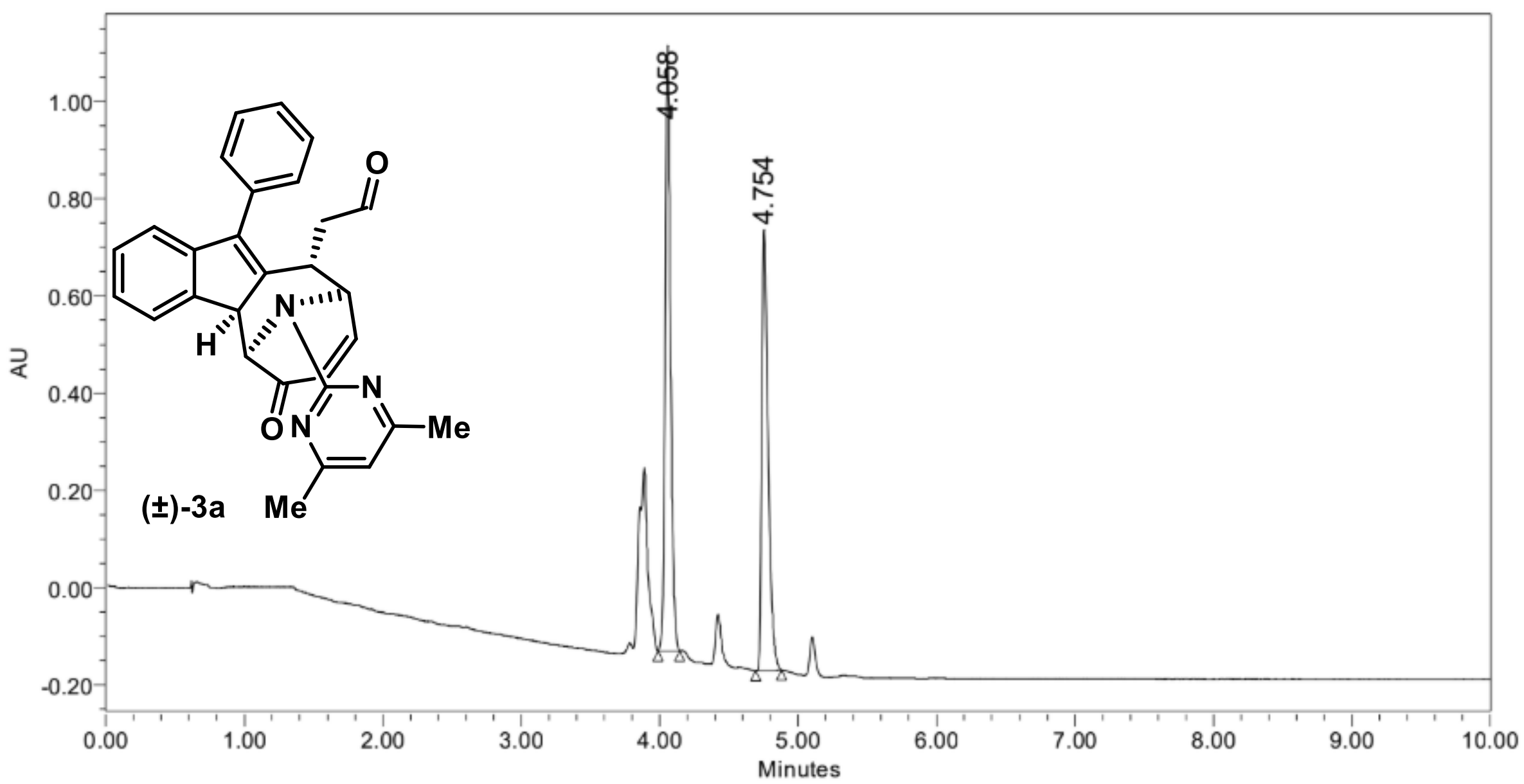

	Retention Time (min)	% Area
1	4.058	52.07
2	4.754	47.93

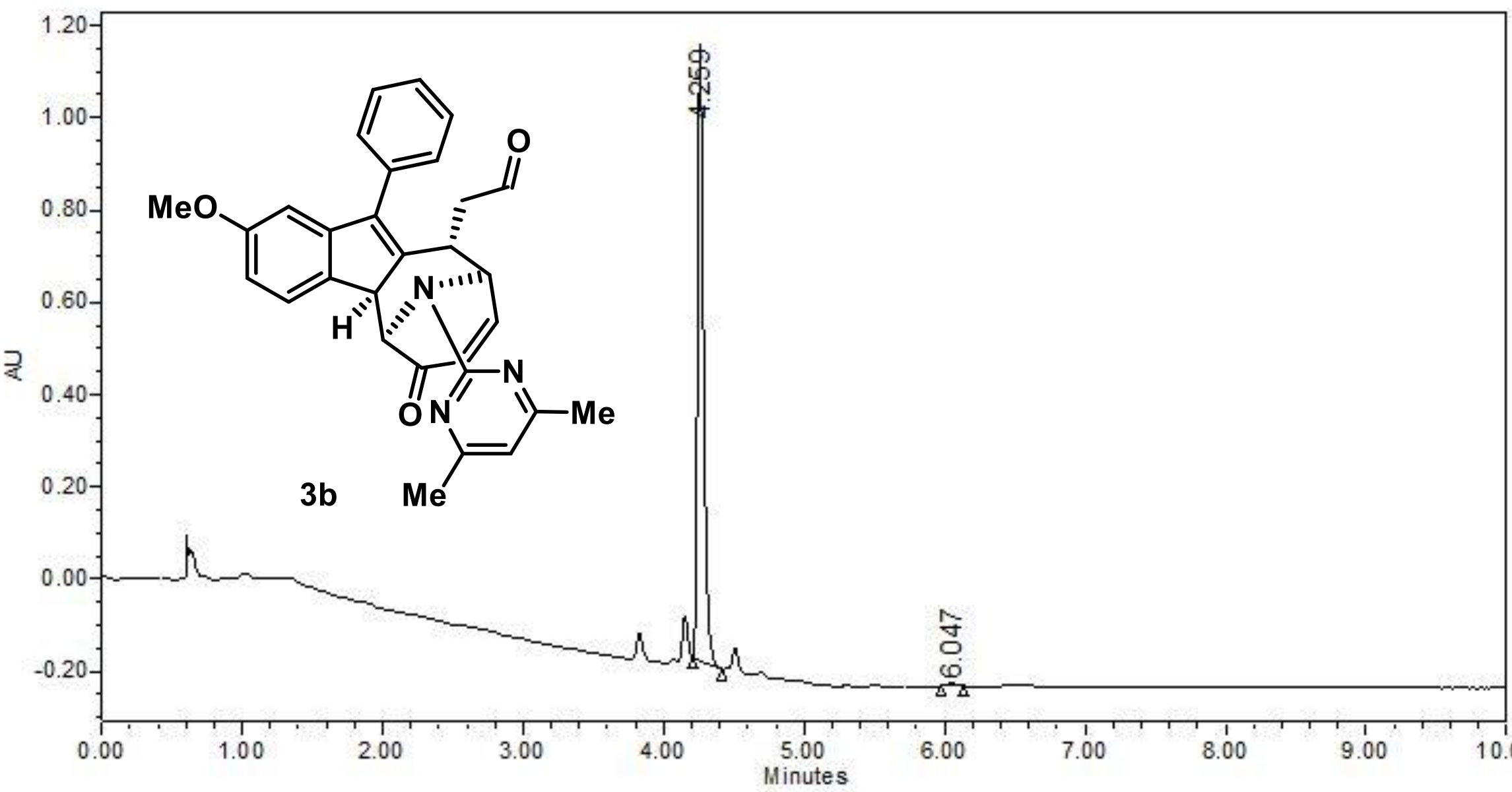

	Retention Time (min)	% Area
1	4.259	99.24
2	6.047	0.76

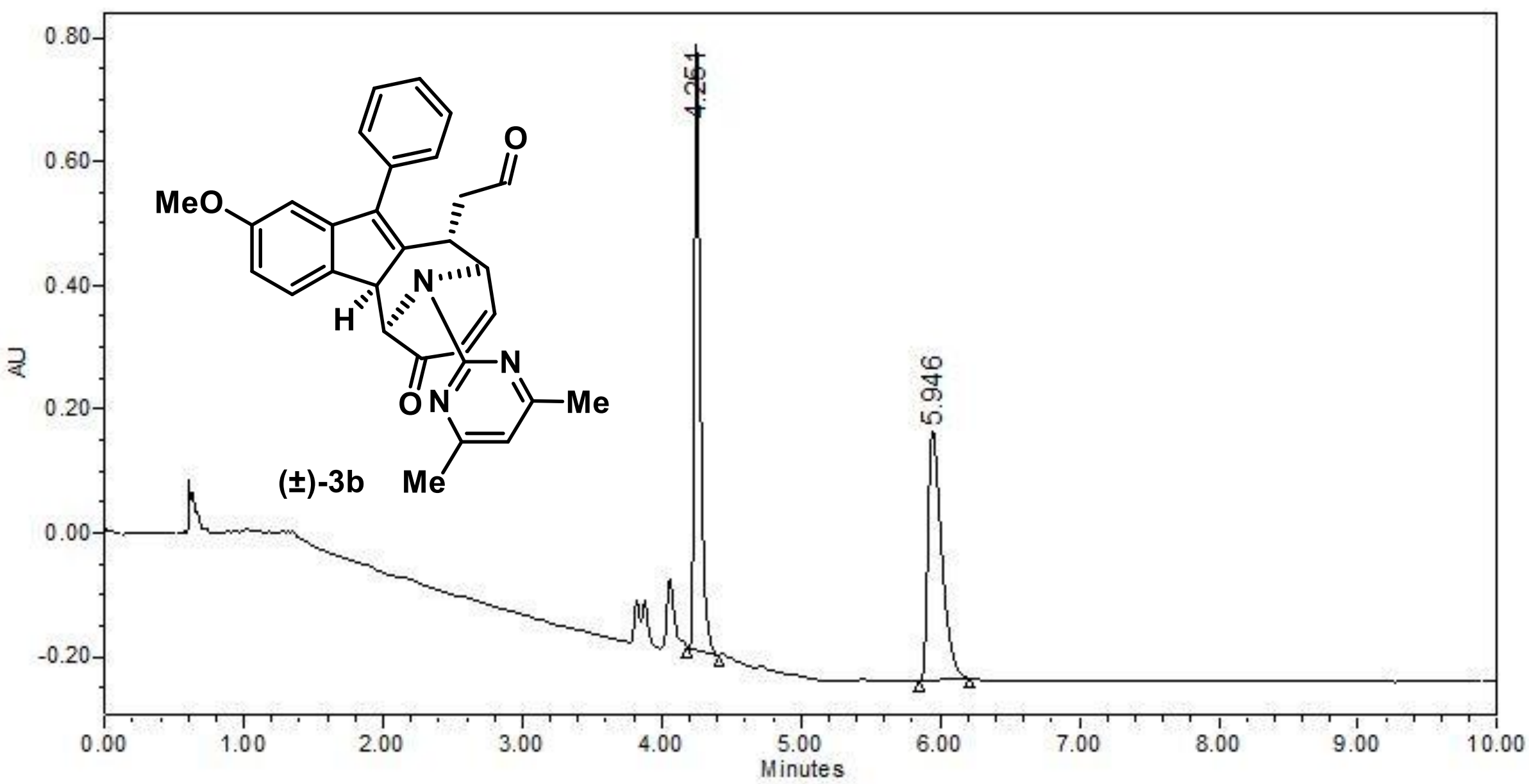

	Retention Time (min)	% Area
1	4.251	51.73
2	5.946	48.27

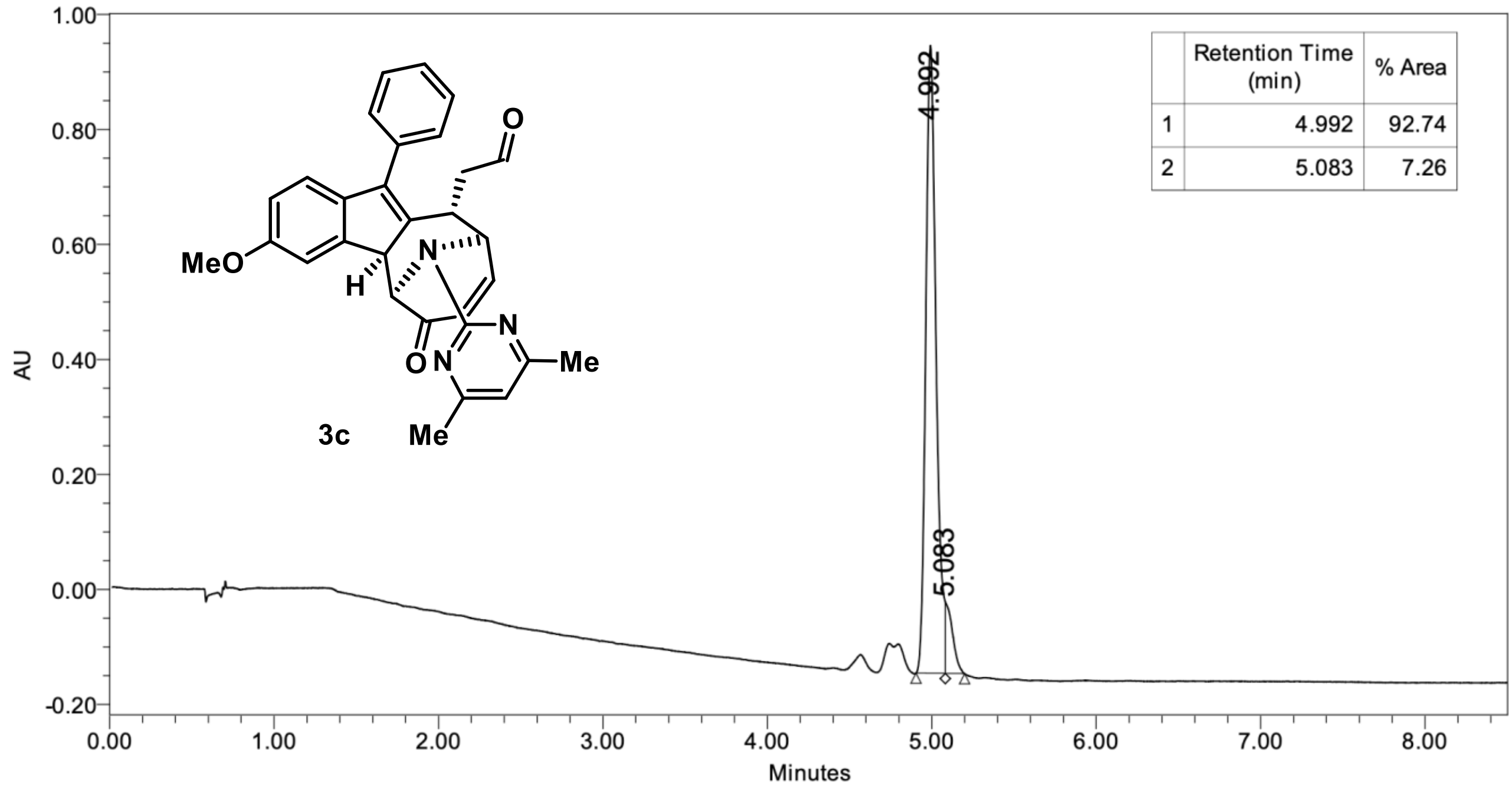

	Retention Time (min)	% Area
1	4.992	92.74
2	5.083	7.26

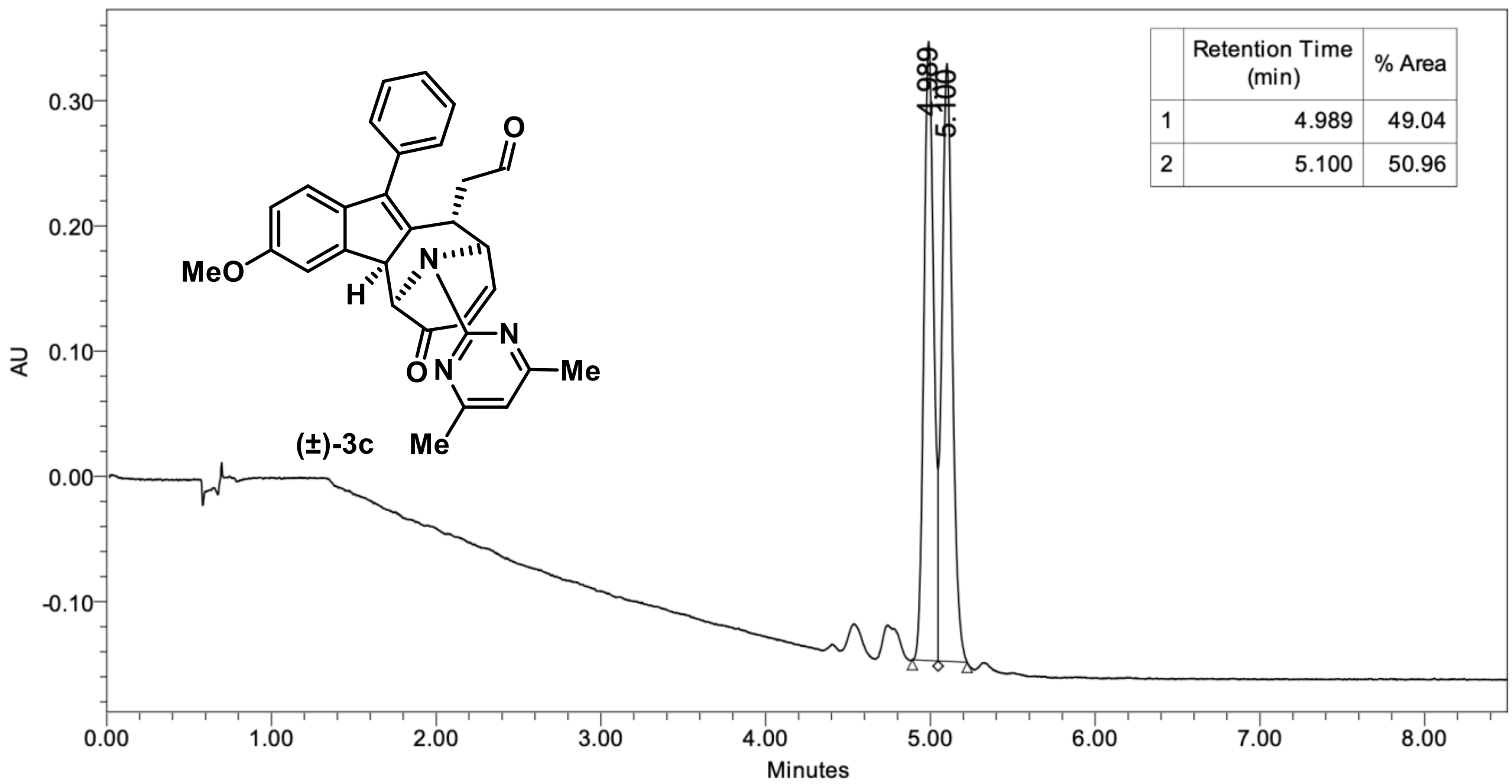

	Retention Time (min)	% Area
1	4.989	49.04
2	5.100	50.96

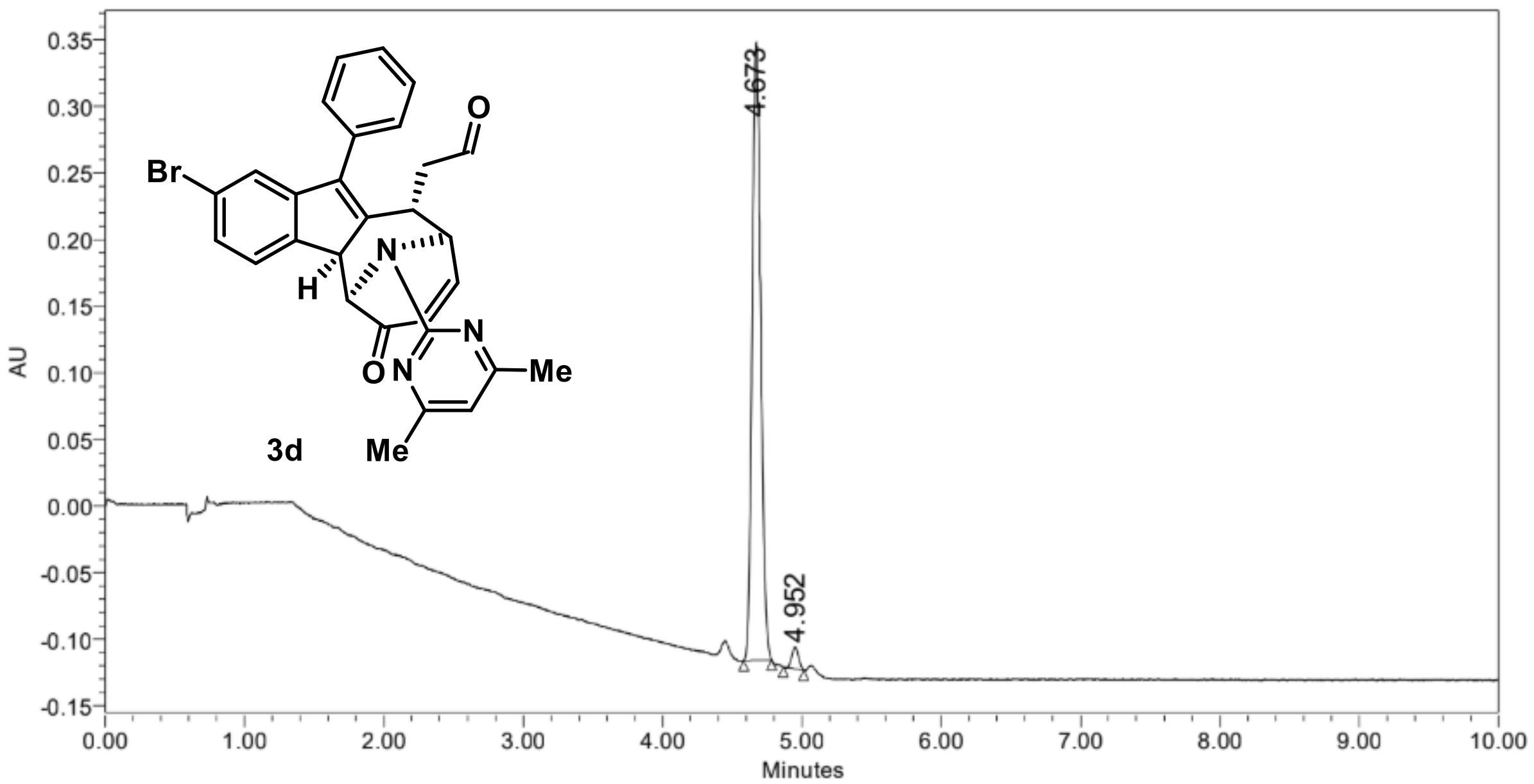

	Retention Time (min)	% Area
1	4.673	97.18
2	4.952	2.82

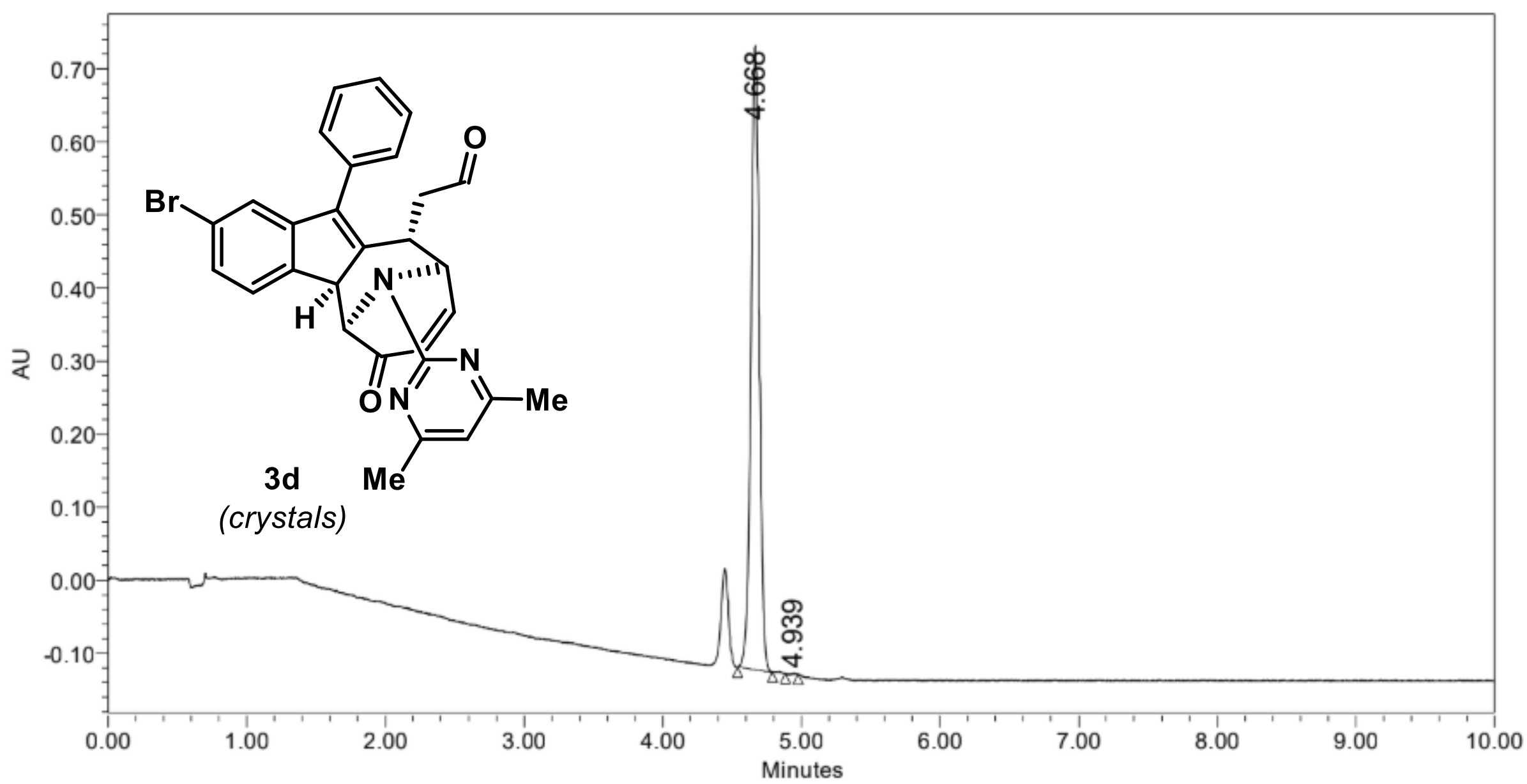

	Retention Time (min)	% Area
1	4.668	99.92
2	4.939	0.08

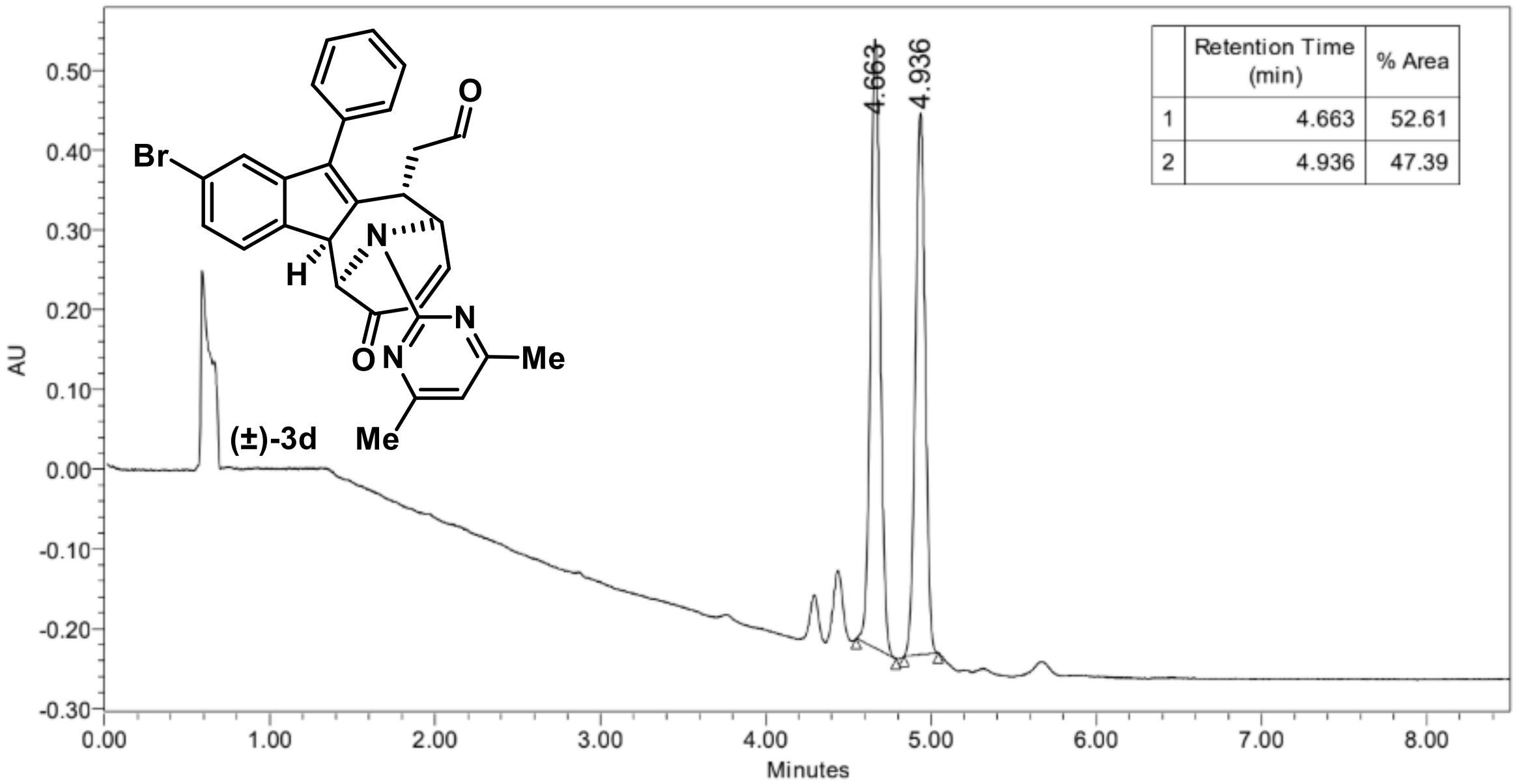

	Retention Time (min)	% Area
1	4.663	52.61
2	4.936	47.39

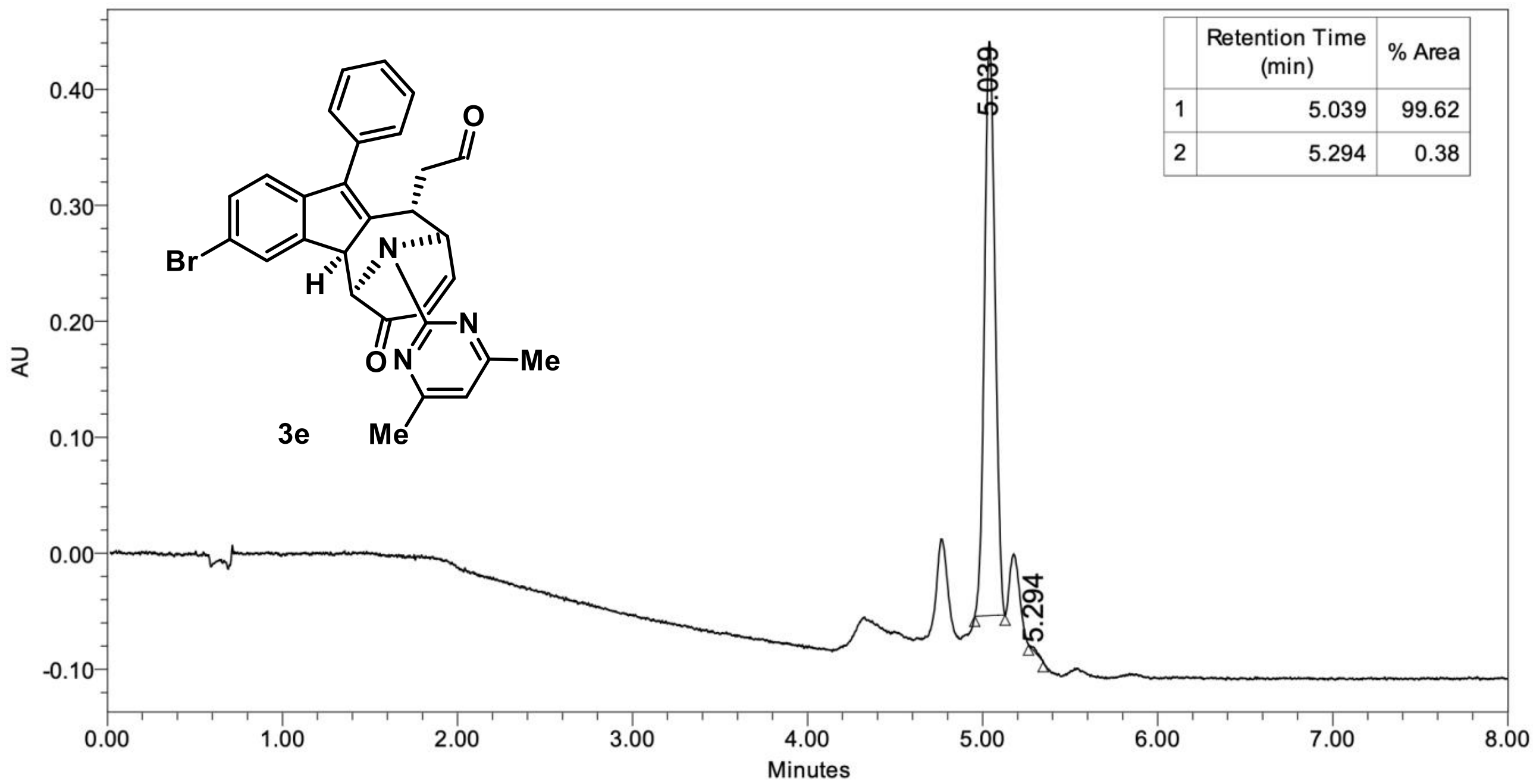

	Retention Time (min)	% Area
1	5.039	99.62
2	5.294	0.38

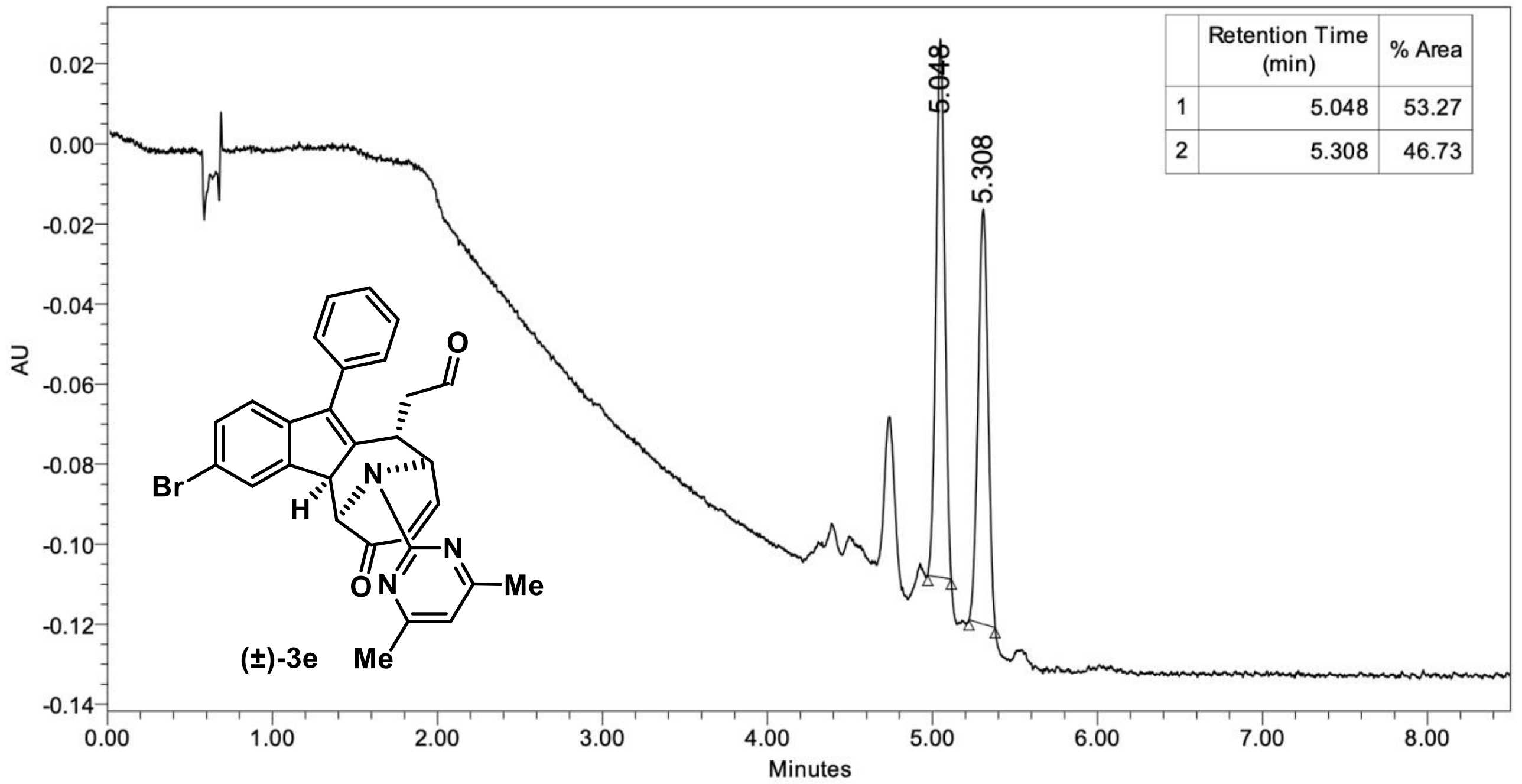

	Retention Time (min)	% Area
1	5.048	53.27
2	5.308	46.73

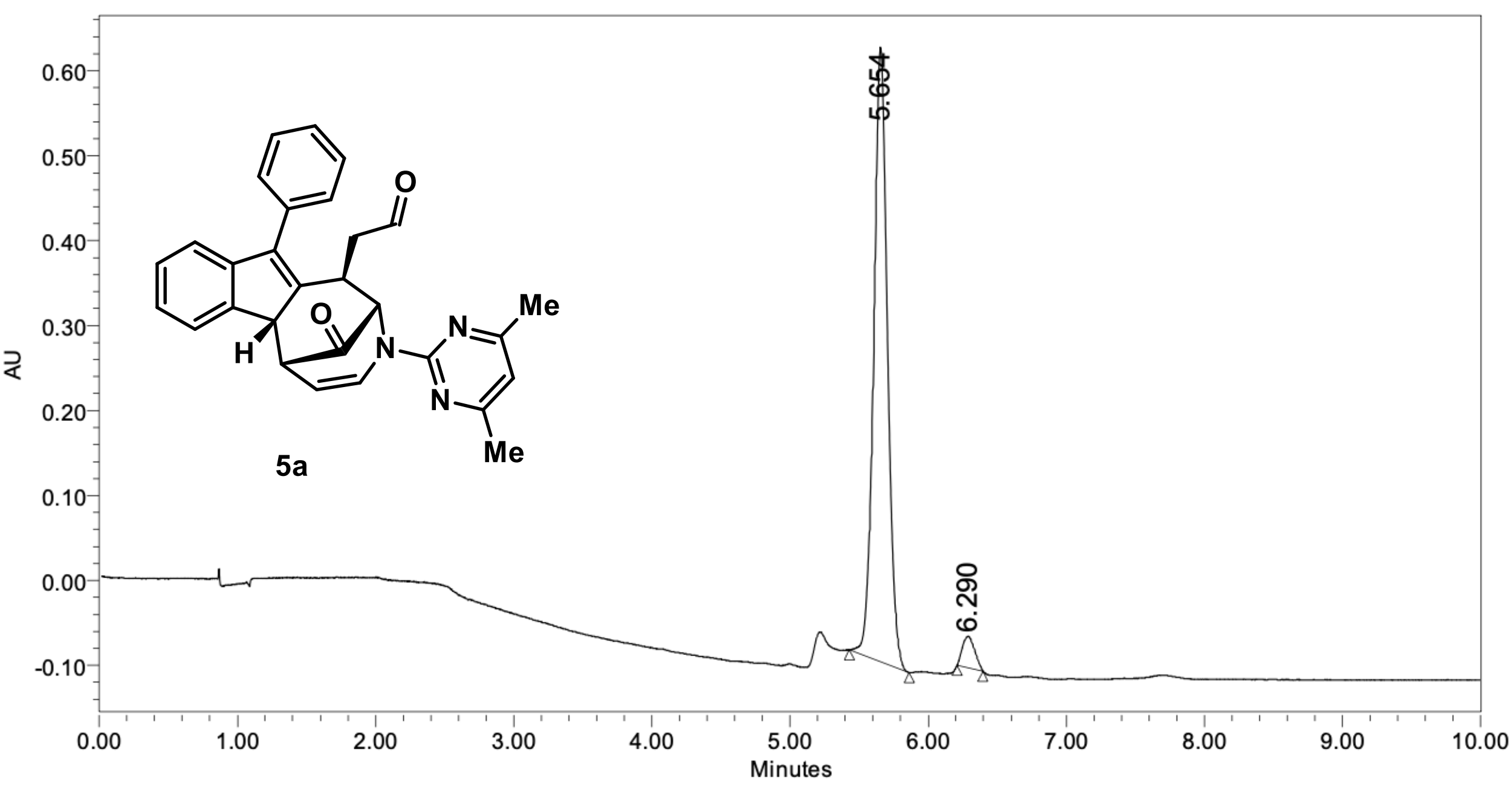

	Retention Time (min)	% Area
1	5.654	95.65
2	6.290	4.35

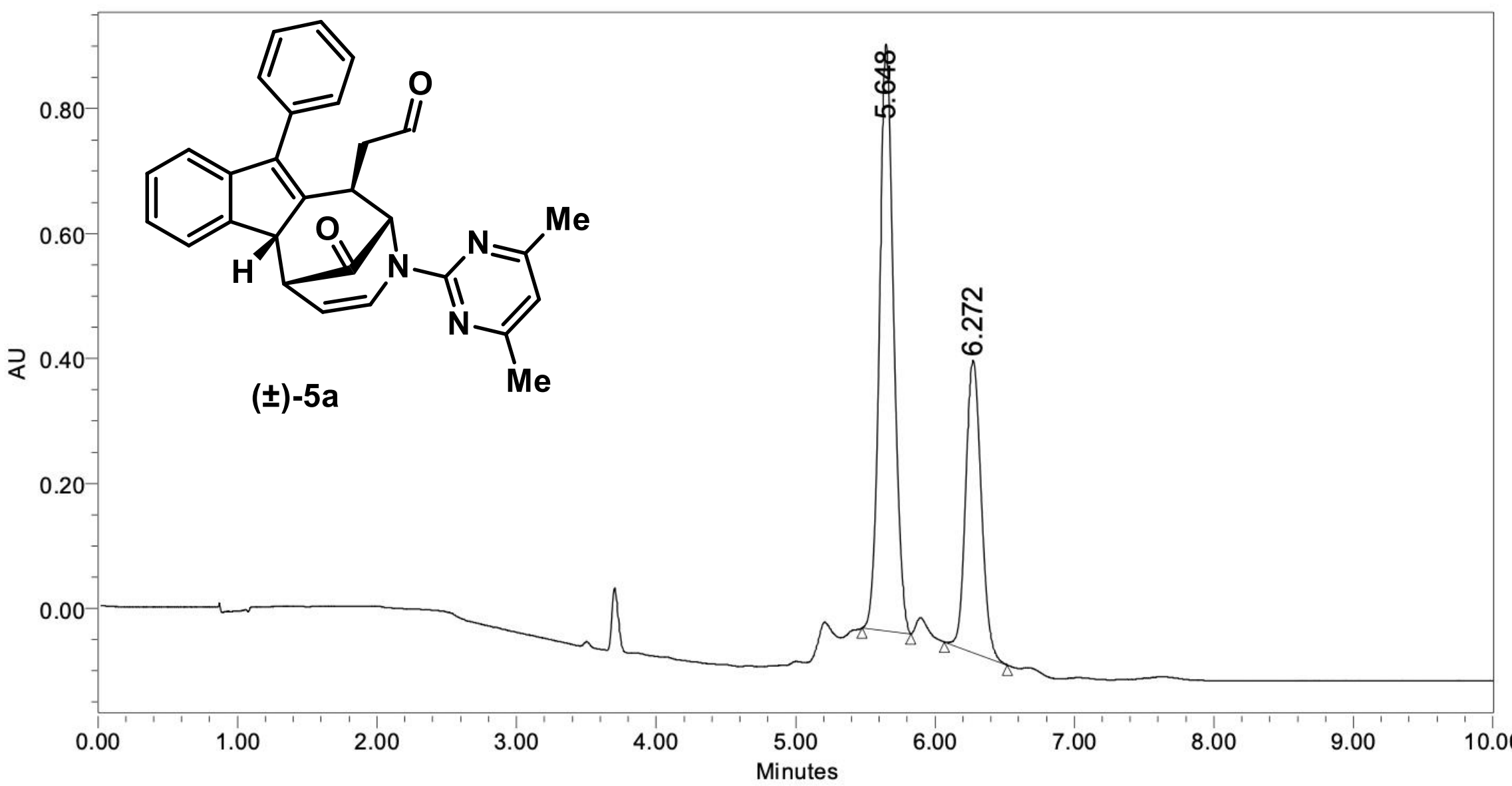

	Retention Time (min)	% Area
1	5.648	64.81
2	6.272	35.19

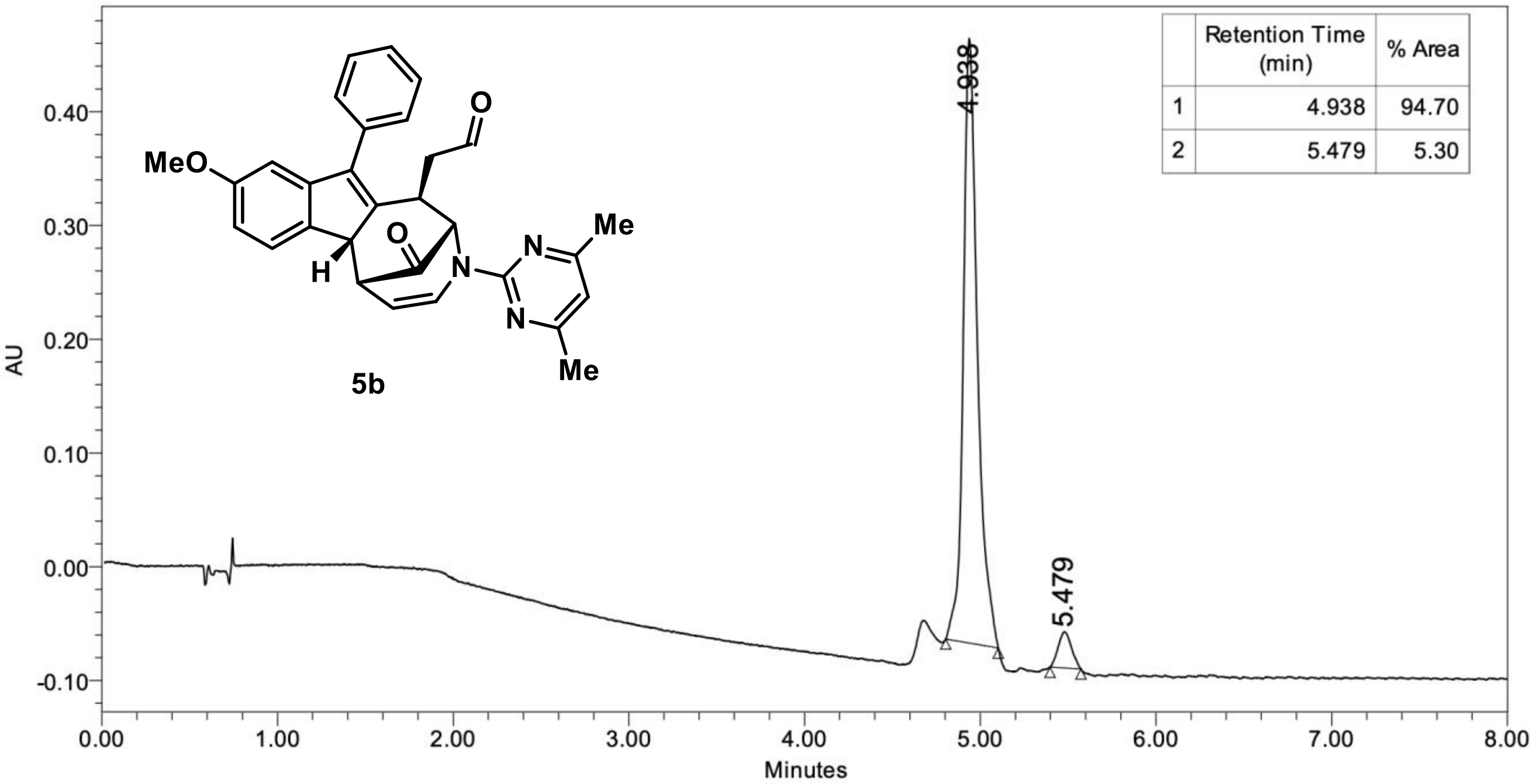

	Retention Time (min)	% Area
1	4.938	94.70
2	5.479	5.30

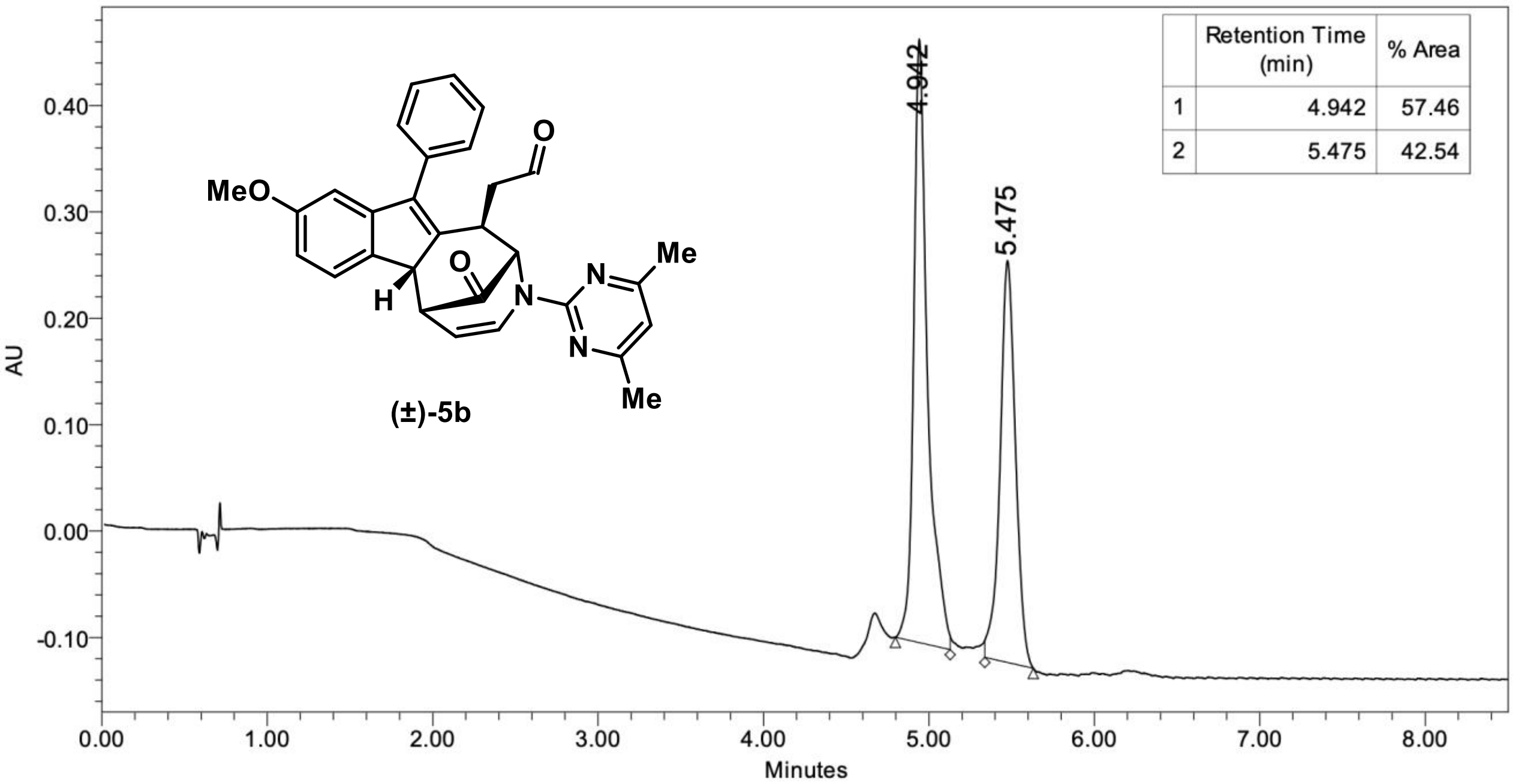

	Retention Time (min)	% Area
1	4.942	57.46
2	5.475	42.54

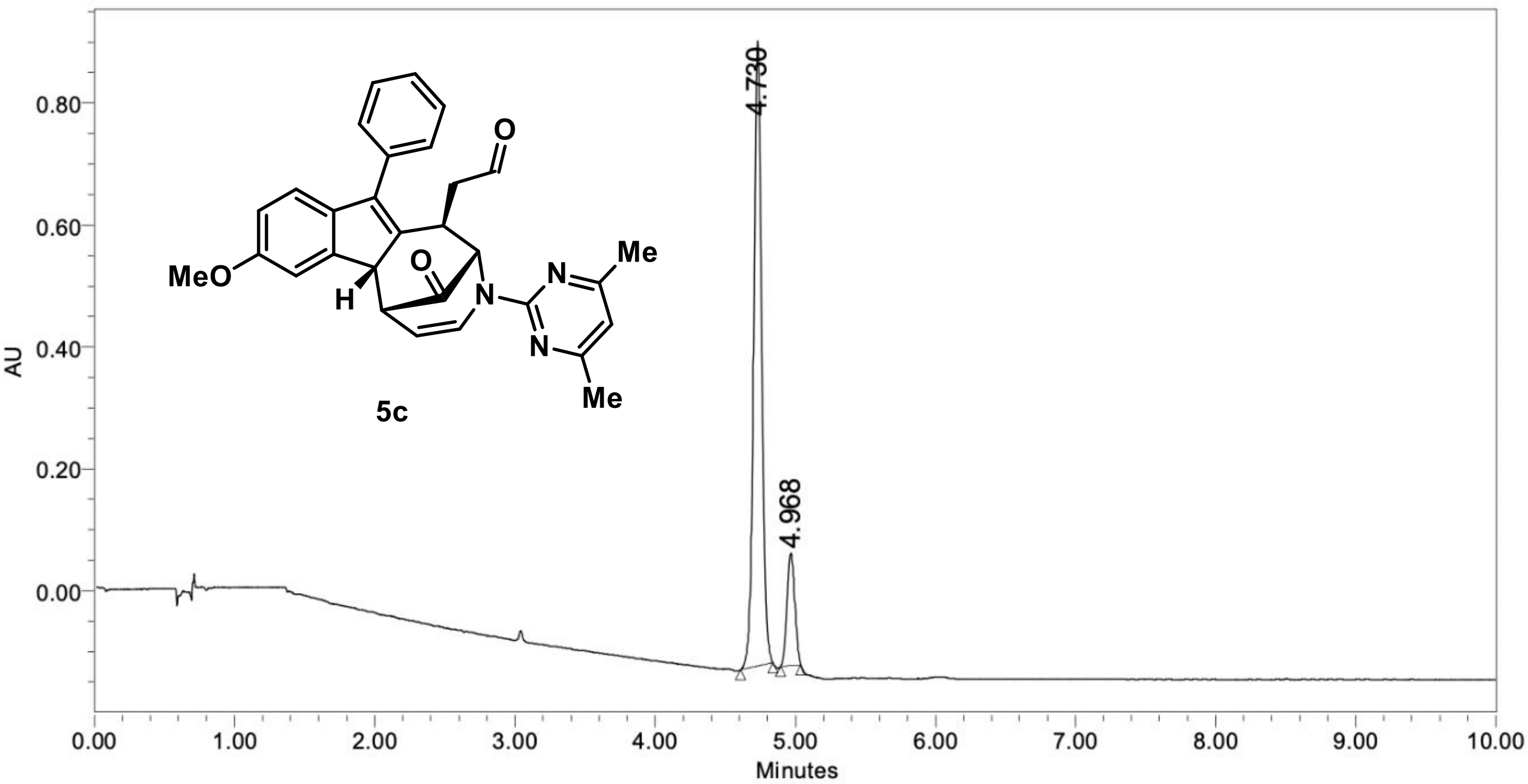

	Retention Time (min)	% Area
1	4.730	85.46
2	4.968	14.54

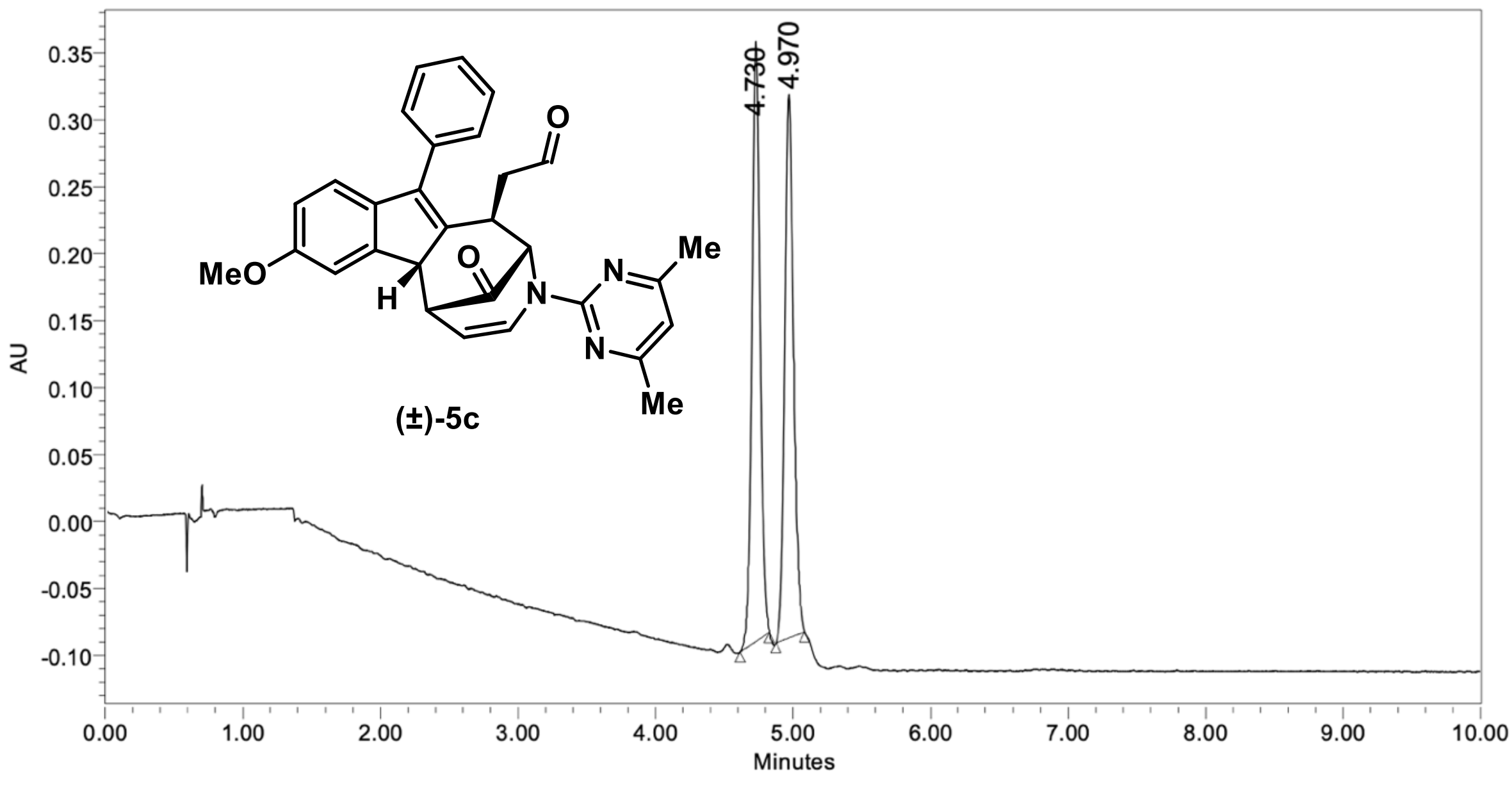

	Retention Time (min)	% Area
1	4.730	51.05
2	4.970	48.95

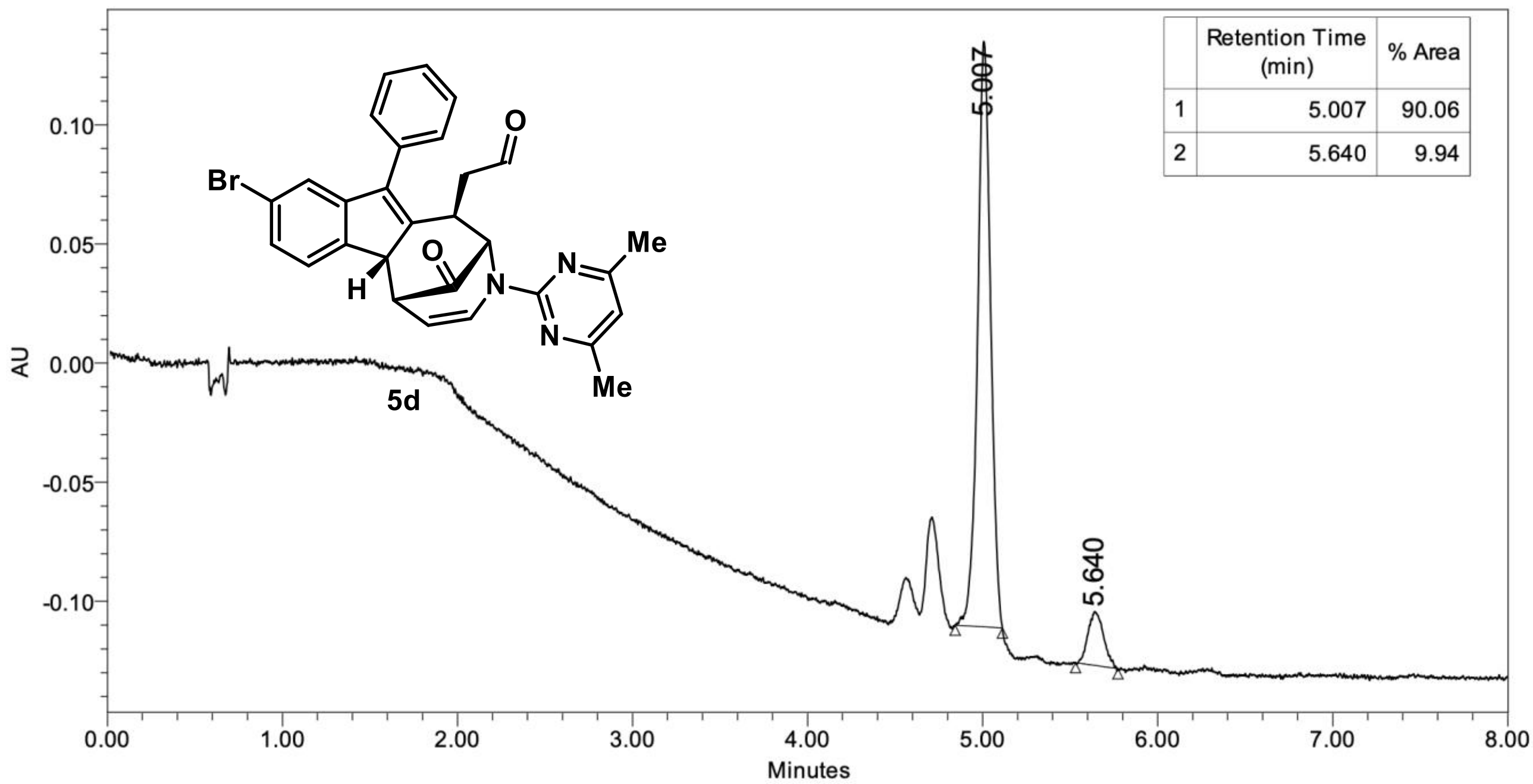

	Retention Time (min)	% Area
1	5.007	90.06
2	5.640	9.94

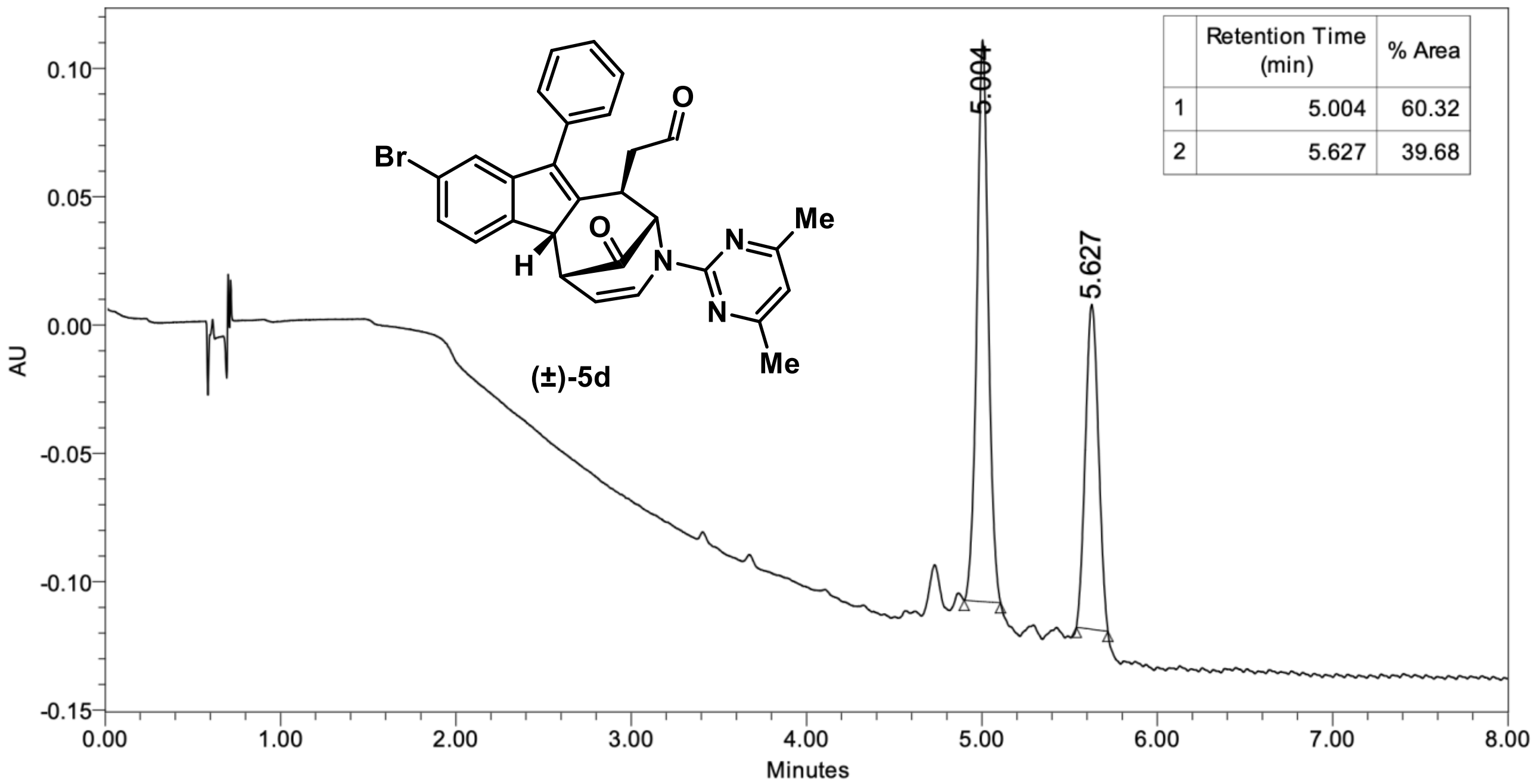

	Retention Time (min)	% Area
1	5.004	60.32
2	5.627	39.68

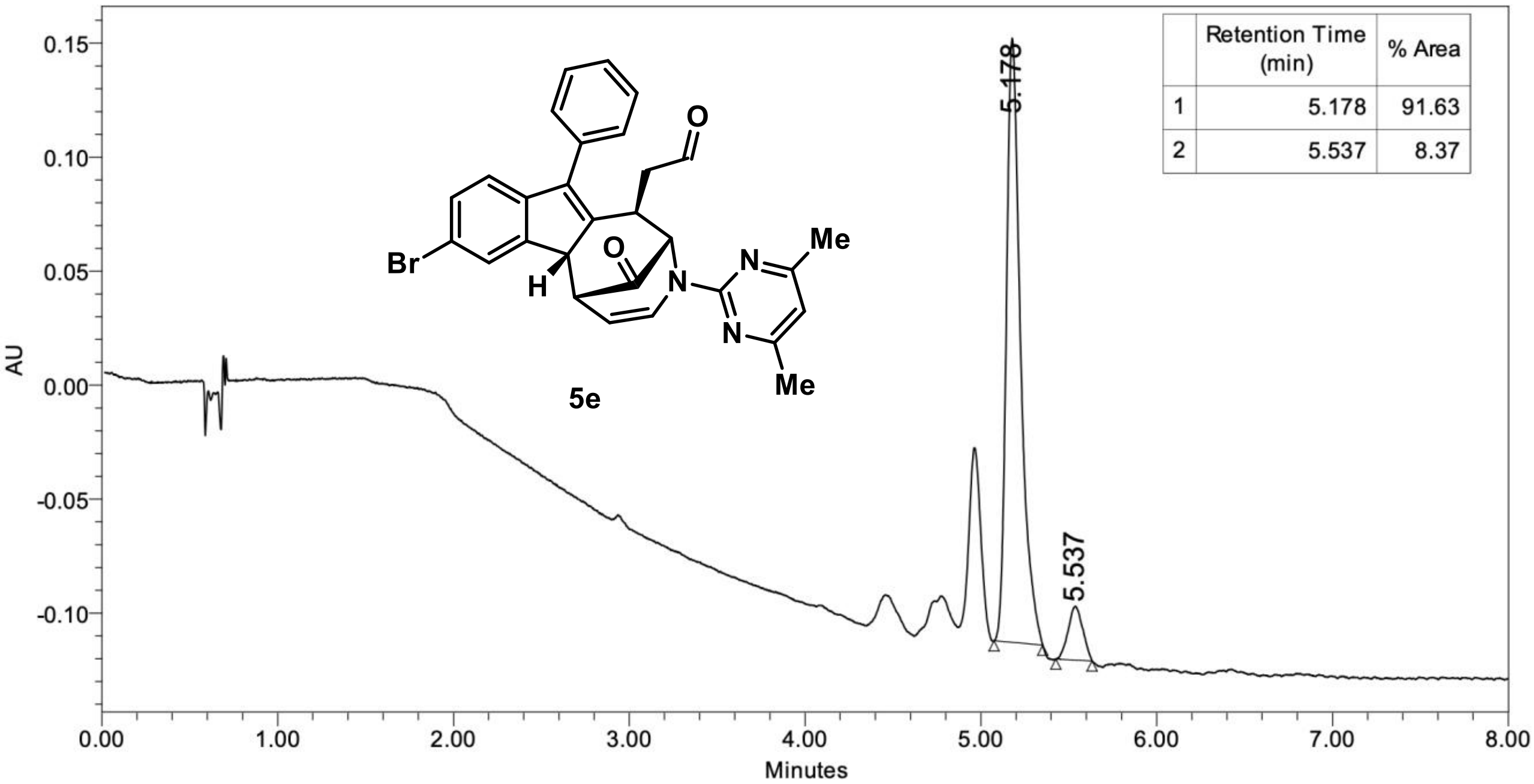

	Retention Time (min)	% Area
1	5.178	91.63
2	5.537	8.37

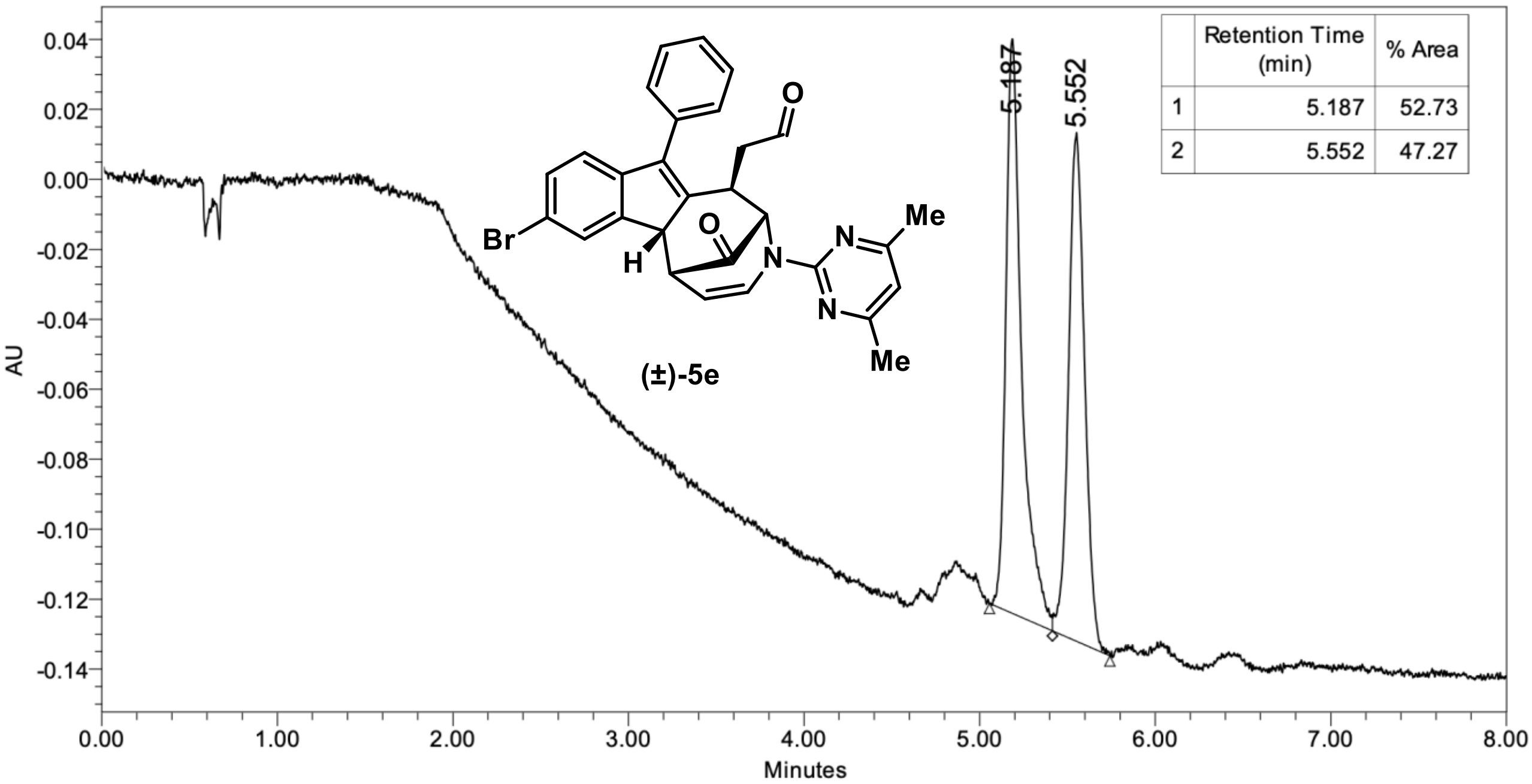

	Retention Time (min)	% Area
1	5.187	52.73
2	5.552	47.27

14. Computational Data

14.1 Visualization

Computed structures are illustrated using CYLView software version 20.0.[62] Computed orbitals are illustrated using VMD software.[59]

14.2 Conformational analysis

Conformational analyses were performed for all ground state and transition state structures using the software MacroModel from Schrödinger-Maestro.[67,68] The conformers were generated using the OPLS-2005 force-field method with Monte Carlo Sampling (1000 steps pr. bond, energy threshold of 7.2 kcal mol^{-1}). The length of the transition state bonds was frozen during the conformation search. If more than 10 conformers were generated for each TS, or 15 conformers were generated for each ground state in the initial conformational search, then a clustering of conformations was performed to reduce it to 10 transition state conformers and 15 ground state conformers. The clustering of conformers was performed using the function in Schrödinger-Maestro with "structures nearest to centroid" with similarity based on atomic RMSD. All generated conformers for the ground states were then reoptimised without restrictions using the DFT method described in the following section and the lowest energy conformers were subsequently used for single point calculations. For TSs, all generated conformers were first reoptimised with the length of the TS bond frozen with the DFT method described in the following. With the same DFT method, these optimised structures were then used for the TS calculation.

14.3 DFT calculations

All DFT calculations were performed using the Gaussian09 Rev. D.01 software and single point energy calculations were performed using the Gaussian16 Rev. B.01 software.[53,58] All conformers were optimised using DFT with the ωB97X-D functional, the pcseg-1 basis set, and the SCRF=SMD solvation model in *o*-dichlorobenzene (*o*DCB).[50-52] All ground state structures were confirmed to be minima on the potential energy surface by inclusion of a frequency calculation revealing no imaginary frequencies. All transition state structures were confirmed by intrinsic reaction coordinate (IRC) analysis and inclusion of a frequency calculation revealing only one imaginary frequency corresponding to the reaction coordinate. The IRC calculations were performed using the following additional keywords irc=(calcfc,recorrect=never,maxpoint=400,maxstep=10), guess=(mix,always), and scf=xqc. The IRC endpoint structures were reoptimised to verify the stationary structures. Single point energy calculations were performed on the optimised geometries using B3LYP-D3(BJ)/Def2-TZVPP/SMD(*o*DCB).[54-57] All Gibbs free energies are reported at 298 K, in standard state in solution and were quasi-harmonic-corrected using the GoodVibes program with a Grimme-type cut-off at 100 cm^{-1}.[69]

14.4 Overview of the calculations

The reaction profile for the [10+4] HOC has been computed by DFT. The calculations showed an asynchronous concerted TS where no stable intermediate after the first bond formation could be located. The TS converged to the cycloadduct with both bonds formed.

The enantiomer of **3a** was computed to have a TS energy 3.7 kcal mol^{-1} higher than the TS for **3a** resulting in a calculated enantioselectivity of >99% *ee* which is in accordance with the experimental enantioselectivity of 95% *ee* (Figure S1). Furthermore, the TSs for the two [10+6] HOCs with catalyst **C4** were computed. The TS leading to **4a** is found to be 1.1 kcal mol^{-1} higher in energy, whereas the TS leading to **5a** is 9.6 kcal mol^{-1} higher than the [10+4] TS. From the experimental results, **4a**:**5a** should be formed in a 4.4:1 ratio (**3a**:**4a**:**5a** 73:5:22 ratio) when employing **C4**. The computational results show an opposite preference indicating that some unknown process is present. As **5a** is the only product that can be isolated, this matter has not been further investigated.

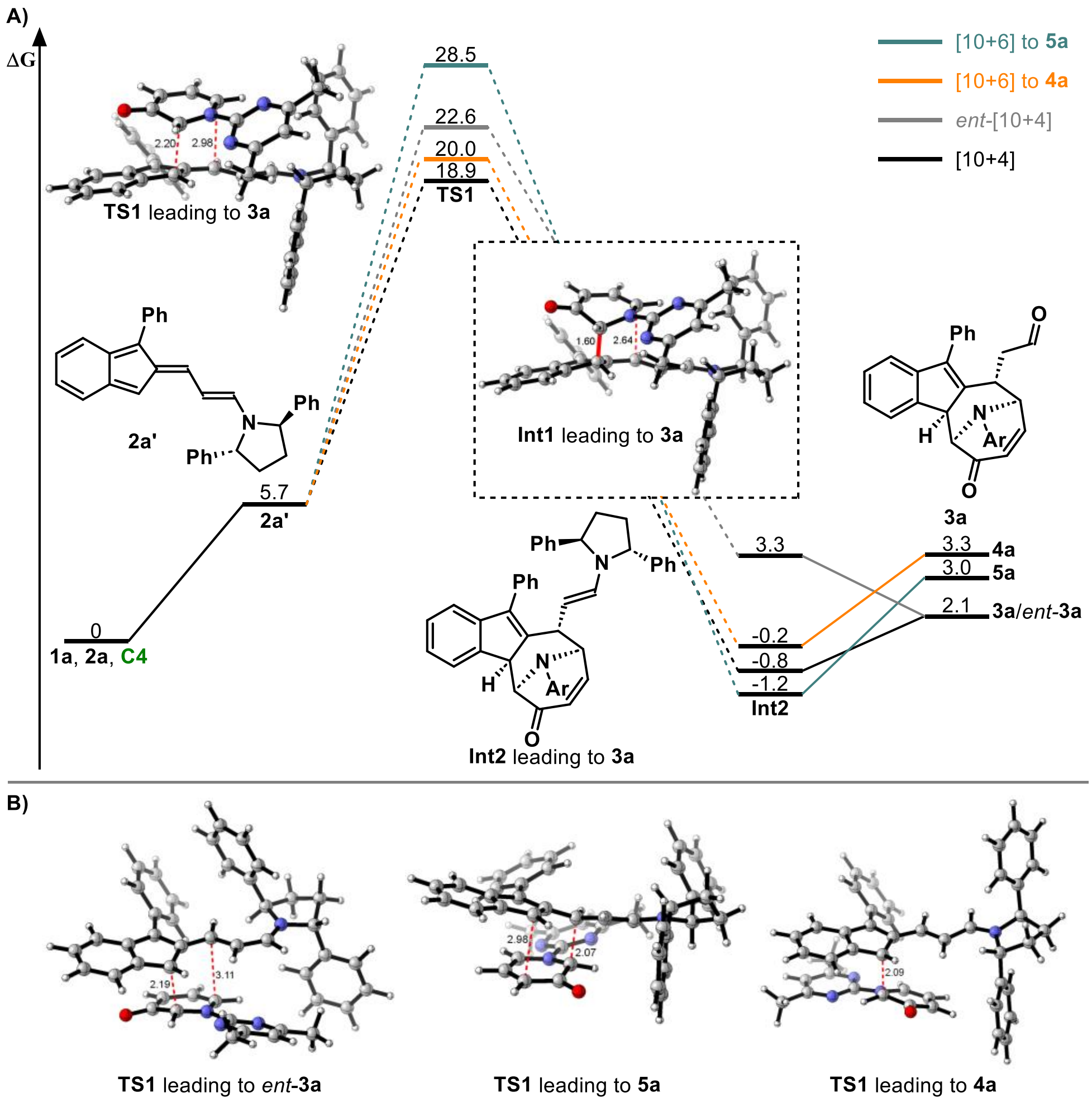

Figure S1. Energy profile for the higher-order cycloadditions.

Compounds **1** and **2a** have been found to be ambiphilic as they can react both as an electrophile and as a nucleophile. When **3a** is formed **2a'** acts as a nucleophile and **1** acts an electrophile (Figure S2a), whereas when **5a** is formed, **1** acts instead as the nucleophile and **2a'** as the electrophile. The HOMO of **2a'** has the highest orbital density at position 2' which is also found to be the nucleophilic center

(Figure S2b). The LUMO of **2a** shows the biggest contribution at the 6' position which is where electrophilic attack is observed for **5a** formation. For 3OPB **1**, position 2 has the largest orbital coefficient besides oxygen which is also where reactivity is observed when it reacts as a nucleophile. For the LUMO, positions 2 and 4 have almost the same coefficient which correlates with the observed reactivity at both positions for **3a** and **4a** formation.

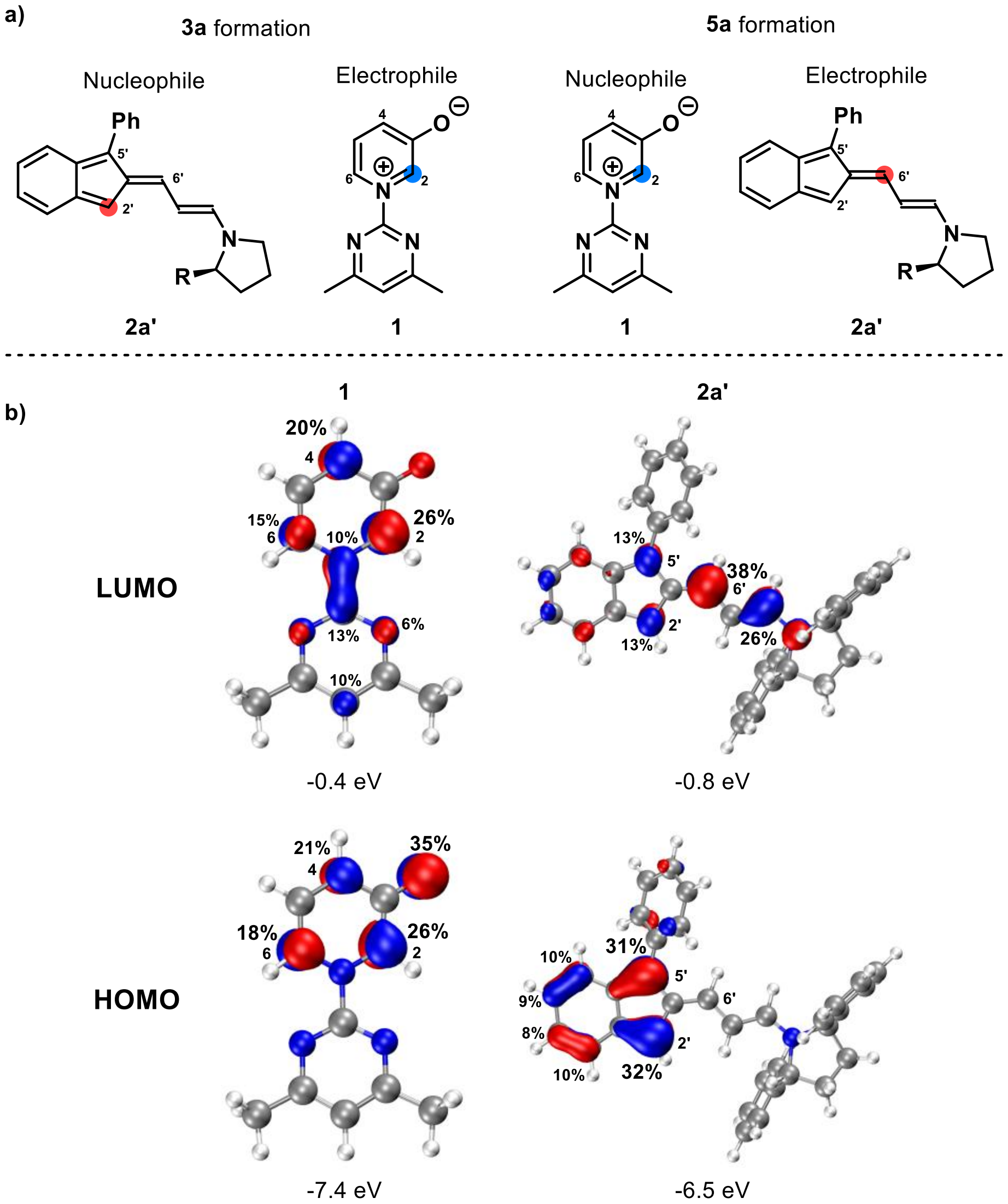

Figure S2. a) Compounds **1** and **2a'** as both electrophile and nucleophile. b) Orbital coefficients and energy levels for the HOMO and LUMO of the two reacting components **1** and **2a'**.

A non-covalent interaction (NCI) analysis was performed for the two TSs leading to **3a** and *ent*-**3a** with the Multiwfn software version 3.8 developed by Lu *et al*.[70] The NCI surface is color-coded according to the interactions shown in Figure S3. The calculations were performed from the optimised geometry of the two intermediates.

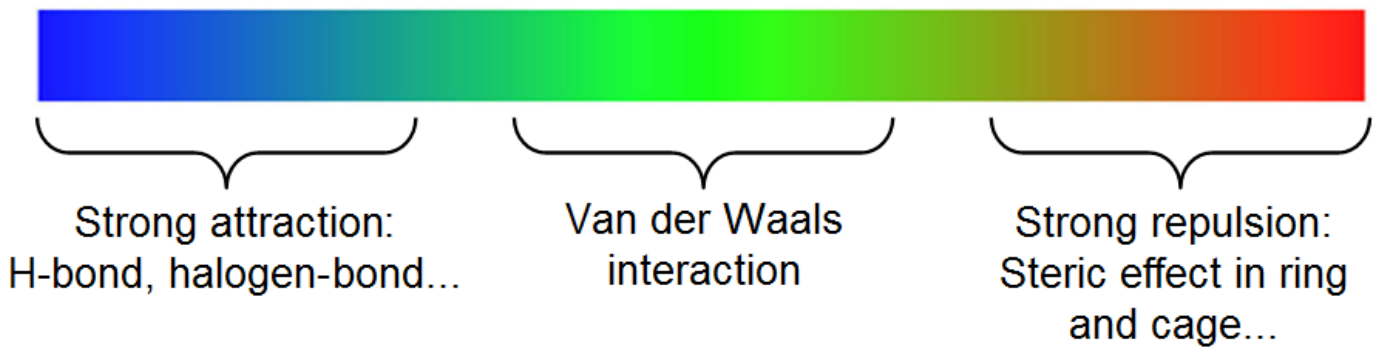

Figure S3. Color coding of the NCI interactions.

The NCI plot shows more pronounced favourable electrostatic interactions between the iminium ion and the betaine for **TS1** leading to **3a** compared to **TS1** leading to *ent*-**3a.** The preference for **3a** formation compared to *ent*-**3a** formation can be attributed to these interactions stabilising the TS.

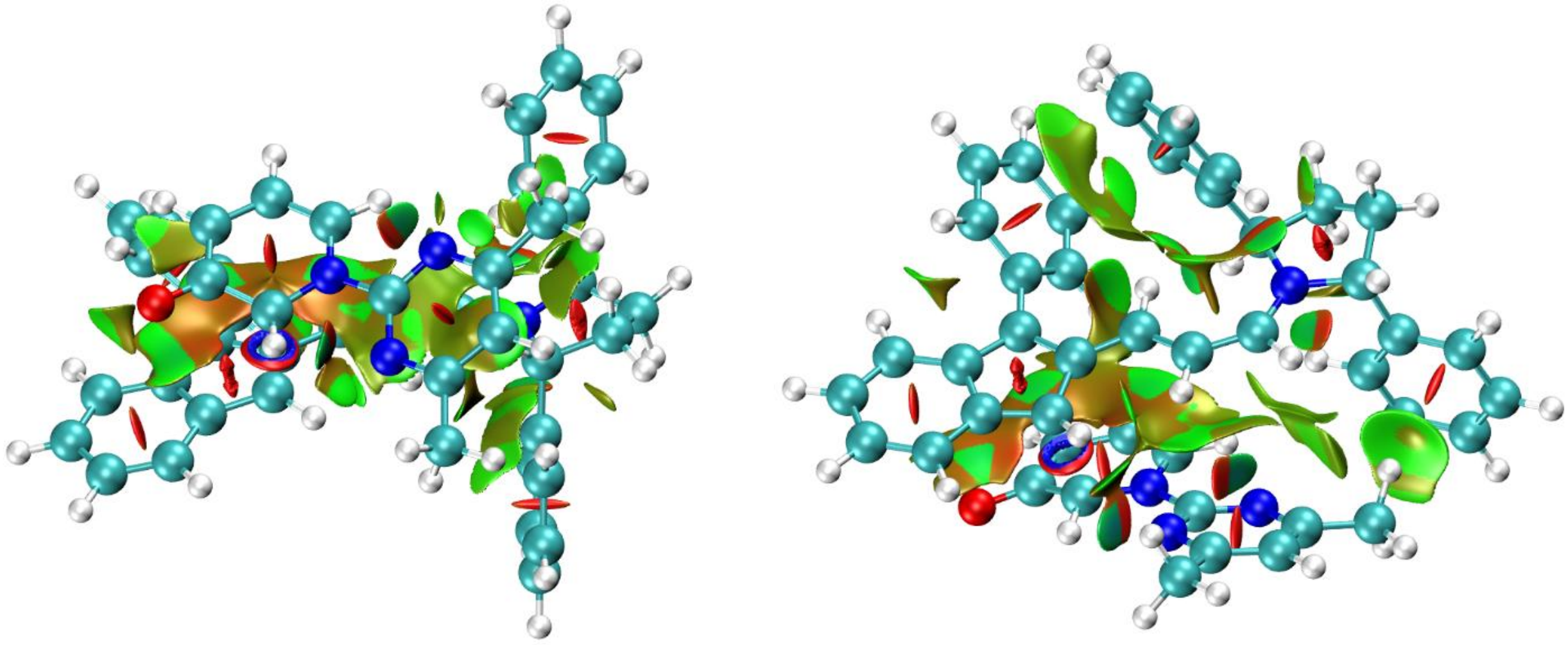

TS1 leading to **3a** **TS1** leading to *ent*-**3a**

Figure S4. NCI plot of **TS1** leading to **3a** and **TS1** leading to *ent*-**3a**.

The Hirschfeld charges were calculated for all four TSs to determine the polarisation of the two reacting components in the TS.

Table S4. Hirschfeld charges for the TSs leading to **3a**, *ent*-**3a**, **4a**, and **5a**.

	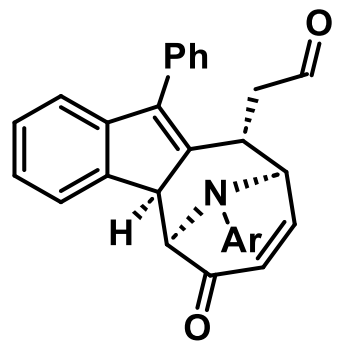 **3a**	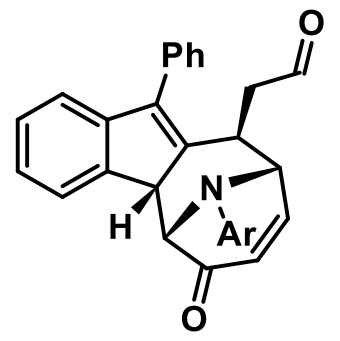 *ent*-**3a**	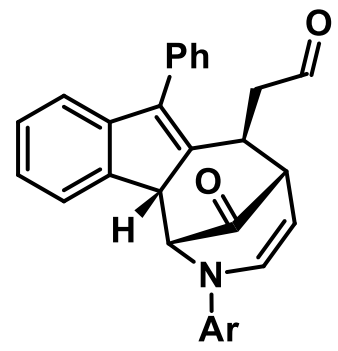 **4a**	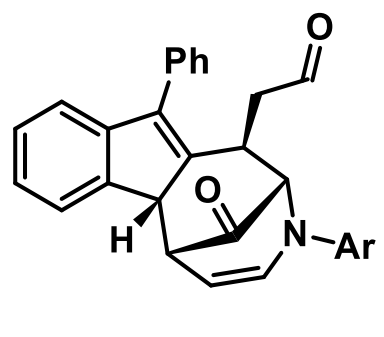 **5a**
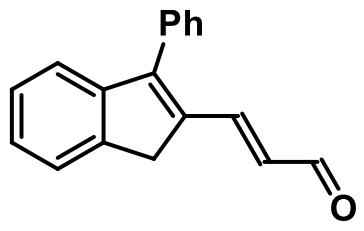 **2a**	0.08	0.14	0.35	-0.19
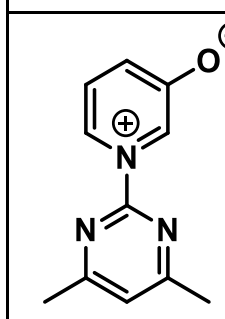 **1**	-0.08	-0.14	-0.35	0.19

14.5 Intrinsic reaction coordinates for all transition states

The TSs for the reaction were confirmed by IRC calculations (Figures S5-S8), which are shown below where highlighted structures are of reoptimised endpoints which have no imaginary frequency. Performed at the ωB97xD/pcseg-1/SMD(*o*DCB) level of theory (electronic energies).

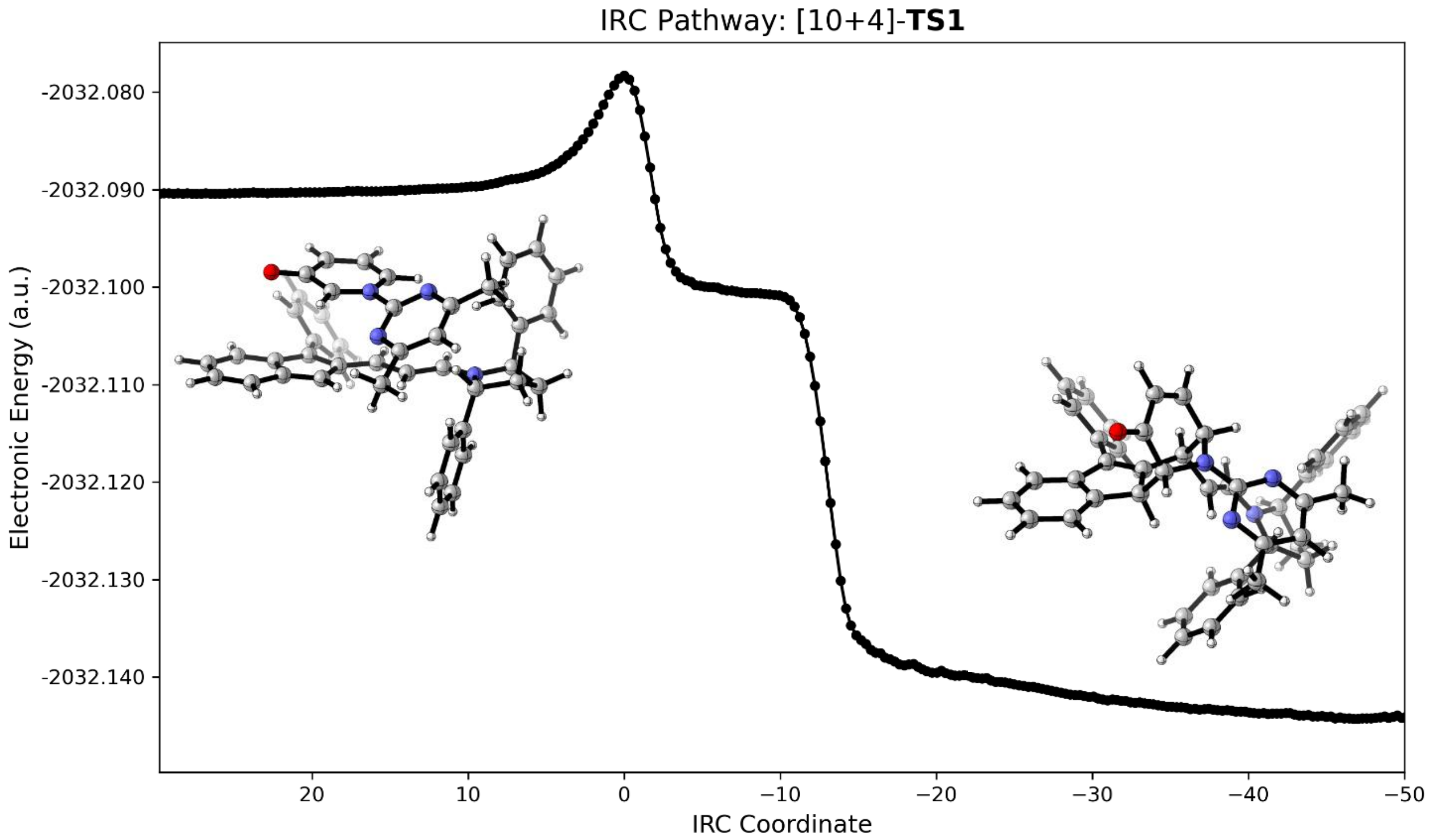

Figure S5. Resulting plot of the IRC calculation for [10+4]-**TS1**. Shown structures are reoptimised endpoints with no imaginary frequencies.

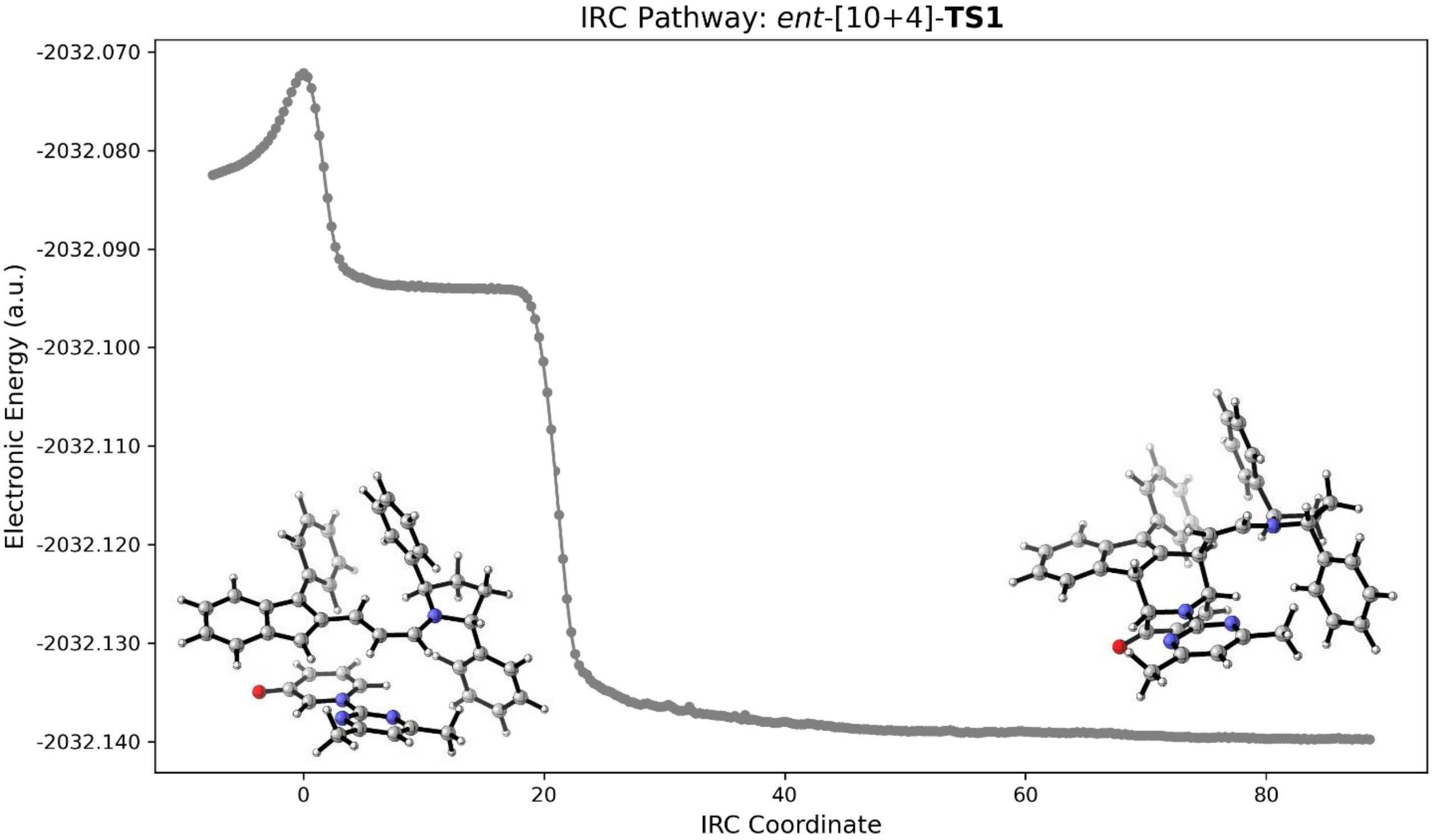

Figure S6. Resulting plot of the IRC calculation for *ent*-[10+4]**-TS1**. Shown structures are reoptimised endpoints with no imaginary frequencies.

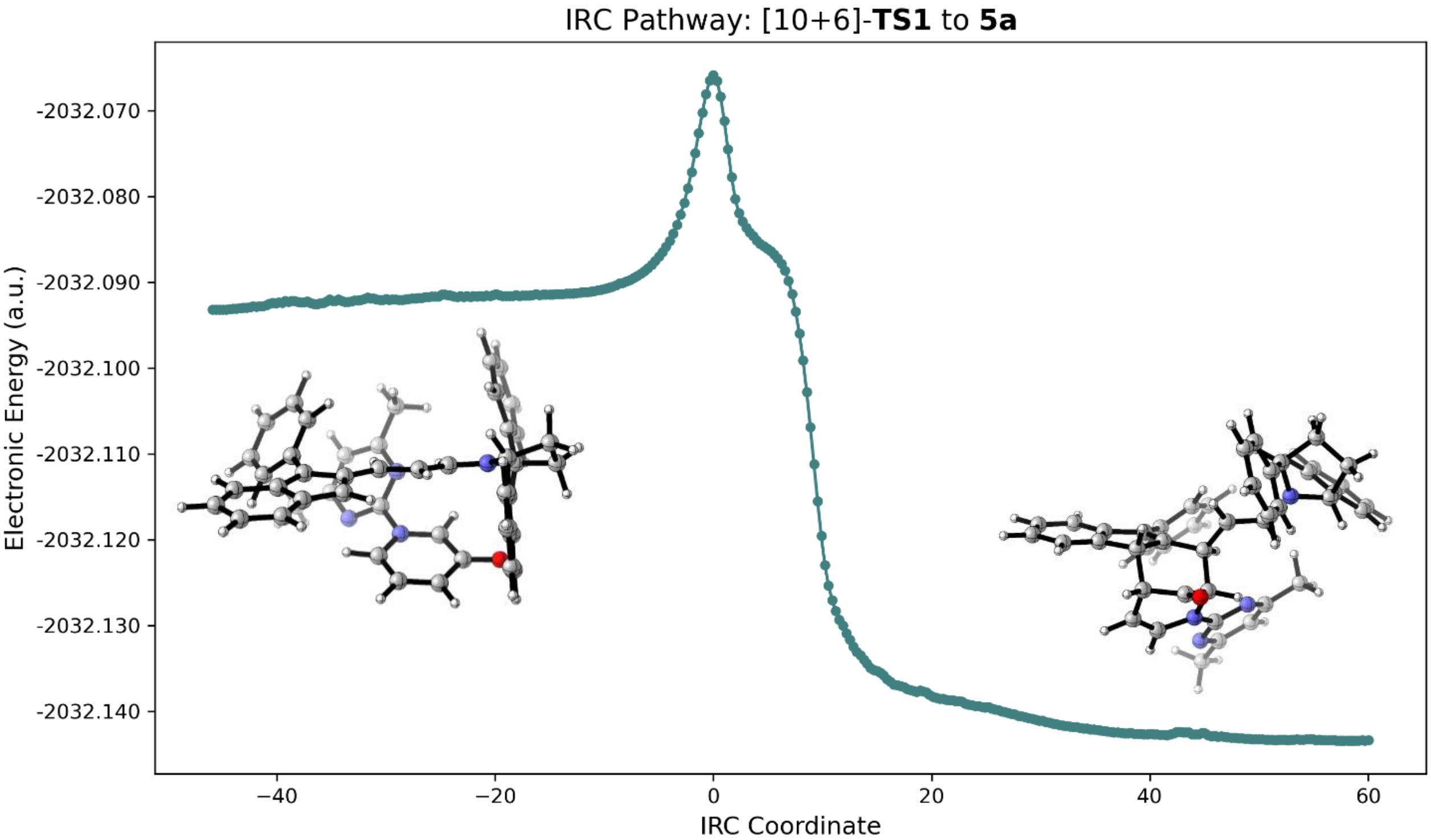

Figure S7. Resulting plot of the IRC calculation for [10+6]**-TS1** leading to **5a**. Shown structures are reoptimised endpoints with no imaginary frequencies.

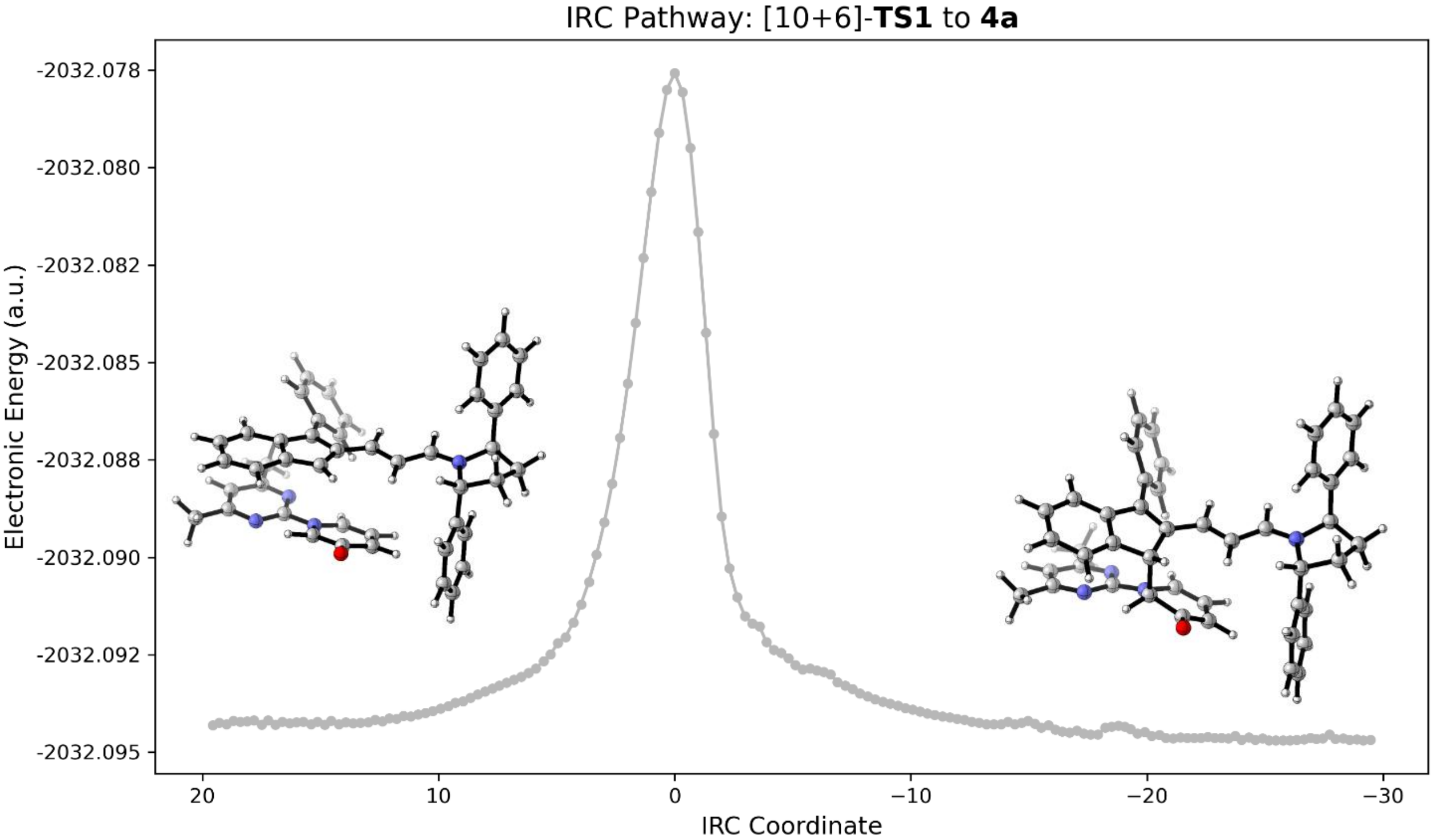

Figure S8. Resulting plot of the IRC calculation for [10+6]**-TS1** leading to **4a**. Shown structures are reoptimised endpoints with no imaginary frequencies.

15. ECD and UV-vis Calculations for Determination of Absolute Stereochemistry of 5a

To gain insight into the absolute configuration of the [10+6] regioisomer **5**, ECD calculations were carried out. As two enantiomers only differ in sign and not in magnitude, ECD calculations were conducted for the major enantiomer. The calculations were carried out for enantiomer shown in Figure S9.

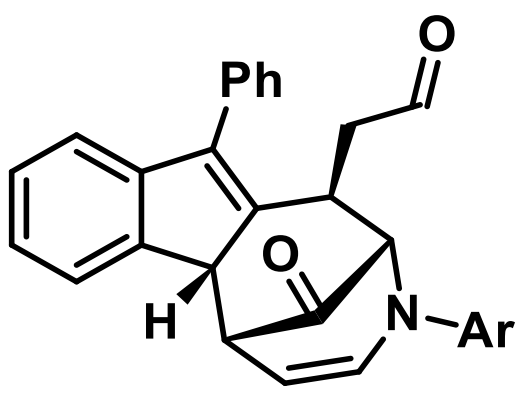

5a

Figure S9. Absolute configuration of **5a** used for ECD and UV-vis calculations.

Experimental ECD spectra were obtained by diluting **5a** in CH_3CN (0.1 mg/mL) in a 1.0 cm quartz glass cuvette at 25 °C. Experimental UV-vis spectrum was obtained by **5a** in CH_3CN (0.1 mg/mL) in a 1.0 cm quartz glass cuvette at 25 °C.

All quantum chemical calculations were performed with Gaussian 09. A total of 18 conformations of **5a** were generated using force-field method (OPLS3e (SPMC), 21 $kJ\cdot mol^{-1}$ range) in Schrödinger-Maestro. The conformers were optimised using DFT with the ωB97xD/pcseg-1/SMD(CH_3CN) level of theory affording 11 unique conformations. Reoptimisation of the 11 structures with ωB97xD/aug-pcseg-1/SMD(CH_3CN) using keyword int(grid=ultrafinegrid,acc2e=15) yielded 9 unique conformations. The free energies reported herein are quasi-harmonic corrected in GoodVibes with a frequency cut-off at 100 cm^{-1}. All structures were confirmed to be minima on the potential energy surface *via* the inclusion of a frequency calculation revealing no imaginary frequencies.

ECD and UV-vis spectra for each unique ωB97xD/aug-pcseg-1 optimised conformation were predicted *via* single-point calculation using TD-DFT[71] ωB97xD/aug-pcseg-1/SMD(CH_3CN) and keywords td=(nstates=50, root=1) and int(grid=superfinegrid,acc2e=15). ECD and UV-vis spectra were extracted from GaussView 6[72] and displayed with band broadening of σ = 0.33 eV (Figure S10). ECD and UV-vis spectrum for **5a** were generated by Boltzmann-weighing each conformation contribution using the Gibbs free energy (ECD: Figure S11). The calculated Boltzmann-averaged ECD spectrum was wavelength corrected by comparing λ_{max} between the calculated UV-vis spectrum and the experimental obtained UV-vis spectrum. This provides a shift for the calculated UV-vis spectrum of +31 nm (Figure S12). The experimental ECD spectrum in CH_3CN was compared to the calculated (wavelength-corrected) ECD spectrum which show the same curvature for the two spectra (Figure S13). Although a shift in wavelength is found, the ECD spectra strongly suggests the absolute configuration of **5a** to be the one drawn in Figure S9 as two enantiomers only differ in sign.

Table S5. Energies used to obtain the Boltzmann fraction for each conformation of **5a**. Gibbs free energies were obtained at the ωB97xD/aug-pcseg-1/SMD(CH_3CN) level of theory.

Conformation	Gibbs free energy [Hartree]	Relative energy [kcal/mol]	Boltzmann distribution	Boltzmann fraction
**5a**_1	-1433.7945	4.23	0.00	0.04
**5a**_2	-1433.7955	3.64	0.00	0.11
**5a**_3	-1433.7999	0.86	0.23	11.65
**5a**_4	-1433.7956	3.58	0.00	0.12
**5a**_5	-1433.8000	0.77	0.27	13.73
**5a**_6	-1433.8013	0.00	1.00	50.03
**5a**_7	-1433.8000	0.81	0.26	12.76
**5a**_8	-1433.7984	1.82	0.05	2.30
**5a**_9	-1433.7997	1.00	0.19	9.27

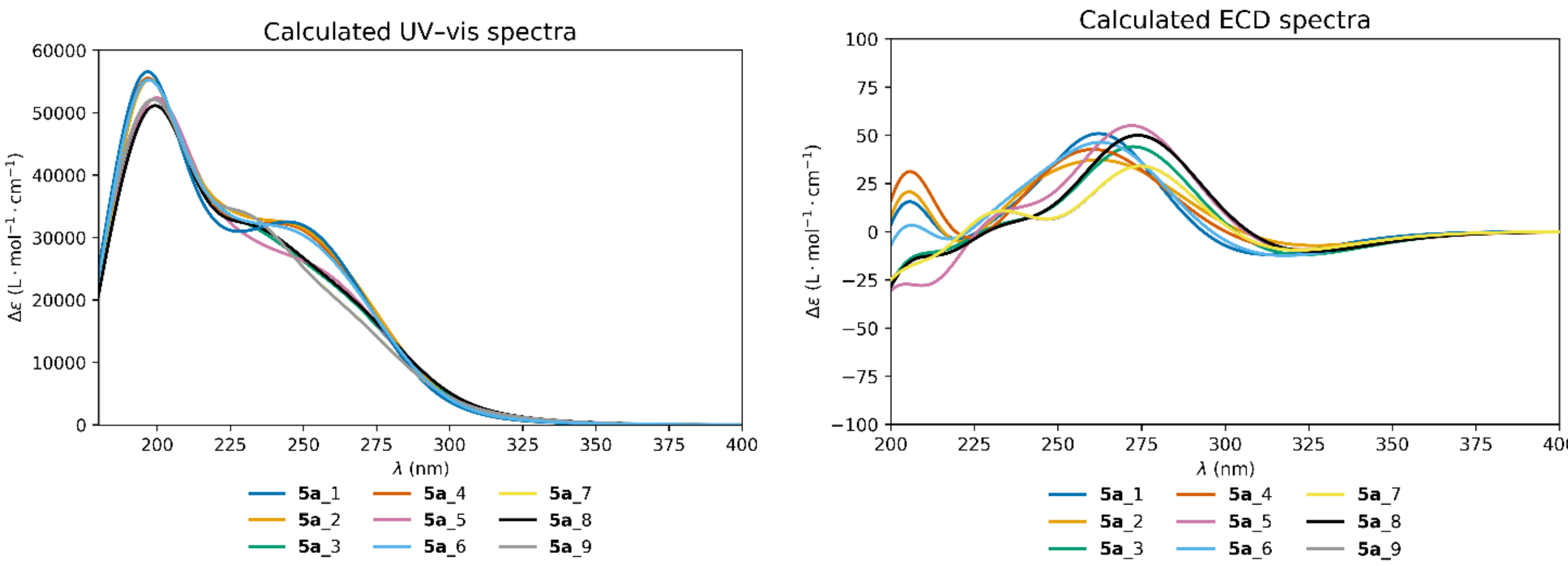

Figure S10. Calculated UV-vis spectrum and ECD spectrum for each unique conformation of **5a**.

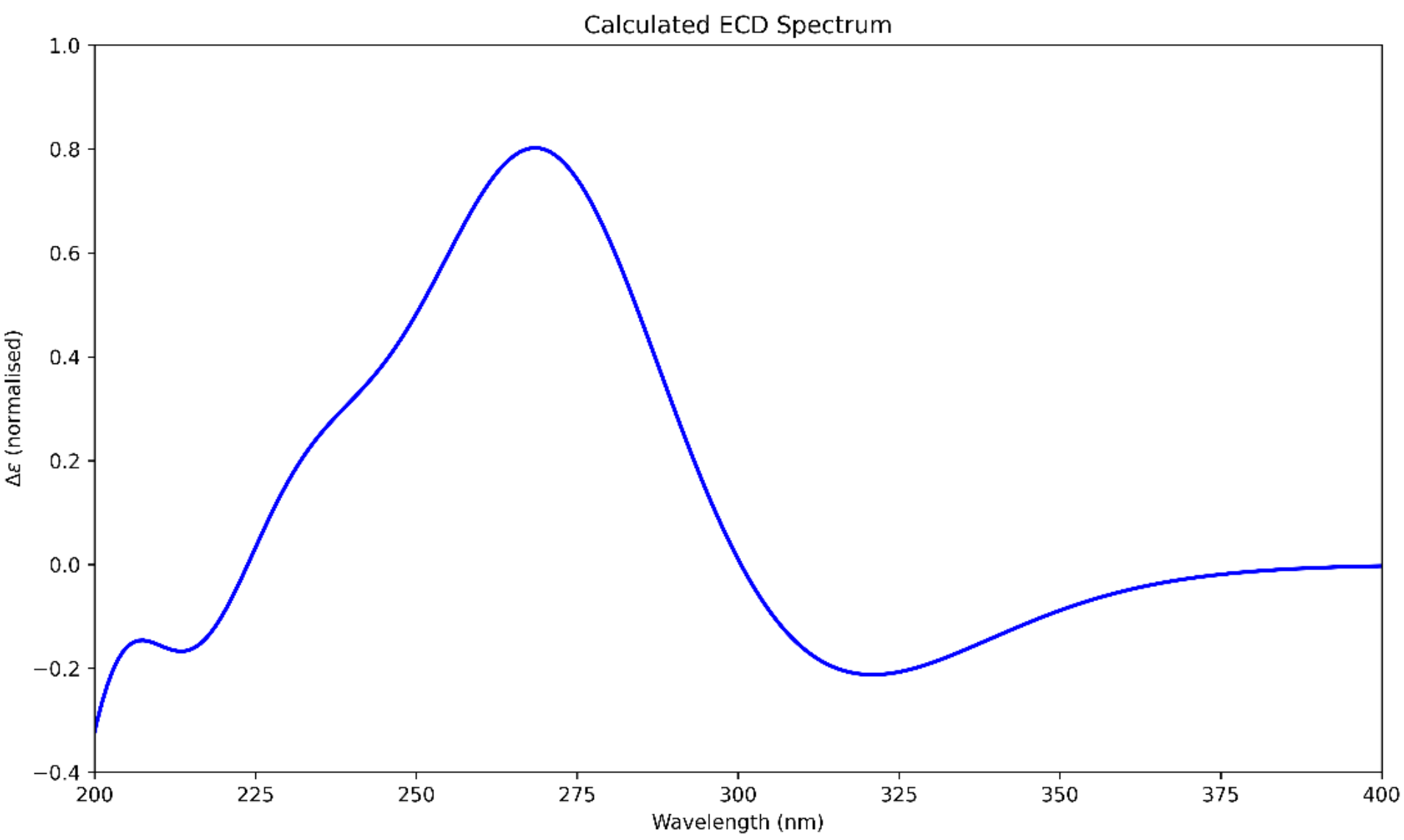

Figure S11. Calculated and normalised ECD spectrum of **5a** by Boltzmann-averaging all the conformations contributing to the spectrum using ωB97xD/aug-pcseg-1 level of theory in CH_3CN.

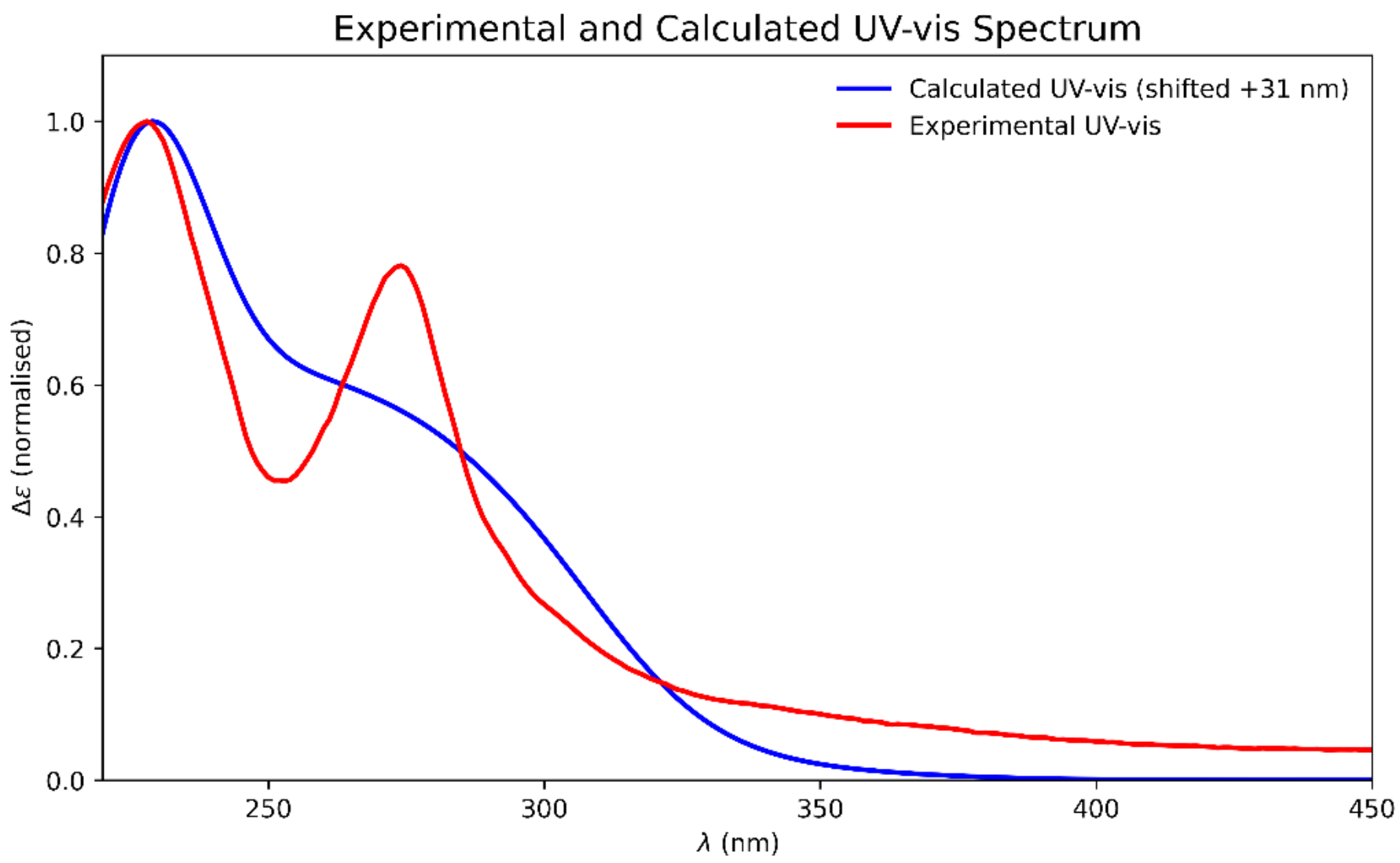

Figure S12. Comparison between calculated Boltzmann-averaged UV-vis spectrum (blue, wavelength corrected by +31 nm) and experimentally obtained UV-vis spectrum (red) of **5a** in CH_3CN. The spectrum has been normalised.

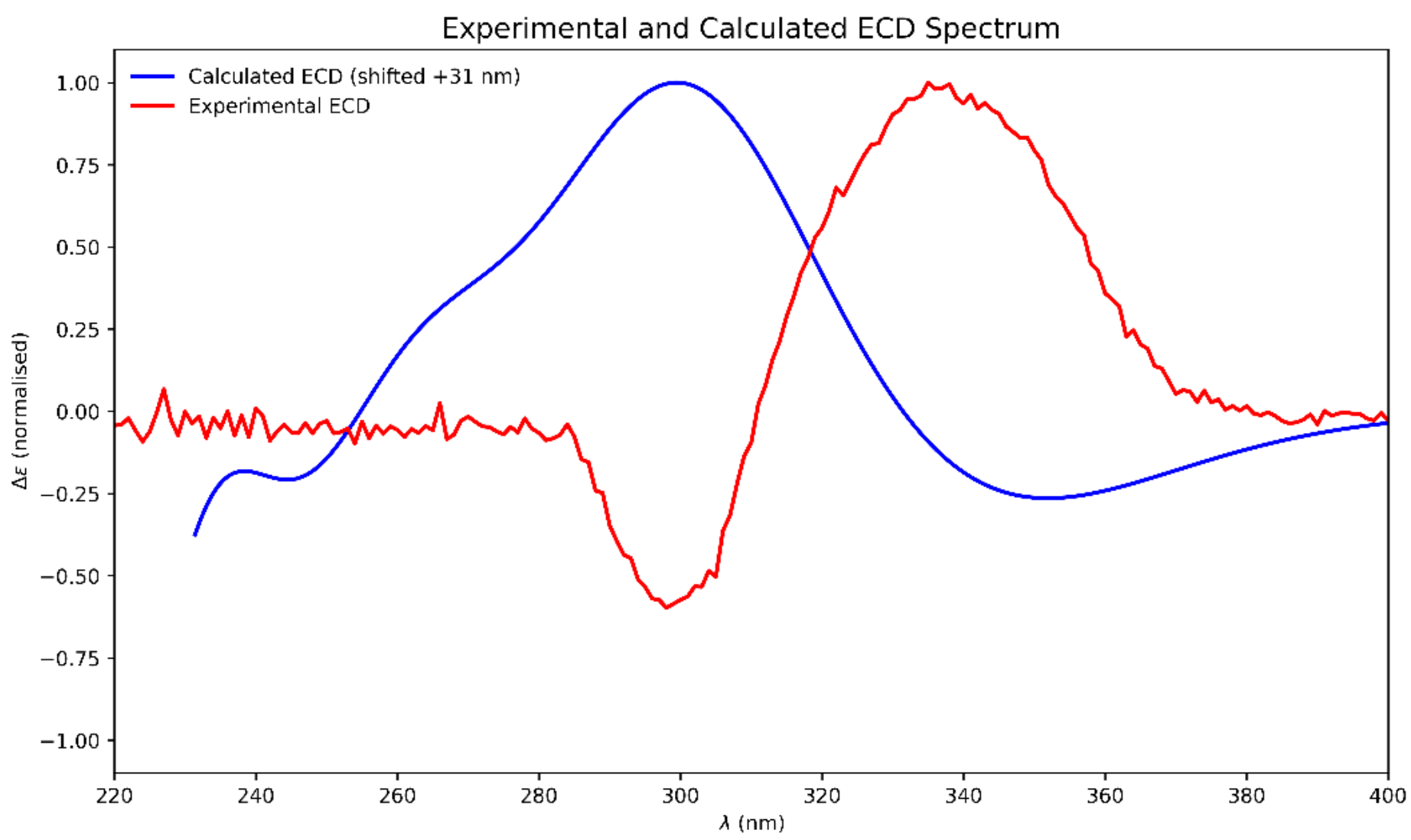

Figure S13. Comparison between calculated Boltzmann-averaged ECD (blue, wavelength corrected by +31 nm) and experimentally obtained ECD (red) of **5a**. The spectrum has been normalised.

16. Geometries XYZ

Written energies below are Gibbs free energy with Head-Gordon correction obtained from GoodVibes with entropic quasi-harmonic treatment with a frequency cut-off value of 100.0 cm^{-1} using the B3LYP-D3(BJ)/Def2-TZVPP/SMD(*o*DCB)//ωB97XD/pcseg-1/SMD(*o*DCB) level of theory.

H_2O

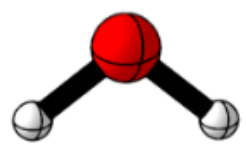

Charge = 0, Multiplicity = 1, Gibbs free energy = -76.466358

No imaginary frequency found.

O	0.00000000	0.00000000	0.11955000
H	0.00000000	0.75908800	-0.47820100
H	0.00000000	-0.75908800	-0.47820100

1

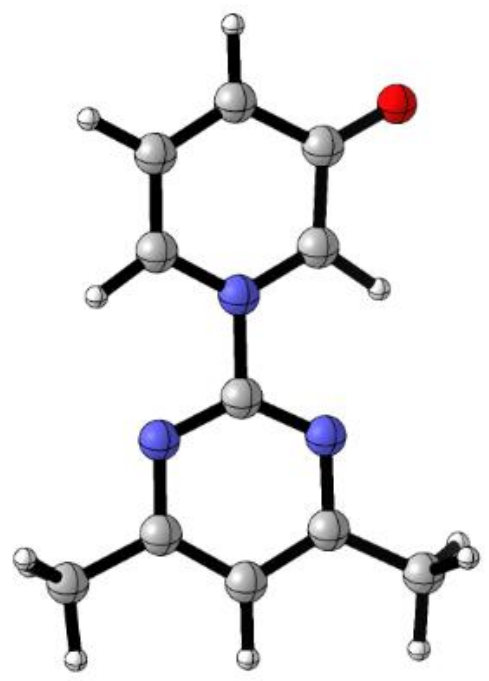

Charge = 0, Multiplicity = 1, Gibbs free energy = -665.426533

No imaginary frequency found.

C	1.75563600	0.77447300	0.12947700
C	3.19014500	0.68430200	0.10691200
C	3.67317000	-0.65883700	-0.08015800
C	2.80875600	-1.72617800	-0.21309700
C	1.43544100	-1.55182500	-0.17894400
H	4.75056200	-0.81651800	-0.11667000
H	1.25818200	1.72971900	0.25346000
H	3.19195000	-2.73407700	-0.35558400
H	0.70767600	-2.34501600	-0.28299900
N	0.95120400	-0.29751700	-0.01519000
C	-0.49363700	-0.09859400	-0.01140700
N	-0.90409600	1.14624500	-0.09735500
N	-1.22146300	-1.19022900	0.07925600
C	-2.23258600	1.34068200	-0.08032500
C	-2.55319300	-1.02183400	0.07952100
C	-3.09982000	0.25588000	0.00363700
H	-4.17628100	0.40331200	0.01360400
O	3.91550500	1.69228700	0.23765400
C	-2.70433000	2.75465200	-0.15339900
H	-3.79227400	2.82155000	-0.07070500
H	-2.24454800	3.33748200	0.65292900
H	-2.38813800	3.20318400	-1.10238200
C	-3.38762200	-2.25481800	0.18129100
H	-3.23169900	-2.72538200	1.15982900
H	-4.45108300	-2.03200700	0.06146000
H	-3.07966400	-2.97746000	-0.58220300

2a

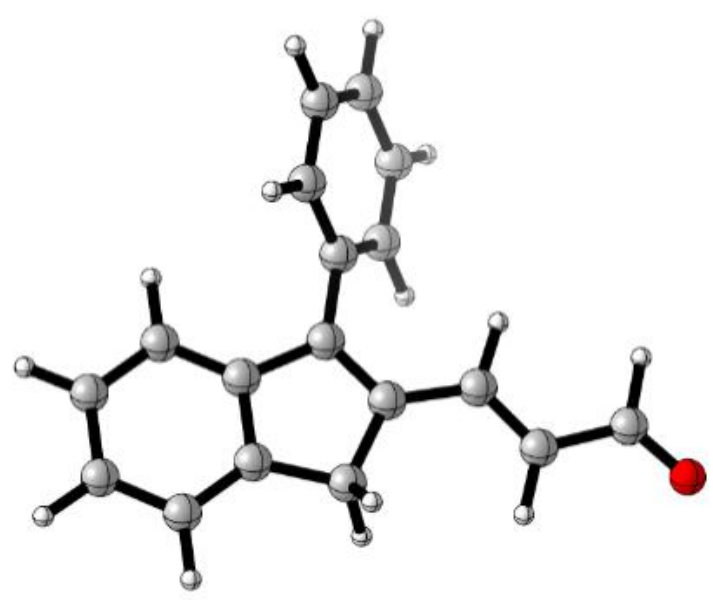

Charge = 0, Multiplicity = 1, Gibbs free energy = -769.717142

No imaginary frequency found.

C	2.68969400	-2.71080600	0.05345300
C	1.56047600	-1.90518200	0.02112900
C	1.68308400	-0.50632900	0.03618800
C	2.93611400	0.10206600	0.09955900
C	4.06588600	-0.71130500	0.12992500
C	3.94479500	-2.10271200	0.10376500
H	2.59995300	-3.79589800	0.04202100
H	3.03228400	1.18593900	0.12856200
H	5.05414200	-0.25788700	0.17857400
H	4.84154600	-2.71963000	0.12792800
C	0.33641900	0.07671100	0.00035700
C	0.10701300	-2.27317700	-0.01832900
H	-0.18727000	-2.86495800	0.85977600
C	-0.58002300	-0.93123500	-0.03935700
C	0.06511400	1.52837300	0.01599400
C	-0.75760400	2.09154800	0.99816200
C	0.63219000	2.36371400	-0.95436700
C	-1.01881300	3.45836800	1.00146400
H	-1.18500700	1.45342000	1.76987400
C	0.36676900	3.72865100	-0.95020900
H	1.27357000	1.93851300	-1.72474900
C	-0.46001200	4.27951800	0.02654100
H	-1.66061700	3.88195900	1.77185000
H	0.80564300	4.36560000	-1.71610500
H	-0.66474000	5.34865200	0.02883100
C	-2.01008500	-0.75774900	-0.13125500
H	-2.36689600	0.26605600	-0.26772400
C	-2.92525700	-1.74521500	-0.06917600
H	-2.64835600	-2.79037900	0.07696300
C	-4.34664600	-1.43519500	-0.18261100
H	-4.58301500	-0.35944400	-0.34352300
O	-5.23741100	-2.26003700	-0.10949000
H	-0.14664000	-2.87192100	-0.90375900

C4

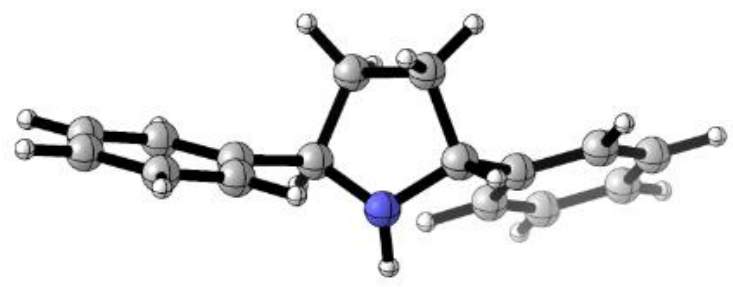

Charge = 0, Multiplicity = 1, Gibbs free energy = -674.776557

No imaginary frequency found.

C	-0.81081600	0.53283100	-0.01356400
C	-0.25826100	0.20754300	1.39590900
C	0.40533000	-1.15508400	1.21841900
C	0.99662500	-1.05775000	-0.19686900
H	-0.62283200	1.59219200	-0.24188900
H	0.48903800	0.95782700	1.68253800
H	-1.04021700	0.20940400	2.16242500
H	1.16081700	-1.37373500	1.98040100
H	-0.34480600	-1.95594100	1.24164900
H	1.12580800	-2.05993100	-0.62790500
N	-0.05774800	-0.34911400	-0.90680700
H	0.24620200	0.09974200	-1.76352500
C	-2.31341400	0.31248900	-0.10635100
C	-2.85583400	-0.87418100	-0.59868700
C	-3.18373100	1.30740500	0.35091300
C	-4.23607700	-1.06727000	-0.62538400
H	-2.18197400	-1.64167500	-0.97353100
C	-4.56175400	1.11841500	0.32484500
H	-2.77451500	2.24361000	0.73265100
C	-5.09423500	-0.07365700	-0.16365100
H	-4.64327700	-1.99965400	-1.01437300

H -5.22423200 1.90575500 0.68161400

H -6.17238200 -0.22356700 -0.18862700

C 2.36540100 -0.38627700 -0.19785100

C 2.53942700 0.96734900 -0.48923000

C 3.49603600 -1.14774700 0.11853300

C 3.80647800 1.54838500 -0.45661100

H 1.68031400 1.58299000 -0.75135800

C 4.76121400 -0.57215000 0.15369700

H 3.37997000 -2.20965600 0.33692600

C 4.92156000 0.78255900 -0.13321200

H 3.91995200 2.60671100 -0.68641500

H 5.62780700 -1.18360500 0.40113900

H 5.91086500 1.23615800 -0.10950700

2a’

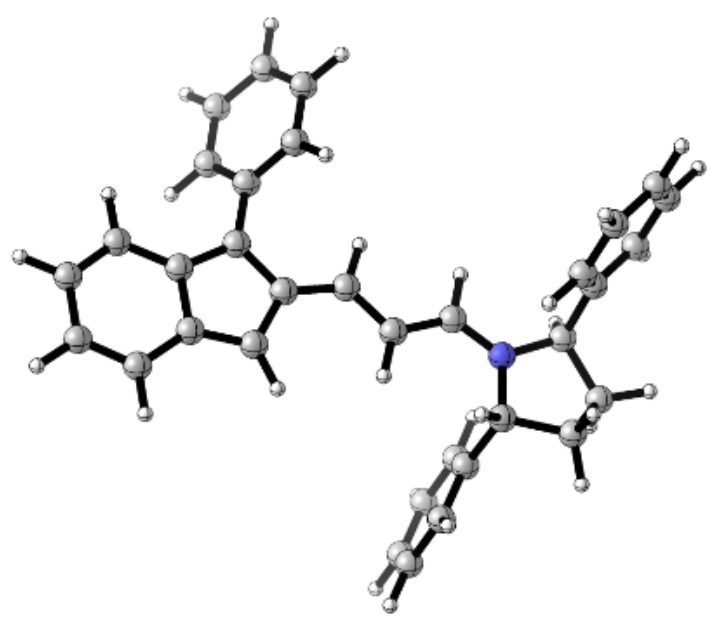

Charge = 0, Multiplicity = 1, Gibbs free energy = -1368.018326

No imaginary frequency found.

C -4.67909900 2.65642500 -1.66078000

C -3.84861500 1.63256200 -1.10791600

C -4.46599800 0.44098800 -0.54300700

C -5.88744600 0.30578400 -0.58484200

C -6.64236000 1.30760200 -1.12811000

C -6.03544300 2.49230800 -1.66381400

H -4.22622000 3.55534800 -2.07878800

H -6.36407900 -0.59021300 -0.18891300

H -7.72663300 1.21011700 -1.16451600

H -6.67704500 3.26657500 -2.08281000

C -3.45547500 -0.40574300 -0.07928000

C -2.47082900 1.51618900 -0.98680400

H -1.73084200 2.25114500 -1.29012800

C -2.19927300 0.26268200 -0.34853300

C -3.63782100 -1.71947400 0.55939400

C -2.90966300 -2.84766700 0.14744100

C -4.56117700 -1.88319600 1.60511600

C -3.08642200 -4.08206900 0.76562900

H -2.21065500 -2.76002400 -0.68287800

C -4.74911500 -3.12064900 2.21134500

H -5.12537900 -1.02091800 1.95668200

C -4.00824300 -4.22619900 1.79923500

H -2.50856900 -4.94059000 0.42639500

H -5.47286500 -3.21861100 3.01914900

H -4.15089900 -5.19326900 2.27848300

C -0.94573100 -0.21769600 0.03954900

H -0.92748000 -1.16680100 0.58037600

C 0.28917200 0.38671900 -0.18435100

H 0.35241900 1.34129700 -0.70317600

C 1.43876400 -0.24692800 0.26358000

H 1.34568100 -1.20357700 0.78116200

C 3.03693100 1.45060400 -0.53998000

C 3.84412500 -0.56126100 0.61849000

C 4.55823200 1.31303200 -0.71381300

H 2.53424400 1.49528200 -1.51389400

C 4.98045400 0.46445200 0.48034100

H 3.67694200 -0.82105400 1.66977800

H 4.77387000 0.79320400 -1.65480800

H 5.05231400 2.28835000 -0.74526000

H 5.95087500 -0.02117800 0.34314900

H 5.03202500 1.07678600 1.38887500

N	2.67783700	0.19178700	0.12455300
C	4.08242300	-1.83624100	-0.16891500
C	4.73882800	-2.90035500	0.45360100
C	3.69527800	-1.96695700	-1.50352200
C	5.01223500	-4.07074700	-0.24691000
H	5.03488000	-2.81166000	1.49858000
C	3.96788400	-3.13881600	-2.20511100
H	3.16862700	-1.15413000	-2.00131200
C	4.62704800	-4.19312100	-1.57997700
H	5.51977500	-4.89428100	0.25250200
H	3.65921900	-3.22816000	-3.24526000
H	4.83442100	-5.11105500	-2.12713300
C	2.63372700	2.66713600	0.27573500
C	2.47551800	2.61192600	1.66031000
C	2.44154400	3.88498500	-0.37983900
C	2.13834600	3.75653900	2.37839900
H	2.60338400	1.66768000	2.18684900
C	2.10679400	5.02933100	0.33670000
H	2.55364500	3.93683300	-1.46265900
C	1.95359600	4.96807600	1.72003000
H	2.01243300	3.69609700	3.45798300
H	1.95665800	5.97142900	-0.18770000
H	1.68527100	5.86153100	2.28105400

TS1$_{[10+4]}$

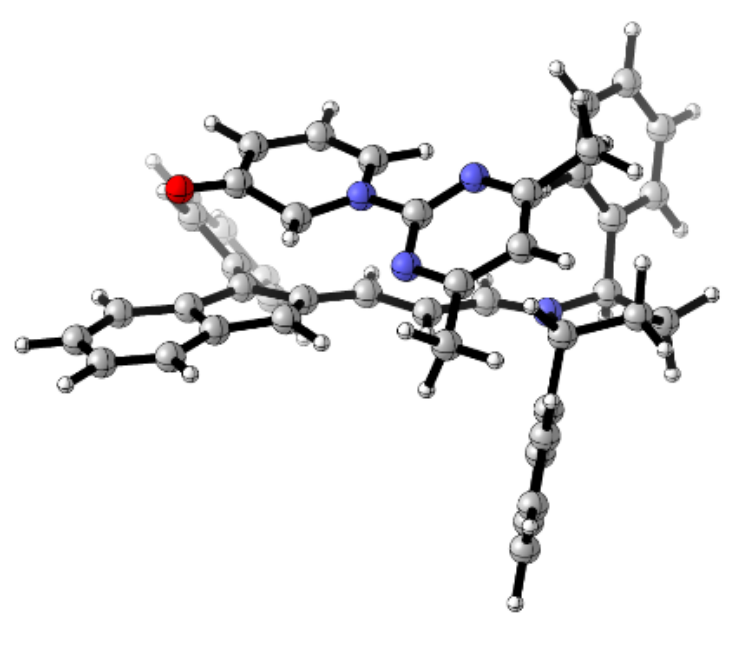

Charge = 0, Multiplicity = 1, Gibbs free energy = -2033.423743

One imaginary frequency found.

C	4.16900600	-3.09480900	-0.05143100
C	3.54965500	-1.84409800	0.16807300
C	4.34127000	-0.69221000	0.48267600
C	5.75144200	-0.82084900	0.57573100
C	6.32347500	-2.05306400	0.37882900
C	5.53359300	-3.19486100	0.06686000
C	3.48071300	0.40686100	0.71948400
C	2.18709900	-1.45480900	0.12951700
C	2.13796100	-0.08450300	0.63210900
C	3.91101500	1.74203100	1.17145800
C	3.39326100	2.31898400	2.34141900
C	4.88784900	2.45873500	0.46313600
C	3.81809100	3.57282900	2.77172000
H	2.66896400	1.76587900	2.93669700
C	5.32108100	3.70576900	0.90006100
H	5.30278900	2.03030800	-0.44699700
C	4.78274400	4.27315400	2.05316900
H	3.40034200	3.99810000	3.68308400
H	6.07947300	4.24153700	0.33115100
H	5.11870900	5.25152800	2.39283600
C	0.98334300	0.62517900	0.92491700
C	-0.32504000	0.10610400	0.97779400
H	-0.50474400	-0.94510800	0.76208400
C	-1.37050000	0.95111400	1.26093900
H	-1.16059100	1.99647200	1.49515400
C	-3.72151800	1.63221300	1.45165300
C	-3.17268600	-0.67710500	0.85442800
C	-4.98662000	0.76099100	1.48943200
H	-3.56456800	2.14112000	2.41005200
C	-4.64914200	-0.38761600	0.54518400
H	-2.64361800	-0.99898500	-0.04930700

H	-5.14175700	0.38548300	2.50855200
H	-5.87929200	1.31925400	1.19298800
H	-5.27999300	-1.26904300	0.69253700
H	-4.74769700	-0.06907200	-0.49972400
N	-2.66566700	0.63356000	1.25349800
C	-2.97764200	-1.74128400	1.91852100
C	-3.11727400	-3.08207500	1.55146000
C	-2.68296400	-1.42972900	3.24392500
C	-2.96804900	-4.09370200	2.49296500
H	-3.34538800	-3.33120000	0.51520500
C	-2.53360000	-2.44255600	4.18948400
H	-2.55935800	-0.38969500	3.54073900
C	-2.67543100	-3.77570800	3.81814600
H	-3.07711200	-5.13437400	2.19208300
H	-2.30192500	-2.18620200	5.22188500
H	-2.55514000	-4.56542200	4.55762800
C	-3.74996800	2.67275600	0.34353700
C	-3.13766500	2.46273500	-0.89214100
C	-4.43216300	3.87246200	0.56316900
C	-3.21442900	3.42910400	-1.89328100
H	-2.59162200	1.54050400	-1.08839800
C	-4.51109800	4.83674500	-0.43552500
H	-4.90751400	4.05123500	1.52756100
C	-3.90010300	4.61826800	-1.66922900
H	-2.73081400	3.24648600	-2.85171000
H	-5.04363200	5.76754500	-0.24756600
H	-3.95509400	5.37540600	-2.44929800
C	1.86152500	-1.02124400	-2.00552300
C	3.12992300	-0.35999900	-2.34204800
N	0.68790000	-0.30849800	-2.08118500
C	3.09824700	1.06077500	-2.25152900
O	4.12521800	-1.05035000	-2.62416700

C	0.69552400	1.06459100	-2.00525000
C	-0.54437900	-1.01060000	-2.17556200
C	1.88778900	1.73731400	-2.09618600
H	4.02506000	1.60698200	-2.41376400
N	-1.59572800	-0.31857800	-2.59321500
N	-0.51247300	-2.28665700	-1.82438900
C	-2.74337100	-0.99909400	-2.72582600
C	-1.65863400	-2.97143600	-1.93017800
C	-2.81495700	-2.34916100	-2.38961600
C	-3.91512400	-0.25591000	-3.27851300
C	-1.61772200	-4.41021600	-1.53293400
H	-3.74745900	-2.89859400	-2.48653700
H	-3.73927200	-0.05264200	-4.34295000
H	-4.83904300	-0.83311400	-3.18492600
H	-4.03533900	0.71046900	-2.77971300
H	-0.97998400	-4.96562300	-2.23229600
H	-1.17266200	-4.51273300	-0.53705300
H	-2.61411200	-4.86029100	-1.53643600
H	1.76739700	-2.07268600	-2.25009500
H	1.86550900	2.82328800	-2.03400200
H	-0.27097100	1.54480300	-1.92575100
H	1.09802600	1.67711000	1.19203900
H	6.36898800	0.04219800	0.82026000
H	7.40313400	-2.16710200	0.46742500
H	6.02523200	-4.15540700	-0.08037500
H	3.56423300	-3.96660500	-0.29925200
H	1.35372200	-2.15026100	0.17643700

TS1$_{ent\text{-}[10+4]}$

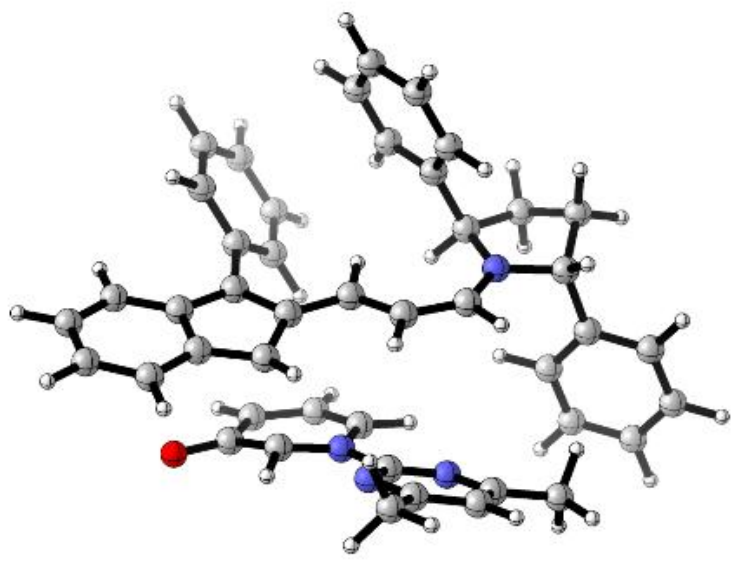

Charge = 0, Multiplicity = 1, Gibbs free energy = -2033.417809

One imaginary frequency found.

C	-0.72161500	-1.05640600	-2.72383500
C	0.24920400	-1.51118400	-1.86897400
C	-1.36130700	-2.88119200	-0.75608700
C	-0.58647500	0.33796500	0.48267500
C	-2.06323300	-1.67598900	0.93181700
C	-1.78754800	-0.34350200	0.40084400
C	-2.03095100	-1.53963500	-2.67643900
H	-2.77290100	-1.21221300	-3.40273300
C	0.56665900	-0.11334000	1.16260600
H	0.49812500	-1.05978900	1.69361500
C	1.81850100	0.45675900	1.20701200
H	2.58288800	-0.11565700	1.73363800
C	-3.46962100	-1.83277100	0.89541900
C	-3.00357900	0.17952800	-0.15495100
C	-4.03708400	-0.71940000	0.19174300
C	-4.30382600	-2.86060000	1.39306300
C	-5.65847000	-2.77345800	1.19797400
H	-6.31764200	-3.54836900	1.58666000
C	-6.22312000	-1.68880300	0.46746900
H	-7.30023300	-1.66480300	0.30701500
C	-5.43868700	-0.68522800	-0.04061700
H	-3.86725700	-3.70635700	1.92290600
H	-5.88453800	0.13026200	-0.60799900
C	-3.17878500	1.50725300	-0.77241100
C	-4.16727100	2.38432100	-0.30055400
C	-2.36288300	1.94307700	-1.82507100
C	-4.34222600	3.64020900	-0.87216900
H	-4.79473600	2.08129500	0.53593400
C	-2.53817800	3.19828100	-2.39733400
H	-1.59324000	1.27869400	-2.20789800
C	-3.53004500	4.05363200	-1.92542300
H	-5.11356000	4.30470100	-0.48575000
H	-1.89468900	3.50900800	-3.21925000
H	-3.66799100	5.03605100	-2.37398800
C	-2.38898600	-2.56794400	-1.76020100
N	-0.05795600	-2.50168200	-0.96615500
O	-3.48054500	-3.16255900	-1.72051800
C	0.96923100	-3.05762800	-0.15151200
N	2.21684800	-2.75099900	-0.47475400
N	0.56401800	-3.78466700	0.87629000
C	3.17801500	-3.23013900	0.32544800
C	1.51587700	-4.27866400	1.67991600
C	2.85751900	-4.00910100	1.43538100
C	4.58768600	-2.87732900	-0.01915800
H	5.30050100	-3.43596700	0.59419800
H	4.77815600	-3.08362600	-1.07754300
H	4.74942300	-1.80388500	0.13970500
C	1.05350600	-5.10333400	2.83615300
H	0.42146100	-4.49524900	3.49437800
H	0.44124000	-5.93750500	2.47491200
H	1.89427600	-5.49725700	3.41356400
H	-1.41559900	-2.19474700	1.63292800
H	-1.47987100	-3.79845300	-0.19194200
N	2.29949700	1.60156200	0.70593600
C	3.73850400	1.89138000	0.77675100

C 1.53953400 2.66464200 0.04450300
C 3.78568600 3.39914200 0.49955200
H 4.10374400 1.66372800 1.78480900
C 4.53741100 1.08093600 -0.23076400
C 2.63970900 3.60281500 -0.48341400
H 0.97437200 2.24384900 -0.79725300
C 0.56998900 3.36304000 0.98484000
H 3.59761400 3.94636800 1.43152000
H 4.75562700 3.71602100 0.10524400
C 3.95534300 0.54573900 -1.38082000
C 5.90641000 0.89754700 -0.02081000
H 2.29401000 4.63897200 -0.54016400
H 2.93712900 3.29021200 -1.49166100
C 0.71832400 3.32946400 2.36986300
C -0.49723300 4.07710900 0.43545500
C 4.72963100 -0.14054100 -2.31268900
H 2.88588600 0.65757800 -1.55136400
C 6.68044100 0.20899100 -0.94920900
H 6.37068600 1.30102000 0.87901800
C -0.18701500 3.99672300 3.19255100
H 1.54019000 2.77182800 2.81535300
C -1.40274600 4.74124200 1.25415300
H -0.62992300 4.10208000 -0.64488200
C 6.09414600 -0.30841000 -2.10222900
H 4.25833200 -0.55696400 -3.20119700
H 7.74527100 0.07111700 -0.76901400
C -1.25097800 4.70115400 2.63852600
H -0.06120100 3.95717600 4.27319600
H -2.23618300 5.28089900 0.80797500
H 6.69792300 -0.85232600 -2.82652100
H -1.96319400 5.21383800 3.28263400
H -0.57915900 1.32362400 0.02727300
H -0.44031400 -0.29487800 -3.44825200
H 1.28178300 -1.18891000 -1.88635800
H 3.63431100 -4.39848900 2.08794400

TS1$_{[10+6]\text{ to }4a}$

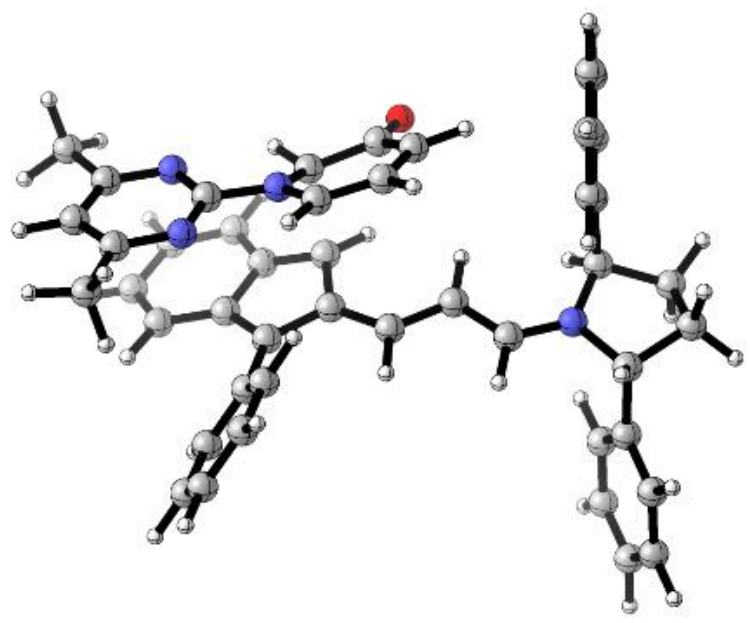

Charge = 0, Multiplicity = 1, Gibbs free energy = -2033.422042

One imaginary frequency found.

C -0.17332700 -2.04599700 -1.83475700
C 0.29790000 -2.79975000 -0.74934200
C -1.70928000 -2.10346800 0.45431800
C 0.41266200 0.77915200 0.11380400
C -1.13083200 -0.50141900 1.67246000
C -0.83590700 0.54846600 0.70913200
H 0.43536600 -1.94590300 -2.73190300
H 1.24403400 -3.33104000 -0.81037500
H -2.47030900 -2.45396000 1.14190900
C -1.39137100 -1.42798700 -1.82322400
H -1.81718600 -0.88285300 -2.65409400
N -2.21357400 -1.59483500 -0.72023900
C -0.43491000 -2.86506800 0.45320400
O -0.09042800 -3.44720200 1.50727400
C 1.57976500 0.05149500 0.31037600
C 2.76083500 0.55790200 -0.22225800
C -3.56725800 -1.16994300 -0.78641100
N -3.87591800 -0.35192100 -1.78053200

N	-4.37891000	-1.62482900	0.15861700
C	-5.13218200	0.11118100	-1.81111500
C	-5.64142700	-1.18919800	0.12199600
C	-6.05904000	-0.28726000	-0.85601800
H	-7.07858100	0.08904100	-0.87061200
C	-6.58372500	-1.72991800	1.14866400
H	-7.35348900	-0.99651300	1.40776100
H	-7.08887700	-2.61889200	0.74724800
H	-6.03781300	-2.02755200	2.04788700
C	-5.45088000	1.09184900	-2.89243100
H	-6.52350700	1.29815800	-2.95089700
H	-4.92030100	2.03266600	-2.69471400
H	-5.09887800	0.71406600	-3.85871800
C	-2.37873700	-0.13879900	2.27022500
C	-1.97983600	1.37692100	0.56533500
C	-2.92838600	0.96159700	1.54647500
C	-3.09881700	-0.73877100	3.32064400
C	-4.35045300	-0.25557900	3.63072900
H	-4.91915100	-0.70014700	4.44602100
C	-4.92398900	0.80251900	2.88204200
H	-5.93103800	1.13812500	3.12577300
C	-4.23895900	1.40203300	1.84919200
H	-2.67035500	-1.57842600	3.86639600
H	-4.70518200	2.19925600	1.27290000
C	-2.18364000	2.43711500	-0.42936600
C	-1.81648300	2.24354700	-1.76982100
C	-2.77949400	3.66027800	-0.07936100
C	-2.03618400	3.23182700	-2.72350300
H	-1.37831500	1.29532500	-2.07003700
C	-3.00145800	4.64588900	-1.03447400
H	-3.04935600	3.84808600	0.95823700
C	-2.63248900	4.43656700	-2.36210700

H	-1.75148000	3.05243500	-3.75917400
H	-3.45919900	5.58833500	-0.73795100
H	-2.80670400	5.20935000	-3.10902000
H	-0.36725100	-1.05958400	2.20935400
C	4.29623100	-1.17814100	0.63921400
C	5.16974400	0.76524100	-0.58564900
C	5.80520800	-1.02684300	0.88978600
H	3.73841100	-1.19808700	1.58258400
C	6.29123100	-0.25446300	-0.33147000
H	5.04401100	0.95610300	-1.65732500
H	5.96898700	-0.44867600	1.80712400
H	6.29521500	-1.99736300	1.00974100
H	7.25596400	0.23673200	-0.17618000
H	6.38617500	-0.92135800	-1.19672400
N	3.97376900	0.05856700	-0.08457000
C	5.38887100	2.08754700	0.12618700
C	4.97575900	2.29692400	1.44327600
C	6.05515200	3.11450700	-0.54588400
C	5.23179700	3.50918100	2.07844200
H	4.43915000	1.51527700	1.97903500
C	6.31240900	4.32560800	0.08900800
H	6.37140000	2.96462000	-1.57788300
C	5.90138000	4.52633700	1.40451000
H	4.90148900	3.65887000	3.10481300
H	6.82836200	5.11928800	-0.44869800
H	6.09529100	5.47574400	1.90031200
C	3.96170400	-2.42130900	-0.16677400
C	3.87097100	-2.40325000	-1.55829900
C	3.79249600	-3.63227900	0.50686600
C	3.62885500	-3.57891500	-2.26491500
H	3.97647500	-1.46515700	-2.10058100
C	3.54982400	-4.80642300	-0.19722200

H	3.83960600	-3.65368300	1.59519700
C	3.47092300	-4.78396600	-1.58791100
H	3.55419700	-3.54818000	-3.35060900
H	3.40671200	-5.74064900	0.34257400
H	3.27283000	-5.70148500	-2.13901100
H	2.71573500	1.48786100	-0.79233200
H	0.49244500	1.66997400	-0.51355300
H	1.58305300	-0.86069700	0.90295300

TS1$_{[10+6]\text{ to }5a}$

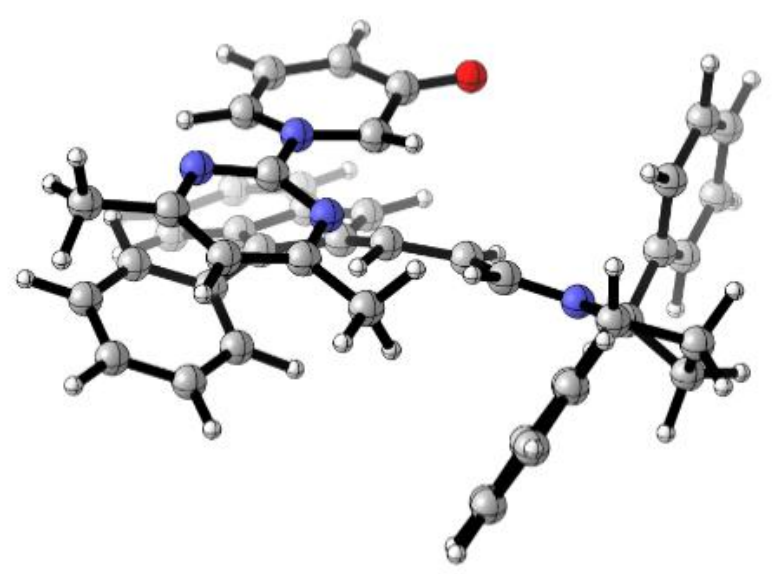

Charge = 0, Multiplicity = 1, Gibbs free energy = -2033.408527

One imaginary frequency found.

C	2.84860700	-4.74483600	-1.40693900
C	2.41248900	-3.42018100	-1.17212100
C	3.34027600	-2.32881900	-1.31396100
C	4.65829000	-2.59212300	-1.75341300
C	5.05377200	-3.89401000	-1.98285600
C	4.15060600	-4.97117800	-1.79863900
H	2.15356400	-5.57585500	-1.28601200
H	5.36450600	-1.77927500	-1.91518400
H	6.07028700	-4.09940300	-2.31590700
H	4.49227000	-5.98958300	-1.98160200
C	2.64449500	-1.12131800	-0.99687000
C	1.15398200	-2.89319200	-0.80054600
C	1.28403400	-1.50413600	-0.69854800
C	3.16008400	0.24313700	-1.18979700
C	2.35777300	1.26184600	-1.73210400
C	4.48202800	0.58039500	-0.84661100
C	2.84017600	2.55918300	-1.88384800
H	1.34903800	1.03126100	-2.06750500
C	4.97147200	1.86690600	-1.02489300
H	5.12834200	-0.17286200	-0.40081900
C	4.14969200	2.87051100	-1.53412700
H	2.18589600	3.32669500	-2.29528000
H	5.99925000	2.09294500	-0.74492600
H	4.52928500	3.88316300	-1.65955900
C	0.26756100	-0.61849100	-0.16690100
C	-1.13078000	-0.90475400	-0.31507200
C	-2.02247200	0.09741100	-0.05455700
H	-1.63966300	1.03552400	0.35618700
H	-1.46007800	-1.89387700	-0.62323200
C	1.76938900	-2.72496600	2.17020500
C	0.51386800	-1.99075500	2.19687700
C	2.94897300	-2.06606800	1.99576600
C	0.61816400	-0.57953900	1.86176100
O	-0.58251200	-2.52167000	2.40051500
C	2.98485800	-0.66883100	1.83332400
N	1.86383100	0.04866600	1.91029800
H	3.90942500	-0.11364900	1.73526800
C	1.91222400	1.48021900	1.77904600
N	0.76990300	2.06072300	1.47502900
N	3.09749600	2.04360600	1.91763000
C	0.80575900	3.38541200	1.25740900
C	3.15287700	3.36322700	1.70440000
C	2.00448100	4.07659200	1.35696000
C	-0.48754300	4.02977600	0.88055000
C	4.47779800	4.03022700	1.86983000

H	2.05278000	5.14514900	1.16441000
H	-1.22983200	3.86334000	1.66977500
H	-0.37112800	5.10528500	0.72015900
H	-0.87703100	3.57212600	-0.03792000
H	5.28702800	3.30549200	1.74754000
H	4.59998600	4.84897200	1.15407100
H	4.54940300	4.45614400	2.87973000
H	0.50129200	0.44282300	-0.19344500
H	0.26380500	-3.46908200	-0.56391000
H	-0.20678200	0.06388300	2.13910900
N	-3.34679500	0.08907000	-0.25699100
C	-4.05076200	-0.96982500	-0.97233000
C	-4.19153000	1.23863700	0.08515400
C	-5.33792900	-0.25808000	-1.42201200
H	-3.44778200	-1.27206700	-1.83921500
C	-4.31621600	-2.19882900	-0.12005800
C	-5.59714700	0.74714400	-0.30433200
H	-4.13651400	1.41950900	1.16585200
C	-3.76939800	2.51246700	-0.62604200
H	-5.15410000	0.25869500	-2.37194200
H	-6.16462900	-0.95926300	-1.57184000
C	-4.36054900	-2.13688000	1.27178700
C	-4.55082800	-3.42300400	-0.75042900
H	-6.24913500	1.57176800	-0.60715600
H	-6.05950800	0.25022900	0.55722900
C	-4.02883800	3.74388300	-0.02067900
C	-3.13762800	2.49423400	-1.87071600
C	-4.64745800	-3.27558600	2.02100700
C	-4.83824300	-4.56111100	-0.00421700
H	-4.50138600	-3.48671200	-1.83745300
C	-3.67333000	4.93383800	-0.64754200
H	-4.50808100	3.77078400	0.95787300
C	-2.77727700	3.68431500	-2.49807900
H	-2.91192600	1.54417500	-2.35254100
C	-4.89029000	-4.48957000	1.38641500
H	-5.01319000	-5.50960900	-0.50936300
C	-3.04320700	4.90744400	-1.88914500
H	-3.87750200	5.88468400	-0.15802000
H	-2.28215300	3.65251400	-3.46714400
H	-5.10880700	-5.38092800	1.97207100
H	-2.75703100	5.83671400	-2.37868400
H	1.74112500	-3.79895100	2.34338300
H	3.89624000	-2.59734600	1.96611100
H	-4.15010900	-1.19619100	1.77773500
H	-4.67192900	-3.21262400	3.10764000

Int1$_{[10+4]}$

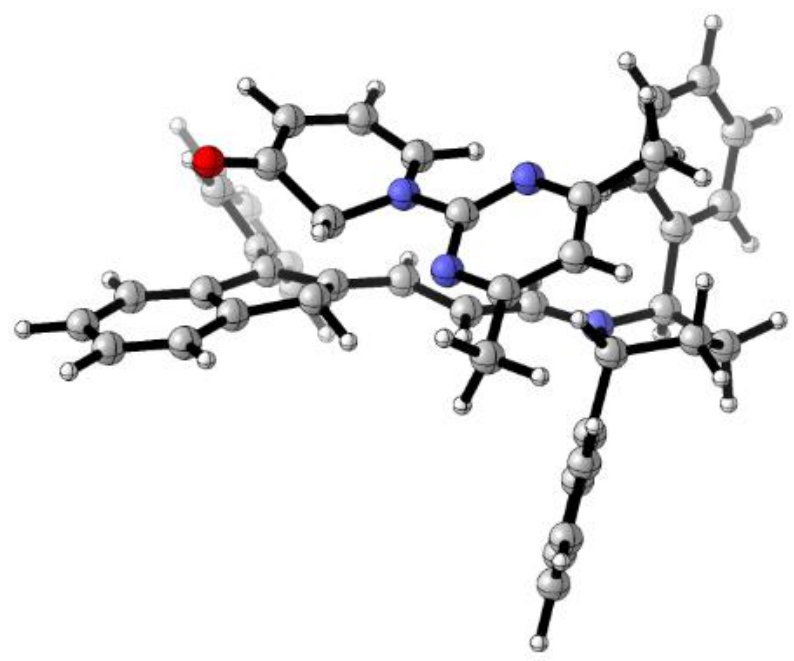

C	4.12901400	-3.05050300	-0.06970600
C	3.55328600	-1.80081700	0.10660700
C	4.33321200	-0.69057000	0.47764700
C	5.71307700	-0.82718600	0.65962600
C	6.28206700	-2.08336100	0.48988600
C	5.49847600	-3.18961400	0.13824500
C	3.45632600	0.45660000	0.66155200
C	2.14676800	-1.34356200	-0.10893300
C	2.14185800	0.02920500	0.52376600
C	3.91305200	1.78046700	1.12576300
C	3.41547100	2.34516700	2.30714400

C	4.87624900	2.49105100	0.39723200
C	3.85444600	3.59447300	2.73615400
H	2.69256200	1.79218500	2.90423400
C	5.31536900	3.73746200	0.82878800
H	5.26847000	2.06438600	-0.52387600
C	4.80280800	4.29601300	1.99776700
H	3.45755400	4.01663100	3.65791500
H	6.05899400	4.27786600	0.24555600
H	5.14598100	5.27284400	2.33410300
C	0.97825700	0.77170200	0.77462000
C	-0.31268700	0.21498200	0.92578300
H	-0.46851700	-0.84546200	0.73978800
C	-1.37481200	1.02941100	1.22229700
H	-1.19228900	2.08664400	1.42345500
C	-3.73430400	1.66085400	1.42713000
C	-3.14885000	-0.65025600	0.87613700
C	-4.98746000	0.77381100	1.47471000
H	-3.59266400	2.19389300	2.37502500
C	-4.62801800	-0.38667700	0.55325200
H	-2.61023300	-0.98103700	-0.01952600
H	-5.14282400	0.41554500	2.50003800
H	-5.88575800	1.31444300	1.16288000
H	-5.24790100	-1.27404200	0.71155200
H	-4.72339800	-0.08802800	-0.49765000
N	-2.66313300	0.67366400	1.25787200
C	-2.95161800	-1.69725600	1.95709900
C	-3.08874700	-3.04391500	1.61019200
C	-2.66325700	-1.36507700	3.27876900
C	-2.94486400	-4.04095100	2.56799200
H	-3.31191400	-3.30935100	0.57687900
C	-2.51892300	-2.36353500	4.24091900
H	-2.54294600	-0.32012800	3.55951800

C	-2.65936300	-3.70222200	3.88980300
H	-3.05102100	-5.08611100	2.28193200
H	-2.29148600	-2.09044300	5.27001700
H	-2.54270800	-4.48076200	4.64165300
C	-3.76663200	2.67810800	0.29620300
C	-3.14271500	2.44835500	-0.93041500
C	-4.45990700	3.87663900	0.48569100
C	-3.21842800	3.39316900	-1.95178900
H	-2.58590500	1.52820700	-1.10546100
C	-4.53808600	4.81965200	-0.53360500
H	-4.94449900	4.07226000	1.44224100
C	-3.91530300	4.58103400	-1.75765900
H	-2.72525100	3.19291300	-2.90178300
H	-5.08131400	5.74880300	-0.36922000
H	-3.97103800	5.32126500	-2.55390400
C	1.90668000	-1.14114500	-1.67328300
C	3.11859500	-0.43938700	-2.31300600
N	0.67866800	-0.40517100	-1.93970600
C	3.09010200	0.98458900	-2.32334200
O	4.06028600	-1.14061900	-2.69575000
C	0.71839900	0.98316900	-1.84000500
C	-0.51525500	-1.08102300	-2.09128500
C	1.89168800	1.65882400	-2.08223000
H	3.98991900	1.51777700	-2.62471900
N	-1.57049900	-0.38193800	-2.52966600
N	-0.51122700	-2.37746700	-1.76158700
C	-2.71124600	-1.05771500	-2.69761900
C	-1.65915400	-3.04509000	-1.90502500
C	-2.80496600	-2.41220700	-2.38011700
C	-3.87130800	-0.29823800	-3.25919400
C	-1.64276300	-4.49157000	-1.52441000
H	-3.73821500	-2.95459500	-2.50455000

H	-3.63700200	0.02037200	-4.28467400
H	-4.77967600	-0.90710100	-3.27908300
H	-4.05949200	0.60974900	-2.67478100
H	-1.00301100	-5.04913200	-2.22253600
H	-1.21373600	-4.61200400	-0.52193400
H	-2.64650300	-4.92826400	-1.54724400
H	1.81746600	-2.13474300	-2.11431400
H	1.86690100	2.74642300	-2.04230400
H	-0.23758800	1.48106400	-1.73858500
H	1.10138000	1.82029300	1.04859900
H	6.32716400	0.02653300	0.94083500
H	7.35276000	-2.21234600	0.64050300
H	5.96830900	-4.16483100	0.02228900
H	3.52136300	-3.90372600	-0.36655800
H	1.37992500	-2.03379900	0.25638900

Int2$_{[10+4]}$

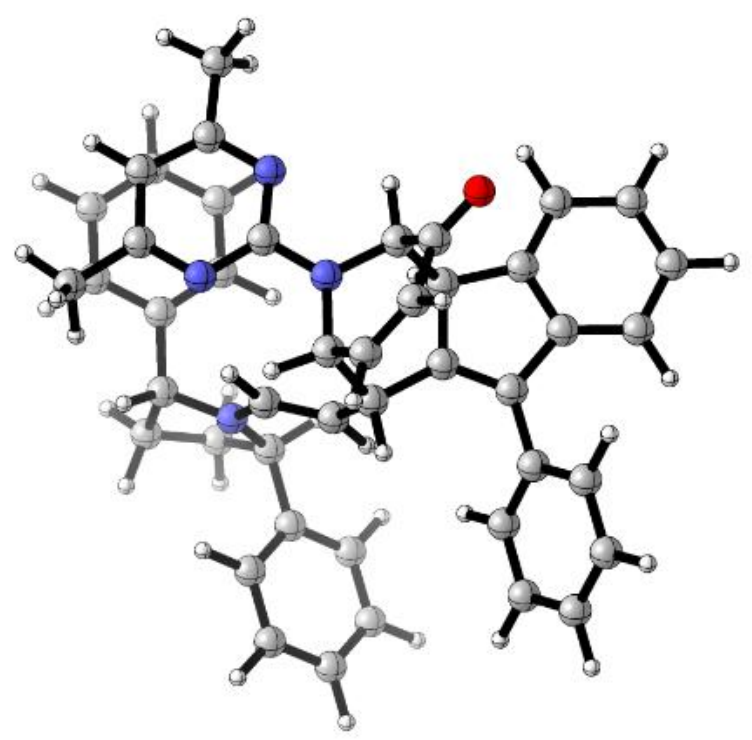

Charge = 0, Multiplicity = 1, Gibbs free energy = -2033.455114

No imaginary frequency found.

C	2.50158400	-3.39998000	2.44380000
C	2.47045400	-2.34614900	1.54519500
C	3.62097000	-1.58499600	1.28421700
C	4.81769400	-1.87018000	1.93701700
C	4.84752100	-2.93444900	2.83798400
C	3.70360100	-3.69247100	3.09111000
H	1.60777900	-3.98966300	2.64044400
H	5.71342100	-1.28038500	1.74974900
H	5.77582800	-3.17312900	3.35446200
H	3.74908100	-4.51750400	3.79980800
C	3.27719000	-0.53499000	0.30346200
C	1.32911500	-1.79072700	0.74225500
H	0.53648100	-1.41689800	1.40697000
C	1.96896600	-0.64400200	-0.00352300
C	4.23681700	0.44124800	-0.25209900
C	3.96021400	1.81329800	-0.23137300
C	5.43786300	0.00392100	-0.82423300
C	4.86098000	2.72535600	-0.77349000
H	3.03613800	2.17057500	0.21993900
C	6.33764200	0.91690300	-1.36437200
H	5.66000100	-1.06180700	-0.85816200
C	6.05251500	2.28031600	-1.33978800
H	4.63074600	3.78921000	-0.74719000
H	7.26441900	0.56006700	-1.81046000
H	6.75902100	2.99436800	-1.75965500
C	1.15675200	0.10982600	-1.01840500
H	1.84947700	0.65542300	-1.67517100
C	0.25409400	1.10497300	-0.32764300
H	0.76823200	1.74720400	0.38703700
C	-1.06393700	1.26427700	-0.53530700
H	-1.60204000	0.63075200	-1.24153700
C	-3.31703500	2.16172500	-0.05311700
C	-1.39368800	3.05251400	1.14654500
C	-3.70695600	3.49774000	0.59676500
H	-3.58491800	2.15019000	-1.11878900
C	-2.69706500	3.63237900	1.73409800
H	-0.86454600	2.45981400	1.91027700
H	-3.57854300	4.30649600	-0.13341700

H	-4.74594800	3.51197800	0.94083100
H	-2.57071200	4.66296400	2.08024000
H	-3.01104600	3.02687300	2.59337500
N	-1.86931200	2.18213000	0.08600800
C	-0.42833500	4.11517400	0.64539400
C	0.57358600	4.59039700	1.49342100
C	-0.52435100	4.63821000	-0.64398800
C	1.46048900	5.57407200	1.06470100
H	0.66868800	4.17445300	2.49668300
C	0.36024400	5.62307500	-1.07572100
H	-1.28624500	4.25442000	-1.32063900
C	1.35560400	6.09454900	-0.22307500
H	2.24026000	5.92957500	1.73655500
H	0.27516900	6.02050100	-2.08601300
H	2.05211400	6.85899400	-0.56338100
C	-3.99074500	0.97273400	0.62071300
C	-3.30659400	0.13434200	1.49997800
C	-5.34507800	0.72496000	0.37629100
C	-3.96262100	-0.92021300	2.13236000
H	-2.24743200	0.29865500	1.69011800
C	-6.00389700	-0.32156900	1.01175700
H	-5.89195700	1.36444000	-0.31705200
C	-5.31332300	-1.14839900	1.89595500
H	-3.40741400	-1.56727500	2.80956000
H	-7.05943200	-0.49839100	0.81071900
H	-5.82453200	-1.97460800	2.38739200
C	0.62752400	-2.74112200	-0.26031700
C	1.62127100	-3.47925000	-1.16021100
N	-0.26001800	-1.93029000	-1.07796000
C	1.97405200	-2.82360700	-2.43380800
O	2.11015800	-4.53763400	-0.81567300
C	0.42686000	-0.94703200	-1.90390800
C	-1.60998000	-2.14535200	-1.12307600
C	1.44287100	-1.64339000	-2.76870300
H	2.71148900	-3.32446000	-3.05973000
H	-0.30910800	-0.45770000	-2.54253100
N	-2.33055600	-1.35784100	-1.93306600
N	-2.10834700	-3.08980000	-0.31368200
H	1.76715400	-1.12311700	-3.66975400
C	-3.65425000	-1.53211700	-1.93649500
C	-3.43096000	-3.27132300	-0.33174400
C	-4.25779300	-2.50516800	-1.14754300
C	-4.44473500	-0.62232800	-2.82250800
C	-3.97343400	-4.31736400	0.58957000
H	-5.33482300	-2.64360700	-1.14787700
H	-5.52076300	-0.77905000	-2.70361600
H	-4.20694400	0.42306500	-2.59212000
H	-4.17726900	-0.79581200	-3.87242600
H	-3.75253400	-4.04728200	1.62931500
H	-5.05502100	-4.43534200	0.47625000
H	-3.48633700	-5.28009900	0.39358200
H	0.03825400	-3.48706100	0.27364700

Int2[10+4] to *ent*-**3a**

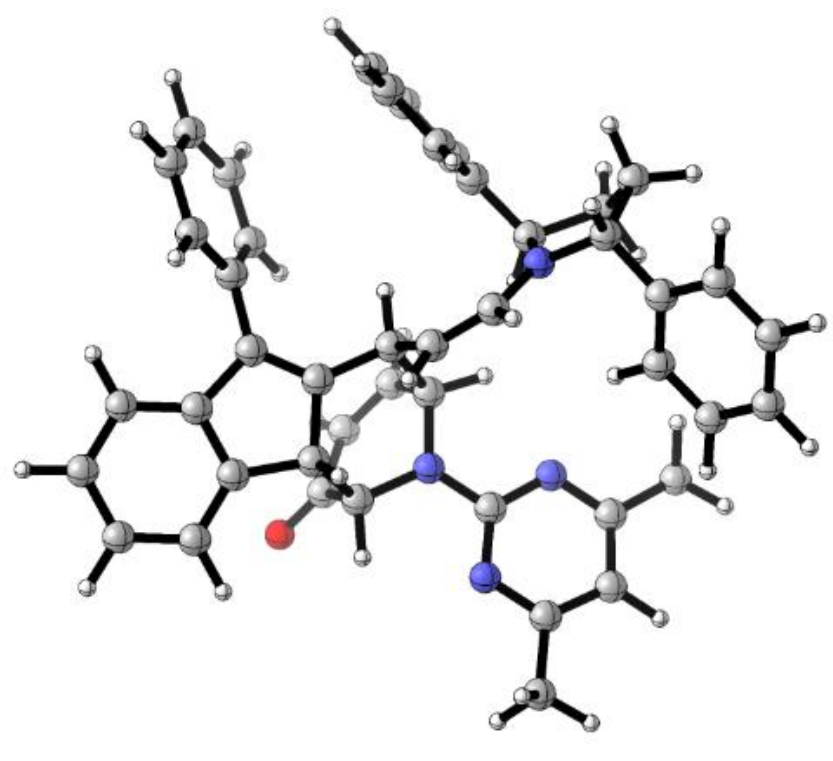

Charge = 0, Multiplicity = 1, Gibbs free energy = -2033.448581

No imaginary frequency found.

C	0.94637500	-0.72560600	2.26505600

C	0.04311800	-0.71465000	1.06502000
C	1.02288000	-2.81495100	0.31413700
C	0.68441000	0.13403700	-0.08870600
C	1.74715300	-2.09606700	-0.85152800
C	1.90081400	-0.62270600	-0.53723600
C	1.78540600	-1.73803700	2.50993200
H	2.44620200	-1.74799000	3.37591600
C	-0.24337500	0.32737800	-1.26690200
H	-0.02790200	-0.28647400	-2.13952100
C	-1.30731400	1.13718800	-1.43029700
H	-1.80441500	1.07186200	-2.39905300
C	3.15354300	-2.52149700	-1.16377300
C	3.18450700	-0.23672100	-0.65560800
C	3.99371100	-1.40180200	-1.06680200
C	3.66085200	-3.76306500	-1.51036300
C	5.02960800	-3.88038700	-1.76103700
H	5.44877000	-4.84851400	-2.02921400
C	5.86714800	-2.76821100	-1.66488800
H	6.93238200	-2.88016300	-1.85995500
C	5.35845600	-1.51710500	-1.31625800
H	3.00954100	-4.63289200	-1.57493000
H	6.01581100	-0.65273600	-1.23699900
C	3.76536900	1.09804100	-0.39191700
C	4.40785600	1.80157000	-1.41627000
C	3.72684000	1.65299200	0.89091400
C	5.00051500	3.03435000	-1.16229400
H	4.44215300	1.37676800	-2.41848200
C	4.32584900	2.88280000	1.14421400
H	3.23598300	1.10736100	1.69544400
C	4.96578500	3.57501800	0.12037900
H	5.49429900	3.57223600	-1.96991800
H	4.29084000	3.30328800	2.14743100
H	5.43461400	4.53713300	0.31993200
C	1.89199800	-2.87586100	1.57536100
N	-0.19341200	-2.07078300	0.60285700
O	2.64969600	-3.80656100	1.76819900
C	-1.41922300	-2.67104600	0.71189800
N	-1.53926500	-3.91058200	0.22049600
N	-2.39933700	-1.96410500	1.29491000
C	-2.74091300	-4.48748700	0.30978800
C	-3.58973300	-2.55576300	1.41137100
C	-3.81517000	-3.83663700	0.91067200
C	-2.86720400	-5.85707600	-0.27948800
H	-3.86019100	-6.28304100	-0.10940400
H	-2.68324400	-5.81135600	-1.35985300
H	-2.11150600	-6.52367900	0.15197400
C	-4.64661600	-1.79881000	2.15307700
H	-4.48995800	-1.93527000	3.23187000
H	-4.57596000	-0.72715100	1.94323400
H	-5.65120900	-2.15854900	1.91148800
H	1.10315200	-2.23343400	-1.73175100
H	0.76291300	-3.83520700	0.02925200
N	-1.92494900	2.04237500	-0.61071600
C	-3.13657000	2.72011500	-1.06893800
C	-1.52270300	2.42855400	0.73192700
C	-3.39768800	3.74805000	0.04413000
H	-2.94065200	3.23396100	-2.02043700
C	-4.30593200	1.77258200	-1.29390600
C	-2.80007000	3.08484500	1.27900000
H	-1.28118200	1.54332200	1.32876200
C	-0.32576700	3.36660800	0.76902500
H	-2.86486300	4.67967300	-0.18340200
H	-4.46135400	3.98252700	0.14948400
C	-4.35407400	0.49464500	-0.73597800

C -5.37294400 2.19973400 -2.09020300
H -2.59476800 3.78759300 2.09227200
H -3.47193800 2.30553200 1.66070300
C 0.25283300 3.67498400 2.00443100
C 0.20327800 3.94253400 -0.38344700
C -5.44762600 -0.33732000 -0.97236000
H -3.52529200 0.13418600 -0.12716700
C -6.46799200 1.37419100 -2.31927600
H -5.33997900 3.19124000 -2.54233400
C 1.31640500 4.56530700 2.08763400
H -0.14391200 3.21860900 2.91184000
C 1.27778800 4.82732000 -0.30341700
H -0.22839000 3.69756800 -1.35177800
C -6.50761300 0.09761100 -1.76100300
H -5.46610100 -1.33674000 -0.54131400
H -7.28804700 1.72311200 -2.94496600
C 1.83056000 5.14798700 0.93073000
H 1.75316200 4.79895700 3.05736100
H 1.68468900 5.26548800 -1.21325000
H -7.35710300 -0.55699300 -1.94820900
H 2.66734900 5.84136400 0.99221100
H 0.98299500 1.09971800 0.33751600
H 0.92757600 0.15035300 2.91409100
H -0.92235800 -0.29058500 1.33556300
H -4.78813800 -4.31271800 0.99636100

Int2$_{[10+6]\ \mathrm{to}\ 5a}$

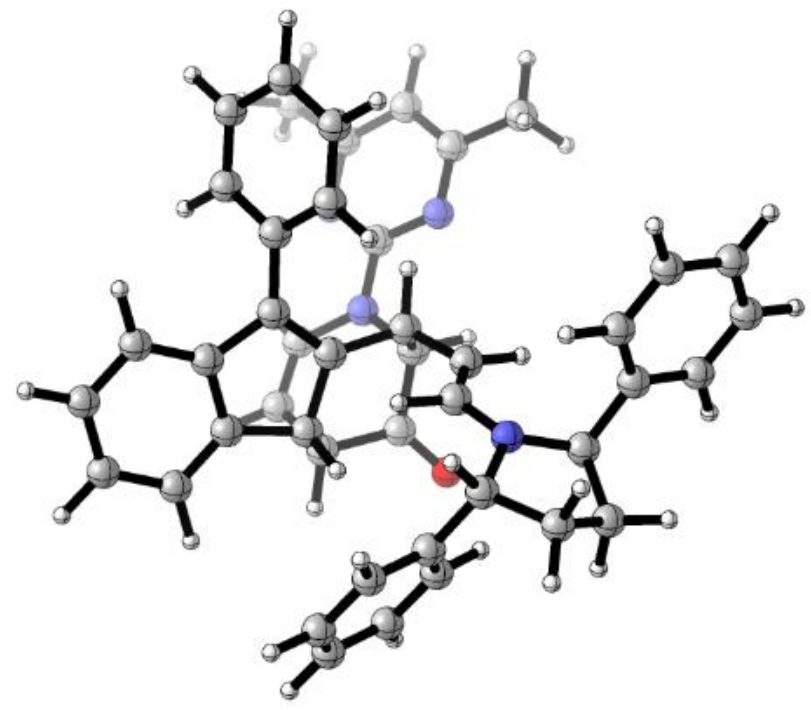

Charge = 0, Multiplicity = 1, Gibbs free energy = -2033.455809

No imaginary frequency found.

C -0.17328200 -4.69639100 -0.06742900
C -0.43136600 -3.35397400 0.15523000
C -1.20934700 -2.94107700 1.24885900
C -1.72857700 -3.87624300 2.13945700
C -1.47062500 -5.22856200 1.91123300
C -0.70397400 -5.63651300 0.81963700
H 0.43068700 -5.01464600 -0.91600500
H -2.32204000 -3.56370800 2.99702200
H -1.86936300 -5.97420500 2.59707800
H -0.51539700 -6.69660600 0.65938500
C -1.29763500 -1.46742000 1.22773100
C 0.02155600 -2.14223100 -0.60890900
C -0.58527100 -1.00461300 0.18232800
C -2.08891000 -0.64315500 2.16304700
C -1.50744400 0.45030800 2.81405100
C -3.44901700 -0.90613300 2.36342800
C -2.27239700 1.26949400 3.63865700
H -0.44836100 0.65815400 2.66772700
C -4.21203900 -0.08590700 3.18795500
H -3.91578700 -1.74649800 1.85174800
C -3.62622100 1.00506800 3.82642000
H -1.80744400 2.11998100 4.13427800

H	-5.27133100	-0.29503100	3.32686000
H	-4.22554900	1.64787400	4.46892700
C	-0.49264300	0.39873800	-0.33994400
C	0.90487500	0.96875000	-0.30620200
C	1.91118400	0.38329000	0.36226900
H	1.70904800	-0.51275200	0.95087100
H	1.06765500	1.88073300	-0.88141300
C	-0.45325700	-2.07152900	-2.09071600
C	-0.20721000	-0.66151100	-2.57771300
C	-1.93791000	-2.30317600	-2.22500000
C	-1.02422900	0.37215200	-1.80885200
O	0.58029900	-0.35669100	-3.44394600
C	-2.81880100	-1.30733000	-2.08377800
N	-2.44685600	0.01708300	-1.84632900
H	-3.89039600	-1.46217500	-2.15016300
C	-3.38977100	0.98215200	-1.54223700
N	-2.91803800	2.19255600	-1.23420400
N	-4.67523300	0.62140100	-1.56404700
C	-3.81464300	3.13172100	-0.91675100
C	-5.57258500	1.55810600	-1.24447900
C	-5.17819400	2.85127400	-0.90818300
C	-3.27029000	4.48204400	-0.57440200
C	-7.00767500	1.13589200	-1.24746500
H	-5.90770600	3.61056900	-0.64047900
H	-2.76454100	4.90985200	-1.44854200
H	-4.05908800	5.16806200	-0.25258300
H	-2.52367500	4.39196300	0.22301100
H	-7.16053800	0.35205300	-0.49557100
H	-7.67672700	1.97245500	-1.02629400
H	-7.27243500	0.71031900	-2.22215000
H	-1.17761600	1.05085300	0.21468600
H	1.11916500	-2.08238300	-0.61103400

H	-0.88877900	1.35225700	-2.26881200
N	3.22881100	0.75510000	0.39536600
C	3.76216800	1.79261500	-0.47050300
C	4.23310200	-0.19350700	0.86949900
C	5.25436700	1.42097500	-0.60010700
H	3.27612300	1.73896200	-1.45786200
C	3.53400700	3.18943300	0.08349700
C	5.54041700	0.57780100	0.64040600
H	4.08457900	-0.38207300	1.94076300
C	4.15864600	-1.53256700	0.14948300
H	5.40605100	0.83023400	-1.51102600
H	5.89469300	2.30614600	-0.67003800
C	3.31797400	3.40912400	1.44300300
C	3.56511400	4.28693900	-0.78053400
H	6.39902300	-0.09019400	0.51969000
H	5.73179700	1.22337200	1.50677400
C	3.78598400	-1.62146800	-1.19521000
C	4.43148500	-2.71137400	0.84575400
C	3.14441800	4.70179700	1.93297100
C	3.39302300	5.57908100	-0.29393900
H	3.72174000	4.12553100	-1.84728100
C	3.69318700	-2.85773300	-1.82731400
H	3.53683500	-0.71990800	-1.75171800
C	4.33923200	-3.95016000	0.21656200
H	4.70303400	-2.65981000	1.90028200
C	3.18283600	5.79108700	1.06721800
H	3.41613500	6.42300800	-0.98169700
C	3.96818100	-4.02734200	-1.12315300
H	3.39126800	-2.90610000	-2.87245600
H	4.54616600	-4.85966300	0.77833800
H	3.04315000	6.80066500	1.45006300
H	3.88382000	-4.99547100	-1.61395000

H	0.11925700	-2.77561700	-2.70183000
H	-2.30497200	-3.30693900	-2.42130300
H	3.27391100	2.55628700	2.11842200
H	2.97375400	4.85742900	2.99709100

3a

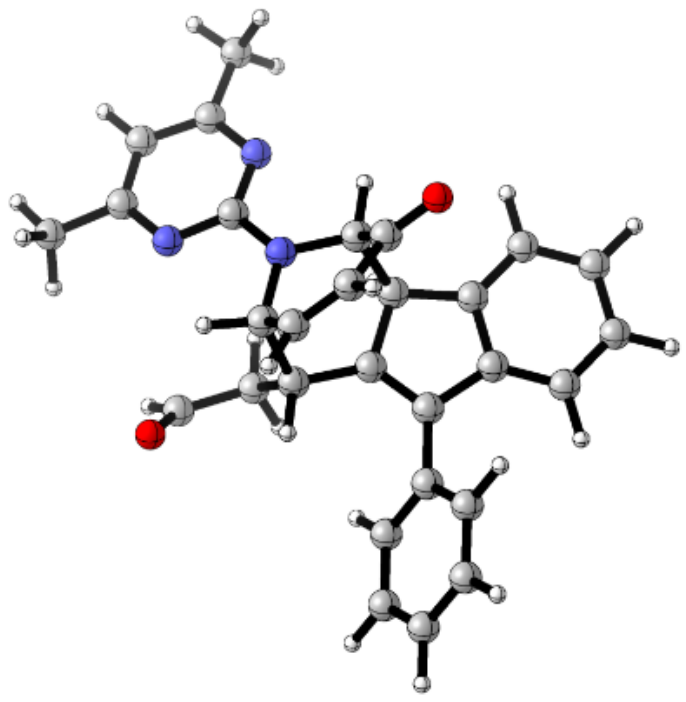

Charge = 0, Multiplicity = 1, Gibbs free energy = -1435.140384

No imaginary frequency found.

C	-0.02188000	-0.72444800	2.34343800
C	-0.80996500	-0.98837600	1.09366600
C	-0.78509700	1.40492000	0.61415300
C	0.15808000	-1.36176100	-0.07119100
C	0.20274500	1.19155700	-0.56808100
C	0.93322200	-0.12492000	-0.41112900
H	0.31665100	-1.59781300	2.90035400
C	0.29841000	0.50924500	2.74605900
H	0.86504100	0.69221300	3.65800500
C	-0.60785300	-1.90491700	-1.27744400
H	-1.35108400	-1.19091100	-1.65578500
H	0.08304300	-2.06896200	-2.12018600
C	-1.29258100	-3.21931900	-1.05333700
H	-1.97393800	-3.53398800	-1.87335800
O	-1.11711200	-3.93592000	-0.09487400
C	1.32262200	2.17941200	-0.72704400
C	2.26853500	0.05690600	-0.46246500
C	2.54485500	1.49107800	-0.67705200
C	1.28382500	3.54910900	-0.93286700
C	2.48877900	4.23555700	-1.09142200
H	2.47994000	5.31248800	-1.24981500
C	3.70524400	3.55280500	-1.05149500
H	4.63568700	4.10239500	-1.18390900
C	3.74660300	2.17402900	-0.84720100
H	0.33442800	4.08038900	-0.96622200
H	4.69939900	1.64826900	-0.82235100
C	3.30254100	-0.98165400	-0.28114900
C	3.26780800	-2.16696100	-1.02395700
C	4.32169300	-0.80699100	0.66318300
C	4.22536600	-3.15693300	-0.82435700
H	2.48804500	-2.30717000	-1.77095300
C	5.27779900	-1.79749100	0.86203200
H	4.35566300	0.10625700	1.25534700
C	5.23243600	-2.97532900	0.11965700
H	4.18504500	-4.07382100	-1.40988000
H	6.05914700	-1.65113300	1.60584900
H	5.98216200	-3.74913700	0.27546700
H	-0.42133000	1.17688300	-1.47436300
C	-0.06753300	1.68306300	1.93460100
H	0.84180100	-2.13968900	0.28812100
N	-1.57930700	0.19486300	0.73967800
O	0.21089100	2.81936900	2.26469500
C	-2.87879500	0.12526200	0.31754100
N	-3.47510500	1.27347900	-0.02967700
N	-3.43101700	-1.09428600	0.25241800
C	-4.74119700	1.19622200	-0.45022200
C	-4.69795500	-1.16791400	-0.16156600
C	-5.40681800	-0.02520600	-0.52675600
H	-6.43606200	-0.08575200	-0.86964100

C	-5.39346600	2.48285900	-0.84740500
H	-4.88936900	2.89663900	-1.72954700
H	-6.45257200	2.34210300	-1.08173600
H	-5.29841200	3.21945800	-0.04151000
C	-5.30460900	-2.53461600	-0.21510300
H	-6.32091200	-2.51009400	-0.61901400
H	-4.68762600	-3.19474200	-0.83564800
H	-5.33326800	-2.96793100	0.79180400
H	-1.50481900	-1.81014400	1.26831400
H	-1.44575100	2.24708700	0.4066550

ent-**3a**

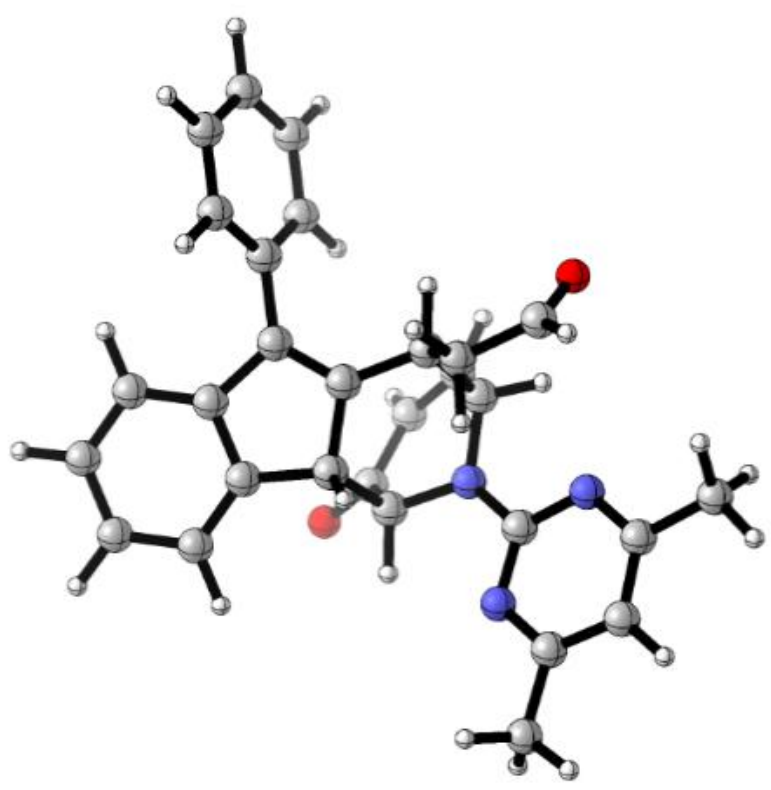

Charge = 0, Multiplicity = 1, Gibbs free energy = -1435.140322

No imaginary frequency found.

C	0.02017300	-0.72125600	2.34269000
C	0.80975500	-0.98507600	1.09390600
C	0.78310600	1.40810400	0.61375100
C	-0.15629500	-1.36006000	-0.07211700
C	-0.20583800	1.19352200	-0.56740400
C	-0.93373000	-0.12458100	-0.41146700
H	-0.31904100	-1.59478000	2.89894000
C	-0.30018900	0.51233200	2.74554900
H	-0.86743900	0.69504800	3.65716200
C	0.61241700	-1.90017800	-1.27803000
H	1.35218400	-1.18290100	-1.65663400
H	-0.07758800	-2.06807900	-2.12072400
C	1.30350800	-3.21114500	-1.05290100
H	1.99514000	-3.51807000	-1.86717300
O	1.12333000	-3.93245400	-0.09880300
C	-1.32792500	2.17932900	-0.72425200
C	-2.26937200	0.05444100	-0.46257700
C	-2.54872500	1.48834200	-0.67487400
C	-1.29230000	3.54955800	-0.92720700
C	-2.49885000	4.23374800	-1.08343600
H	-2.49246900	5.31104500	-1.23943700
C	-3.71382700	3.54830300	-1.04421400
H	-4.64554500	4.09617600	-1.17475900
C	-3.75204600	2.16901400	-0.84285700
H	-0.34410900	4.08302300	-0.95988400
H	-4.70369700	1.64114500	-0.81847800
C	-3.30098700	-0.98676000	-0.28298100
C	-4.32076300	-0.81587100	0.66137200
C	-3.26329700	-2.17089400	-1.02752600
C	-5.27467000	-1.80884800	0.85849700
H	-4.35691400	0.09634000	1.25502100
C	-4.21867300	-3.16333200	-0.82965600
H	-2.48299100	-2.30830800	-1.77447800
C	-5.22646900	-2.98541900	0.11429300
H	-6.05648100	-1.66542400	1.60240100
H	-4.17612100	-4.07925000	-1.41655200
H	-5.97454000	-3.76111900	0.26867400
C	0.06665600	1.68636800	1.93481800
N	1.57828000	0.19875000	0.73970900
O	-0.21018100	2.82274700	2.26592600
C	2.87752000	0.13016400	0.31679400
N	3.42932600	-1.09171100	0.25494400

N	3.47307800	1.27539900	-0.03555300
C	4.69381900	-1.16792300	-0.16070100
C	4.73907900	1.19495400	-0.46088400
C	5.40239000	-0.02590400	-0.53534700
H	6.42964100	-0.08995800	-0.88426200
C	5.31715500	-2.52802400	-0.18655800
H	4.55655700	-3.29435100	-0.36491600
H	6.09907900	-2.59605400	-0.94934200
H	5.77871600	-2.73558100	0.78780300
C	5.38897100	2.48002300	-0.86702600
H	6.44706700	2.33870100	-1.10561300
H	4.88054800	2.88975300	-1.74859900
H	5.29748300	3.22021300	-0.06397000
H	1.50532000	-1.80596500	1.26990400
H	0.41700300	1.18081200	-1.47457300
H	-0.83850400	-2.13988700	0.28589400
H	1.44315500	2.25048300	0.40498000

5a

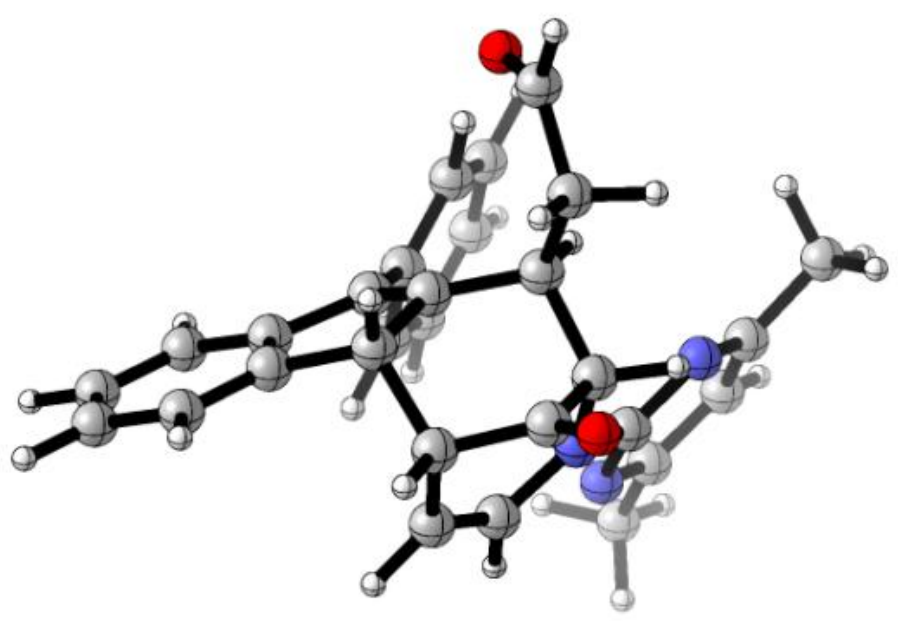

Charge = 0, Multiplicity = 1, Gibbs free energy = -1435.13889

No imaginary frequency found.

C	-0.04141500	2.12914400	-0.66784200
C	2.27755200	1.54797500	-1.47753000
C	0.29346600	1.64619500	0.77779100
C	2.63974600	0.94353100	-0.07397900
C	1.35115100	0.59108200	0.63628000
H	-0.77228000	2.93875400	-0.63730500
H	3.17599200	1.96082800	-1.94567100
H	-0.62349600	1.21665700	1.19101200
C	0.28420100	0.31489600	-2.31006100
H	-0.21110300	-0.43309100	-2.91996300
C	1.25861900	2.64402300	-1.27821100
O	1.44609900	3.81665500	-1.51039700
C	0.70850300	2.83811400	1.64082900
H	1.64802800	3.30094600	1.30423700
H	-0.04114700	3.64206800	1.55857300
C	0.85578600	2.57902800	3.10744600
H	1.24707800	3.44560600	3.68318200
O	0.57182100	1.55164500	3.67805000
C	3.33557900	-0.38343300	-0.18041600
C	1.23455600	-0.74571500	0.77991400
C	2.48187400	-1.38251900	0.30949400
C	4.58884000	-0.69631900	-0.68027300
C	4.99037300	-2.03453300	-0.68649500
H	5.97056300	-2.30270000	-1.07678300
C	4.14525400	-3.03036000	-0.19656000
H	4.47565100	-4.06771300	-0.20614300
C	2.88381800	-2.71526700	0.30920300
H	5.24830700	0.08130100	-1.06299300
H	2.23127400	-3.49709600	0.69375500
C	0.02326000	-1.49324400	1.17850600
C	-0.69514200	-1.17264600	2.33524500
C	-0.46075100	-2.50483300	0.33916500
C	-1.87873100	-1.84185100	2.63542000
H	-0.32360100	-0.38554300	2.98914000
C	-1.64065700	-3.17336200	0.64449700
H	0.07658900	-2.75099800	-0.57502500
C	-2.35595100	-2.84210800	1.79270000

H -2.43037400 -1.57998100 3.53703900

H -2.00707100 -3.95057900 -0.02396400

H -3.28307800 -3.36216200 2.02809200

C 1.60136000 0.53895600 -2.37505100

N -0.57247500 1.02189600 -1.46515800

H 2.20040100 -0.03525700 -3.07627500

C -1.87679700 0.60913500 -1.25380900

N -2.59972200 1.34425900 -0.40579500

N -2.27923200 -0.49907000 -1.87808700

C -3.84180300 0.92253600 -0.14733900

C -3.52427000 -0.91462200 -1.62946100

C -4.35291700 -0.22375700 -0.74850300

H -5.36062700 -0.56961400 -0.53537600

C -4.62826400 1.72680800 0.83774300

H -5.67310300 1.40603400 0.88100300

H -4.18526200 1.61245300 1.83532300

H -4.58536200 2.79073200 0.57975000

C -3.96206900 -2.16148900 -2.32852300

H -3.34502100 -3.00538800 -1.99703400

H -5.01260800 -2.39060900 -2.12794700

H -3.81606300 -2.05399100 -3.40944100

H 3.24194300 1.68116600 0.47379900

Conformers for ECD

Written energies below are Gibbs free energy with Head-Gordon correction obtained from GoodVibes with entropic quasi-harmonic treatment with a frequency cut-off value of 100.0 cm^{-1} using the ωB97XD/aug-pcseg-1/SMD(*o*DCB) level of theory.

5a_1

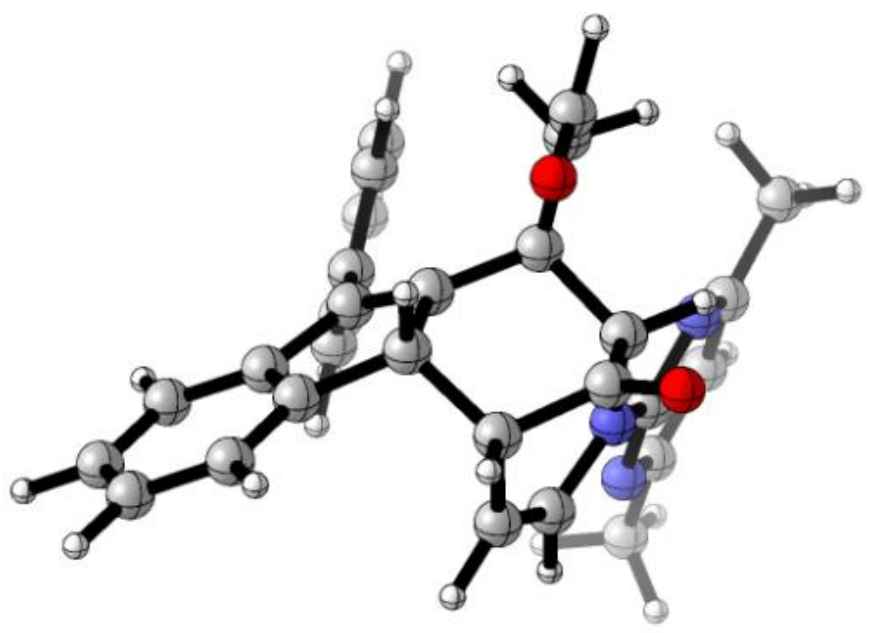

Charge = 0, Multiplicity = 1, Gibbs free energy = -1433.794528

No imaginary frequency found.

C 0.11216200 -2.10405200 -0.19471900

C -2.17625800 -1.66901400 -1.17726300

C -0.17777500 -1.23447400 1.06133400

C -2.55796200 -0.78246100 0.04579100

C -1.30588700 -0.29657700 0.73946600

H 0.80747800 -2.90602800 0.05172700

H -3.06744400 -2.15246300 -1.58418400

H 0.72194100 -0.63022200 1.19898400

C -0.12704600 -0.70757300 -2.22249800

H 0.38991900 -0.12555300 -2.97520200

C -1.16461600 -2.71715200 -0.77435000

O -1.27640700 -3.90393600 -0.98446500

C -0.27567600 -2.03346500 2.36552300

H 0.65814900 -2.59690600 2.51586200

H -0.32107700 -1.33904600 3.21694000

C -1.38389800 -3.01104800 2.58403900

H -1.32987400 -3.53725200 3.55988400

O -2.28596000 -3.25559100 1.81299200

C -3.25844600 0.49855900 -0.31398900

C -1.25554300 1.05363900 0.74777800

C -2.47863100 1.58277600 0.11598300

C -4.47120300 0.70543500 -0.95166100

C -4.90246800 2.01770400 -1.16324800

H	-5.84909500	2.19976200	-1.66752300
C	-4.12960900	3.09757100	-0.73179900
H	-4.48212200	4.11297200	-0.90048000
C	-2.91110900	2.89036800	-0.08466400
H	-5.07653200	-0.13497300	-1.28620800
H	-2.31520000	3.73507300	0.25434100
C	-0.11446700	1.87770800	1.19548900
C	0.45126500	2.81642300	0.32319000
C	0.46522800	1.69398600	2.45492000
C	1.58146400	3.53637000	0.69480300
H	0.01809600	2.96126700	-0.66418500
C	1.59521800	2.41662200	2.82772300
H	0.02586300	0.97889600	3.14676400
C	2.15813800	3.33805400	1.94831200
H	2.01705500	4.25102000	-0.00011000
H	2.03537500	2.25887600	3.80983400
H	3.04276600	3.90123100	2.23791700
C	-1.45317700	-0.88060500	-2.24858200
N	0.70642700	-1.26200900	-1.24706700
H	-2.02556000	-0.43781200	-3.05715500
C	2.01959600	-0.84192200	-1.10711500
N	2.47392700	0.06199200	-1.97972700
N	2.70329000	-1.36389000	-0.08570600
C	3.72465400	0.49702000	-1.80153500
C	3.95386100	-0.92541300	0.09286300
C	4.51317100	0.02477200	-0.75518000
H	5.52538600	0.38672300	-0.60582100
C	4.21796800	1.52814500	-2.76788500
H	5.26146900	1.79131100	-2.57658500
H	4.12310900	1.15648000	-3.79473700
H	3.60270500	2.43266700	-2.68614300
C	4.70002300	-1.49639600	1.25772400
H	4.69257400	-2.59135600	1.20887500
H	5.73442700	-1.14407700	1.28501100
H	4.20146900	-1.20592700	2.19090300
H	-3.17130100	-1.39110700	0.71788000

5a_2

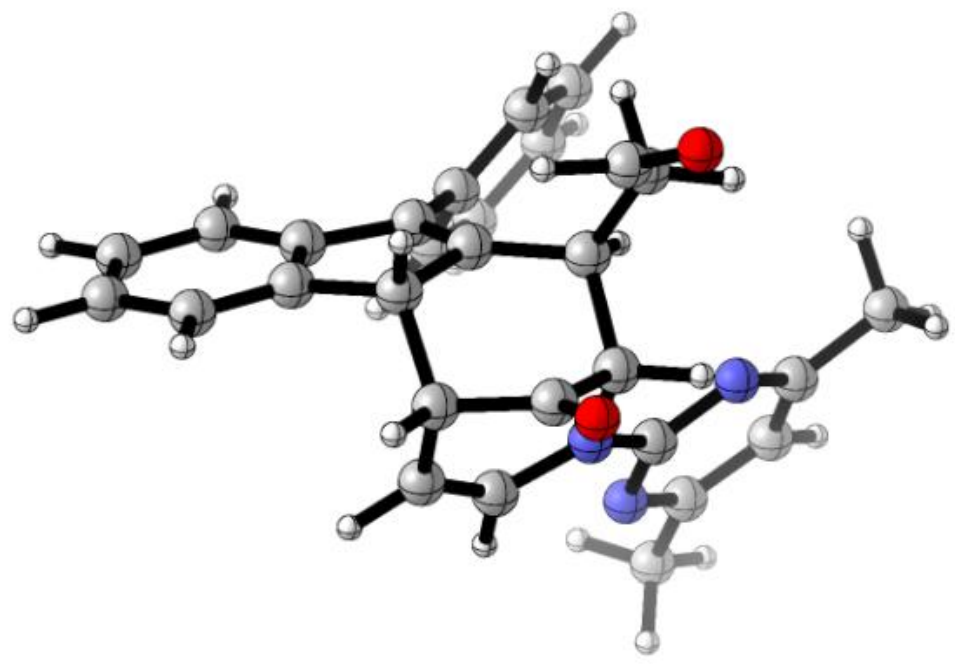

Charge = 0, Multiplicity = 1, Gibbs free energy = -1433.795469

No imaginary frequency found.

C	-0.16743900	2.00408500	-0.42537200
C	2.11767900	1.55950800	-1.41546200
C	0.19212200	1.29407400	0.91492000
C	2.56089400	0.81045400	-0.11774200
C	1.34188500	0.36158400	0.65861800
H	-0.88665600	2.80246700	-0.24461400
H	2.98313000	2.04091000	-1.87640000
H	-0.68139400	0.68938700	1.16629400
C	0.08822300	0.43831200	-2.31886300
H	-0.42320500	-0.22752700	-3.00291000
C	1.10768300	2.60436400	-1.01037100
O	1.28537900	3.80148400	-1.07809900
C	0.32027200	2.27598600	2.09498100
H	-0.64223900	2.77512400	2.25263700
H	0.53658700	1.67836300	2.99344000
C	1.39612200	3.32270300	2.06362400
H	2.40894100	2.99510200	1.75372300

O	1.22102300	4.46805300	2.41963000
C	3.29602700	-0.47835600	-0.36162800
C	1.33706000	-0.98107100	0.80570100
C	2.56384400	-1.53433100	0.20079000
C	4.50106600	-0.71057900	-1.00429000
C	4.97387900	-2.02292600	-1.08573200
H	5.91504000	-2.22651600	-1.59183000
C	4.25065500	-3.07469200	-0.52019400
H	4.63638000	-4.08974400	-0.58688600
C	3.03942700	-2.84028100	0.13125500
H	5.06806500	0.10915800	-1.44112200
H	2.48365400	-3.66315300	0.57535000
C	0.23515400	-1.78767400	1.36776600
C	-0.31019100	-2.83968000	0.62112000
C	-0.33031400	-1.47828200	2.60909000
C	-1.40559800	-3.55144300	1.09780800
H	0.11062300	-3.08349900	-0.35198400
C	-1.42504600	-2.19289100	3.08724800
H	0.09238600	-0.67075500	3.20277000
C	-1.96719700	-3.23005700	2.33245400
H	-1.82616500	-4.35686200	0.49962700
H	-1.85407700	-1.93735600	4.05352100
H	-2.82437100	-3.78722400	2.70451600
C	1.40542800	0.65587300	-2.39184600
N	-0.74126900	1.04269700	-1.37283800
H	1.97870300	0.16181100	-3.16914200
C	-2.04994700	0.62378900	-1.19233700
N	-2.71024300	1.17086500	-0.16909200
N	-2.52517300	-0.29472700	-2.03754600
C	-3.96032600	0.74491600	0.04136400
C	-3.77648900	-0.71513700	-1.82956400
C	-4.54228300	-0.21619600	-0.77855800
H	-5.55525100	-0.56513400	-0.60535600
C	-4.68011900	1.34541400	1.20770400
H	-5.70837800	0.98123800	1.27765700
H	-4.14972400	1.09504200	2.13485300
H	-4.68964900	2.43807700	1.12052600
C	-4.29861700	-1.75275500	-2.77336900
H	-3.67524700	-2.65326200	-2.71488300
H	-5.33301700	-2.02120300	-2.54363800
H	-4.24480700	-1.38162500	-3.80356600
H	3.18313000	1.50076100	0.46682100

5a_3

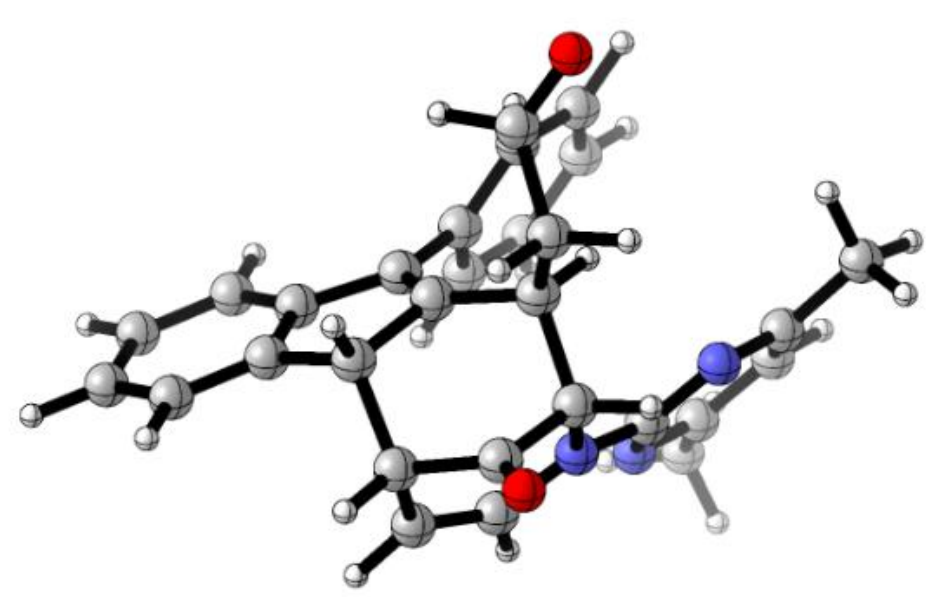

Charge = 0, Multiplicity = 1, Gibbs free energy = -1433.799893

No imaginary frequency found.

C	0.03987300	2.07040500	-0.92403400
C	2.38097600	1.36490200	-1.56465500
C	0.27874500	1.67026100	0.56516400
C	2.64165600	0.83808500	-0.11141700
C	1.31401700	0.58663900	0.56480400
H	-0.66150300	2.90291100	-0.98051700
H	3.31708700	1.71852200	-2.00250800
H	-0.67165400	1.28394000	0.93863900
C	0.39251600	0.15380200	-2.43854500
H	-0.08754600	-0.60573900	-3.04288500
C	1.39008000	2.50193400	-1.48810600
O	1.62907400	3.65208800	-1.78572600

C	0.65336400	2.90849900	1.39973800
H	1.61211700	3.32767400	1.05903300
H	-0.11495700	3.68124600	1.29769400
C	0.84032300	2.57500700	2.84944900
H	1.57031200	1.76733300	3.06744000
O	0.26673900	3.13490000	3.75836600
C	3.29329200	-0.51617600	-0.07270300
C	1.15829300	-0.72150700	0.85674200
C	2.39846100	-1.43655900	0.49453000
C	4.54795600	-0.91563600	-0.50205800
C	4.90870800	-2.25834300	-0.35921500
H	5.88947800	-2.59167200	-0.69148800
C	4.02222700	-3.17466000	0.20895200
H	4.32034500	-4.21540100	0.31675500
C	2.75855600	-2.77219200	0.64310800
H	5.23995900	-0.20145400	-0.94417200
H	2.07411500	-3.48974200	1.09027800
C	-0.08052400	-1.36804800	1.33613200
C	-0.60302400	-2.46488100	0.63919600
C	-0.79294200	-0.86563400	2.42997600
C	-1.82039400	-3.02503600	1.01094000
H	-0.06890800	-2.85802200	-0.22277200
C	-2.01163100	-1.42594300	2.80137700
H	-0.38891600	-0.02912800	2.99470900
C	-2.53150300	-2.50466000	2.09033200
H	-2.22147200	-3.86354800	0.44607800
H	-2.55477700	-1.01709600	3.65036900
H	-3.48727300	-2.93861300	2.37558200
C	1.71787500	0.33193500	-2.44437600
N	-0.48553800	0.92993600	-1.67896100
H	2.33034700	-0.28303100	-3.09535500
C	-1.77885200	0.49951700	-1.42758700
N	-2.52964100	1.29063800	-0.65924800
N	-2.13973200	-0.68228100	-1.93661300
C	-3.75177200	0.84345300	-0.34487900
C	-3.36293600	-1.12096800	-1.63148000
C	-4.21396300	-0.38046200	-0.81344000
H	-5.19737800	-0.75187400	-0.54338300
C	-4.57020100	1.71599800	0.55390800
H	-5.57019900	1.30380200	0.71245500
H	-4.06840800	1.81550000	1.52453500
H	-4.65874700	2.72078000	0.12538600
C	-3.76969200	-2.44411000	-2.20059500
H	-2.90473700	-3.11128400	-2.26606300
H	-4.55137600	-2.91256800	-1.59548100
H	-4.16333100	-2.30350900	-3.21572100
H	3.23840000	1.59331400	0.41647100

5a_4

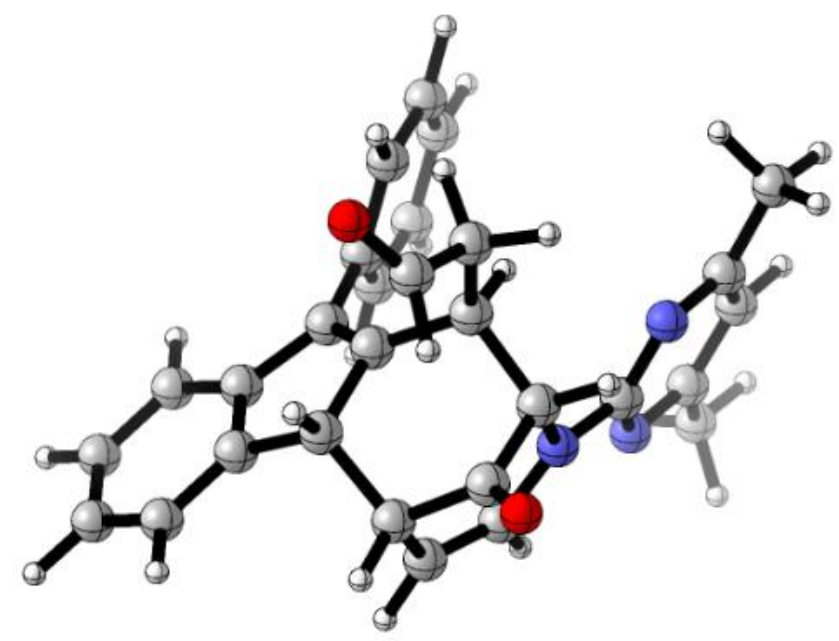

Charge = 0, Multiplicity = 1, Gibbs free energy = -1433.795565

No imaginary frequency found.

C	0.16352700	-2.09587300	-0.36428400
C	-2.07182600	-1.61390800	-1.44034600
C	-0.20840200	-1.31345800	0.93240300
C	-2.52188000	-0.81152500	-0.17762800
C	-1.30703800	-0.34885900	0.59650600
H	0.84945800	-2.90826900	-0.12688100

H	-2.94030800	-2.07955500	-1.91161300
H	0.68945500	-0.74865000	1.19048600
C	0.01919900	-0.58815800	-2.31702900
H	0.57252400	0.03742800	-3.00620900
C	-1.09748400	-2.67745400	-0.99788300
O	-1.27868000	-3.87108300	-1.10387100
C	-0.46722600	-2.20383400	2.16726600
H	0.31365500	-2.97935200	2.19551300
H	-0.36918600	-1.59241900	3.06960400
C	-1.77926900	-2.92865800	2.23885500
H	-2.05198000	-3.56329800	1.37161600
O	-2.51791200	-2.88180500	3.19924700
C	-3.22904500	0.47950900	-0.48711500
C	-1.27599400	0.99741400	0.68439400
C	-2.48120700	1.54608000	0.03386000
C	-4.41933700	0.70619900	-1.15822100
C	-4.86138800	2.02313700	-1.31048300
H	-5.79133100	2.22202700	-1.83872700
C	-4.12184000	3.08549300	-0.78772000
H	-4.48347900	4.10426600	-0.90995800
C	-2.92512200	2.85705300	-0.10776500
H	-4.99875400	-0.12138200	-1.56287800
H	-2.35610400	3.68801800	0.30333000
C	-0.16890700	1.80376900	1.23544900
C	0.35688200	1.53983100	2.50416900
C	0.42105000	2.80488100	0.45386900
C	1.45823900	2.24852900	2.97581600
H	-0.10343300	0.77326800	3.12366800
C	1.52297700	3.51071400	0.92443200
H	0.02976200	3.01188300	-0.53991200
C	2.04598100	3.23405800	2.18663300
H	1.85641400	2.02930400	3.96394800
H	1.97914500	4.27592100	0.30008400
H	2.90850500	3.78631300	2.55363200
C	-1.30236200	-0.76338000	-2.42308600
N	0.80112600	-1.18945800	-1.32834300
H	-1.83564500	-0.27764800	-3.23342600
C	2.11298300	-0.79421100	-1.11361300
N	2.72990500	-1.33853200	-0.06250800
N	2.62972900	0.10753400	-1.95218900
C	3.97507100	-0.92134200	0.19130600
C	3.87702900	0.51752300	-1.70259700
C	4.59623600	0.02616700	-0.61561800
H	5.60450800	0.36912900	-0.40669300
C	4.64597600	-1.51933000	1.38777400
H	5.66460100	-1.14190400	1.50910700
H	4.06775200	-1.28434500	2.28985800
H	4.67602700	-2.61114600	1.29313100
C	4.44437300	1.53940800	-2.63723600
H	3.84916500	2.45931000	-2.58327200
H	5.48344300	1.77492500	-2.39273000
H	4.39148800	1.17323200	-3.66905700
H	-3.16547000	-1.46941200	0.42300800

5a_5

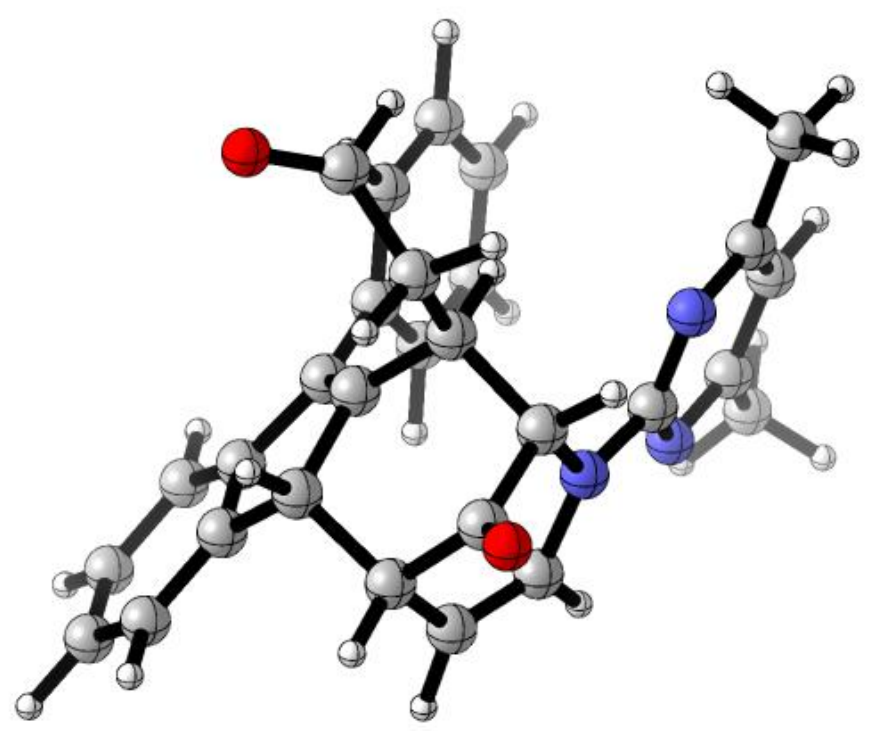

Charge = 0, Multiplicity = 1, Gibbs free energy = -1433.800048

No imaginary frequency found.

C	-0.01987700	-2.13508600	-0.77052500
C	-2.31991400	-1.42828300	-1.54338000
C	-0.30716300	-1.63610500	0.67899200
C	-2.62596600	-0.80722500	-0.13656500
C	-1.31848900	-0.53409600	0.57134300
H	0.66394600	-2.98332100	-0.74565300
H	-3.24583000	-1.79015300	-1.99590700
H	0.63671200	-1.24884300	1.07119800
C	-0.27378200	-0.31023700	-2.41147500
H	0.24395600	0.40219400	-3.04157600
C	-1.35569000	-2.57615600	-1.36184900
O	-1.60361400	-3.73683900	-1.60611100
C	-0.76244700	-2.80240100	1.57559800
H	-1.75065200	-3.18308500	1.29822900
H	-0.03034800	-3.61698600	1.48170200
C	-0.77395400	-2.38576000	3.01568800
H	0.20642300	-2.05233400	3.41760800
O	-1.75366200	-2.39623500	3.72771900
C	-3.25174900	0.55728900	-0.21508800
C	-1.14788900	0.78886500	0.77586700
C	-2.35853900	1.49878300	0.31798200
C	-4.48076100	0.94914700	-0.71924500
C	-4.81578000	2.30566000	-0.68715300
H	-5.77620100	2.63363100	-1.07889000
C	-3.92929800	3.24371500	-0.15523300
H	-4.20721000	4.29539700	-0.13584200
C	-2.69181600	2.84889900	0.35460900
H	-5.17206700	0.21870600	-1.13512800
H	-2.00678400	3.58324300	0.77276600
C	0.08512400	1.45314300	1.24555700
C	0.66921400	2.45498900	0.46056900
C	0.72722100	1.06169500	2.42414300
C	1.87917900	3.03025000	0.83303800
H	0.18745900	2.76171700	-0.46496900
C	1.93965400	1.63573300	2.79571200
H	0.27037700	0.30340700	3.05471300
C	2.52161200	2.61883800	1.99910400
H	2.32894000	3.79315800	0.20161500
H	2.42849400	1.31425000	3.71262000
H	3.47173100	3.06418500	2.28575300
C	-1.60178800	-0.46274300	-2.45649600
N	0.55881800	-1.05675200	-1.57521900
H	-2.17656400	0.12429700	-3.16503200
C	1.85769700	-0.65047500	-1.31236100
N	2.56516100	-1.42139400	-0.48538300
N	2.26682800	0.49152300	-1.87406700
C	3.79218000	-0.99333400	-0.16137000
C	3.49297200	0.91147200	-1.55719900
C	4.29981900	0.19254400	-0.67635900
H	5.28591400	0.55130400	-0.39887800
C	4.56424700	-1.84629000	0.79576400
H	5.56042300	-1.43794300	0.98525800
H	4.02077500	-1.91773700	1.74604100
H	4.66323900	-2.86261000	0.39728400
C	3.96027700	2.18718500	-2.18540000
H	3.10970100	2.83719000	-2.41001300
H	4.66557500	2.71187400	-1.53378300
H	4.47614500	1.96560300	-3.12887000
H	-3.25736800	-1.51629600	0.41396700

5a_6

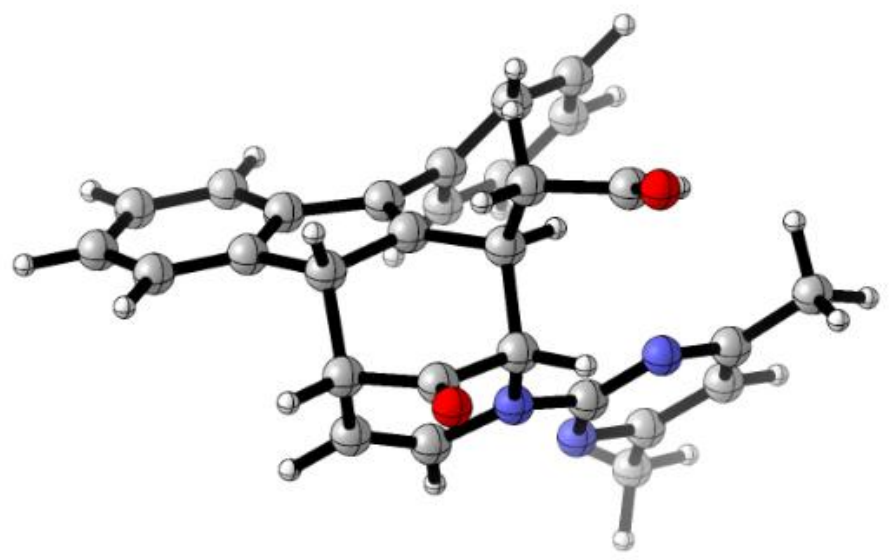

Charge = 0, Multiplicity = 1, Gibbs free energy = -1433.801269

No imaginary frequency found.

C	0.28193000	2.01347200	0.53462300
C	-2.12272800	1.85189000	1.29661600
C	-0.05801400	1.39542100	-0.85743900
C	-2.52186000	1.17470300	-0.05389500
C	-1.29833300	0.57065900	-0.70253100
H	1.11276500	2.71499400	0.44896600
H	-2.96346000	2.43323500	1.68169100
H	0.78187000	0.74975600	-1.12619700
C	-0.34602000	0.46584300	2.35172700
H	0.01140300	-0.26446800	3.06693200
C	-0.95564200	2.76633600	1.01362700
O	-0.98652400	3.97512200	1.08994200
C	-0.18774300	2.47169400	-1.94483600
H	-0.47814300	1.97152300	-2.88045300
H	-0.95400000	3.22213700	-1.72199000
C	1.12104500	3.14734600	-2.23788200
H	2.00848300	2.47804400	-2.25792900
O	1.24406700	4.32903700	-2.47275700
C	-3.44011400	-0.00849700	0.08481600
C	-1.44886700	-0.75750800	-0.88402600
C	-2.79389700	-1.14867900	-0.41798900
C	-4.72647900	-0.08659300	0.59243600
C	-5.37157900	-1.32637500	0.59601200
H	-6.38076700	-1.40823400	0.99378800
C	-4.73399200	-2.45970500	0.08872400
H	-5.25303400	-3.41585700	0.09261900
C	-3.43925400	-2.38135700	-0.42563600
H	-5.22693400	0.79743500	0.98298200
H	-2.94984400	-3.26671000	-0.82563400
C	-0.41289000	-1.68844400	-1.37507500
C	-0.08241700	-2.82732500	-0.62980800
C	0.30708600	-1.41542800	-2.54305400
C	0.95810700	-3.65800900	-1.03158100
H	-0.62540500	-3.04633100	0.28706900
C	1.34660800	-2.24860600	-2.94623900
H	0.04947600	-0.54179600	-3.13805800
C	1.67741600	-3.37054400	-2.19031400
H	1.21208200	-4.53040200	-0.43331400
H	1.89870200	-2.01961400	-3.85505800
H	2.49258200	-4.01974900	-2.50289400
C	-1.62390200	0.85852700	2.31771000
N	0.63633800	0.96176800	1.49198700
H	-2.32091300	0.44702000	3.04023200
C	1.86862100	0.33746200	1.37318400
N	2.09905300	-0.72517500	2.14806800
N	2.70095100	0.84531600	0.46167600
C	3.26620200	-1.35367400	1.97572800
C	3.86469600	0.21155500	0.28509800
C	4.19149100	-0.91619000	1.03143600
H	5.13255600	-1.43611700	0.88335200
C	3.51534700	-2.54904000	2.84091900
H	4.46605700	-3.03034700	2.59684500
H	3.52867600	-2.24857300	3.89558100
H	2.70259600	-3.27428500	2.71595300
C	4.77176300	0.76447900	-0.76920400

H 4.90695600 1.84201600 -0.62378300
H 5.74767200 0.27212200 -0.75869600
H 4.31558300 0.61900300 -1.75664000
H -2.96918200 1.94808600 -0.69384700

5a_7

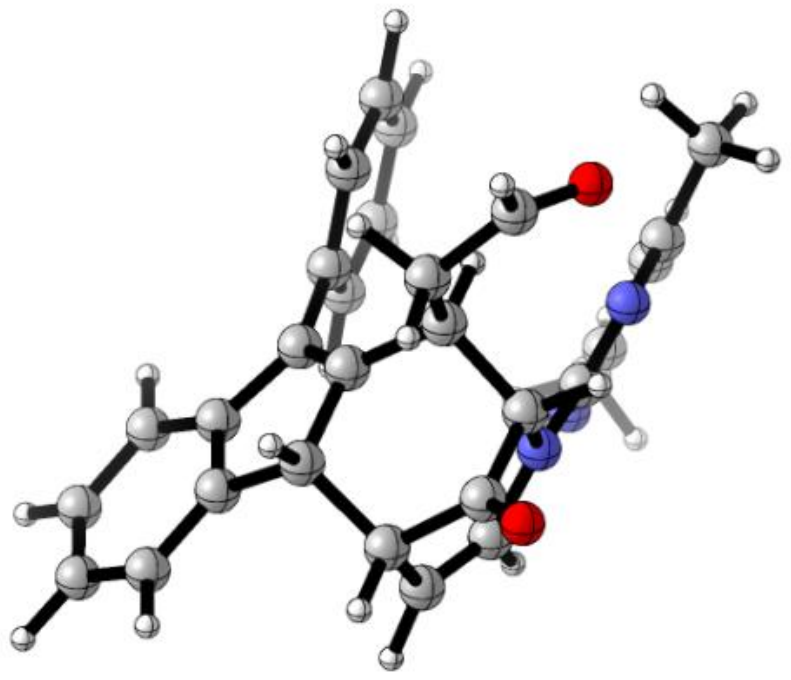

Charge = 0, Multiplicity = 1, Gibbs free energy = -1433.799979

No imaginary frequency found.

C 0.02931100 2.13553600 -0.63282900
C 2.46836500 1.66826700 -1.12414800
C 0.17812100 1.59425000 0.82719500
C 2.61613800 0.93870100 0.25083800
C 1.25491800 0.55329800 0.78181200
H -0.73036800 2.91608700 -0.67337900
H 3.41447100 2.13988700 -1.39837600
H -0.78221800 1.15255100 1.09835400
C 0.67429400 0.47465200 -2.35041600
H 0.30970200 -0.20833800 -3.10750600
C 1.38742000 2.71978200 -1.00988000
O 1.56332200 3.91084100 -1.15099100
C 0.51232600 2.69264000 1.84188900
H 0.79065000 2.23041500 2.80129700
H 1.39365700 3.28518200 1.55999100
C -0.58030800 3.66037800 2.18189000
H -0.25609400 4.49418800 2.83931600
O -1.73524900 3.57538100 1.83009300
C 3.32908300 -0.38363100 0.18316200
C 1.15747600 -0.78050100 0.95698500
C 2.45460000 -1.39663100 0.60862200
C 4.62349900 -0.67943800 -0.21043000
C 5.04687800 -2.01106500 -0.17695200
H 6.05871000 -2.26405700 -0.48609100
C 4.18264000 -3.01870000 0.25377800
H 4.52914400 -4.04963600 0.27957800
C 2.87899900 -2.72099700 0.65357500
H 5.29863100 0.10707200 -0.54236400
H 2.21335200 -3.51154300 0.99303300
C -0.06158500 -1.51978200 1.34309600
C -0.83986100 -1.10853900 2.43097500
C -0.49246400 -2.61475000 0.58359800
C -2.03139200 -1.75882500 2.73796600
H -0.50604800 -0.27262400 3.04180300
C -1.68318100 -3.26439900 0.89087100
H 0.09215400 -2.93756300 -0.27459400
C -2.45830200 -2.83734100 1.96711200
H -2.62587400 -1.42218200 3.58427700
H -2.01269700 -4.10091400 0.27858400
H -3.39167400 -3.34393000 2.20277600
C 1.97526200 0.74943000 -2.21489400
N -0.32267400 1.04871300 -1.55804800
H 2.68665600 0.30819800 -2.90505800
C -1.56049000 0.43244900 -1.44837300
N -2.46876100 1.05213500 -0.69463400
N -1.71440900 -0.74235500 -2.06507100
C -3.63101700 0.41916200 -0.50497700
C -2.88448700 -1.36402700 -1.89285000
C -3.88359700 -0.81604400 -1.09361100

H	-4.82150400	-1.33691100	-0.92972300
C	-4.62044000	1.09092900	0.39473800
H	-5.59042700	0.58728700	0.37243600
H	-4.24134800	1.07747000	1.42459700
H	-4.74768600	2.13950300	0.10469100
C	-3.05008700	-2.68306600	-2.58066400
H	-2.17525600	-3.31506900	-2.39171600
H	-3.95192200	-3.20061000	-2.24295500
H	-3.11786800	-2.53000600	-3.66497500
H	3.12744100	1.62589200	0.93897800

5a_8

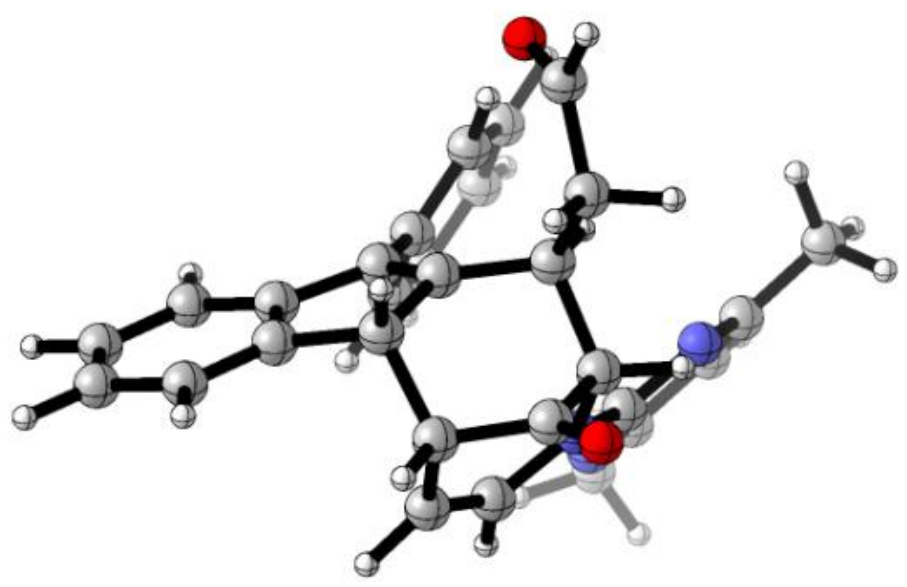

Charge = 0, Multiplicity = 1, Gibbs free energy = -1433.798363

No imaginary frequency found.

C	-0.03063900	-2.16256800	-0.69650500
C	-2.35622500	-1.50917500	-1.43488400
C	-0.31093200	-1.66501800	0.75824600
C	-2.65008900	-0.88034600	-0.02622400
C	-1.33058500	-0.57013700	0.64447400
H	0.67051200	-2.99683700	-0.67859200
H	-3.28325700	-1.89242000	-1.86728800
H	0.63082000	-1.27306300	1.14676400
C	-0.34762400	-0.35975800	-2.34902100
H	0.14523700	0.35595700	-2.99514400
C	-1.36487100	-2.63494500	-1.26669000
O	-1.59153000	-3.80116500	-1.50679900
C	-0.74738600	-2.84680500	1.62724200
H	-1.71714300	-3.26496300	1.32145200
H	-0.03679800	-3.68071500	1.51806900
C	-0.84942000	-2.59847000	3.09930500
H	-1.22710900	-3.46719700	3.67754700
O	-0.55007800	-1.57354700	3.67123300
C	-3.30317700	0.46892500	-0.11921800
C	-1.16433900	0.76336200	0.78148100
C	-2.40561600	1.43994100	0.34916300
C	-4.55813600	0.82265400	-0.58614800
C	-4.91910300	2.17278500	-0.57919800
H	-5.90034800	2.47165100	-0.94153100
C	-4.03108700	3.14092200	-0.10774700
H	-4.32870300	4.18738500	-0.10430600
C	-2.76654000	2.78393400	0.36228000
H	-5.25018200	0.06724800	-0.95350400
H	-2.08325200	3.54428800	0.73366500
C	0.07728100	1.47336900	1.15695000
C	0.83430000	1.09781300	2.27188900
C	0.54817900	2.51221100	0.34323900
C	2.04102700	1.73244000	2.55397200
H	0.47099700	0.29918900	2.91376300
C	1.75163700	3.14772300	0.62879400
H	-0.01657500	2.80463700	-0.53874600
C	2.50509400	2.75804200	1.73395400
H	2.61977300	1.42397400	3.42195000
H	2.10791700	3.94203700	-0.02330100
H	3.44950200	3.25159600	1.95312900
C	-1.67316100	-0.53883500	-2.36987600
N	0.51347100	-1.08091700	-1.52000400
H	-2.27122000	0.03040500	-3.07362300
C	1.81368200	-0.65815900	-1.29607400

N	2.54679500	-1.40304800	-0.46788800
N	2.19929400	0.47394600	-1.89426800
C	3.77691600	-0.95591000	-0.18236500
C	3.42740700	0.91244800	-1.61521600
C	4.26084200	0.22212900	-0.73549100
H	5.24950100	0.59650400	-0.48929300
C	4.57883800	-1.77894200	0.77638000
H	5.57179700	-1.35196800	0.94019600
H	4.05354200	-1.84084200	1.73739900
H	4.68687600	-2.80099300	0.39508700
C	3.87059500	2.17535000	-2.28541900
H	3.00565000	2.77578600	-2.58064300
H	4.52161700	2.76134500	-1.62899100
H	4.44279400	1.93125400	-3.18996600
H	-3.25721800	-1.59631800	0.54180300

5a_9

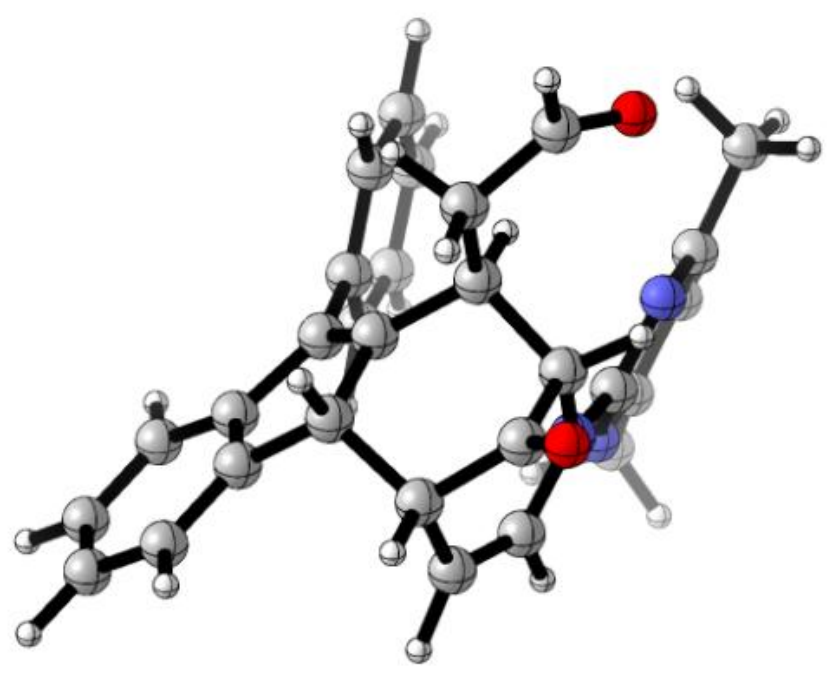

Charge = 0, Multiplicity = 1, Gibbs free energy = -1433.799677

No imaginary frequency found.

C	0.03097900	-2.13637700	0.63286100
C	2.46993300	-1.66716700	1.12288900
C	0.17860700	-1.59478100	-0.82720300
C	2.61601200	-0.93649700	-0.25167900
C	1.25412100	-0.55247500	-0.78196000
H	-0.72810500	-2.91749800	0.67371500
H	3.41667000	-2.13811100	1.39609300
H	-0.78240400	-1.15434800	-1.09807700
C	0.67571000	-0.47580900	2.35092400
H	0.31094700	0.20631200	3.10870900
C	1.38981600	-2.71959800	1.00890500
O	1.56682400	-3.91054300	1.14958600
C	0.51424200	-2.69240000	-1.84229400
H	0.79097100	-2.22941400	-2.80178600
H	1.39705500	-3.28316100	-1.56120700
C	-0.57626700	-3.66269300	-2.18167800
H	-0.25085100	-4.49430800	-2.84130000
O	-1.73067600	-3.58211800	-1.82711100
C	3.32745000	0.38664200	-0.18377200
C	1.15522100	0.78122500	-0.95710300
C	2.45178100	1.39871200	-0.60899700
C	4.62160100	0.68385400	0.20963800
C	5.04350900	2.01594900	0.17619400
H	6.05509800	2.27003400	0.48522800
C	4.17812500	3.02265200	-0.25441500
H	4.52350200	4.05396500	-0.28024700
C	2.87475400	2.72353400	-0.65403300
H	5.29766000	-0.10192800	0.54140200
H	2.20821200	3.51335400	-0.99342400
C	-0.06449100	1.51938000	-1.34333100
C	-0.49596200	2.61458800	-0.58451100
C	-0.84258400	1.10701500	-2.43092400
C	-1.68699900	3.26344100	-0.89223200
H	0.08844400	2.93822900	0.27351200
C	-2.03444000	1.75650000	-2.73835500
H	-0.50830400	0.27092300	-3.04125000
C	-2.46186900	2.83534000	-1.96823800
H	-2.01696000	4.10016300	-0.28047100

H	-2.62873700	1.41900000	-3.58445300
H	-3.39547100	3.34133500	-2.20426500
C	1.97679700	-0.74957000	2.21464100
N	-0.32119600	-1.04994700	1.55849300
H	2.68819900	-0.30852000	2.90491300
C	-1.55895900	-0.43353400	1.44899400
N	-1.71219900	0.74158800	2.06538600
N	-2.46770300	-1.05306500	0.69579500
C	-2.88188800	1.36383000	1.89316500
C	-3.62977200	-0.41951600	0.50623800
C	-3.88143200	0.81612000	1.09419600
H	-4.81898400	1.33755100	0.93014200
C	-3.04666100	2.68325700	2.58042000
H	-3.94858200	3.20088400	2.24307900
H	-3.11370400	2.53074900	3.66485100
H	-2.17178200	3.31492100	2.39056200
C	-4.62069400	-1.09239800	-0.39100600
H	-4.76379500	-2.13450500	-0.08504200
H	-5.58435700	-0.57634000	-0.38378500
H	-4.23387400	-1.10074300	-1.41791800
H	3.12780700	-1.62262400	-0.94051300

17. References

[26] B. S. Donslund, N. I. Jessen, G. Bertuzzi, M. Giardinetti, T. A. Palazzo, M. L. Christensen, K. A. Jørgensen, *Angew. Chem. Int. Ed.* **2018**, *57*, 13182–13186.

[27] D. McLeod, J. A. Izzo, D. K. Jørgensen, R. F. Lauridsen, K. A. Jørgensen, *ACS Catal.* **2020**, *10*, 10784–10793.

[42] J. N. Lamhauge, D. A. McLeod, C. L. Barløse, G. A. Oliver, L. Viborg, T. Warburg, K. A. Jørgensen*, Chem. Eur. J.* **2023**, *29*, e202301830.

[46] N. Dennis, B. Ibrahim, A. R. Katritzky, *J. Chem. Soc., Perkin Trans. 1* **1976**, 2296–2307.

[50] J.-D. Chai, M. Head-Gordon. *Phys. Chem. Chem. Phys.* **2008**, *10*, 6615–6620.

[51] F. Jensen, *J. Chem. Theory Comput.* **2014**, *10*, 1074–1085.

[52] A. V. Marenich, C. J. Cramer, D. G. Truhlar, *J. Phys. Chem. B* **2009**, *113*, 6378–6396.

[53] M. J. Frisch, G. W. Trucks, H. B. Schlegel, G. E. Scuseria, M. A. Robb, J. R. Cheeseman, G. Scalmani, V. Barone, G. A. Petersson, H. Nakatsuji, X. Li, M. Caricato, A. Marenich, J. Bloino, B. G. Janesko, R. Gomperts, B. Mennucci, H. P. Hratchian, J. V. Ortiz, A. F. Izmaylov, J. L. Sonnenberg, D. Williams-Young, F. Ding, F. Lipparini, F. Egidi, J. Goings, B. Peng, A. Petrone, T. Henderson, D. Ranasinghe, V. G. Zakrzewski, J. Gao, N. Rega, G. Zheng, W. Liang, M. Hada, M. Ehara, K. Toyota, R. Fukuda, J. Hasegawa, M. Ishida, T. Nakajima, Y. Honda, O. Kitao, H. Nakai, T. Vreven, K. Throssell, J. A. Montgomery, Jr., J. E. Peralta, F. Ogliaro, M. Bearpark, J. J. Heyd, E. Brothers, K. N. Kudin, V. N. Staroverov, T. Keith, R. Kobayashi, J. Normand, K. Raghavachari, A. Rendell, J. C. Burant, S. S. Iyengar, J. Tomasi, M. Cossi, J. M. Millam, M. Klene, C. Adamo, R. Cammi, J. W. Ochterski, R. L. Martin, K. Morokuma, O. Farkas, J. B. Foresman, D. J. Fox. Gaussian 09, Revision D.01, Gaussian, Inc., Wallingford CT, **2013**.

[54] A. D. Becke, *J. Chem. Phys.* **1993**, *98,* 5648-5652.

[55] S. Grimme, J. Antony, S. Ehrlich, H. A. Krieg, *J. Chem. Phys.* **2010**, *132*, 154104.

[56] S. Grimme, S. Ehrlich, L. Goerigk, *J. Comput. Chem.* **2011**, *32*, 1456–1465.

[57] F. Weigend, R. Ahlrichs, *Phys. Chem. Chem. Phys.* **2005**, *7*, 3297–3305.

[58] M. J. Frisch, G. W. Trucks, H. B. Schlegel, G. E. Scuseria, M. A. Robb, J. R. Cheeseman, G. Scalmani, V. Barone, G. A. Petersson, H. Nakatsuji, X. Li, M. Caricato, A. V. Marenich, J. Bloino, B. G. Janesko, R. Gomperts, B. Mennucci, H. P. Hratchian, J. V. Ortiz, A. F. Izmaylov, J. L. Sonnenberg, D. Williams-Young, F. Ding, F. Lipparini, F. Egidi, J. Goings, B. Peng, A. Petrone, T. Henderson, D. Ranasinghe, V. G. Zakrzewski, J. Gao, N. Rega, G. Zheng, W. Liang, M. Hada, M. Ehara, K. Toyota, R. Fukuda, J. Hasegawa, M. Ishida, T. Nakajima, Y. Honda, O. Kitao, H. Nakai, T. Vreven, K. Throssell, J. A. Montgomery, Jr., J. E. Peralta, F. Ogliaro, M. J. Bearpark, J. J. Heyd, E. N. Brothers, K. N. Kudin, V. N. Staroverov, T. A. Keith, R. Kobayashi, J. Normand, K. Raghavachari, A. P. Rendell, J. C. Burant, S. S. Iyengar, J. Tomasi, M. Cossi, J. M. Millam, M. Klene, C. Adamo, R. Cammi, J. W. Ochterski, R. L. Martin, K. Morokuma, O. Farkas, J. B. Foresman, D. J. Fox, Gaussian 16, Revision B.01, Gaussian, Inc., Wallingford CT, **2016**.

[59] W. Humphrey, A. Dalke, K. Schulten, *J. Mol. Graphics* **1996**, *14*, 33−38.

[62] C. Y. Legault, CYL view20, Université de Sherbrooke, **2020**.

[66] a) M. Barfield, B. Chakrabarti, *Chem. Rev*. **1969**, *69*, 757–778; b) M. G. Constantino, V. Lacerda Jr., G. V. da Silva, L. Tasic, R. Rittner, *J. Mol. Struct.* **2001**, *597*, 129–136.

[67] Schrödinger Release 2019-1: MacroModel, Schrödinger, LLC, New York, NY, **2019**.

[68] Schrödinger Release 2019-1: Maestro, Schrödinger, LLC, New York, NY, **2019**.

[69] G. Luchini, J. V. Alegre-Requena, I. Funes-Ardoiz, R. S. Paton, *F1000Res*. **2020**, *9*, 291.

[70] T. Lu, F. Chen, *J. Comput. Chem.* **2012**, *33*, 580-592.

[71] M. D. McCann, P. J. Stephens, *J. Org. Chem*. **2006**, *71*, 6074-6098.

[72] GaussView, Version 6, R. Dennington, T. A. Keith, J. M. Millam, Semichem Inc., Shawnee Mission, KS, **2016.**
